# In Silico Analysis of Fungal and Chloride-Dependent α-Amylases within the Family GH13 with Identification of Possible Secondary Surface-Binding Sites

**DOI:** 10.3390/molecules26185704

**Published:** 2021-09-21

**Authors:** Zuzana Janíčková, Štefan Janeček

**Affiliations:** 1 Department of Biology, Faculty of Natural Sciences, University of Ss. Cyril and Methodius, SK-91701 Trnava, Slovakia; zuzana.janickova1@gmail.com; 2 Laboratory of Protein Evolution, Institute of Molecular Biology, Slovak Academy of Sciences, SK-84551 Bratislava, Slovakia

**Keywords:** α-amylase family GH13, fungal α-amylases, chloride-dependent α-amylases, GH13 subfamilies, surface-binding sites, unique sequence features, evolutionary relationships

## Abstract

This study brings a detailed bioinformatics analysis of fungal and chloride-dependent α-amylases from the family GH13. Overall, 268 α-amylase sequences were retrieved from subfamilies GH13_1 (39 sequences), GH13_5 (35 sequences), GH13_15 (28 sequences), GH13_24 (23 sequences), GH13_32 (140 sequences) and GH13_42 (3 sequences). Eight conserved sequence regions (CSRs) characteristic for the family GH13 were identified in all sequences and respective sequence logos were analysed in an effort to identify unique sequence features of each subfamily. The main emphasis was given on the subfamily GH13_32 since it contains both fungal α-amylases and their bacterial chloride-activated counterparts. In addition to in silico analysis focused on eventual ability to bind the chloride anion, the property typical mainly for animal α-amylases from subfamilies GH13_15 and GH13_24, attention has been paid also to the potential presence of the so-called secondary surface-binding sites (SBSs) identified in complexed crystal structures of some particular α-amylases from the studied subfamilies. As template enzymes with already experimentally determined SBSs, the α-amylases from *Aspergillus niger* (GH13_1), *Bacillus halmapalus*, *Bacillus paralicheniformis* and *Halothermothrix orenii* (all from GH13_5) and *Homo sapiens* (saliva; GH13_24) were used. Evolutionary relationships between GH13 fungal and chloride-dependent α-amylases were demonstrated by two evolutionary trees—one based on the alignment of the segment of sequences spanning almost the entire catalytic TIM-barrel domain and the other one based on the alignment of eight extracted CSRs. Although both trees demonstrated similar results in terms of a closer evolutionary relatedness of subfamilies GH13_1 with GH13_42 including in a wider sense also the subfamily GH13_5 as well as for subfamilies GH13_32, GH13_15 and GH13_24, some subtle differences in clustering of particular α-amylases may nevertheless be observed.

## 1. Introduction

α-Amylase (EC 3.2.1.1) is a starch hydrolase catalysing the hydrolysis of α-1,4-glycosidic linkages in starch, glycogen and related α-glucans into maltooligosaccharides that, depending on the source of the enzyme, may vary in their actual lengths [1]. Since α-amylases are produced by most organisms from *Bacteria*, *Archaea* and *Eucarya* [1,2,3,4,5], due to genome sequencing projects, thousands of their sequences have become available in the sequence-based classification of carbohydrate-active enzymes, i.e., the CAZy database [6]. Interestingly, the α-amylase enzyme specificity is obviously present in more glycoside hydrolase (GH) families: (i) the main and the largest α-amylase family GH13 forming with families GH70 and GH77 the clan GH-H; (ii) the second and the smaller α-amylase family GH57 exhibiting a relatedness with the little family GH119; and (iii) eventually also the family GH126 [1,6,7,8,9,10].

The α-amylase family GH13 belongs to the largest GH families within the entire Carbohydrate-Active enZymes (CAZy) database; the number of sequences, at the September 2021 update, being more than 120 thousand members ([6]; http://www.cazy.org/GH13.html (accessed on 10 September 2021)). The family, however, is a polyspecific one, i.e., in addition to α-amylase, it covers also pullulanase, isoamylase, cyclodextrin glucanotransferase, glucan branching and debranching enzymes and others whose number has already exceeded 30 different enzyme specificities [1,6,7,11,12,13,14,15]. All GH13 members exhibit several exclusive characteristics, such as the presence of a TIM-barrel catalytic domain with 4–7 seven conserved sequence regions (CSRs), sharing the catalytic machinery of Asp (at the strand β4), Glu (β5) and Asp (β7) as the catalytic nucleophile, proton donor and transition-state stabilizer, respectively, and employing the retaining reaction mechanism [1,7,12,13,14,15,16,17,18]. Although some sequentially closely related groups of enzymes were suggested to define the subfamilies of oligo-1,6-glucosidase and neopullulanase with an intermediary group in 2002 [19], the entire family has been officially divided into 35 GH13 subfamilies by CAZy curators in 2006 [20]. Currently, there are 44 GH13 subfamilies [6,21]; their number is expected to rise even further in the future [22,23].

Of the 44 GH13 subfamilies, 15 can be considered as those comprising the α-amylase enzyme specificity: GH13_1, 5, 6, 7, 15, 19, 24, 27, 28, 32, 36, 37, 41, 42 and 43 [1,6,7,20,21]. Some of them reflect also taxonomic differences known from the time when the family was originally established [1,7]. Thus, for example: (i) subfamilies GH13_1, 6, 7, 15 and 24 have long time been recognized as those containing typical fungal, plant, archaeal, insect and animal α-amylases, respectively; (ii) subfamilies GH13_5 and 28, have both been treated as bacterial ones comprising, respectively, liquefying and saccharifying α-amylases from bacilli; and (iii) with regard to subfamilies GH13_32 and 36, the former has originally been reserved for α-amylases from actinobacteria closely related to animal counterparts, whereas the latter has been devoted to the so-called intermediary group of α-amylases with the extended enzyme specificity including the activity to cyclodextrins [24,25,26,27].

Most of the above-mentioned GH13 subfamilies have later expanded, e.g., both typical plant and archaeal α-amylase subfamilies GH13_6 and 7 contain today also various bacterial homologues [6,7] and fungal α-amylases have recently been found in four GH13 subfamilies, i.e., in addition to the subfamily GH13_1, they have also been classified in subfamilies GH13_5, 32 and even 42 [6,28,29,30]. In this respect, the presence of fungal α-amylases in the subfamily GH13_32, revealed originally in 2013 [29], is of a special interest because this subfamily has been best known as that grouping the α-amylases from Actinomycetes, which exhibit close sequence-structural similarities with α-amylases from insects (GH13_15) and animals (GH13_24) [21,25,26]. Moreover, the most deeply studied member of the subfamily GH13_32, the α-amylase from *Pseudoalteromonas haloplanktis* [31], which was revealed to resemble closely even the α-amylases from mammals [24,32]. The resemblance has later been confirmed also with regard to binding chloride anion [33,34,35], the feature otherwise typical for insect and animal α-amylases from both subfamilies GH13_15 and 24 [36,37,38,39,40,41,42].

The second characteristic feature of α-amylases that has attracted a significant interest is represented by the presence of some secondary surface-binding sites (SBSs) situated on the catalytic TIM-barrel domain [43]. These SBSs are different from those located within a distinct starch-binding domain (SBD), which exists separately from the catalytic domain and have been classified as carbohydrate-binding module (CBM) families [6,44]. They should improve the substrate adsorption, but in contrast to SBDs, they are not easily recognized at the sequence level [43,44,45,46,47,48]. The SBSs have thus been revealed in three-dimensional structures solved as complexes with various ligands in several α-amylases, e.g., *Aspergillus niger* from the subfamily GH13_1 [49], *Bacillus halmapalus*, *Bacillus paralicheniformis* and *Halothermothrix orenii* (AmyB) from the subfamily GH13_5 [50,51,52], and pig (pancreas) and human (saliva) from the subfamily GH13_24 [53,54].

This study therefore delivers the in silico analysis of 268 α-amylase sequences from subfamilies GH13_1, 5, 15, 24, 32 and 42. It was based on the above-mentioned phenomenon of close relatedness of bacterial α-amylases from Actinomycetes from the subfamily GH13_32 with their animal counterparts from insects and mammals from subfamilies GH13_15 and GH13_24, respectively, which are chloride-activated, i.e., chloride-dependent α-amylases. Since the dependence on chloride anion has already been confirmed for some bacterial α-amylases in the subfamily GH13_32, it would be interested to investigate whether or not also the fungal α-amylases classified in this subfamily may require the chloride for their proper functioning. In addition to this main goal of the present study, a detailed sequence-structural comparison with respect to the eventual presence of SBSs within the entire set of 268 studied sequences of α-amylases has also been performed.

## 2. Results and Discussion

The present in silico study is a continuation of the previous bioinformatics analysis delivering an exhaustive comparison of 522 sequences of α-amylase mostly of fungal origin classified in three subfamilies GH13_1, GH13_5 and GH13_32 [30]. Here, in addition to the three GH13 fungal subfamilies mentioned above, also α-amylase sequences from the family GH13_42 were included. However, since the subfamily GH13_32 covers also the so-called animal-like α-amylases from actinobacteria [29], the two GH13 subfamilies with typical chloride-activated animal α-amylases, GH13_15 and GH13_24 [24,26,33,35], were taken into the comparison. Overall, 268 α-amylase sequences were thus studied (Table 1).

### 2.1. Sequence Logos of α-Amylases from Different GH13 Subfamilies

**Figure 1:**
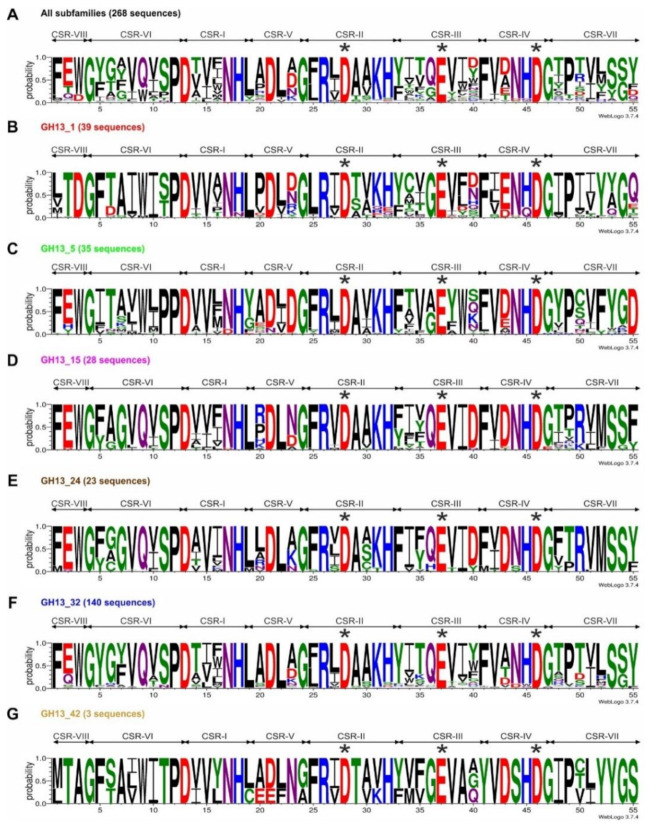
Sequence logos of α-amylases from studied GH13 subfamilies. Logos for (**A**) all the six studied subfamilies (268 sequences); (**B**) subfamily GH13_1 (39 sequences); (**C**) subfamily GH13_5 (35 sequences); (**D**) G13_15 (28 sequences); (**E**) subfamily GH13_24 (23 sequences); (**F**) subfamily GH13_32 (140 sequences); and (**G**) G13_42 (3 sequences). CSR-I, residues 13–18; CSR-II, residues 24–32; CSR-III, residues 33–40; CSR-IV, residues 41–46; CSR-V, residues 19–23; CSR-VI, residues 4–12; CSR-VII, residues 47–55; CSR-VIII, residues 1–3. The catalytic triad, i.e., the catalytic nucleophile (No. 28, aspartic acid in CSR-II), the proton donor (No. 37, glutamic acid in CSR-III) and the transition-state stabilizer (No. 46, aspartic acid in CSR-IV) are indicated by asterisks.

In all 268 collected sequences (Table S1), seven CSRs characteristic for the α-amylase family GH13 [1,18] were identified along with the eighth CSR located around the strand β1 of the catalytic TIM-barrel [24]. Overall, seven sequence logos were constructed (Figure 1), i.e., the first one for all 268 studied sequences, whereas the remaining six logos were prepared for individual subfamilies GH13_1, GH13_5, GH13_15, GH13_24, GH13_32 and GH13_42. Each logo was calculated based on the alignment of all eight CSRs extracted from the alignment of all 268 α-amylase sequences that spanned the sequence segment from the beginning of the strand β1 (CSR-VIII) to the end of the strand β8 (CSR-VII) of the catalytic TIM-barrel domain including the entire domain B (Figure S1).

It is clearly obvious that each GH13 subfamily retains its unique sequence features discriminating the subfamilies from each other (Figure 1). In addition to catalytic machinery, i.e., positions 28 (Asp, catalytic nucleophile), 37 (Glu, catalytic proton donor) and 46 (Asp, transition-state stabilizer), the sequence logo contains also the residues involved in binding the chloride anion by animal and animal-like α-amylases from subfamilies GH13_15, GH13_24 and also GH13_32 (Figure 1)—positions 26 (arginine), 44 (asparagine) and 50 (arginine/lysine) [33,34,35,36,37,38,39,40,41,42].

The unique features within the sequence logo for the three fungal GH13 subfamilies GH13_1, GH13_5 and GH13_32 as well as those shared by two of the three subfamilies have already been described in a detail recently [30]. Here, also the subfamily GH13_42, although it currently contains only one putative fungal α-amylase [6], has been added. It is evident that it also exhibits its own unique sequence features, such as positions 1–3 (CSR-VIII) occupied by residues mTA and positions 53–56 (CSR-VII) with the motif YYGS. Single positions, i.e., threonine, tyrosine and eventually also serine in positions 11, 41 and 44, respectively, may be of interest since these positions in the counterpart α-amylases from other GH13 subfamilies are occupied by different residues (Figure 1). It should be pointed out, however, the importance of the logo for the subfamily GH13_42 is still limited because it was based on 3 sequences only—one putative fungal α-amylase and two characterized members from bacteria [6] (cf. Table 1).

### 2.2. Analysis of Chloride Anion Binding

The chloride anion, which is necessary for allosteric activation of mainly animal (mammalian) α-amylases [36,37,38,39,40,41], is co-ordinated by side-chains of two arginines and one asparagine (the second arginine being substituted by a lysine) that correspond to Arg196, Asn286 and Lys324 in the GH13_32 α-amylase from *Pseudoalteromonas haloplanktis* [33,34,35]. Overall, the substitution of the second arginine by a lysine should, however, in no case eliminate the ability of an α-amylase to bind the chloride anion, since the lysine is, similar to arginine, also positively charged [42].

All these three residues have been in this study identified only in members of subfamilies GH13_15 and GH13_24 as well as in some bacterial α-amylases from the subfamily GH13-32 (Figure S1). The arginine (Arg196 above)—located in the CSR-II (strand β4; in position *i-2* with regard to catalytic nucleophile)—is present in all 268 α-amylases studied here; it represents, in fact, the fourth most invariant residue within the entire family GH13, in addition to the catalytic triad [18]. Concerning the two other residues (Asn286 and Lys324), the asparagine positioned in the CSR-IV (strand β7) three residues before the transition state stabilizer—like the above-mentioned arginine—also belongs to most highly conserved residues in the α-amylase family GH13 [7,14]. Interestingly, aside the typical fungal subfamily GH13_1, this residue is also not invariantly conserved even in the animal α-amylase subfamily GH13_24 (Figure S1), for members of which the activation by chloride anion has been well documented [42]. This asparagine is replaced by a serine in mites from *Acarus siro* ([55]; GenBank: ABL09312.1), *Dermatophagoides pteronyssinus* ([56]; AAD38942.1) and *Euroglyphus maynei* ([56]; AAD38943.1). The third residue involved in chloride binding, which is—for animal α-amylases—typically arginine (Arg321 and Arg337 in GH13_15 *Tenebrio molitor* and GH13_24 human salivary α-amylases, respectively [39,40])—is located in the CSR-VII (strand β8). It is conserved invariantly in GH13_24, almost invariantly in GH13_15, where in the latter it is substituted by a lysine in four cases: *Anthonomus grandis* ([57]; AAN77138.1 and AAN77139.1), *Callosobruchus chinensis* ([58]; BAB72257.1) and *Zabrotes subfasciatus* ([59]; AAF73435.1). It is worth mentioning that in the subfamily GH13_32, it is present only as a lysine and only in a few bacterial cases: *Halomonas meridiana* ([60]; CAB92963.1), *Pseudoalteromonas haloplanktis* ([31]; CAA41481.1), *Pseudomonas* sp. KFCC10818 ([61]; AAA86835.1), *Thermobifida fusca* ([62]; ABF13430.1) and *Thermomonospora curvata* ([63]; CAA41881.1), because none of GH13_32 α-amylase of fungal origin possesses it (Figure S1). Finally, in remaining α-amylase subfamilies from this analysis—GH13_1, GH13_5 and GH13_42—neither arginine, nor lysine is found in the position corresponding with this arginine from the CSR-VII. Based on these observations, it is most probable that fungal α-amylases from any GH13 subfamily (1, 5, 32 and even 42) are not able to bind the chloride, and thus, they cannot be activated by this anion.

### 2.3. Analysis of Surface Binding Sites

The secondary SBSs represent a binding site for a ligand, located outside the active site of the enzyme [43,44,45,46,47,48]. If the entire family GH13 is considered, no special conserving of any amino acid residue involved in an SBS has been observed [49,50,51,52,53,54]. However, within the individual GH13 subfamilies, the preservation may obviously be higher (Figure S1).

As far as the GH13 subfamilies of the present study are concerned, the SBSs were identified in three-dimensional structures of only three of them—GH13_1, GH13_5 and GH13_24. In the subfamily GH13_1, one SBS was revealed in the α-amylase from *Aspergillus niger* formed by residues Tyr382 and Trp385 [49]. In the subfamily GH13_5, SBSs were found in three α-amylases: (i) the α-amylase from *Bacillus halmapalus* possessing three SBSs: SBS-I—residues Trp439 and Trp469, SBS-II—residue Trp347, and SBS-III—residue Tyr363 [50]; (ii) α-amylase from *Bacillus licheniformis* (known also as *Bacillus paralichenifromis*) having one SBS formed by residues Phe257 and Tyr358 [51]; and (iii) the α-amylase AmyB from *Halothermothrix orenii* with three SBSs: SBS-I—residues Tyr460 and Trp488, SBS-II—residues Trp260 and Trp287, and SBS-III - residues Trp306 and W310 [52]. Finally, in the subfamily GH13_24, in addition to two SBSs seen in the α-amylase from pig pancreas [53], four SBSs were demonstrated in the α-amylase from human saliva [54] as follows: SBS-I—residues Tyr276 and Trp284, SBS-II—residues Trp316 and Trp388, SBS-III—residue Trp203, and SBS-IV—residue Trp134.

The residues involved in the above-mentioned experimentally identified SBSs were checked for their correspondences within the particular subfamilies (Figure S1), especially if they are replaced by non-aromatic residues. Thus, in the subfamily GH13_1, both SBS residues Tyr382 and Trp385 of the α-amylase from *Aspergillus niger* [49] were found as conserved in counterpart α-amylases from (Table 2) *Aspergillus awamori* ([64]; GenBank: BAD06002.1), *Aspergillus flavus* ([65]; AAF14264.1), *Aspergillus kawachii* ([66]; BAD01051.1), *Aspergillus oryzae* ([67]; CAA31218.1), *Aspergillus shirousami* ([68]; BAA01255.1), *Fusicoccum* sp. BCC4124 ([69]; ABG48762.1), *Lipomyces spencermartinsiae* ([70]; AAC49622.1) and *Lipomyces starkeyi* ([71]; AAN75021.1). As two aromatic positions, it even might be preserved in other α-amylases, e.g., from *Sclerotinia sclerotiorum* ([72]; ACN82436.1). On the other hand, the α-amylase from *Saccharomycopsis fibuligera* able to degrade raw starch [73] obviously does not possess the corresponding SBS (Table 2). It is of note that in the recently solved three-dimensional structure of the GH13_1 α-amylase from *Cordyceps farinosa* [74] an SBS was identified in domain C with modelled maltose and only one aromatic residue—Phe387—involved in binding. It, however, corresponds with neither of the two aromatic residues forming the SBS-I of the GH13_1 *A. niger* counterpart (Table 2). Interestingly, when the domain C of *C. farinosa* α-amylase was superimposed with the CBM20 of GH15 glucoamylase from *Aspergillus niger* [75], its SBS was seen in a close proximity with the binding site of the CBM20 from the glucoamylase [74].

With regard to the subfamily GH13_5, residues Phe257 and Y358 forming the SBS of the α-amylase from *B. licheniformis* (*paralicheniformis*) [51] were best conserved, although the former residue was observed being substituted by leucine, while the latter one was replaced by valine, arginine, leucine and isoleucine (Table 3). The two residues—Trp347 and Tyr363—from the SBS-II and SBS-III, respectively, of *B. halmapalus* α-amylase [50] were also found as almost totally conserved; the position of Trp347 was in some cases occupied by asparagine, serine and lysine, whereas valine, arginine, leucine and isoleucine were present in that of Tyr363. The residues defining the SBS-I of this α-amylase, i.e., Trp439 and Trp469, belong to less conserved residues because in the position of Trp439, there is often an arginine or threonine and serine, and the Trp469 is substituted rather frequently by asparagine or glutamic acid. Concerning the SBSs from the *H. orenii* α-amylase AmyB [52], the residues Trp260 and Trp287 (SBS-II) and eventually also the Trp306 (SBS-III) rank among the best conserved positions, while the remaining ones were either less or even not conserved at all (Table 3).

The SBSs identified in the human salivary α-amylase [54] were all, in fact, well conserved; the positions of Trp284 (SBS-I), Trp388 (SBS-II) and Trp203 (SBS-III) being found conserved invariantly (Table 4). The other three residues were in a few cases substituted by asparagine, threonine, leucine and cysteine—Trp134 (SBS-IV), threonine and asparagine—Tyr276 (SBS-I) and arginine—Trp316 (SBS-II).

Based on the above analysis, it is possible to assume that the SBSs experimentally identified in tertiary structures of individual α-amylases may really exist in their homologues (Table 2, Table 3 and Table 4; cf. Figure S1). Simultaneously, it is important to take into account the fact that the SBSs, even within a particular GH13 subfamily, may be localized in different parts of α-amylase structure—as seen, e.g., for the three bacterial α-amylases from the subfamily GH13_5 (Table 3). In order to verify the in silico data presented here that concern the conserving the aromatic positions corresponding to real SBSs, it is necessary to confirm the involvement of homologous residues experimentally.

With regard to SBSs, a remark on SBDs, i.e., distinct domains responsible for starch or—in a wider sense—α-glucan binding and classified as various CBM families [44], could be of interest. Some, i.e., not all, fungal and bacterial (from actinomycetes) α-amylases from subfamilies GH13_1 and GH13_32, respectively, contain such an SBD, mostly from the family CBM20 positioned at their C-terminus; or more rarely—mainly some α-amylases of the yeast origin—the SBD of the family CBM21 at their N-terminus [76]. The reasons why some of fungal (yeast) and actinobacterial α-amylases do possess a distinct SBD and why some (others) exhibit rather an isolated SBS (or even more SBSs) are still not completely understood, but those α-amylases having the distinct SBD may represent a unique group of four-domain hydrolases from the family GH13 deserving the future attention [77,78,79]. Concerning the fungal α-amylases from the subfamily GH13_5, these may also represent a special group, sequentially closely similar even to liquefying α-amylases from bacilli [28,80], but most probably involved in synthesizing the α-1,4-oligoglucan primers for synthesis of the outer α-1,3-glucan layer in their cell walls, which behaves as a virulence factor [81,82,83,84,85].

### 2.4. Evolutionary Relatedness of α-Amylases from Fungi and Other Taxa

**Figure 2:**
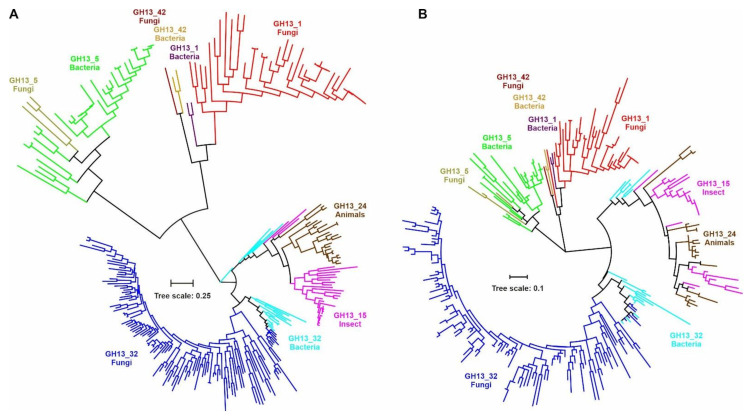
Evolutionary trees of all 268 α-amylases from six studied GH13 subfamilies. The trees are based on the alignment spanning the sequence segment between the strands β1 and β8 of catalytic TIM-barrel (**A**) and covering just CSRs (**B**). For the sake of simplicity, only the branches are shown. The same trees with all the leaves described are presented in supplementary Figure S2. Details concerning all α-amylases compared in the tree as well as their colour codes are given in Table S1.

The phylogenetic relationships between fungal and chloride-dependent α-amylases are shown in their evolutionary trees (Figure 2). Two trees have been prepared: (i) one (Figure 2A) based on the alignment (718 positions) spanning the segment of sequences from beginning of the strand β1 (CSR-VIII) to the end of the strand β8 (CSR-VII) of the catalytic TIM-barrel domain including the entire domain B (cf. Figure S1); and (ii) the other one (Figure 2B) based on the alignment of eight extracted CSRs, i.e., only 55 positions (cf. Figure 1) that may represent the so-called sequence fingerprints of the α-amylases family GH13 [1,18,21,22,23,24,25,26,27,28,29,30]. In order to emphasize just the clustering of individual subfamilies and/or their groups as well as for a higher clarity, the details of all particular 268 sequences (Table S1) were removed in the trees. However, the same evolutionary trees with all the details of individual sources, i.e., mainly the origin and database accession number, are shown in Figure S2.

It is obvious that despite the identical catalytic machinery and substantial similarities within all CSRs (Figure 1), each GH13 subfamily keeps its uniqueness and independency. Nevertheless, it is also clear that α-amylases from the subfamily GH13_42 exhibit a closer evolutionary relatedness with their counterparts from subfamilies GH13_1 and GH13_5 (Figure 2). The subfamily GH13_5 might be of a special importance since it was originally established as a subfamily for liquefying bacterial α-amylases originating mainly from bacilli [1,20], but later some fungal counterparts have been revealed as worth to be included [28]. Of interest is also a hypothetical GH13_5 α-amylase from psychrophilic yeast *Glaciozyma antarctica* exhibiting all sequence-structural features typical for GH13_5 subfamily members, but it still awaits its biochemical characterization [86]. This subfamily has recently expanded its taxonomic coverage even towards *Archaea* [6]. This is rather similar with subfamilies GH13_6 and GH13_7 that were originally defined for α-amylases from plants and hyperthermophilic archaeons, respectively [20,25,87], but currently, both subfamilies contain experimentally confirmed bacterial α-amylases [1,6,7,21,88,89]. Moreover, recent studies focused on the NF-Daqu, i.e., a fermentation starter for a Chinese liquor, have shown [90,91] that a total of 15 GH13 α-amylases—10 from the subfamily GH13_1 and 5 from the subfamily GH13_5—may be involved in the processing, both members of GH13_1 and GH13_5 demonstrating a high synergistic effect on starch degradation [91]. The evolutionary relationships among various fungal α-amylases based on analysis of 85 genomes and focused on taxonomy of fungi have been described in an insightful phylogenetic study in 2012 [92]. Unfortunately, it was performed before the additional GH13 subfamilies with fungal α-amylases (especially the GH13_32) were established; that analysis would therefore deserve to be updated.

Further, fungal α-amylases along with most bacterial α-amylases from actinomycetes—including the one from *Bacillus* sp. 195 ([93]; BAA22082.1)—from the subfamily GH13_32 form their own cluster, too. The additional GH13_32 α-amylases from remaining bacteria—including, on the other hand, also two α-amylases from actinobacteria *Thermobifida fusca* ([62]; GenBank: ABF13430.1) and *Thermomonospora curvata* ([63]; CAA41881.1)—are positioned adjacently to their animal counterparts from subfamilies GH13_15 and GH13_24 to form a larger cluster with them (Figure S2). The two actinobacterial enzymes stand even separately in the evolutionary tree based on the alignment of the sequence segment from beginning of the strand β1 to the end of the strand β8 (Figure 2A). This finding just confirms the original postulate [25] that one may observe some subtle differences in evolutionary trees calculated on alignments of complete sequences and isolated CSRs. The GH13_32 bacterial α-amylases—outside actinobacteria—clustering with the chloride-activated GH13_15 and GH13_24 α-amylases from animals—come from *Halomonas meridiana* ([60]; CAB92963.1), *Pseudoalteromonas haloplanktis* ([31]; CAA41481.1), *Pseudomonas* sp. KFCC10818 ([61]; AAA86835.1) and *Aeromonas hydrophila* ([94]; AAA21016.1).

It is of note that this evolutionary relatedness has been described previously [1,26,29], even before the α-amylase from *P. haloplanktis* [31,32,33,34,35] was assigned to subfamily GH13_32 [6]. What is, however, more important is that all these six bacterial α-amylases closely related to animal counterparts should be able to bind the chloride anion, i.e., to be chloride-activated, since they possess all the three residues identified as crucial Cl^-^-binding residues; with the eventual exception of the *A. hydrophila* α-amylase having in the position of the third residue—being Arg/Lys—a glutamine (cf. Figure S1; CSR-VII).

Concerning the subfamily GH13_15, the α-amylases from the starfish *Asterias rubens* ([95]; AAO13755.1), cockroach *Blattella germanica* ([96]; AAY23288.1) and one of the two from mosquito *Aedes aegypti* ([97]; AAB60935.1) may be of a special interest, since—in the evolutionary tree based on the alignment of the larger sequence segment (from strand β1 to strand β8; Figure 2A; Figure S2A)—they occupy positions at the border of the cluster, common for both GH13_15 and GH13_24, and outside the rest of the subfamily GH13_15 members. Note that in the CSR-based tree (Figure 2B and Figure S2B), sequences from both animal GH13 subfamilies 15 and 24 are rather scattered or mixed to each other within their cluster, indicating thus their mutual very close evolutionary relationships.

### 2.5. Conclusions

The present bioinformatics study has been devoted to—by in silico approaches—the sequences of 268 α-amylases from six subfamilies GH13_1, GH13_5, GH13_15, GH13_24, GH13_32 and GH13_42 of the main α-amylase family GH13. The analyses were focused on fungal α-amylases classified in four of the six studied subfamilies (1, 5, 32 and 42) with a special emphasis on the subfamily GH13_32 containing also bacterial α-amylases closely related to animal counterparts from subfamilies GH13_15 and GH13_24. Since most of animal α-amylases are chloride-activated, including also some bacterial homologues from the subfamily GH13_32, the attention was paid to investigate whether or not fungal α-amylases from the subfamily GH13_32 could bind the chloride anion, too. This is, however, rather impossible since they do not possess the complete triad of required binding residues. The additional goal of the study was to make a sequence comparison concerning the eventual presence of secondary surface-binding sites observed experimentally in several tertiary structures of a few α-amylases from subfamilies GH13_1, GH13_5 and GH13_24 determined as complexes with α-glucans. It was seen that the conservation of these sites cannot be generalized even within a particular GH13 subfamily. With regard to evolutionary relationships, the members of subfamilies GH13_15, GH13_24 and GH13_32 clustered together, whereas members of the subfamily GH13_5 were seemingly more related to those from both GH13_1 and GH13_42 that exhibit very close relatedness to each other. In summary, the present study together with the previous one [30] may add to the overall understanding of evolutionary relationships within the α-amylase family GH13 and the knowledge of properties of α-amylases originating from various taxonomic sources. In a wider perspective, these results may become a base for future experiments aimed at protein engineering and design of α-amylases.

## 3. Materials and Methods

### 3.1. Sequence Collection

The study has dealt with fungal and chloride-dependent GH13 α-amylases classified in the subfamilies GH13_1, GH13_5, GH13_32 and GH13_42 (subfamilies containing the fungal α-amylases) and GH13_15, GH13_24 and GH13_32 (chloride-dependent, respectively, insect, animal and animal-like bacterial α-amylases). Sequences were first taken from the CAZy database [6] focusing on experimentally characterized members of the above-mentioned subfamilies. Since the subfamily GH13_42 has contained only one fungal (eukaryotic) member, the putative fungal α-amylase from *Pecoramyces ruminatium* was added. With regard to the subfamily GH13_32, it has also not covered any biochemically characterized α-amylases originating from fungi (*Eucarya*); putative α-amylase of fungal origin were therefore included. In addition, further fungal α-amylases were obtained by the protein BLAST search ([98]; https://blast.ncbi.nlm.nih.gov/Blast.cgi (accessed on 19 March 2019)), using the complete amino acid sequence of fungal α-amylase from *Pholiota microspora* (UniProt Accession No.: A0A1E1ERR9; [99]), which has been classified in CAZy in the subfamily GH13_32 [6]. The reason for using this fungus as the sequence query for performing the BLAST search was that *P. microspora*, although being a mushroom, possesses in its genome the genes coding for α-amylases from all the three GH13 subfamilies 1, 5 and 32 [6].

All selected sequences had to possess the α-amylase family GH13 characteristic features, such as complete catalytic triad and convincingly typical CSRs. Many eventual sequences exhibited high sequence similarity and even identity (e.g., insect α-amylases from various drosophilas from the subfamily GH13_15); the number of resulting sequences was therefore reduced accordingly (i.e., at the level >90% of sequence identity, only one sequence from that particular group was taken into comparison). Overall, 268 sequences were collected (Table S1) as follows: (i) subfamily GH13_1–39 sequences; (ii) subfamily GH13_5–35 sequences; (iii) subfamily GH13_15–28 sequences; (iv) GH13_24–23 sequences; (v) GH13_32–140 sequences; and (vi) GH13_42–3 sequences.

### 3.2. Sequence Analysis

All 268 studied sequences (Table S1) were obtained from GenBank ([100]; https://www.ncbi.nlm.nih.gov/genbank/) or UniProt ([101]; https://www.uniprot.org/) databases. Their alignment was done using the program Clustal-Omega ([102]; https://www.ebi.ac.uk/Tools/msa/clustalo/) available on the European Bioinformatics Institute’s server. The alignment, which included the necessary but gentle manual tuning with regard to correct alignment of all CSRs [1,18], was manually cut at the N-terminus to start each sequence by the stretch representing the beginning of the strand β1 (CSR-VIII) of the catalytic TIM-barrel domain. With regard to the C-terminus, one version of the alignment was cut just after the end of the strand β8 (CSR-VII), whereas the other one was kept longer bearing also a segment of the domain C succeeding the catalytic TIM-barrel. The former alignment was used for calculating the evolutionary tree and the latter served for identifying the residues involved in secondary SBSs.

Sequence logos of eight CSRs were created using the WebLogo3 online server ([103]; http://weblogo.threeplusone.com/). Seven sequence logos were calculated—the first one for all studied sequences; whereas the additional logos were prepared for the six individual GH13 subfamilies GH13_1, 5, 15, 24, 32 and 42.

### 3.3. Comparison of Chloride- and Surface-Binding Sites

The experimentally determined three-dimensional structures of chloride-activated α-amylases were retrieved from Protein Data Bank (PDB; [104]; https://www.rcsb.org/): (i) subfamily GH13_15—*Tenebrio molitor* ([40]; PDB code: 1JAE); (ii) subfamily GH13_24:—*Sus scrofa* (pancreas; [41]; PDB: 1WO2), *Homo sapiens* (saliva; [39]; PDB: 1SMD); and (iii) subfamily GH13_32—*Pseudoalteromonas haloplanktis* ([35]; PDB: 1JD7). The eventual presence of a chloride-binding site in individual α-amylases from the entire studied set of 268 sequences was evaluated with respect to conserving the residues involved in binding the chloride anion in the above structures by their comparison in the sequence alignment.

The structures of α-amylases solved as complexes with identified additional SBSs were also obtained from PDB [104]: (i) subfamily GH13_1—*Aspergillus niger* ([49]; PDB: 2GUY); (ii) subfamily GH13_5—*Bacillus halmapalus* ([50]; PDB: 2GJP); *Bacillus paralicheniformis* ([51]; PDB: 6TOZ) and *Halothermothrix orenii* ([52]; PDB: 3BC9); and (iii) subfamily GH13_24—*Homo sapiens* (saliva; [54]; PDB: 3BLP). The possible existence of a corresponding SBS in individual α-amylases from the three subfamilies GH13_1, 5 and 24 was deduced from the inspection of the above tertiary structures with experimentally observed SBSs and by the comparison of relevant residues within the sequence alignment.

### 3.4. Evolutionary Relationships

Two evolutionary trees were prepared: (i) one based on the alignment of the segment from the beginning of the strand β1 (CSR-VIII) to the end of the strand β8 (CSR-VII) of the catalytic TIM-barrel domain, including the domain B of all 268 studied sequences; and (ii) the other one based on the alignment of eight selected CSRs for the same sample of all 268 sequences. Both evolutionary trees were calculated as maximum-likelihood trees [105] using the bootstrapping procedure with 500 bootstrap trials [106] implemented in the MEGA software ([107]; https://www.megasoftware.net/) applying default program parameters. The trees were displayed with the program iTOL ([108]; http://itol.embl.de/).

## Figures and Tables

**Table 1:** Summary of 268 fungal and chloride-dependent α-amylases from the family GH13 used in the present study ^1^.

Subfamily	GH13_1		GH13_5		GH13_15		GH13_24		GH13_32		GH13_42	
	P	E	P	E	P	E	P	E	P	E	P	E
	2	37	32	3	0	28	0	23	20	120	2	1
Total	39		35		28		23		140		3	

^1^ The set was created based on sequences classified in the CAZy family GH13 completed by α-amylases obtained by BLAST searches. The “P” and “E” mean *Procarya* and *Eucarya*, respectively.

**Table 2:** Residues corresponding with the SBS from *A. niger* α-amylase *^a^*.

α-Amylase	I	I
AAA85446_*Paenibacillus_polymyxa*	**G**	**R**
CAA49465_*Thermoactinomyces_vulgaris*	**G**	**A**
BAD06003_*Aspergillus_awamori*	**Y**	**D**
BAD06002_*Aspergillus_awamori*	**Y**	**W**
AAF14264_*Aspergillus_flavus*	**Y**	**W**
BAD01051_*Aspergillus_kawachii*	**Y**	**W**
BAA22993_*Aspergillus_kawachii*	**Y**	**D**
EAA64850_*Aspergillus_nidulans*	**Y**	**Y**
AAF17100_*Aspergillus_nidulans*	**S**	**T**
P56271_*Aspergillus_niger*	**Y**	**D**
CAK44871_*Aspergillus_niger*	**Y**	**W**
CAK40249_*Aspergillus_niger*	**V**	**Y**
CAK41088_*Aspergillus_niger*	**S**	**Y**
CAA31218_*Aspergillus_oryzae*	**Y**	**W**
BAA01255_*Aspergillus_shirousami*	**Y**	**W**
AEB80431_*Aspergillus_tubingensis*	**Y**	**D**
BAA12010_*Cryptococcus*_sp_S_2	**Y**	**Q**
ABG48762_*Fusicoccum*_sp_BCC4124	**Y**	**W**
AAO12212_*Lipomyces_kononenkoae*	**D**	**V**
AAC49622_*Lipomyces_spencermartinsiae*	**Y**	**W**
AAN75021_*Lipomyces_starkeyi*	**Y**	**W**
AFD54462_*Malbranchea_cinnamomea*	**T**	**D**
ABF72529_*Ophiostoma_floccosum*	**S**	**S**
EPS26265_*Penicillium_oxalicum*	**S**	**N**
ABO42285_*Phanerochaete_chrysosporium*	**S**	**E**
BAW15173_*Pholiota_microspora*	**K**	**Q**
BAF98616_*Pichia_burtonii*	**T**	**S**
AGJ52081_*Rhizomucor_pusillus*	**T**	**M**
ADL28123_*Rhizopus_oryzae*	**T**	**M**
ADD80242_*Saccharomycopsis_fibuligera*	**S**	**S**
ABS76467_*Saitozyma_flava*	**Y**	**Q**
CAB11471*_Schizosaccharomyces_pombe*	**S**	**H**
CAB40006_*Schizosaccharomyces_pombe*	**S**	**Q**
CAA34162_*Schwanniomyces_occidentalis*	**Y**	**S**
CAA51912_*Schwanniomyces_occidentalis*	**Y**	**D**
ACN82436_*Sclerotinia_sclerotiorum*	**Y**	**Y**
CAA03110_*Thermomyces_lanuginosus*	**S**	**K**
BAG69580_*Trichoderma_viride*	**N**	**K**
CAJ21046_*Valsaria_rubricosa*	**N**	**V**

*^a^* The potential SBSs in α-amylases from the subfamily GH13_1 have been analysed according to SBS-I observed in the three-dimensional structure of the counterpart α-amylase from *Aspergillus niger* ([49]; PDB code: 2GVY): Y402 (Y382) and W405 (W385)—the numbering in parentheses applies if the N-terminal signal peptide is removed), which is highlighted by red-colour inversion. The individual α-amylases are presented by their GenBank accession Nos. and binomial name of the producing organism. The aromatic residues corresponding with the two forming the real SBS-I are highlighted by black inversion.

**Table 3:** Residues corresponding with SBSs of α-amylases from *B. halmapalus*, *B. licheniformis* and *H. orenii ^a^*.

α-Amylase	I	I	II	III	I	I	I	I	II	II	III	III
AWX66236_*Alicyclobacillus*_sp_18711	**T**	**N**	**W**	**Y**	**F**	**Y**	**D**	**I**	**W**	**W**	**W**	**G**
AAQ01675_*Alkalimonas_amylolytica*	**S**	**Y**	**N**	**V**	**F**	**V**	**I**	**F**	**W**	**-**	**N**	**-**
CAL14744_*Anoxybacillus_flavithermus*	**W**	**W**	**W**	**Y**	**F**	**Y**	**P**	**I**	**W**	**W**	**W**	**G**
AEW07376_*Bacillus_acidicola*	**W**	**W**	**W**	**Y**	**F**	**Y**	**P**	**I**	**W**	**W**	**W**	**G**
AAA22191_*Bacillus_amyloliquefaciens*	**R**	**W**	**W**	**Y**	**F**	**Y**	**T**	**T**	**W**	**W**	**W**	**G**
ABY86223_*Bacillus_cereus*	**W**	**W**	**W**	**Y**	**F**	**Y**	**P**	**T**	**W**	**W**	**W**	**G**
CAD26699_*Bacillus_halmapalus*	**W**	**W**	**W**	**Y**	**F**	**Y**	**E**	**I**	**W**	**W**	**W**	**G**
AAA22226_*Bacillus_licheniformis*	**R**	**W**	**W**	**Y**	**F**	**Y**	**T**	**T**	**W**	**W**	**W**	**G**
AEM05860_*Bacillus_licheniformis*	**R**	**W**	**W**	**Y**	**F**	**Y**	**T**	**T**	**W**	**W**	**W**	**G**
AAK00598_*Bacillus_megaterium*	**W**	**W**	**W**	**Y**	**F**	**Y**	**P**	**T**	**W**	**W**	**W**	**G**
AGN35141_*Bacillus_paralicheniformis*	**R**	**W**	**W**	**Y**	**F**	**Y**	**T**	**T**	**W**	**W**	**W**	**G**
AAR68734_*Bacillus*_sp	**W**	**W**	**W**	**Y**	**F**	**Y**	**S**	**I**	**W**	**W**	**W**	**G**
AAA22231_*Bacillus*_sp_707	**W**	**W**	**W**	**Y**	**F**	**Y**	**E**	**I**	**W**	**W**	**W**	**G**
BAF03567_*Bacillus*_sp_JAMB_204	**R**	**W**	**W**	**Y**	**F**	**Y**	**S**	**T**	**Y**	**Y**	**W**	**G**
CAC39917_*Bacillus*_sp_KSM_K38	**W**	**W**	**W**	**Y**	**F**	**Y**	**D**	**I**	**W**	**W**	**W**	**G**
AAB18785_*Bacillus*_sp_MK_716	**W**	**W**	**W**	**Y**	**F**	**Y**	**P**	**I**	**W**	**W**	**W**	**G**
AAA63900_*Bacillus*_sp_TS_23	**W**	**W**	**W**	**Y**	**F**	**Y**	**P**	**I**	**W**	**W**	**W**	**G**
ABW87262_*Bacillus*_sp_YX_YX1	**R**	**W**	**W**	**Y**	**F**	**Y**	**T**	**T**	**W**	**W**	**W**	**G**
AAF00567_*Cytophaga*_sp	**R**	**Y**	**W**	**Y**	**F**	**Y**	**P**	**T**	**W**	**W**	**W**	**G**
AAC74994_*Escherichia_coli*	**T**	**E**	**W**	**V**	**F**	**V**	**P**	**A**	**W**	**H**	**W**	**G**
AFZ41193_*Exiguobacterium*_sp_DAU5	**W**	**W**	**W**	**Y**	**F**	**Y**	**N**	**T**	**W**	**W**	**S**	**G**
AAA22235_*Geobacillus_stearothermophilus*	**W**	**W**	**W**	**Y**	**F**	**Y**	**P**	**I**	**W**	**W**	**W**	**G**
ABX83871_*Geobacillus_thermodenitrificans*	**W**	**R**	**W**	**R**	**F**	**R**	**P**	**I**	**W**	**W**	**F**	**K**
AFC87833_*Geobacillus_thermoleovorans*	**W**	**W**	**W**	**Y**	**F**	**Y**	**P**	**I**	**W**	**W**	**W**	**G**
ACL70573_*Halothermothrix_orenii*	**W**	**Y**	**S**	**V**	**F**	**V**	**Y**	**W**	**W**	**W**	**W**	**W**
CAQ30277_*Nostoc*_sp_PCC_7119	**W**	**W**	**W**	**Y**	**F**	**Y**	**P**	**A**	**Y**	**Y**	**D**	**G**
AAA27110_*Salmonella_typhimurium*	**T**	**E**	**W**	**V**	**F**	**V**	**P**	**A**	**W**	**H**	**W**	**G**
BAA24178_*Streptococcus_equinus*	**R**	**Y**	**W**	**L**	**Y**	**L**	**E**	**I**	**W**	**Y**	**N**	**G**
AAA97431_*Streptococcus_equinus*	**R**	**Y**	**W**	**L**	**Y**	**L**	**E**	**I**	**W**	**Y**	**N**	**G**
AAN59233_*Streptococcus_mutans*	**R**	**W**	**W**	**I**	**Y**	**I**	**E**	**I**	**W**	**Y**	**S**	**G**
CCD30600_uncultured_bacterium	**R**	**Y**	**K**	**I**	**F**	**I**	**Y**	**Y**	**W**	**W**	**Y**	**R**
ALP73597_*Vibrio_alginolyticus*	**W**	**W**	**W**	**Y**	**F**	**Y**	**W**	**A**	**W**	**W**	**W**	**G**
ABK62854_*Histoplasma_capsulatum*	**R**	**Y**	**F**	**Y**	**L**	**Y**	**D**	**V**	**W**	**F**	**W**	**G**
ABS11196_*Paracoccidioides_brasiliensis*	**R**	**Y**	**F**	**Y**	**F**	**Y**	**E**	**L**	**W**	**Y**	**W**	**G**
BAW15172_*Pholiota_microspora*	**R**	**W**	**N**	**Y**	**F**	**Y**	**D**	**N**	**W**	**W**	**W**	**G**

*^a^* The potential SBSs in α-amylases from the subfamily GH13_5 have been analysed according to SBSs observed in the three-dimensional structures of the counterpart α-amylases from: (i) *Bacillus halmapalus* ([50]; PDB code: 2GJP)—SBS-I: W439 and W469; SBS-II: W347; and SBS-III: Y363; (ii) *Bacillus licheniformis* ([51]; PDB code: 6TOZ)—SBS-I: F257 and Y368; and (iii) *Halothermothrix orenii* ([52]; PDB code: 3BC9)—SBS-I: Y484 (Y460) and W512 (W488); SBS-II: W284 (W260) and W311 (W287); and SBS-III: W330 (W306) and W334 (W310)—the numbering in parentheses applies if the N-terminal signal peptide is removed; which all are highlighted by green-colour inversion. The individual α-amylases are presented by their GenBank accession Nos. and binomial name of the producing organism. The aromatic residues corresponding with the those forming the real SBSs are highlighted by black inversion.

**Table 4:** Residues corresponding with the SBSs from *H. sapiens* α-amylase *^a^*.

α-Amylase	I	I	II	II	III	IV
ABL09312_*Acarus_siro*	**T**	**W**	**F**	**W**	**W**	**N**
BAB85635_*Anguilla_japonica*	**Y**	**W**	**W**	**W**	**W**	**W**
AAL37207_*Crassostrea_gigas*	**W**	**W**	**Y**	**W**	**W**	**W**
AAL37183_*Crassostrea_gigas*	**W**	**W**	**F**	**W**	**W**	**W**
AAD38942_*Dermatophagoides_pteronyssinus*	**W**	**W**	**F**	**W**	**W**	**N**
AAD38943_*Euroglyphus_maynei*	**W**	**W**	**F**	**W**	**W**	**N**
AAC60246_*Gallus_gallus*	**Y**	**W**	**W**	**W**	**W**	**W**
ABO26610_*Haliotis_discus_discus*	**Y**	**W**	**F**	**W**	**W**	**T**
BAM74656_*Haliotis_discus_hannai*	**Y**	**W**	**F**	**W**	**W**	**T**
AAA51724_*Homo_sapiens* (pancreas)	**Y**	**W**	**W**	**W**	**W**	**W**
AAH63129_*Homo_sapiens* (saliva)	**Y**	**W**	**W**	**W**	**W**	**W**
AAA37221_*Mus_musculus*	**Y**	**W**	**W**	**W**	**W**	**F**
AAA37230_*Mus_musculus*	**Y**	**W**	**W**	**W**	**W**	**W**
H2N0D4_*Oryzias_latipes*	**Y**	**W**	**W**	**W**	**W**	**L**
CAA68065_*Pecten_maximus*	**N**	**W**	**F**	**W**	**W**	**W**
CAA54524_*Penaeus_vannamei*	**Y**	**W**	**R**	**W**	**W**	**F**
CAB65552_*Penaeus_vannamei*	**Y**	**W**	**R**	**W**	**W**	**F**
AAF65827_*Pseudopleuronectes_americanus*	**Y**	**W**	**W**	**W**	**W**	**C**
AAA40725_*Rattus_norvegicus*	**Y**	**W**	**W**	**W**	**W**	**W**
AAH88228_*Rattus_norvegicus*	**Y**	**W**	**W**	**W**	**W**	**F**
P83053_*Struthio_camelus*	**Y**	**W**	**W**	**W**	**W**	**W**
AAF02828_*Sus_scrofa*	**Y**	**W**	**W**	**W**	**W**	**W**
CAC87125_*Tetraodon_nigroviridis*	**Y**	**W**	**W**	**W**	**W**	**L**

*^a^* The potential SBSs in α-amylases from the subfamily GH13_24 have been analysed according to SBSs observed in the three-dimensional structures of the counterpart human salivary α-amylase ([54]; PDB code: 3BLP)—SBS-I: Y291 (Y276) and W299 (W284); SBS-II: W331 (W316) and W403 (W388); SBS-III: W218 (W203); and SBS-IV: W149 (W134)—the numbering in parentheses applies if the N-terminal signal peptide is removed); which is highlighted by the walnut-colour inversion. The individual α-amylases are presented by their GenBank accession Nos. and the binomial name of the producing organism. The aromatic residues corresponding with the those forming the real SBSs are highlighted by black inversion.

## Data Availability

Not applicable.

## Supplementary Materials

Figure S1: Sequence alignment of all 268 collected sequences representing α-amylases from subfamilies GH13_1, GH13_5, GH13_15, GH13_24, GH13_32 and GH13_42. Figure S2: Evolutionary trees of studied α-amylases based on the alignment of (a) sequences spanning the segment between the strands β1 and β8 of catalytic TIM-barrel; and (b) extracted eight conserved sequence regions. Table S1: List of 268 sequences from GH13 subfamilies containing fungal and chloride-dependent α-amylases.

## Abbreviations

CAZy: Carbohydrate-Active enZymes; CBM: carbohydrate-binding module; CSR: conserved sequence region; GH: glycoside hydrolase; PDB: Protein Data Bank; SBD: starch-binding domain; SBS: surface-binding site.
