# Supplementary material for: In Silico Analysis of Fungal and Chloride-Dependent α-Amylases within the Family GH13 with Identification of Possible Secondary Surface-Binding Sites
Source: Molecules. 2021 Sep 21;26(18):5704. doi: 10.3390/molecules26185704 (PMC8467227; doi:10.3390/molecules26185704)
Supplement: Supplementary file 1 [file molecules-26-05704-s001.zip › molecules-1345785-supplementary.pdf]

# Supplementary Materials

# In Silico Analysis of Fungal and Chloride-Dependent $\alpha$-Amylases within the Family GH13 with Identification of Possible Secondary Surface-Binding Sites

**Zuzana Janíčková [1,2] and Štefan Janeček [1,2,*]**

1 Department of Biology, Faculty of Natural Sciences, University of SS. Cyril and Methodius, SK-91701 Trnava, Slovakia

2 Laboratory of Protein Evolution, Institute of Molecular Biology, Slovak Academy of Sciences, SK-84551 Bratislava, Slovakia

```
                                            CSR-VIII                                          CSR-VI
1_AAA85446_Paenibacillus_polymyxa           MTDRFSNGDPSNDNYGGFNS---NNSDQRKWHGGDFQGIINKLDYIKNM-GFTAIWITPVTMQK----S----------------------------EYAYHGYHTYDFYA-VD-GHLGT
1_CAA49465_Thermoactinomyces_vulgaris       MTDRFDNGDPSNDNYGGFNA---DKSDPRKYHGGDFQGIINRLDYIKNM-GFTAIWITPVTMQK----S----------------------------ANAYHGYWTYDFYS-VD-GHLGS
1_BAD06003_Aspergillus_awamori              LTDRFGRTDNSTTATC---D---TG--DQIYCGGSWQGIINHLDYIQGM-GFTAIWISPITEQL----PQDT-----------------------SDGEAYHGYWQQKIYD-VN-SNFGT
1_BAD06002_Aspergillus_awamori              LTDRFARTDGSTTATC---N---TA--DQKYCGGTWQGIIDKLDYIQGM-GFTAIWITPVTAQL----PQTT-----------------------AYGDAYHGYWQQDIYS-LN-ENYGT
1_AAF14264_Aspergillus_flavus               LTDRFARTDGSTTATC---N---TA--DRKYCGGTWQGIIDKLDYIQGM-GFTAIWITPVTAQL----PQTT-----------------------AYGDAYHGYWQQDIYS-LN-ENYGT
1_BAD01051_Aspergillus_kawachii             LTDRFARTDGSTTATC---N---TA--DQKYCGGTWQGIIDKLDYIQGM-GFTAIWITPVTAQL----PQTT-----------------------AYGDAYHGYWQQDIYS-LN-ENYGT
1_BAA22993_Aspergillus_kawachii             LTDRFGRTDNSTTATC---N---TG--DQIYCGGSWQGIINHLDYIQGM-GFTAIWISPITEQL----PQDT-----------------------SDGEAYHGYWQQKIYY-VN-SNFGT
1_EAA64850_Aspergillus_nidulans             LTDRFARTDNSTTAEC---D---TS---AKYCGGTWQGIINQLDYIQGM-GFTAIWITPVTANL----E-DG-----------------------QHGEAYHGYWQQDIYA-LN-PHFGT
1_AAF17100_Aspergillus_nidulans             LTDRFARTDGSTTAAC---D---LA--QRRYCGGSWQGIINQLDYIQDM-GFTAIWITPITEQI----PDVT-----------------------AVGTGFHGYWQKNIYG-VD-TNLGT
1_P56271_Aspergillus_niger                  LTDRFGRTDNSTTATC---N---TG--NEIYCGGSWQGIIDHLDYIEGM-GFTAIWISPITEQL----PQDT-----------------------ADGEAYHGYWQQKIYD-VN-SNFGT
1_CAK44871_Aspergillus_niger                LTDRFARTDGSTTATC---N---TA--DQKYCGGTWQGIIDKLDYIQGM-GFTAIWITPVTAQL----PQTT-----------------------AYGDAYHGYWQQDIYS-LN-ENYGT
1_CAK40249_Aspergillus_niger                MTDRFARTDGSTTSPC---N---TT--EGLYCGGTWRGMINHLDYIQGM-GFDAVMISPIIENV----EGRV-----------------------EYGEAYHGYWPVDLYS-LN-SHFGT
1_CAK41088_Aspergillus_niger                MTDRFALTNGSTTAPC---N---TT--VANYCGGSWQGTIDKLDYIQGM-GFDAIMISPVIKNI----AGRS-----------------------KDGEAYHGYWPLDLYE-IN-SHFGT
1_CAA31218_Aspergillus_oryzae               LTDRFARTDGSTTATC---N---TA--DQKYCGGTWQGIIDKLDYIQGM-GFTAIWITPVTAQL----PQTT-----------------------AYGDAYHGYWQQDIYS-LN-ENYGT
1_BAA01255_Aspergillus_shirousami           LTDRFARTDGSTTATC---N---TA--DQKYCGGTWQGIIDKLDYIQGM-GFTAIWITPVTAQL----PQTT-----------------------AYGDAYHGYWQQDIYS-LN-ENYGT
1_AEB80431_Aspergillus_tubingensis          LTDRFGRTDNSTTATC---N---TG--DQIYCGGSWQGIINHLDYIQGM-GFTAIWISPITEQL----PQDT-----------------------SDGEAYHGYWQQKIYY-VN-SNFGT
1_BAA12010_Cryptococcus_sp_S_2              VTDRFALDNGGNSPSC---S---GQSELNLYCNGTFAGIIDKLDYIQNM-GFTAIWISPVVKNI----DGGS------------------PNGYTPDGSAYHGYWAQDIYE-IN-PHFGG
1_ABG48762_Fusicoccum_sp_BCC4124            LTDRFARTDGSTTATC---N---TQ--DRVYCGGTYQGLINRLDYIQNM-GFSAIWISPIVYNL----PETT-----------------------GYGQAYHGYWQQDLYR-LN-ENFGS
1_AAO12212_Lipomyces_kononenkoae            ITDRFATTNLT-APDC---W-------IRAYCGGTWKGLERKLDYIQNM-GFDAVWISPVIHNI----EVNT-----------------------TWGFAFHGYWGDDPYR-LN-EHFGT
1_AAC49622_Lipomyces_spencermartinsiae      VTDRFARTDGSITYSC---D---VT--DRVYCGGSYRGIINMLDYIQGM-GFTAIWISPIVENI----PDDT-----------------------GYGYAYHGYWMKDIFA-LN-TNFGG
1_AAN75021_Lipomyces_starkeyi               VTDRFARTDGSTTYLC---D---VT--DRVYCGGSYQGIINMLDYIQGM-GFTAIWISPIVENI----PDDT-----------------------GYGYAYHGYWMKDIFA-LN-TNFGT
1_AFD54462_Malbranchea_cinnamomea           LTDRFARGDGSPDAPC---D---TG--ARKYCGGNYRGLISQLDYIQGM-GFDSVWISPITKQF----EDDW------------------------NGAPYHGYWQTDLYA-LN-EHFGT
1_ABF72529_Ophiostoma_floccosum             LTDRFGRTDNSTSATC---N---TG--DRAYCGGSWQGVINHLDYIQGM-GFTAIWITPVTGQF----YEST-----------------------GDGTSYHGYWQQDIYS-LN-SHLGD
1_EPS26265_Penicillium_oxalicum             MTDRFGRTDNSVTASC---N---TN--DRVYCGGTWQGIINQLDYIQGM-GFTAIWITPVTEQL----PQDT-----------------------GDGEAYHGYWQQEIYN-VN-NNYGT
1_ABO42285_Phanerochaete_chrysosporium      IVDRYALPDGADPTKC---N---PA--DQTWCGGTWNTVRENLDYIQNA-GFTAIWISPVSQNY----QGPR------------------T----AYGDPYHGYWIADASK-LN-DRFGT
1_BAW15173_Pholiota_microspora              ITDRFAAPNGTDPAAC---Q---AS--ERTWCGGSWNTIRENLDYVQNA-GFTAIWISPVNQNY----HGPT------------------S----AYGDAYHGYWIADISQ-LN-DRFGT
1_BAF98616_Pichia_burtonii                  VTDRFARTDGSTSASC---D---VS--KREYCGGSFQGIIDKLDYIQGM-GFTAIWISPVVEQI----PDKT-----------------------EYGYGYHGYWMKNIYT-IN-DNFGT
1_AGJ52081_Rhizomucor_pusillus              LTDRFGRADDS--TSN---C---SN--LSNYCGGTYEGITKHLDYISGM-GFDAIWISPIPKNS----DG---------------------------------GYHGYWATDFYQ-LN-SNFGD
1_ADL28123_Rhizopus_oryzae                  LTDRFAKSTDD--TNG---C---SN--LSDYCGGTFQGIINHLDYIAGM-GFDAIWISPIPKNV----NG---------------------------------GYHGYWASDFSQ-IN-EHFGT
1_ADD80242_Saccharomycopsis_fibuligera      VTDRFARTDGDTSASC---N---TE--DRLYCGGSFQGIINKLDYIKDM-GFTAIWISPVVENI----PDNT-----------------------AYGYAYHGYWMKNIYK-IN-ENFGT
1_ABS76467_Saitozyma_flava                  VTDRFALDNGGNSPSC---S---GQSELNLYCNGTFAGIIDKLDYIQNM-GFTAIWISPVVKNI----DGGS------------------PNGYTPDGSAYHGYWAQDIYE-IN-PHFGG
1_CAB11471_Schizosaccharomyces_pombe        LTDRFASTN---PKPC---N---PE--DREYCGGNWRGIIDKLDYIQGM-GFTAIWISPIIKNI----EGRT-----------------------KYGEAYHGYWPQDLYT-LN-PHFGT
1_CAB40006_Schizosaccharomyces_pombe        LTDRFAVDDGSTDNPC---D---PD--ANQYCGGTWKGIENKLDYIEDM-GFNAIWISPIDKNI----EGDI------------------D----GAGYAYHGYWNTDYES-LN-EHFGT
1_CAA34162_Schwanniomyces_occidentalis      VTDRFARSDGSTTADC---L---VS--DRKYCGGSYKGIIDKLDYIQGM-GFTAIWISPVVEQI----PDNT-----------------------AYGYAYHGYWMKNIDE-LN-TNFGT
1_CAA51912_Schwanniomyces_occidentalis      VTDRFARTDGSTSATC---N---TG--DRVYCGGTFQGIIDKLDYIQGM-GFTAIWISPVVEQI----PDDT-----------------------GYGYAYHGYWMKDIYA-IN-SNFGT
1_ACN82436_Sclerotinia_sclerotiorum         ITDRFARTDGSTTASC---N---VN----EYCGGSWQGIIKHLDYIQNM-GFTAIWISPIVQNI----VGDS-----------------------VDGSSYHGYWAQNIYQ-VN-SNFGT
1_CAA03110_Thermomyces_lanuginosus          LTDRFARTDNSTTAPC---D---TT--AGKYCGGTWRGIINNLDYIQDM-GFTAIWITPVTAQW----DDDV------------------D---AADATSYHGYWQKDLYS-LN-SKFGT
1_BAG69580_Trichoderma_viride               LTDRIARSSSDTGGSA---C---TN--LNDYCGGTFQGLESKLDYIKGM-GFDAIWINPVVTNS----DF---------------------------------GFHGYWALDLNT-IN-SHYGT
1_CAJ21046_Valsaria_rubricosa               LTDRVANPST---TTA---C---SD--LSNYCGGTWSGLSSKLDYIQGM-GFDSIWITPVVENC----DG---------------------------------GYHGYWAKALYN-VN-TNYGS
5_AWX66236_Alicyclobacillus_sp_18711        FEWYLPNDG------------------------TLWTKMGSDASHLKSI-GITGVWFPPAYKGQ----S-QS------------------DVGYGVYDMYDLGEFN-QKGT-VR-TKYGT
5_AAQ01675_Alkalimonas_amylolytica          FYWDAYP--------------------------GLWANLPAMAAPLAER-GITSMWLPPAAKGM----NGTF------------------SVGYDVYDFWDLGEFY-QKGT-TA-TRYGS
5_CAL14744_Anoxybacillus_flavithermus       FEWYLPDDG------------------------TLWTKVANNAQSLANL-GITALWLPPAYKGT----S-SS------------------DVGYGVYDLYDLGEFN-QKGT-VR-TKYGT
5_AEW07376_Bacillus_acidicola               FEWYLPDDG------------------------TLWTKVANEANNLSSL-GITALWLPPAYKGT----S-RS------------------DVGYGVYDLYDLGEFN-QKGT-VR-TKYGT
5_AAA22191_Bacillus_amyloliquefaciens       FEWYTPNDG------------------------QHWKRLQNDAEHLSDI-GITAVWIPPAYKGL----S-QS------------------DNGYGPYDLYDLGEFQ-QKGT-VR-TKYGT
5_ABY86223_Bacillus_cereus                  FEWYAPNDG------------------------NHWNRLRTDAENLAEK-GITSVWIPPAYKGT----T-QN------------------DVGYGAYDLYDLGEFN-QKGT-VR-TKYGT
5_CAD26699_Bacillus_halmapalus              FEWHLPNDG------------------------NHWNRLRDDASNLRNR-GITAIWIPPAWKGT----S-QN------------------DVGYGAYDLYDLGEFN-QKGT-VR-TKYGT
5_AAA22226_Bacillus_licheniformis           FEWYMPNDG------------------------QHWKRLQNDSAYLAEH-GITAVWIPPAYKGT----S-QA------------------DVGYGAYDLYDLGEFH-QKGT-VR-TKYGT
5_AEM05860_Bacillus_licheniformis           FEWYMPNDG------------------------QHWKRLQNDSAYLAEH-GITAVWIPPAYKGT----S-QD------------------DVGYGAYDLYDLGEFH-QKGT-VR-TKYGT
5_AAK00598_Bacillus_megaterium              FEWYAPNDG------------------------NHWNRLRTDAENLAQK-GITSVWIPPAYKGT----T-QN------------------DVGYGAYDLYDLGEFN-QKGT-VR-TKYGT
5_AGN35141_Bacillus_paralicheniformis       FEWYMPNDG------------------------QHWKRLQNDSAYLAEH-GITAVWIPPAYKGT----S-QD------------------DVGYGAYDLYDLGEFH-QKGT-VR-TKYGT
5_AAR68734_Bacillus_sp                      FEWHLPNDG------------------------NHWNRLRDDAANLKSK-GITAVWIPPAWKGT----S-QN------------------DVGYGAYDLYDLGEFN-QKGT-VR-TKYGT
5_AAA22231_Bacillus_sp_707                  FEWYLPNDG------------------------NHWNRLNSDASNLKSK-GITAVWIPPAWKGA----S-QN------------------DVGYGAYDLYDLGEFN-QKGT-VR-TKYGT
5_BAF03567_Bacillus_sp_JAMB_204             FEWYLPNDG------------------------QHWNKMNNDAAYLSSI-GITALWIPPAYKGT----S-QA------------------DVGYGAYDLYDLGEFN-QKGT-VR-TKYGT
5_CAC39917_Bacillus_sp_KSM_K38              YEWHLENDG------------------------QHWNRLHDDAAALSDA-GITAIWIPPAYKGN----S-QA------------------DVGYGAYDLYDLGEFN-QKGT-VR-TKYGT
5_AAB18785_Bacillus_sp_MK_716               FEWYLPDDG------------------------TLWTKVANEANNLSSL-GITALWLPPAYKGT----S-RS------------------DVGYGVYDLYDLGEFN-QKGA-VR-TKYGT
5_AAA63900_Bacillus_sp_TS_23                FEWDLPNDG------------------------TLWTKVKNEAANLSSL-GITALWLPPAYKGT----S-QS------------------DVGYGVYDLYDLGEFN-QKGT-IR-TKYGT
5_ABW87262_Bacillus_sp_YX_YX1               FEWYTPNDG------------------------QHWKRLQNDAEHLSDI-GITAVWIPPAYKGT----S-QS------------------DNGYGPYDLYDLGEFQ-QKGT-VR-TKYGT
5_AAF00567_Cytophaga_sp                     FEWYVPNDG------------------------QQWNRLRTDAPYLSSV-GITAVWTPPAYKGT----S-QA------------------DVGYGPYDLYDLGEFN-QKGT-VR-TKYGT
5_AAC74994_Escherichia_coli                 FHWYYPEGG------------------------KLWPELAERADGFNDI-GINMVWLPPAYKGA----SGGY------------------SVGYDSYDLFDLGEFD-QKGS-IP-TKYGD
5_AFZ41193_Exiguobacterium_sp_DAU5          FEWYVPNDG------------------------LHWNHLSNDSQHLKDI-GISTVWIPPAYKGT----S-QN------------------DVGYGAYDLYDLGEFN-QKGT-TR-TKYGT
5_AAA22235_Geobacillus_stearothermophilus   FEWYLPDDG------------------------TLWTKVANEANNLSSL-GITALWLPPAYKGT----S-RS------------------DVGYGVYDLYDLGEFN-QKGA-VR-TKYGT
5_ABX83871_Geobacillus_thermodenitrificans  FEWYLSCDP------------------------QSFSEPPQCEVKVANL-GITALWLPPAYKGT----S-RS------------------DVGYGVYDLYDLGEFN-QKGA-VR-TKYGT
5_AFC87833_Geobacillus_thermoleovorans      FEWYLPDDG------------------------TLWTKVANEANNLSSL-GITALWLPPAYKGT----S-RS------------------DVGYGVYDLYDLGEFN-QKGT-VR-TKYGT
5_ACL70573_Halothermothrix_orenii           FYWEMNTGEYAT---------------EHPEEANLWNLLAERAPELAEA-GFTAVWLPPANKGM----AGIH------------------DVGYGTYDLWDLGEFD-QKGT-VR-TKYGT
5_CAQ30277_Nostoc_sp_PCC_7119               FHWYIPNDG------------------------NLWSKVEASAPELADA-GFTAMWLPPAYKGF----AGSF------------------DVGYGVYDLFDLGEFD-QKGS-VR-TKYGT
5_AAA27110_Salmonella_typhimurium           FHWYYPDGG------------------------KLWSELAERADGLNDI-GINMVWLPPACKGA----SGGY------------------SVGYDTYDLFDLGEFD-QKGT-IA-TKYGD
5_BAA24178_Streptococcus_equinus            FEWYLPNDG------------------------KHWMRLAADAPTLAQK-GITKIWMPPAFKAT----H-DG------------------DVGYGVYDLFDLGEFD-QKGT-IR-TKYGT
5_AAA97431_Streptococcus_equinus            FEWYLPNDG------------------------KHWKRLAADASHLAQK-GITKIWMPPAFKAT----H-DS------------------DVGYGVYDLFDLGEFN-QKGT-VR-TKYGT
5_AAN59233_Streptococcus_mutans             FEWYLPNDG------------------------KHWQHLAEDASHLKNI-GISKVWMPPAFKGT----G-SN------------------DVGYGVYDLYDLGEFN-QNGT-VR-TKYGS
5_CCD30600_uncultured_bacterium             FYWEMGETG------------------HPKEEENLWLLLTQKAPELSEV-GFTGVWLPPANKAM----GGKK------------------DVGYATYDLWDLGEFN-QKDT-VR-TKYGT
5_ALP73597_Vibrio_alginolyticus             FHWYVPNDG------------------------ALWTQVENNASALSDN-GFTALWLPPAYKGA----GGSN------------------DVGYGVYDMYDLGEFD-QQGS-VR-TKYGT
5_ABK62854_Histoplasma_capsulatum           FEWYVPDDQ------------------------RHWLRLRAALPSLKEI-GVTSILLPPGCKAM----S-PS------------------GNGYELYDLYDLGEFY-QKGS-RA-TKWGT
5_ABS11196_Paracoccidioides_brasiliensis    FEWFAPDDK------------------------KHWRRLQAALPSLKAI-GVTSIWLPPGCKAM----H-PS------------------GNGYDIYDLYDLGEFN-QKGT-KA-TKWGT
5_BAW15172_Pholiota_microspora              FTWDCLHER-----------------------LSWWKHFEEEIPRLAEL-GVTQVWLPPPNKAA----E-PK------------------GRGYDAYDLWDLGEFD-QKGS-IK-TRWGT
15_AAB60935_Aedes_aegypti                   FEWKFSDIAE------------------------------ECEKVLGPN-GYGGVQVSPINEYLVS-------------------------PS----RAWWERYQP-ISFE-IK-SRSGN
15_AAB60934_Aedes_aegypti                   FEWRWNDIAD------------------------------ECERFLAPR-GYAGVQVSPPTENIIV-------------------------SG----RPWWERYQP-ASYH-LN-TRSGS
15_AAA03322_Aedes_atropalpus                FEWRWNDIAD------------------------------ECERFLAPK-GYAGVQVSPPTENIVV-------------------------SG----RPWWERYQP-ASYH-LN-TRSGS
15_AAA03323_Anopheles_merus                 FEWKWSDIAD------------------------------ECERFLGPK-GYGGVQLSPVNENIVIRLA---------------------DGS----RPWWERYQP-ISFK-LD-TRSGS
15_AAN77138_Anthonomus_grandis              FEWKWADIAS------------------------------ECENFLSKK-GFAGVQISPPSENAV--V-----------------------SG----RPWWEKYQP-VSYV-LT-TRSGD
15_AAN77139_Anthonomus_grandis              FDWTWSDIAD------------------------------ECENFLGPN-GFGGVQTSPPNENII-WVQ---------------------NND----RPWWEAYQP-VSYK-LE-NRHGN
15_AAO13755_Asterias_rubens                 FEWRWVDIAA------------------------------ECERWLGPH-GFAGVQVSPPNQHLELP------------------------PD----RPWFERYQP-IGYA-LH-SRSGS
15_AAY23288_Blattella_germanica             FEWKFTDIAT------------------------------ECENFLGPK-GFAGVQVSPVHENLVV--T---------------------TPN----RPWWERYQV-ISYN-IT-SRSGN
15_BAB72257_Callosobruchus_chinensis        FEWRWDDIAN------------------------------ECETFLGPK-GFAGVQISPPSENIV--V-----------------------NG----RPWWERYQP-LSYD-LT-TRSGD
15_AAA03350_Culex_tarsalis                  FEWRWNDIAD------------------------------ECERFLAPK-GYAGVQVSPPTENAIVW-S---------------------PVR----RPWWERYQP-MSYE-LT-TRSGN
15_AAF20998_Diabrotica_virgifera_virgifera  FEWHWDAIAN------------------------------ECENFLGPK-GFAGVQVSPPNENCV--V-----------------------NG----RPWWERYQP-VSYQ-LI-TRSGD
15_AAC35243_Drosophila_ananassae            FEWKWKDIAT------------------------------ECENFLAPK-GYAGVQVSPVNENAVK-------------------------ER----RPWWERYQP-ISYQ-LM-TRSGN
15_BAB91544_Drosophila_bocki                FEWKWDDIAA------------------------------ECENFLGPN-GFAGVQVSPVNENAVK-------------------------DS----RPWWERYQP-ISYK-LV-TRSGN
15_BAA04582_Drosophila_erecta               FEWKWDDIAA------------------------------ECENFLGPN-GFAGVQVSPVNENAVK-------------------------DS----RPWWERYQP-ISYK-LD-TRSGN
15_BAB91509_Drosophila_kikkawai             FEWKWDDIAA------------------------------ECENFLGPN-GFAGVQVSPVNENAVK-------------------------DS----RPWWERYQP-ISYK-LV-TRSGN
15_BAA95437_Drosophila_leontia              FEWKWDDIAA------------------------------ECENFLGPN-GFAGVQVSPVNENAVK-------------------------DS----RPWWERYQP-ISYK-LV-TRSGN
15_BAA95441_Drosophila_lini                 FEWKWDDIAA------------------------------ECENFLGPN-GFAGVQVSPVNENAVK-------------------------DS----RPWWERYQP-ISYK-LV-TRSGN
15_BAA04583_Drosophila_mauritiana           FEWKWDDIAA------------------------------ECENFLGPN-GFAGVQVSPVNENAVK-------------------------DS----RPWWERYQP-ISYK-LE-TRSGN
15_AAK93479_Drosophila_melanogaster_Amyrel  FEWKWSDIAQ------------------------------ECESFLGPR-GFAGVQVSPVNENILS-------------------------AG----RPWWERYQP-ISYK-LT-TRSGN
15_CAA28238_Drosophila_melanogaster         FEWKWDDIAA------------------------------ECENFLGPN-GYAGVQVSPVNENAVK-------------------------DS----RPWWERYQP-ISYK-LE-TRSGN
15_AAC46839_Drosophila_pseudoobscura        FEWKWDDIAA------------------------------ECENFLGPQ-GYAGLQVSPVNENAVK-------------------------SG----RPWWERYQP-ISYK-LT-TRSGN
15_BAA04585_Drosophila_sechellia            FEWKWDDIAA------------------------------ECENFLGPN-GFAGVQVSPVNENAVK-------------------------DS----RPWWERYQP-ISYK-LE-TRSGN
15_AAC46463_Drosophila_virilis              FEWKWDDIAA------------------------------ECENFLGPY-GYAGLQVSPVNENAVI-------------------------GN----RPWWERYQP-ISYK-LN-TRSGN
15_AAG60011_Drosophila_yakuba               FEWKWDDIAA------------------------------ECENFLGPN-GFAGVQVSPVNENAVK-------------------------DS----RPWWERYQP-ISYK-LE-TRSGN
15_CAA76926_Phaedon_cochleariae             FEWHWDDIAN------------------------------ECENFLGPK-GFAGVQISPPAENTV--I-----------------------GD----RPWWERYQP-ISYA-LN-TRSGD
15_P56634_Tenebrio_molitor                  FEWKWNDIAD------------------------------ECERFLQPQ-GFGGVQISPPNEYLV--A-----------------------DG----RPWWERYQP-VSYI-IN-TRSGD
15_AAA03708_Tribolium_castaneum             FEWKWSDIAD------------------------------ECERFLAPK-GFGGVQISPPNENLV--VT---------------------SSN----RPWWERYQP-VSYI-LN-TRSGD
15_AAF73435_Zabrotes_subfasciatus           FEWNWGNLAK------------------------------ECETFLGPK-GFAGIQISPPNENVV--VG---------------------DEG----RPWWERYQP-LSYQ-LT-TRSGD
```

```
24_ABL09312_Acarus_siro                   MQWKFDDIAA------------------------------ECERFLGPK-GYGGIQLSPVNEHAVLG-------------------------N----RPWYELYQP-VGYK-IQ-SRSGN
24_BAB85635_Anguilla_japonica             FEWRWADIAD------------------------------ECERYLAPN-GFAGVQISPPSESIVLQ-----------------------NPW----RPWYERYQP-ISYN-LC-SRSGT
24_AAL37207_Crassostrea_gigas             FEWKWSDIAA------------------------------ECERFLGPK-GYCGVQISPPSENRIVT-----------------------NPN----RPWEERYQP-VSYK-LV-TRSGN
24_AAL37183_Crassostrea_gigas             FEWKWSDIAA------------------------------ECERFLGPM-GYCGVQISPPNENRIVT-----------------------SPN----RPWWERYQP-VSYK-LV-TRSGN
24_AAD38942_Dermatophagoides_pteronyssinus MEWKYDDIGD------------------------------ECERFLGPY-GYGGVQVSPVNEHAILD-------------------------R----RPWYERYQP-VSYD-IR-TRSGD
24_AAD38943_Euroglyphus_maynei            MEWKYDDIGD------------------------------ECERFLGPY-GYGGVQVSPVNEHAILD-------------------------R----RPWYERYQP-VSYD-LR-TRSGD
24_AAC60246_Gallus_gallus                 FEWRWADIAL------------------------------ECEHYLAPN-GFGGVQVSPPNENIVIT-----------------------NPN----RPWWERYQP-ISYK-IC-SRSGN
24_ABO26610_Haliotis_discus_discus        FEWTWSDIAK------------------------------ECERFLGPY-GYCGVQISPPNENRVVT-----------------------NPR----RPWWERYQP-VSYK-LQ-TRSGS
24_BAM74656_Haliotis_discus_hannai        FEWTWSDIAK------------------------------ECERFLGPY-GYCGVQISPPNENRVVT-----------------------NPR----RPWWERYQP-VSYK-LQ-TRSGS
24_AAA51724_Homo_sapiens_pancreatic       FEWRWVDIAL------------------------------ECERYLAPK-GFGGVQVSPPNENVAIY-----------------------NPF----RPWWERYQP-VSYK-LC-TRSGN
24_AAH63129_Homo_sapiens_salivary         FEWRWVDIAL------------------------------ECERYLAPK-GFGGVQVSPPNENVAIH-----------------------NPF----RPWWERYQP-VSYK-LC-TRSGN
24_AAA37221_Mus_musculus                  FEWRWVDIAK------------------------------ECERYLAPN-GFAGVQVSPPNENIVVH-----------------------SPS----RPWWERYQP-ISYK-IC-SRSGN
24_AAA37230_Mus_musculus                  FEWRWVDIAK------------------------------ECERYLAPK-GFGGVQVSPPNENVVVH-----------------------NPS----RPWWERYQP-ISYK-IC-TRSGN
24_H2N0D4_Oryzias_latipes                 FEWRWADIAA------------------------------ECERFLGPK-GFAGVQISPPNEHILVS-----------------------SPW----RPWWQRYQP-ISYN-LC-SRSGG
24_CAA68065_Pecten_maximus                FEWKWTDIAK------------------------------ECERFLGPN-GFCGVQISPPNENRLVN-------------------------N----RPWWERYQP-VSYK-LQ-TRSGS
24_CAA54524_Penaeus_vannamei              FEWKWSDIAA------------------------------ECENFLGPR-GFAGVQVSPPNEYVEVYQG---------------------DVK----RPWWERYQP-VSYK-LV-TRSGD
24_CAB65552_Penaeus_vannamei              FEWKWPDIAA------------------------------ECENFLAPR-GFAGVQVSPPTECLDAYQG---------------------DVQ----RPWWERYQP-VSYK-IA-SRSGD
24_AAF65827_Pseudopleuronectes_americanus FEWRWADIAA------------------------------ECERYLGPN-GFGGVQISPPNEHIMLD-----------------------NPW----RPWWMRYQP-ISYN-LC-SRSGS
24_AAA40725_Rattus_norvegicus             FEWRWADIAK------------------------------ECERYLAPK-GFGGVQVSPPNENIIIN-----------------------NPS----RPWWERYQP-ISYK-IC-SRSGN
24_AAH88228_Rattus_norvegicus             FEWRWVDIAK------------------------------ECERYLAPN-GFGGVQVSPPNENIVVN-----------------------SPF----RPWWERYQP-ISYK-IC-SRSGN
24_P83053_Struthio_camelus                FEWRWADIAL------------------------------ECERYLAPY-GFGGVQVSPPNENVIIT-----------------------NPY----RPWWERYQP-VSYK-LC-TRSGN
24_AAF02828_Sus_scrofa                    FEWRWVDIAL------------------------------ECERYLGPK-GFGGVQVSPPNENIVVT-----------------------NPS----RPWWERYQP-VSYK-LC-TRSGN
24_CAC87125_Tetraodon_nigroviridis        FEWRWTDIAA------------------------------ECERFLGPN-GFAGVQISPPSEHIILD-----------------------SPW----RPWWQRYQP-ISYN-LC-SRSGS
32_AAA21016_Aeromonas_hydrophila          FEWKFNDIAN------------------------------ECETVLGPK-GFGGVQVSPPAEHKQ--------------------------GS----QVWWTVYQP-VSYKNFN-SFGGC
32_BAA22082_Bacillus_sp_195               FSWTWNAIAR------------------------------ECTENLGPA-GYGYVQTSPPQEHI----QGAA---------------------------WWTHYQP-VSYK-IE-SRFGT
32_AAY89038_Bifidobacterium_breve         FQTNWNSVAK------------------------------ECTEAYGPE-GVGYVQVSPPMESI----QGTE---------------------------WWTSYQP-VSYK-LD-SKLGT
32_CAB92963_Halomonas_meridiana           FEWQWEDVAQ------------------------------ECENWLGPK-GFKAVQVSPPQEHIQ--------------------------GD-----AWWTRYQP-VSYQ-LE-SRSGS
32_BAJ52728_Kocuria_varians               FQWTWDSVAR------------------------------ACEQTLGPA-GYGAVQTSPPQEHI----TGDQ---------------------------WWVHYQP-VSYQ-LE-SRLGT
32_AKG25402_Microbacterium_aurum          FSYTWNKIAS------------------------------ECEDTLGPA-GYGYVQTSPPQEHAMVAGEGNP---------------------------WWIYYQP-VSYK-LE-SRMGT
32_CAA41481_Pseudoalteromonas_haloplanktis FEWNWQDVAQ------------------------------ECEQYLGPK-GYAAVQVSPPNEHIT--------------------------GS-----QWWTRYQP-VSYE-LQ-SRGGN
32_AAA86835_Pseudomonas_sp_KFCC10818      FEWSWQDIAQ------------------------------ECEEFLGPK-GFAAVQVSPPQKSVD--------------------------NP-----AWWSRYQP-VSYA-FE-GRSGN
32_AAA96317_Streptomyces_albus            FEWDYASVAK------------------------------ECTSTLGPA-GYGYVQVSPPAEHI----QGSQ---------------------------WWTSYQP-VSYK-IA-GRLGD
```

```
CSR-VIII (alignment position 1)   CSR-VI (alignment position 51)
32_BAC73693_Streptomyces_avermitilis  FEWKFDSVAR------------------------------ECTNTLGPA-GYGYVQVSPPAEHI----QGSQ--------------------------WWTSYQP-VSYK-IA-GRLGD
32_CAA40798_Streptomyces_griseus  FEWKFASVAR------------------------------ACTDSLGPA-GYGYVQVSPPQEHI----QGSQ--------------------------WWTSYQP-VSYK-IA-GRLGD
32_AAA26698_Streptomyces_hygroscopicus  FERKYVDVAK------------------------------ACTDQLGPA-GYGYVEVSPASEHI----QGGQ--------------------------WWTSYQP-VSYK-IA-GRLGD
32_AAA88554_Streptomyces_limosus  FEWKFASVAR------------------------------ACTDSLGPA-GYGYVQVSPPQEHI----QGSQ--------------------------WWTSYQP-VSYK-IA-GRLGD
32_CAB06622_Streptomyces_lividans  FEWKFTSVGQ------------------------------ACTDTLGPA-GYGYVQVSPPQEHI----QGGQ--------------------------WWTSYQP-VSYR-IA-GRLGD
32_CAA73775_Streptomyces_sp_TO1  FEWKYDSVAK------------------------------ECTSHLGP--GLPYVQVSPPAEHI----QGSQ--------------------------WWTSYQP-VSYK-IA-GPVGD
32_AAA82875_Streptomyces_sp_WL6  FERPYADVAS------------------------------ACTDQLGPA-GYGYVQVSPATEHI----QGDQ--------------------------WWTSYQP-VSYR-IA-GRLGD
32_AAA26697_Streptomyces_thermoviolaceus  FEWDYVSVAK------------------------------ECTSTLGPA-GYGYVQVSPPAEHI----QGSQ--------------------------WWTSYQP-VSYK-IA-GRLGD
32_AAB36561_Streptomyces_venezuelae  FEWNFASVAR------------------------------ECTDRLGPA-GYGYVQVSPPQEHL----QGGQ--------------------------WWTSYQP-VSYK-IA-GRLGD
32_ABF13430_Thermobifida_fusca  FQWRWKSIAD------------------------------ECRTTLGPH-GFGAVQVSPPQEHVVLPAEDYP--------------------------WWQDYQP-VSYK-LDQTRRGS
32_CAA41881_Thermomonospora_curvata  FQWRWKSIAD------------------------------ECRTTLGPH-GFGAVQVSPPQEHVVLPAEDYP--------------------------WWQDYQP-VSYK-LDQTRRGS
32_BAV60949_Pholiota_microspora  FEWTWDSIAA------------------------------ECTNFIGPA-GYGFVQVSPPQEHI----A----------------------G----SQWWTDYQP-VSYI-LT-SKRGN
32_KJA26527_Hypholoma_sublateritium  FEWTWDSIAA------------------------------ECTNFIGPA-GYGFVQVSPPQEHI----A----------------------G----AQWWTDYQP-VSHI-LV-SKRGN
32_KDR78015_Galerina_marginata  FQWTWDSIAA------------------------------ECTNFIGPA-GYGFVQVSPAQEHI----A----------------------G----PQWWTDYQP-VSYT-LT-SKRGS
32_PPQ83160_Psilocybe_cyanescens  FEWTWNSVAT------------------------------ECTDFIGPA-GYGFVQVSPPQEHI----T----------------------G----SQWWTDYQP-VSYI-LT-SKRGN
32_KTB32361_Moniliophthora_roreri  FEWSWDSVAS------------------------------ECTNFIGPA-GYGFVQVSPPAEHV----Q----------------------G----SQWWTDYQP-VSYT-LT-SKRGN
32_KDQ30003_Pleurotus_ostreatus  FEWTWDSVAA------------------------------ECTSFIGPA-GYGFVQVSPPAEHI----Q----------------------G----SQWWTDYQP-VSYT-LN-SKRGN
32_RDX41646_Polyporus_brumalis  FEWTWDSVAA------------------------------ECTNFIGPA-GYGFVQVSPPQETI----S----------------------G----DQWWTDYQP-VSYI-LT-SKRGN
32_OJT13448_Trametes_pubescens  FEWTWDSVAS------------------------------ECTNFIGPA-AYGFVQVSPPQETI----Q----------------------G----DQWWTDYQP-VSYI-LT-SKRGS
32_PFH45132_Amanita_thiersii  FEWNWDSVAS------------------------------ECRNFIGPN-GYGFVQVSPPAEHV----Q----------------------G----PQWWTDYQP-VSYT-LT-SKRGS
32_KIM37787_Hebeloma_cylindrosporum  FGWNWDSIAA------------------------------ECTNFIGPA-GYGFVQVSPPQEHI----T----------------------G----SQWWTEYQP-VSYN-LT-SKRGN
32_PBK61492_Armillaria_solidipes  FEWTWDSVAS------------------------------ECTDFIGPA-GYGFVQGNPPSEHI----T----------------------G----TQWWTDYQP-VTYT-LI-SKRGD
32_ETW76368_Heterobasidion_irregulare  FEWTWDSIAA------------------------------ECTNFLGPA-GYGFVQASPAAEHI----S----------------------G----SQWWTDYQP-VSYI-LT-SKRGN
32_RDB27268_Hypsizygus_marmoreus  FDWTWDSIAV------------------------------ECTNFIGPA-GYGFVQVSPPQEHI----Q----------------------G----SQWWTDYQP-VSYT-LT-SKHGD
32_PPQ71147_Gymnopilus_dilepis  FEWNWDSIAA------------------------------ECTNFIGSA-GYGFVQVSPPQEHV----T----------------------G----PQWWTDYQP-VSYN-LT-SKRGD
32_OSD07916_Trametes_coccinea  FEWTWDSVAA------------------------------ECTNFIGPA-GYGFVQVSPPQEHI----Q----------------------G----DQWWTDYQP-VSYI-LT-SKRGD
32_KZW01389_Exidia_glandulosa  FEWSWDSVAA------------------------------ECTNFIGPA-GYGFVQVSPPAEHI----Q----------------------G----AQWWTDYQP-VSYT-LT-SKRGN
32_EIW55835_Trametes_versicolor  FEWTWDSVAS------------------------------ECTNFIGPA-GYGFVQVSPPQETI----Q----------------------G----DQWWTDYQP-VSYI-LT-SKRGS
32_SJL11930_Armillaria_ostoyae  FEWTWDSVAS------------------------------ECTDFIGPA-GYGFVQVSPPSEHV----T----------------------G----TQWWTDYQP-VTYT-LI-SKRGD
32_PAV18085_Pyrrhoderma_noxium  FEWSWDSIAA------------------------------ECTNFIGPA-GYGFIQASPAQETI----T----------------------G----DQWWTDYQP-VSYI-LT-GKRGD
32_RPD65356_Lentinus_tigrinus  FEWTWDSVAA------------------------------ECTNFIGPA-GYGFVQVSPPQETI----T----------------------G----DQWWTDYQP-VSYI-LT-SKRGN
32_PBK84861_Armillaria_gallica  FEWTWDSIAS------------------------------ECTDFIGPA-GYGFVQGNPPEEHI----T----------------------G----TQWWTDYQP-VTYT-LT-SKRGD
32_KLO18298_Schizopora_paradoxa  FEWNWDSIAA------------------------------ECTSFIGPA-GYGFVQASPAQEHI----T----------------------G----DQWWTDYQP-VSYI-LT-SKRGD
32_PPR08271_Panaeolus_cyanescens  FEWNWDSVAA------------------------------ECSAFIGPA-GYGFVQVSPPQEHI----T----------------------G----PQWWTDYQP-VSYN-IT-SKRGN
32_KIK53845_Gymnopus_luxurians  FEWTWDSIAA------------------------------ECTNFLGPA-GYGYVQTSPPQEHI----T----------------------G----TQWWTDYQP-VSYI-LT-SKRGD
32_KIJ56431_Sphaerobolus_stellatus  FEWTWDSVAA------------------------------ECTNFIGPA-GYGFVQVSPPQEHI----Q----------------------G----SQWWTDYQP-VSYI-LT-SKRGN
32_EJD07843_Fomitiporia_mediterranea  FEWTWDSIAA------------------------------ECASFIGPA-GYGFIQASPAQEHV----T----------------------G----DQWWTDYQP-VSYI-LT-SKRGN
32_EPQ53992_Gloeophyllum_trabeum  FEWNWDSLAA------------------------------ECTNFLGPA-GYGFVQASPAQEHI----T----------------------G----SQWWTDYQP-VSYT-LT-SKRGN
32_KIP11288_Phlebiopsis_gigantea  FEWTWDSIAA------------------------------ECTAFLGPN-GYGFVQASPAMEHV----Q----------------------G----TQWWTDYQP-VSYI-LT-SKRGD
32_KIY62009_Cylindrobasidium_torrendii  FEWTWDSIAA------------------------------ECTNFIGPA-GYGYVQVSPPAEHV----T----------------------G----SQWWTDYQP-VSYQ-LI-SKRGN
32_KIJ69360_Hydnomerulius_pinastri  FEWDWDSIAA------------------------------ECTNFIGPA-GYGYVQASPAQEHV----T----------------------G----DEWWTDYQP-VSYT-LT-SKRGH
32_KZT20455_Neolentinus_lepideus  FEWDWDSIAS------------------------------ECTSFLGPA-GYGFVQASPAQEHI----T----------------------G----TQWWTDYQP-VSYT-LT-SKRGN
32_EGN97351_Serpula_lacrymans_var_lacrymans  FEWSWDSIAL------------------------------ECTQFIGPA-GYGYVQASPAQETI----T----------------------G----TQWWTDYQP-VSYT-LT-SKRGN
32_KDQ06010_Botryobasidium_botryosum  FDWNWDSIAA------------------------------ECSAFIGPA-GYGYVQVSPPQEHI----Q----------------------G----NQWWTDYQP-VSYN-LN-SKRGN
32_PPR00074_Panaeolus_cyanescens  FGWNWDSIAA------------------------------ECTNFIGPA-GYGFVQSNPPQEHI----Q----------------------G----SQWWTDYQP-VSYQ-LN-SKRGN
32_EKM53319_Phanerochaete_carnosa  FEWTWDSVAA------------------------------ECYNFIGPA-GYGFVQVSPAQEHV----Q----------------------G----SQWWTDYQP-VSYT-LT-SKRGD
32_TBU45601_Dichomitus_squalens  FEWTWDSVAS------------------------------ECTNFIGPA-GYGFVQVSPPQEHV----T----------------------G----DQWWTDYQP-VSYI-LT-SKRGN
32_PSR82077_Phlebia_centrifuga  FEWTWDSVAA------------------------------ECTNFLGPA-GYGFVQVSPPQEHV----T----------------------G----TQWWTDYQP-VSYT-LT-SKRGD
32_KIM89391_Piloderma_croceum  FEWTWDSVAS------------------------------ECTNFIGPS-GYGYVQVSPAQEHV----T----------------------G----DQWWTDYQP-VSYI-LT-SKRGN
32_PIL31175_Ganoderma_sinense  FEWTWDSVAA------------------------------ECTDFIGPA-GYGFVQVSPAQEHV----T----------------------G----DQWWTDYQP-VSYI-LT-SKRGN
32_KIY70325_Cylindrobasidium_torrendii  FEWTWDSIAS------------------------------ECTDFIGPA-GYGYVQVSPPAEHV----T----------------------G----DAWWTDYQP-VSYQ-LI-SKRGT
32_KDQ58060_Jaapia_argillacea  FEWTWDSVAA------------------------------ECTNSLGPA-GYGFVQVSPAQEHI----T----------------------G----AQWWTDYQP-VSYI-LT-SKRGD
32_CEL62905_Rhizoctonia_solani  FEWSWDSIAA------------------------------ECTNFIGPA-GYGFVQVSPPAEHI----A----------------------G----SQWWTDYQP-VSYT-LT-SKRGN
32_GAT57350_Mycena_chlorophos  FEWTWASVAA------------------------------ECTNFIGPA-GYGFVQVSPPQEHI----T----------------------G----SAWWTDYQP-VSYI-LT-SKRGN
32_KIM25608_Serendipita_vermifera  FEWTWDSIAS------------------------------ECTNFIGPN-GYGYVQVSPPAEHI----Q----------------------G----SSWWTDYQP-VSYT-LT-SKRGN
32_GAW02849_Lentinula_edodes  FEWTWDSIAT------------------------------ECTDFLGPA-GYGYVQTSPPQEHI----T----------------------G----TEWWTDYQP-VSYI-LT-SKRGD
32_TCD63303_Steccherinum_ochraceum  FEWTWDSIAA------------------------------ECTEFLGPA-GYGYVQASPAQEHV----Q----------------------G----PEWWTDYQP-VSYT-LT-SKRGN
32_KZP22139_Fibularhizoctonia_sp_CBS_109695  FQWDWPSVAA------------------------------ECTAFLGPA-GYGYVQVSPPQESI----T----------------------G----TAWWIDYQP-VSYT-LT-SQHGN
32_KII90441_Plicaturopsis_crispa  FQWNWDSVAA------------------------------ECTNFIGPA-GYGFVQVSPPQEAI----T----------------------G----SSWWTDYQP-VSYT-LT-SKYGD
32_KDQ58058_Jaapia_argillacea  FEWSWDSIGT------------------------------ECTSSLGPA-GYGFVQASPAQEHV----TGPPSSL-LIPRSLNLKRFV---G----TQWWTDYQP-VSYI-LT-SKRGN
32_OCH93218_Obba_rivulosa  FEWTWESIAA------------------------------ECEAFLGPT-GYGFVQASPAQEHV----Q----------------------G----SQWWTDYQP-VSYI-LT-SKRGD
32_EMD36390_Gelatoporia_subvermispora  FEWTWESIAA------------------------------ECEAFLGPA-GYGFVQASPAQEHV----Q----------------------G----SEWWTDYQP-VSYI-LT-SKRGD
32_EIW78746_Coniophora_puteana  FEWNWDSIAS------------------------------ECTNFIGPT-GYGFVQVSPPQEAI----Q----------------------G----DQWWTDYQP-VSYN-IT-SKRGN
32_KII86244_Plicaturopsis_crispa  FEWSWDSVAA------------------------------ECTNFIGPA-GYGFVQVSPPAEHI----S----------------------G----DQWWTDYQP-VSYT-LT-SKRGN
32_EKM75205_Agaricus_bisporus  FEWNWDSVAA------------------------------ECRNFIGPH-GYGFVQVSPPQEHI----T----------------------G----SQWWTDYQP-VSYT-LT-SKRGN
32_KZT36083_Sistotremastrum_suecicum  FQWSWASIAA------------------------------ECTSFIGPA-GYGYVQVSPAQEHI----Q----------------------G----AQWWTDYQP-VSYI-IE-SKRGT
32_KZS90559_Sistotremastrum_niveocremeum  FQWSWASIAA------------------------------ECTSFIGPA-GYGYVQVSPAQEHI----Q----------------------G----AQWWTDYQP-VSYI-IE-SKRGT
32_EFI98134_Schizophyllum_commune  FEWNWDSIAQ------------------------------ECTDFIGPA-GYGFVQVSPPQETI----S----------------------G----DQWWTDYQP-VSYI-LQ-GKRGD
32_BBC62173_Tricholoma_matsutake  FEWAWDSVAL------------------------------ECTSFIGPA-GYGYVQVSPPQEHV----T----------------------G----AQWWTDYQA-VSYI-LT-SKRGD
32_CCA69081_Serendipita_indica  FQWSWNSVAA------------------------------ECTTFIGPA-GYGYVQVSPPAEHI----T----------------------G----AQWWTDYQP-VSYI-LT-SKRGS
32_EFI98135_Schizophyllum_commune  FEWNWESVAA------------------------------ECTNFLGPA-GYGYVQVSPPQEHI----Q----------------------G----PQWWTDYQP-VSYN-LV-SKRGD
32_KII85613_Plicaturopsis_crispa  FQWDWVSLAQ------------------------------ECTDFLGPA-GYGFVQTSPANEHI----T----------------------G----VPWYTDYQP-VTYN-IT-SKRGT
32_EIN11730_Punctularia_strigosozonata  FEWTWDSIAA------------------------------ECTAYLGPT-GYGYVQTSPPQEHI----T----------------------G----DQWWTDYQP-VSYT-LT-SKRGT
32_PIL33242_Ganoderma_sinense  FEWSWDSIAA------------------------------ECTTFIGPA-GYGYVQVNPPQEHI----Q----------------------G----DSWWTDYQA-VSYQ-LT-SKHGN
32_KZT18190_Neolentinus_lepideus  FEWTWDSIAD------------------------------ECVSFLGPA-GYAFVQTSPPQEHV----T----------------------G----DQWWTDYQP-VSYI-LT-SKRGN
32_KZV75308_Peniophora_sp_CONT  FQWSWDSIAS------------------------------ECTNFLGPA-GYGYVQTSPPQETI----T----------------------G----GQWWVDYQA-VSYK-LQ-NNHGN
32_KII86838_Plicaturopsis_crispa  FEWDWDSLAA------------------------------ECTNFLGPA-GYGFVQTSPPNEHI----T----------------------G----TQWWTDYQP-VSYN-IT-SKRGN
32_KDQ08018_Botryobasidium_botryosum  FEWNWVSVAN------------------------------ECKNFIGPA-GYGYVQVSPPQEHI----Q----------------------G----GQWWTDYQP-VSYT-LN-SKRGN
32_KIY73061_Cylindrobasidium_torrendii  FEWTWDSIAS------------------------------ECTDFIGPA-GYGFVQVSPPAEHI----S----------------------G----DQWWTDYQP-VSYN-IA-SKRGT
32_KZW01365_Exidia_glandulosa  FGWNWDSIAQ------------------------------ECTQFLGPA-GYGYVQISPPQETV----TGDQ--------------------------WWTDYQP-VSYN-LQ-GKRGD
32_KDQ58178_Jaapia_argillacea  FQWSWDSIAS------------------------------ECTNFLGPA-GYGYVQASPAQEHV----QGPQ--------------------------WWTDYQP-VSYQ-IS-SKRGS
32_KZV75307_Peniophora_sp_CONT  FQWSWDSIAS------------------------------ECTNFLGPA-GYGYVQTSPPQETI----T----------------------G----GQWWVDYQP-VSYT-IQ-NNHGN
32_KIK51212_Gymnopus_luxurians  FQWSWDSIAA------------------------------ECTGFLGPA-GYGYVQASPAQEHI----I----------------------G----DTWWTDYQP-VSYN-LT-SKRGD
32_RDX41931_Polyporus_brumalis  FEWNWDSVAA------------------------------ECQNFIGPA-GYGFVQVNPPQESI----S----------------------G----DQWWTDYQA-VSYQ-LT-SKHGN
32_TBU29254_Dichomitus_squalens  FQWNWDSIAR------------------------------ECTNFVGPA-GYGYVQVNPPQEHI----Q----------------------G----DSWWTDYQA-VSYQ-LT-SKHGN
32_KIY45802_Fistulina_hepatica  FEWDWNSVAA------------------------------ECTAFIGPA-GYEYVQVSPAQESI----TGTQ--------------------------WWTSYQA-VSYI-LT-SKRGD
32_KIL59382_Amanita_muscaria  FEWNWDSVAD------------------------------ECQKFLGPA-GYGFVQVSPPQEHV----S----------------------G----TQWWTSYQP-VSYK-IY-SKRGN
32_KIY53725_Fistulina_hepatica  FEWDWNSVAD------------------------------ECTNFIGPA-GYGYVQGITTMESI----QGDE--------------------------WWTSYQA-VSYT-LT-SKRGT
32_KIY47132_Fistulina_hepatica  FQWSWTSIAQ------------------------------ECTNFLGPA-GYGYVQVSPPMEHI----AGEQ--------------------------WWTDYQP-VSYN-LT-SKHGT
32_RPD55342_Lentinus_tigrinus  FEWNWDSVAA------------------------------ECQNFIGPA-GYGFVQVNPPQESI----S----------------------G----DQWWTDYQA-VSYQ-LT-SKHGN
32_TCD61445_Steccherinum_ochraceum  FQWNYASVAA------------------------------ECTEFIGPA-GYGYVQVSPPTESI----S----------------------G----SQWWTDYQV-VSYT-LT-SKHGS
32_KIM25602_Serendipita_vermifera  FEWNWASIAQ------------------------------ECTDFLGPA-GYGFVQTSPPQEHI----T----------------------G----TQWWTAYQP-VSYN-IA-SNKGT
32_EJD37603_Auricularia_subglabra  FGWNWDSIAQ------------------------------ECTQFLGPA-GYGAIQISPPQETV----TGDQ--------------------------WWTDYQP-VSYK-LE-GKRGT
32_KZT54766_Calocera_cornea  FEWNWDSVAQ------------------------------ECAEFIGPA-GYGFVQVSPPQEHI----EGPQ--------------------------WWTDYQP-VSYN-IT-SKRGN
32_KIY46805_Fistulina_hepatica  FEWNWDSVAE------------------------------ECKNFIGPA-GYAYVQVSPPQESI----QGGQ--------------------------WWTDYQP-VSYK-LQ-SKRGS
32_PPQ69654_Psilocybe_cyanescens  FEWTWDSVAA------------------------------ECTNFIGPA-GYGFVQVSPPQEHI----T----------------------G----SQWWTDYQP-VSYT-LT-SKRGN
32_KZO93971_Calocera_viscosa  FEWNWDNIAQ------------------------------ECVEFIGPA-GYGFVQASPPQEHM----E----------------------G----PQWWTDYQP-VSHN-IT-SKRGN
32_KZP07860_Fibularhizoctonia_sp_CBS_109695  FQWNWNSVAA------------------------------ECTTFIGPA-GYGFAQVSPAQESI----QGAQ--------------------------WSSSYSP-VSYI-ID-NKLGT
32_PVG03687_Serendipita_vermifera  FEWSWDSIAQ------------------------------ECTDFLGPA-GYGYVQTSPPMEHI----QGKLNLFLSLPRLLMLRQEVNGGQ----HIRFDASQP-VSYA-IV-SNRGN
32_EIM87674_Stereum_hirsutum  FEWPWTSIAT------------------------------ECTDYLGPF-GYGYVQVSPPMEHI----TGTQ--------------------------WWCDYQP-VSYT-LT-SRRGT
32_CCA69087_Serendipita_indica  FEWNWNSIKA------------------------------ECVAFLGPA-GYGYVQTSPPNAHI----VGKQ--------------------------WWTAYQP-TSYD-LV-SNRGT
32_CEL62035_Rhizoctonia_solani  FGWNWNSIGE------------------------------ECVQFLGPA-GVGFVQVNPPQEHL----AGDQ--------------------------WWVDYQV-VSYQ-LQ-SKRGS
32_EIN05398_Punctularia_strigosozonata  FQWNWDSIAQ------------------------------ECTQYLGPA-GYEWVQISPPSEHV----T----------------------G----DSWWTDYQV-VSYT-LT-SKRGN
32_EJU02896_Dacryopinax_primogenitus  FEWDWDSVAR------------------------------ECGAFIGPAEGYGFVQVSPPQEHM----VGPQ--------------------------WWTDYQP-VSHN-II-SKRGN
32_KIO30302_Tulasnella_calospora  FQWNWDSIAA------------------------------ECTQFIGPA-GYGYVQVSPPQEHI----T----------------------G----SQWWTDYQP-TSYQ-LV-SKRGN
32_CEL62036_Rhizoctonia_solani  FGWNWKSLAA------------------------------ECEQFLGPA-GVGYVQVNPPQEHL----SGDA--------------------------WWVDYQA-VSYQ-LQ-SKRGS
32_KIO24533_Tulasnella_calospora  FQWNWDSVAR------------------------------ECA-WLGKN-GYGFVQVSPASEHI----QGAQ--------------------------WWTDYQV-VSYK-LQ-SKRGN
32_EIM84100_Stereum_hirsutum  FEWNWNSVAQ------------------------------ECTDFIGPA-GYAYVQVSPATEHI----TGDS--------------------------WWTDYQK-VSNQ-LI-SKRGS
32_KDN35973_Rhizoctonia_solani  FGWSWDSIAV------------------------------ECTDFIGPA-GYGFVQVSPPSEHT----IGSS--------------------------WWTDYQP-VSYI-LT-SKRGD
32_KIY48489_Fistulina_hepatica  FQWSWDSVAA------------------------------ECTNFIGPA-GYGYVQVSPAQEAL----SGDE--------------------------WWTDYQS-ISYK-LI-NRHGD
32_KIY48486_Fistulina_hepatica  FQWSWESVGA------------------------------ECTNFIGPA-GYGYVQVSPAQEAY----SGDE--------------------------WWTDYQS-VSYT-IK-NKHGD
32_KZP26813_Fibularhizoctonia_sp_CBS_109695  FEWDWNSVAT------------------------------ECTQFIGPA-GYGYVQVSPPSEHI----TGSQ--------------------------WWTDYQV-VSYE-LT-SKRGT
32_KDQ32476_Pleurotus_ostreatus  FQWNWKSVAE------------------------------ECEHFIGPA-GYGYVQVSPPNEHI----TGNE--------------------------WWKDYQP-VSYK-IE-SRRGN
32_EIM84087_Stereum_hirsutum  FQWNWDSVAK------------------------------ECTEFIGPA-GYSYVQVSPATEHA----AGDE--------------------------WYFDYQT-VSYN-LD-SRRGN
32_KDQ32475_Pleurotus_ostreatus  FQWDWDSIGE------------------------------ECAEFIGPA-GYGFVQVNPPSEHV----QGSQ--------------------------WWTDYQP-VSYK-IA-LKHGN
32_EAQ88347_Chaetomium_globosum  FEWNYDSVAR------------------------------ECSSTLGPA-GYGYVQVSPPAEHI----QGSQ--------------------------WWTSYQP-VSYK-IA-GRLGN
32_ORY89754_Leucosporidium_creatinivorum  FEYSWDSVAT------------------------------ECTEWLGPQ-GFGYAQISPPQESI----QGKA--------------------------WYTDYQP-ISYN-LV-SKRGN
32_EWZ34052_Fusarium_oxysporum  FEWNFDSVAR------------------------------ECNNTIGPA-GYGYVQVSPPAEHI----QGPE--------------------------WWTSYQP-VSYR-IA-GRLGN
32_OBT91518_Pseudogymnoascus_verrucosus  FEWNFNSVAK------------------------------ECTNTIGPA-GYGYVQVSPPAEHI----QGAQ--------------------------WWTSYQP-VSYK-IA-GRLGD
32_KEY73157_Stachybotrys_chartarum  FEWSYASVAR------------------------------ECTNTLGPA-GYGFVQVSPPAEHI----QGSQ--------------------------WWTSYQP-VSYR-IA-GRLGD
32_OBT62577_Pseudogymnoascus_sp_23342_1_I1  FEWNFNSVAK------------------------------ECTNTIGPA-GYGYVQVSPPAEHI----QGAQ--------------------------WWTSYQP-VSYK-IA-GRLGD
32_EON97874_Phaeoacremonium_minimum  FEWNFDSVAR------------------------------ECTQTLGPA-GYGYVQVSPPAEHI----QGSQ--------------------------WWTSYQP-VSYQ-IA-GRLGN
32_GAT55198_Mycena_chlorophos  FEWPWDSVAT------------------------------ECTSFIGPF-GYGFVQVSPPQEHI----TGPE--------------------------WWTDYQP-VSYN-LT-SKRGT
32_OAG04271_Paraphaeosphaeria_sporulosa  FKSNYDSVAR------------------------------ECARTLGTA-GYGYVQVSPPAEHI----QGSQ--------------------------WWTSYQP-VSYK-LA-GRLGD
32_EGG02960_Melampsora_larici_populina  FQWPHIDVAN------------------------------ECESHLAPA-GYRWVQVSPPQEHI----RGPQ--------------------------WWVDYQP-VSYI-LN-SKRGS
32_EGG02993_Melampsora_larici_populina  FQWSHVDVAN------------------------------ECESHLGPA-GYRWAQVSPPQEHI----EGNA--------------------------WWVDYQP-VSYN-LY-SKRGT
32_OAV91411_Puccinia_triticina  FQWRYPDVGT------------------------------ECETFLGPN-GYRFVQVSPPQEHI----EGPQ--------------------------WWTDYQP-VSYR-IG-SKRGS
32_EFP79978_Puccinia_graminis  FQWRYSDVGD------------------------------ECEAFLGPN-GYRYVQVSPPHEHI----EGPQ--------------------------WWTDYQP-VSYR-IG-SKRGS
32_GAT51088_Mycena_chlorophos  FEWPWTSVAT------------------------------ECTQYLAPF-GYGYVQVSPASEHI----TGTQ--------------------------WWTDYQP-VSYV-LT-SKRGT
32_KNE94052_Puccinia_striiformis  FQWRYSDVGN------------------------------ECEAFLGPN-GYRYVQISPPHEHI----EGPQ--------------------------WWTDYQP-VSYR-IG-SKRGS
32_KNZ54553_Puccinia_sorghi  FQWRYSDVAL------------------------------ECQHFLGPN-GYRYVQISPPHEHI----QGPQ-----------------W-----WTDYQ----P-VSYR-IA-SKRGT
42_AOF40721_Microbacterium_aurum  MTARWNDGDSSNNMGGSQNVKSGNAANNDPMFRGDFKGVIDKLDYIKAL-GFSALWITPVVTNR----S---------------------------DYDFHGYHGWDFQR-VD-PRLET
42_CAB06816_Streptomyces_lividans  LTARFNDGDATNNRGGSQHEKSGNAANDDPMFRGDFKGLVNKLDYIKGL-GFSAVWITPVVLNR----S---------------------------DYDYHGYHGYDFYK-VD-PRLES
42_AWA81565_Pecoramyces_ruminatium  MTARFFDGDSSNNVHCIDDSKA-KNGDDDPCFRGDFKGLIDKLDYIKAL-GFTAIWITPVVENG----S---------------------------SYDYHGYHALNFDR-VD-PRLET
```

```
                                                                                    CSR-I
1_AAA85446_Paenibacillus_polymyxa           MD-KLQELVRKAHDKNIAVMV------------------DVVVNHTGD-------FQPG------------------------------------------------------------
1_CAA49465_Thermoactinomyces_vulgaris       MA-KLQELVRAAHAKGISVML------------------DVVANHTGD-------FQPS------------------------------------------------------------
1_BAD06003_Aspergillus_awamori              AD-DLKSLSDALHARGMYLMV------------------DVVPNHMGY-------AGNG------------------------------------------------------------
1_BAD06002_Aspergillus_awamori              AD-DLKALSSALHERGMYLMV------------------DVVANHMGY-------DGAG------------------------------------------------------------
1_AAF14264_Aspergillus_flavus               AD-DLKALSSALHERGMYLMV------------------DVVANHMGY-------DGAG------------------------------------------------------------
1_BAD01051_Aspergillus_kawachii             AD-DLKALSSALHERGMYLMV------------------DVVANHMGY-------DGAG------------------------------------------------------------
1_BAA22993_Aspergillus_kawachii             AD-DLKSLSDALHARGMYLMV------------------DVVPNHMGY-------AGNG------------------------------------------------------------
1_EAA64850_Aspergillus_nidulans             QD-DLRALSDALHDRGMYLMV------------------DVVANHFGY-------DAPA------------------------------------------------------------
1_AAF17100_Aspergillus_nidulans             AD-DIRALSEALHDRGMYLML------------------DVVANHMSY-------GGPG------------------------------------------------------------
1_P56271_Aspergillus_niger                  AD-NLKSLSDALHARGMYLMV------------------DVVPDHMGY-------AGNG------------------------------------------------------------
1_CAK44871_Aspergillus_niger                AD-DLKALSSALHERGMYLMV------------------DVVANHMGY-------DGAG------------------------------------------------------------
1_CAK40249_Aspergillus_niger                HQ-DLLDLSDALHARDMYLMM------------------DTVINNMAY---ITNGSDPA------------------------------------------------------------
1_CAK41088_Aspergillus_niger                RE-ELLKLSEEIHARGMYLLL------------------DVVINNMAY---MTDGEDPA------------------------------------------------------------
1_CAA31218_Aspergillus_oryzae               AD-DLKALSSALHERGMYLMV------------------DVVANHMGY-------DGAG------------------------------------------------------------
1_BAA01255_Aspergillus_shirousami           AD-DLKALSSALHERGMYLMV------------------DVVANHMGY-------DGAG------------------------------------------------------------
1_AEB80431_Aspergillus_tubingensis          AD-DLKSLSDALHARGMYLMV------------------DVVPNHMGY-------AGNG------------------------------------------------------------
1_BAA12010_Cryptococcus_sp_S_2              AS-GLTDLSNALHSRGMYLMV------------------DVVVNHMAYYCGTDGGCGPG------------------------------------------------------------
1_ABG48762_Fusicoccum_sp_BCC4124            AD-DLKALAAELHDRDMYLMV------------------DVVVNHMAW-------AGSA------------------------------------------------------------
1_AAO12212_Lipomyces_kononenkoae            AA-DLKSLSDSLHARGMSLMV------------------DVVINHLAS-------YTLP------------------------------------------------------------
1_AAC49622_Lipomyces_spencermartinsiae      AD-DLIALATELHNRGMYLMV------------------DIVVNHFAF-------SGNH------------------------------------------------------------
1_AAN75021_Lipomyces_starkeyi               AD-DLIALATELHNRGMYLMV------------------DIVVNHFAF-------SGSH------------------------------------------------------------
1_AFD54462_Malbranchea_cinnamomea           EE-DLRALADELHARGMFLMV------------------DVVINHNGW-------PGDA------------------------------------------------------------
1_ABF72529_Ophiostoma_floccosum             QN-DLKALSAALHARGMYLMV------------------DVVANHMGY-------DGAG------------------------------------------------------------
1_EPS26265_Penicillium_oxalicum             AA-DLKALSQALHSRGMYLMV------------------DVVANHMGY-------AGAG------------------------------------------------------------
1_AB042285_Phanerochaete_chrysosporium      AN-DLKALSDEVHRRGMYLMV------------------DVVVNNVMS---------TS------------------------------------------------------------
1_BAW15173_Pholiota_microspora              AD-DLKALSAEVHRRNMYLMV------------------DVVVNNVMA---------TT------------------------------------------------------------
1_BAF98616_Pichia_burtonii                  AD-ELKKLSEELHDRGMKLMV------------------DIVTNHYGA-------AGDG------------------------------------------------------------
1_AGJ52081_Rhizomucor_pusillus              ES-QLKTLIQAAHERGMYVML------------------DVVANHAGP---------TS------------------------------------------------------------
1_ADL28123_Rhizopus_oryzae                  AD-DLKKLVAAAHAKNMYVML------------------DVVANHAGT---------PS------------------------------------------------------------
1_ADD80242_Saccharomycopsis_fibuligera      AD-DLKSLAQELHDRDMLLMV------------------DIVTNHYGS-------DGSG------------------------------------------------------------
1_ABS76467_Saitozyma_flava                  AS-GLTDLSNALHNRGMYLMV------------------DVVVNHMAYYCGTNGGCGPG------------------------------------------------------------
1_CAB11471_Schizosaccharomyces_pombe        EQ-DLIDLADALHDRGMYLMV------------------DTVVNHMGS--------SDP------------------------------------------------------------
1_CAB40006_Schizosaccharomyces_pombe        ED-DLVSLITAAHKAGIWVML------------------DSIVNSMAL-------APPL------------------------------------------------------------
1_CAA34162_Schwanniomyces_occidentalis      AD-ELKQLASELHSRSMLLMV------------------DVVYNHYAW-------NGDG------------------------------------------------------------
1_CAA51912_Schwanniomyces_occidentalis      AD-DLKNLSNELHKRNMKLMV------------------DIVTNHYAW-------NGAG------------------------------------------------------------
1_ACN82436_Sclerotinia_sclerotiorum         PA-DLKALSAALHARGMYLMV------------------DVVTNHMGY-------LGCG------------------------------------------------------------
1_CAA03110_Thermomyces_lanuginosus          AD-DLKALADALHARGMLLMV------------------DVVANHFGY-------GGSH------------------------------------------------------------
1_BAG69580_Trichoderma_viride               AD-DLKSLVDAAHGKGFYMMV------------------DVVANHMGN---------AN------------------------------------------------------------
1_CAJ21046_Valsaria_rubricosa               AD-DLKNFVAAAHAKGMYVMV------------------DVVANHMGS---------CG------------------------------------------------------------
5_AWX66236_Alicyclobacillus_sp_18711        KA-QLQSAITSLHNNGIQAYG------------------DVVLNHRMGADAT-----ETISAVEVNPSNRN-----------------------QVTSGAYNIS---AWTD--FEFPG-
5_AAQ01675_Alkalimonas_amylolytica          RQ-QLQQALAALDQLGIQAYF------------------DVVFNHRMGADAQ-----EHI---------------------------------------PGFGL---AWTE--YHLQG-
5_CAL14744_Anoxybacillus_flavithermus       KT-QYIQAIQAAHTAGMQVYA------------------DVVFNHKAGADGT-----ELVDAVEVNPSDRN-----------------------QEISGTYQIQ---AWTK--FDFPG-
5_AEW07376_Bacillus_acidicola               KA-QYLQTIQAAHAAGMQVYA------------------DVVFDHKGGADGT-----EWVDAVEVNPSDRN-----------------------QEISGTYQIQ---AWTK--FDFPG-
5_AAA22191_Bacillus_amyloliquefaciens       KS-ELQDAIGSLHSRNVQVYG------------------DVVLNHKAGADAT-----EDVTAVEVNPANRN-----------------------QETSEEYQIK---AWTD--FRFPG-
5_ABY86223_Bacillus_cereus                  KA-QLKSAIDALHKKNIDVYG------------------DVVMNHKGGADYT-----ETVTAVEVDPSNRN-----------------------IEVSGDYEIS---AWTG--FNFPG-
5_CAD26699_Bacillus_halmapalus              RS-QLESAIHALKNNGVQVYG------------------DVVMNHKGGADAT-----ENVLAVEVNPNNRN-----------------------QEISGDYTIE---AWTK--FDFPG-
5_AAA22226_Bacillus_licheniformis           KG-ELQSAIKSLHSRDINVYG------------------DVVINHKGGADAT-----EDVTAVEVDPADRN-----------------------RVISGEHRIK---AWTH--FHFPG-
5_AEM05860_Bacillus_licheniformis           KG-ELQSAINSLHSRDINVYG------------------DVVINHKGGADAT-----EYVTAVEVDPADRN-----------------------RVTSGEQRIK---AWTH--FQFPG-
5_AAK00598_Bacillus_megaterium              KA-QLKSAIDALHKKNIDVYG------------------DVVMNHKGGADYT-----ETVTAVEVDPSNRN-----------------------VEVSGDYEIS---AWTG--FNFPG-
5_AGN35141_Bacillus_paralicheniformis       KG-ELQSAINSLHSRDINVYG------------------DVVINHKGGADAT-----EYVTAVEVDPADRN-----------------------RVTSGEQRIK---AWTH--FQFPG-
5_AAR68734_Bacillus_sp                      RS-QLQGAVTSLKNNGIQVYG------------------DVVMNHKGGADGT-----EMVNAVEVNRSNRN-----------------------QEISGEYTIE---AWTK--FDFPG-
5_AAA22231_Bacillus_sp_707                  RS-QLQAAVTSLKNNGIQVYG------------------DVVMNHKGGADAT-----EMVRAVEVNPNNRN-----------------------QEVTGEYTIE---AWTR--FDFPG-
5_BAF03567_Bacillus_sp_JAMB_204             KG-ELVSAITSLHNRGINVYG------------------DVVMNHKGGADFT-----ETVTAVEVNPNNRN-----------------------QETSGDYQIQ---AYTG--FNFPG-
5_CAC39917_Bacillus_sp_KSM_K38              KA-QLERAIGSLKSNDINVYG------------------DVVMNHKMGADFT-----EAVQAVQVNPTNRW-----------------------QDISGAYTID---AWTG--FDFSG-
5_AAB18785_Bacillus_sp_MK_716               KA-QYLQAIQAAHAAGMQVYA------------------DVVFDHKGGADGT-----EWVDAVEVNPSDRN-----------------------QEISGTYQIQ---AWTK--FDFPG-
5_AAA63900_Bacillus_sp_TS_23                KT-QYIQAIQAAKAAGMQVYA------------------DVVFNHKAGADGT-----EFVDAVEVDPSNRN-----------------------QETSGTYQIQ---AWTK--FDFPG-
5_ABW87262_Bacillus_sp_YX_YX1               KS-ELQDAIGSLHSRNVQVYG------------------DVVLNHKAGADAT-----EDVTAVEVNPANRN-----------------------QETSGEYQIK---AWTD--FRSPG-
5_AAF00567_Cytophaga_sp                     KG-ELKSAVNTLHSNGIQVYG------------------DVVMNHKAGADYT-----ENVTAVEVNPSNRN-----------------------QETSGEYNIQ---AWTG--FNFPG-
5_AAC74994_Escherichia_coli                 KA-QLLAAIDALKRNDIAVLL------------------DVVVNHKMGADEK-----EAIRVQRVNADDRT-----------------------QIDEEIIECE---GWTR--YTFPA-
5_AFZ41193_Exiguobacterium_sp_DAU5          KA-QLQSAISNLRGKGIGVYG------------------DVVMNHKGGADYT-----ESVQAVEVNPSNRN-----------------------QETSGEYSIS---AWTG--FNFAG-
5_AAA22235_Geobacillus_stearothermophilus   KA-QYLQAIQAAHAAGMQVYA------------------DVVFDHKGGADGT-----EWVDAVEVNPSDRN-----------------------QEISGTYQIQ---AWTK--FDFPG-
5_ABX83871_Geobacillus_thermodenitrificans  KA-QYLQAIQAAHAAGMQVYA------------------DVVFNHKGGADGT-----EWVLQISWWVDAVN-----------------------QEISGTYQIQ---AWTK--FDFPG-
5_AFC87833_Geobacillus_thermoleovorans      KA-QYLQAIQAAHAAGMQVYA------------------DVVFDHKGGADGT-----EWVDAVEVNPSDRN-----------------------QEISGTYQIQ---AWTK--FDFPG-
5_ACL70573_Halothermothrix_orenii           KG-ELENAIDALHNNDIKVYF------------------DAVLNHRMGADYA-----ETVLLDENSRDK-----------------------------PGQYIK---AWTG--FNFPG-
5_CAQ30277_Nostoc_sp_PCC_7119               RQ-QYLDAVKSLQTHGLQVYA------------------DAVLNHKMGGDAV-----ETPKATPFPQDDRL-----------------------NPKGGLQDIK---TYTH--YNFPG-
5_AAA27110_Salmonella_typhimurium           KR-QLLTAIDALKKNNIAVLL------------------DVVVNHKMGADEK-----ERIRVQRVNQDDRT-----------------------QIDDNIIECE---GWTR--YTFPA-
5_BAA24178_Streptococcus_equinus            KA-DYLQAISALKENHIAPLA------------------DVVLNHKAAADHT-----ETFSVVEVAPDDRT-----------------------KIISKPFDIE---GWTN--FTFDG-
5_AAA97431_Streptococcus_equinus            KA-DYLEAISALKNNGIEPLA------------------DVILNHKAAADHT-----ETFKVVEVAPEDRT-----------------------KVLSQPFEIE---GWTN--FTFEV-
5_AAN59233_Streptococcus_mutans             RE-DYLNAVNALKEQEIMPIS------------------DIVLNHKANGDAK-----ERFQVVKVNPSNRQ-----------------------EKISEPYEIE---GWTQ--FNFPG-
5_CCD30600_uncultured_bacterium             KE-ELESAIKILHGNGIKVYY------------------DAVLNHRMGADST-----ETVKLSLNSPDK-----------------------------PGQTIL---AWTK--FTFPG-
5_ALP73597_Vibrio_alginolyticus             KD-QYLSAINTAHKNNIQIYG------------------DVVFNHRGGADGK-----SWVDTKRVDWNNRN-----------------------IEL-GDKWIE---AWVE--FDFPG-
5_ABK62854_Histoplasma_capsulatum           KE-ELVALVTKAHEMEIRIIW------------------DTVLNHKAGADHT-----EKCNAVMVHPKDRR-----------------------KVISKPVEIE---AWLG--FTFPG-
5_ABS11196_Paracoccidioides_brasiliensis    KE-ELVSLVTRAHEMEIAVYW------------------DAVLNHKAAADYV-----EKCVAVMVDPKDRR-----------------------RVISEPQEIE---AWSG--FSFPG-
5_BAW15172_Pholiota_microspora              RE-ELLQACKVAKEHGIDILI------------------DAVLNHKLGADKT-----ETFRVIPVHETNRL-----------------------RDAGHARNIK---GWTA--FDFPG-
15_AAB60935_Aedes_aegypti                   EK-QFSDMVKRCMKAGVRIYV------------------DVVVNHMAAPGASA-------------------------------------------PLYGTAGS---TCDPLARDYPGV
15_AAB60934_Aedes_aegypti                   EA-EFASMVRRCNNVGVRIYV------------------DIVINHMAAI---S-------------------------------------------GQGTGGS------QVNGLNFPAV
15_AAA03322_Aedes_atropalpus                ES-EFASMVRRCNQVGVRIYV------------------DIVINHMAAM---S-------------------------------------------GQGTGGS------SVDGLNFPAV
15_AAA03323_Anopheles_merus                 EA-EFADMSRRCNAAGVRLYV------------------DIIINHMGATQPVE-------------------------------------------PAIGTGSS---TAIPSDRQFPAV
15_AAN77138_Anthonomus_grandis              EA-ALADMIKRCNNVGVRIYA------------------DLVVNHMAAS-----------------------------------------------TGIGTAGH---TCDPGSKSYPAV
15_AAN77139_Anthonomus_grandis              EE-AFKDMVKRCNAVGVRVYP------------------DVVVNHMASI-----------------------------------------------STEGTAGD---VCDPTNRDYPAV
15_AAO13755_Asterias_rubens                 PA-EFKDMVSRCYKAGVRIYV------------------DAVINHMAASNPGG---------------------------------------------------------KDAWDFGLV
15_AAY23288_Blattella_germanica             ES-AFRDMVQRCNKEGVRIYV------------------DVILDHMTSNYP---------------------------------------------NAHGVGGN---TANPSNFEYPAI
15_BAB72257_Callosobruchus_chinensis        EG-ALKSMLSRCNKAGIRVYA------------------DLVINHMAAA-----------------------------------------------SGSGTAGH---SCDAGSRSYPTV
15_AAA03350_Culex_tarsalis                  EQ-EFASMVRRCNDVGVRIYV------------------DIVINHMAAI---T-------------------------------------------GDGGTGGS---TGSSQTLSFPGV
15_AAF20998_Diabrotica_virgifera_virgifera  ET-AFANIVRRCNNAGVRIYV------------------DVVINHMSGI-----------------------------------------------TGNGTGGK---SSDAGSLSYPGV
15_AAC35243_Drosophila_ananassae            EE-QFARMVRRCNNVGVRIYV------------------DVVFNHMAAD---G-------------------------------------------GTVGTGGS---IANPSVKSYPAV
15_BAB91544_Drosophila_bocki                EE-QFASMTRRCNAVGVRIYV------------------DVIFNHMAAD---G-------------------------------------------GTYGTGGS---TASPSSKSYPGV
15_BAA04582_Drosophila_erecta               EQ-QFASMVRRCNAVGVRIYV------------------DVVFNHMAAD---G-------------------------------------------GTYGTGGS---TASPSSKSYPGV
15_BAB91509_Drosophila_kikkawai             EE-QFASMTRRCNAVGVRIYV------------------DVIFNHMAAN---G-------------------------------------------GTYGTGGS---TASPSSKSYPGV
15_BAA95437_Drosophila_leontia              EE-QFASMTRRCNAVGVRIYV------------------DVIFNHMAAD---G-------------------------------------------GTYGTGGS---TASPSSKSYPGV
15_BAA95441_Drosophila_lini                 EE-QFASMTRRCNAVGVRIYV------------------DVIFNHMAAD---G-------------------------------------------GTYGTGGS---TASPSSKSYPGV
15_BAA04583_Drosophila_mauritiana           EQ-QFASMVKRCNAVGVRIYV------------------DVIFNHMAAD---G-------------------------------------------GTYGTGGS---TASPSSKSYPGV
15_AAK93479_Drosophila_melanogaster_Amyrel  EE-EFGDMVRRCNDVGVRIYV------------------DVLLNHMSGDFD-G-------------------------------------------VAVGTAGT---EAEPSKKSFPGV
15_CAA28238_Drosophila_melanogaster         EE-QFASMVKRCNAVGVRTYV------------------DVVFNHMAAD---G-------------------------------------------GTYGTGGS---TASPSSKSYPGV
15_AAC46839_Drosophila_pseudoobscura        EE-QFASMVRRCNNAGVRTYV------------------DVIFNHMAAD---G-------------------------------------------GTYGTAGS---TASPSSKSFPAV
15_BAA04585_Drosophila_sechellia            EQ-QFASMVKRCNAVGVRIYV------------------DVIFNHMAAD---G-------------------------------------------GTYGTGGS---TASPSSKSYPGV
15_AAC46463_Drosophila_virilis              EE-QFANMVRRCNNVGVRTYV------------------DVVFNHMAAD---G-------------------------------------------GTTGTGGS---NATPSSKSLPAV
15_AAG60011_Drosophila_yakuba               EQ-QFASMVKRCNAVGVRTYV------------------DVVFNHMAAD---G-------------------------------------------GTYGTGGS---TASPSTKSFPGV
15_CAA76926_Phaedon_cochleariae             ES-ALASMIRRCNNAGVRIYV------------------DAVFNHMSAT-----------------------------------------------SGIGTGGS---SCDVEPSASPAV
15_P56634_Tenebrio_molitor                  ES-AFTDMTRRCNDAGVRIYV------------------DAVINHMTGM-----------------------------------------------NGVGTSGS---SADHDGMNYPAV
15_AAA03708_Tribolium_castaneum             EA-ALADMISRCNAVGVRIYV------------------DTVINHMTGM-----------------------------------------------GGTGTAGS---QADRDGKNYPAV
15_AAF73435_Zabrotes_subfasciatus           EG-ALADMIKRCNNAGVRVYA------------------DVVFNHMAAK-----------------------------------------------GGSGTGGN---NCDPSKKSYPAV
24_ABL09312_Acarus_siro                     EE-QFKGMVQRCNKVGVRIYV------------------DIVMNHMSGAQEGH----------------------------------------------GNCWF---KLQWHHDVSRCS
24_BAB85635_Anguilla_japonica               EK-ELRDMVTRCNNVGVNIYV------------------DAVINHMCGAGGGE-------------------------------------------GKHSSCGS---YFNSHKKDFPAV
24_AAL37207_Crassostrea_gigas               EA-DLRDMIQRCNKVNVRIYA------------------DVVFNHMTGSGA-S-------------------------------------------GT-GTGGS---HWDSGTLSYPGV
24_AAL37183_Crassostrea_gigas               EA-DLRDMVQRCNKVNVRIYA------------------DVVINHMTGAGG-S-------------------------------------------GT-GTGGS---HWDGGSLSYPGV
24_AAD38942_Dermatophagoides_pteronyssinus  EQ-QFRRMVKRCNKAGVRIYV------------------DIVLNHMTGAQSGK----------------------------------------------GTNGH---HYDGNTLQYPGV
24_AAD38943_Euroglyphus_maynei              EQ-QFRRMVKRCNKAGVRIYV------------------DIVLNHMTGGQSGQ----------------------------------------------GTNGN---HYNGITMQYPGV
24_AAC60246_Gallus_gallus                   EN-EFRDMVTRCNNVGVRIYV------------------DAVVNHMCGSMGGT-------------------------------------------GTHSTCGS---YFNTGTRDFPAV
24_AB026610_Haliotis_discus_discus          EQ-DLKDMVSRCNKAGVRIYA------------------DTVINHMTGVGG-T-------------------------------------------GQ-GTAGS---SYDANQLKYPGV
24_BAM74656_Haliotis_discus_hannai          EQ-DLKDMVSRCNKAGVRIYA------------------DTVINHMTGVGG-T-------------------------------------------GQ-GTAGS---SYDANQLKYPGV
24_AAA51724_Homo_sapiens_pancreatic         ED-EFRNMVTRCNNVGVRIYV------------------DAVINHMCGNAVSA-------------------------------------------GTSSTCGS---YFNPGSRDFPAV
24_AAH63129_Homo_sapiens_salivary           ED-EFRNMVTRCNNVGVRIYV------------------DAVINHMCGNAVSA-------------------------------------------GTSSTCGS---YFNPGSRDFPAV
24_AAA37221_Mus_musculus                    ED-EFRDMVNRCNNVGVRIYV------------------DAVINHMCGVGAQA-------------------------------------------GQSSTCGS---YFNPNNRDFPGV
24_AAA37230_Mus_musculus                    ED-EFRDMVTRCNNVGVRIYV------------------DAVINHMCGAGNPA-------------------------------------------GTSSTCGS---YLNPNNREFPAV
24_H2N0D4_Oryzias_latipes                   EN-ELRDMITRCNNVGVNVYV------------------DAVINHMCGAGGGE-------------------------------------------GTHSSCGS---WFNANNKDFPSV
24_CAA68065_Pecten_maximus                  ED-QLRDMISRCNRVNVRIYS------------------DTVINHMTGVGG-S-------------------------------------------GT-GTAGS---HWNGDTLSYPGV
24_CAA54524_Penaeus_vannamei                EN-AFKDMVTRCNNVGVRIYV------------------DAVINHMSGG-WPM-------------------------------------------GTGASGGS---SFDSGAESYPGV
24_CAB65552_Penaeus_vannamei                EN-AFKDMITRCNNVGVRIYV------------------DIVVNHMTGW-WPS-------------------------------------------GTGSTGGS---SFDSGAQSYPGV
24_AAF65827_Pseudopleuronectes_americanus   ED-ELRDMITRCNNVGVNIYV------------------DAVINHMCGASGGE-------------------------------------------GTHSSCGS---WFDAGKKDFPSV
24_AAA40725_Rattus_norvegicus               EN-EFKDMVTRCNNVGVRIYV------------------DAVINHMCGSGNSA-------------------------------------------GTHSTCGS---YFNPNNREFSAV
24_AAH88228_Rattus_norvegicus               ED-EFRDMVNRCNNVGVRIYV------------------DAVINHMCGVGAEA-------------------------------------------GQSSTCGS---YFNPNNRDFPGV
24_P83053_Struthio_camelus                  EN-EFRDMVTRCNNVGVRIYV------------------DAVKNHMCGSGAGS-------------------------------------------GTHSTCGA---YFNAGNRDSPAV
24_AAF02828_Sus_scrofa                      EN-EFRDMVTRCNNVGVRIYV------------------DAVINHMCGSGAAA-------------------------------------------GTGTTCGS---YCNPGNREFPAV
24_CAC87125_Tetraodon_nigroviridis          EN-ELRDMITRCNNVGVNIYV------------------DVVINHMCRASGGE-------------------------------------------GTHSSCGS---WFNAGAKDFPSV
32_AAA21016_Aeromonas_hydrophila            EA-ELRSMIARCNAAGVKVYA------------------DAVFNHMASG-----------------------------------------------SGTATGGG---SYNSGQ--YQYP
32_BAA22082_Bacillus_sp_195                 RA-EFKAMVDTCHAAGVKVIA------------------DAVINHMTGQSAG---------------------------------------------GTGWAGS---TFQH--YDYPG-
32_AAY89038_Bifidobacterium_breve           EA-EFKTMIATCNAAGVEIIA------------------DSVINHTTGADQ-G-------------------------------------------EGTGVAGS---KYDG-EGNFPAI
32_CAB92963_Halomonas_meridiana             SE-AFADMVQRCNAAGVDVYA------------------DAVINHVAHG-----------------------------------------------KGQGIAGS---SYDSEA--LSYP
32_BAJ52728_Kocuria_varians                 RA-DYAAMVQNCHDAGVEVIA------------------DVVINHMAGQES----------------------------------------------GTGWAGS---AFTH--YDYPG-
32_AKG25402_Microbacterium_aurum            EA-EFKSMVQRCNAAGVGVIV------------------DAVINHMSGQSNG---------------------------------------------GTGWAGT---VFQH--YNYPG-
32_CAA41481_Pseudoalteromonas_haloplanktis  RA-QFIDMVNRCSAAGVDIYV------------------DTLINHMAAG-----------------------------------------------SGTGTAGN---SFGNK----SFP
32_AAA86835_Pseudomonas_sp_KFCC10818        RA-QFADMVQRCKNVGVDIYV------------------DAVINHMAAW-----------------------------------------------NRN----------------FPEV
32_AAA96317_Streptomyces_albus              RA-AFRSMVNTCHAAGVKVVV------------------DTVINHMSAGS-----------------------------------------------GTGTGGS---SYTK--YDYPG-
```

```
                                                                      CSR-I
32_BAC73693_Streptomyces_avermitilis        AT-AFQNMINTCHTAGVKVVV------------------DTVVNHMSAGS-----------------------------------------------GTGTGGS---AYTK--YNYPG-
32_CAA40798_Streptomyces_griseus            RA-AFKSMVDTCHAAGVKVVA------------------DSVINHMAAGS-----------------------------------------------GTGTGGS---AYQK--YDYPG-
32_AAA26698_Streptomyces_hygroscopicus      RD-AFASMVSACHAAGVKVIA------------------DAVVNHMAAGS-----------------------------------------------GR--HHA---QYTK--YNYPG-
32_AAA88554_Streptomyces_limosus            RA-AFKSMVDTCHAAGVKVVA------------------DSVINHMAAGS-----------------------------------------------GTGTGGS---AYQK--YDYPG-
32_CAB06622_Streptomyces_lividans           RA-QFKSMVDTCHAAGVKVVA------------------DSVVNHMSAGN-----------------------------------------------GTGTGGS---SYTK--YDYPG-
32_CAA73775_Streptomyces_sp_TO1             RT-AFRNMVNTCHAAGVKVVA------------------DTVINHMSAGS-----------------------------------------------GTGTGGS---SYTK--YSYPG-
32_AAA82875_Streptomyces_sp_WL6             RD-GFAAMVDACHSAGVKVVA------------------DAVINHMAAGS-----------------------------------------------GTGTGGT---AYTK--YDYPG-
32_AAA26697_Streptomyces_thermoviolaceus    RA-AFRSMVNTCHAAGVKVVV------------------DTVINHMSAGS-----------------------------------------------GTGTGGS---SYTK--YDYPG-
32_AAB36561_Streptomyces_venezuelae         RT-AFKNMIDTCHAAGVKVVA------------------DSVINHMANGS-----------------------------------------------GTGTGGT---SFSK--YDYPG-
32_ABF13430_Thermobifida_fusca              RA-DFIDMVNTCREAGVKIYV------------------DAVINHMTGTGSAG-------------------------------------------AGPGSAGS---SYSK--YDYPG-
32_CAA41881_Thermomonospora_curvata         RA-DFIDMVNTCREAGVKIYV------------------DAVINHMTGTGSAG-------------------------------------------AGPGSAGS---SYSK--YDYPG-
32_BAV60949_Pholiota_microspora             RS-QLQNMINTCHSAGVKVIA------------------DTLWNHMAGVDS----------------------------------------------GTGVAGS---SFTH--YNYPGI
32_KJA26527_Hypholoma_sublateritium         RA-QFANMITTCHAAGVKVIA------------------DTIWNHMAGIDS----------------------------------------------GTGVDGS---TFTH--YVYPGI
32_KDR78015_Galerina_marginata              RS-QFQNMISACHTAGVKVIV------------------DTIFNHMAGIDS----------------------------------------------GIGVAGS---SFTH--YVYPGI
32_PPQ83160_Psilocybe_cyanescens            RA-QFQNMVTVCHTAGVKVIA------------------DTIFNHMAGVDS----------------------------------------------GTGTAGS---SFTH--YVYPGI
32_KTB32361_Moniliophthora_roreri           RQ-QFQNMVSACHAAGVKVIA------------------DTILNHMAGSDS----------------------------------------------GTGVAGS---SFTH--YNYPGI
32_KDQ30003_Pleurotus_ostreatus             RS-QFANMVSTCHSAGVKVIT------------------DTIFNHMAGVES----------------------------------------------GTGVGGS---SFTH--YNYPGI
32_RDX41646_Polyporus_brumalis              RD-QFASMISTCHAAGVGVIV------------------DTIWNHMAGVDS----------------------------------------------GTGVAGS---SFTH--YNYPGI
32_OJT13448_Trametes_pubescens              RT-SFANMITTCHAAGVGVIV------------------DTIWNHMAGVDS----------------------------------------------GTGIAGS---SFTH--YVYPGI
32_PFH45132_Amanita_thiersii                RS-QFQSMVSACHSVGVGVIA------------------DTLFNHMSGSDS---------------------------------------------GGTGVAGS---SFTH--YDYPGI
32_KIM37787_Hebeloma_cylindrosporum         RS-QFQNMISTCHTAGVKVLV------------------DTIFNHMAGVSS----------------------------------------------GTGMGGS---SFTH--YNYPGI
32_PBK61492_Armillaria_solidipes            RD-EFANMITTCHAAGVKVIA------------------DTIFNHMTGMDS----------------------------------------------GIGVASD---TYTH--YVYPGI
32_ETW76368_Heterobasidion_irregulare       RS-QYKNMIDTCHAAGVGVIA------------------DTIFNHMAGMDS----------------------------------------------GTGVAGS---SFTH--YNYPGI
32_RDB27268_Hypsizygus_marmoreus            RG-QFQKMISACHGAGVKVIA------------------DTIFNHMAGIDS----------------------------------------------GTGVGGS---SFTH--YNYPGI
32_PPQ71147_Gymnopilus_dilepis              RS-QFKNMIDTCHAAGVGVIV------------------DTIWNHMSGNDN----------------------------------------------GTGVAGS---PYTH--YEYNGI
32_OSD07916_Trametes_coccinea               RT-QFANMISTCHAAGVGVIA------------------DTIFNHMAGVDS----------------------------------------------GTGVAGS---SFTH--YNYPGI
32_KZW01389_Exidia_glandulosa               RS-QFANMISTCHAAGVKVIV------------------DTLWNHMAGIES----------------------------------------------GTGVGGS---SFTH--YNYPGI
32_EIW55835_Trametes_versicolor             RT-SFANMITTCHAAGVGVIV------------------DTIWNHMAGVDS----------------------------------------------GTGIAGS---SFTH--YVYPGI
32_SJL11930_Armillaria_ostoyae              RD-EFANMITTCHAAGVKVIAGIPRSFYHSDSIAIEKPVDTIFNHMTGMDS----------------------------------------------GIGVASD---TYTH--YVYPGI
32_PAV18085_Pyrrhoderma_noxium               RS-QYQNMINTCHAAGVKVIA------------------DTIFNHMTGEDS----------------------------------------------GTGTAGS---SFTH--YVYPGI
32_RPD65356_Lentinus_tigrinus               RD-QFASMISTCHQAGVGVIV------------------DTIWNHMAGVDS----------------------------------------------GTGIAGS---SFTH--YNYPGI
32_PBK84861_Armillaria_gallica              RD-EFANMITTCHAAGVKVIA------------------DTIFNHMTGMDS----------------------------------------------GTGVAGD---TYTH--Y-----
32_KLO18298_Schizopora_paradoxa             RA-QYQNMINTCRAAGVGVIA------------------DTIWNHMSGLDS----------------------------------------------GVGVAGS---SFTH--YNYPGI
32_PPR08271_Panaeolus_cyanescens            RS-QFEKMVSACHSAGVKVIT------------------DTIWNHMAGVDS----------------------------------------------GVGVGGS---SFTH--YDYPGI
32_KIK53845_Gymnopus_luxurians              RA-QFQNMIQTCHTAGVLVIA------------------DTLFNHMTGNAQ----------------------------------------------GTGVAGS---TYTQ--FVYPGI
32_KIJ56431_Sphaerobolus_stellatus          RQ-QFSNMISTCHAAGVRVIA------------------DTLFNHMVS--S----------------------------------------------GSSVVAV---NFTH--YVYPGI
32_EJD07843_Fomitiporia_mediterranea        RD-QYTNMINTCHASGVRVIA------------------DTIFNHMSGSDS----------------------------------------------DVGVAGS---SFTH--YDYPGI
32_EPQ53992_Gloeophyllum_trabeum            RQ-QYQNMVTTCHAAGVRVIA------------------DTIFNHMSGSDS----------------------------------------------GTGTAGT---SFTH--YNYPGT
32_KIP11288_Phlebiopsis_gigantea            RA-QYANMITTCHAAGVNVIA------------------DTIFNHMTGSDS----------------------------------------------GTGVAGS---TFQH--YVYPGI
32_KIY62009_Cylindrobasidium_torrendii      RA-QFANMITTCHAAGVGVIA------------------DTILNHMAGSDS----------------------------------------------GSGVAGS---SYTH--YNYPGI
32_KIJ69360_Hydnomerulius_pinastri          RT-QYANMIQTCEAAGVGIIA------------------DTIFNHMTGESN----------------------------------------------GTGIAGD---SFTQ--YAYPAV
32_KZT20455_Neolentinus_lepideus            ET-QYQNMIKTCHAAGVKIIA------------------DTLFNHMAGADS----------------------------------------------GTGVAGS---TFTH--YDYPGI
32_EGN97351_Serpula_lacrymans_var_lacrymans RA-QYQSMIQTCKSAGVGIIA------------------DTIFNHMTGQSS----------------------------------------------GTGIAGT---QFTQ--YSYPP-
32_KDQ06010_Botryobasidium_botryosum        RQ-QFANMISACHSAGVGVIV------------------DTLWNHMAGIES----------------------------------------------GTGTGGS---FFTH--YNYPGI
32_PPR00074_Panaeolus_cyanescens            RA-QFANMVNTCRAAGVSIIA------------------DTIWNHMAGIGG----------------------------------------------GTGTGGS---SFTQ--YNYPGL
32_EKM53319_Phanerochaete_carnosa           RD-QYTNMINTCHAAGVGVIA------------------DTLLNHMSGSDD---------------------------------------------GGVGVGGS---SFQH--YVYPGI
32_TBU45601_Dichomitus_squalens             RD-QFSSMISTCHAAGVGVIV------------------DTLWNHMTGSDS----------------------------------------------GTGVAGS---SYTH--YNYPGI
32_PSR82077_Phlebia_centrifuga              RS-QFANMITTCHAASVQVIA------------------DTIFNHMTGSDS----------------------------------------------GSGVAGS---TYTH--YVYPGI
32_KIM89391_Piloderma_croceum               RT-QFSNMITTCHAAGVKVIA------------------DAIFNHMTGG-S----------------------------------------------GIGVAGS---PYTH--YVYPGT
32_PIL31175_Ganoderma_sinense               RA-QFANMIATCHAAGVGVIA------------------DTIWNHMTAKDN----------------------------------------------GTGVAGS---SYTH--YTYPGI
32_KIY70325_Cylindrobasidium_torrendii      RA-QFASMISTCHAAGVNVIA------------------DTILNHMAGSDS----------------------------------------------GSGVAGS---SFTH--YNYPGI
32_KDQ58060_Jaapia_argillacea               RA-QYQNMITTCHAAGVRVIA------------------DTIWNHMTGVAS----------------------------------------------GTGTAGS---SFTQ--YDYPGV
32_CEL62905_Rhizoctonia_solani              RS-QFQNMISKCKSAGVGVIA------------------DTIFNHMAGIEG----------------------------------------------GTGVAGS---SFTH--YNYPGI
32_GAT57350_Mycena_chlorophos               QA-QFSAMISACHSAGVRVIA------------------DTIFNHMAGVDS----------------------------------------------GVGVAGS---SFTH--YNYPGI
32_KIM25608_Serendipita_vermifera           RS-QFASMVTTCHNAGVKVLV------------------DTIMNHMAGIDS----------------------------------------------GTGVAGS---SFTH--YNYPGI
32_GAW02849_Lentinula_edodes                RA-QFQSMIETCHAAGVLVIA------------------DTIFNHMTGNAE----------------------------------------------GSGVAGS---TYTQ--FVYPGI
32_TCD63303_Steccherinum_ochraceum          RD-QYANMINTCHSAGVKIIA------------------DTIMNHMSKVES----------------------------------------------GSGVGGS---SFTH--YDYPGT
32_KZP22139_Fibularhizoctonia_sp_CBS_109695 RA-QFASMITTCHAAGVKVIA------------------DTVFNHMSAQSS----------------------------------------------GTGTAGD---SYTM--YSYPTA
32_KII90441_Plicaturopsis_crispa            RD-QFSSMIKTCNSAGVQVIA------------------DTVLNHMSAPGS----------------------------------------------GTGTAGDCKSAYTS--FNYPTV
32_KDQ58058_Jaapia_argillacea               RA-QFQNMINTCHQAGVRVIA------------------DTIWNHMAGANS----------------------------------------------GIGVAGS---TFTH--YNYPGI
32_OCH93218_Obba_rivulosa                   RD-QYADMVSRCNAVGVGIIA------------------DTIFNHMTGESS----------------------------------------------GTGVAGS---SFTQ--FNYPGI
32_EMD36390_Gelatoporia_subvermispora       RD-QYTDMVSRCNAVGVGIIA------------------DTIFNHMTGESN----------------------------------------------GTGVAGS---SFTQ--YNYPGI
32_EIW78746_Coniophora_puteana              RD-QFQNMVSTCATAGVSVIA------------------DTVLNHMTA-GG----------------------------------------------GTGTDGN---SYTQ--FDYAAV
32_KII86244_Plicaturopsis_crispa            RD-QFSNMVSTCHNAGVKVIA------------------DTLFNHMAGIDS----------------------------------------------GTGVGGS---SFTH--YVYPGI
32_EKM75205_Agaricus_bisporus               RS-QFQNMINACHSAGVGVIV------------------DTIFNHMAGIDS----------------------------------------------GSGVGGS---SFSH--YNYPGI
32_KZT36083_Sistotremastrum_suecicum        RA-QYQSMIQTCHAAGVGVIA------------------DTIWNHMAGISG----------------------------------------------GVGVAGS---SFTK--FNYPGI
32_KZS90559_Sistotremastrum_niveocremeum    RA-QYQSMIQTCHAAGVGVIA------------------DTIWNHMAGISG----------------------------------------------GVGVAGS---SFTK--FNYPGI
32_EFI98134_Schizophyllum_commune           RD-GFAAMVEACHDAGVGVIA------------------DTIWNHMAGVDS----------------------------------------------GTGTAGS---SFTH--YDYPGI
32_BBC62173_Tricholoma_matsutake            RT-QFQNMIDTCHNAGVKVIA------------------DTIFNHMAGSDS----------------------------------------------GSGVAGS---PFTH--YNYPGI
32_CCA69081_Serendipita_indica              RS-EFANMVTTCHNAGVLVLV------------------DTILNHMAGINS----------------------------------------------GTGVAGS---TFTH--YVYPGI
32_EFI98135_Schizophyllum_commune           RT-QFATMVATCHAAGVKVIV------------------DTIWNHMSGQDS----------------------------------------------GVGTGGS---HFTH--YDYPGI
32_KII85613_Plicaturopsis_crispa            RE-QFSAMIQTCSAAGVGVIA------------------DVVWNHMQAGGS----------------------------------------------AVGVGGT---QVTR--YSYAGI
32_EIN11730_Punctularia_strigosozonata      RD-EFAAMVTTCNAAGVKIIA------------------DTIWNHMAGIDS----------------------------------------------GSGSAGS---SFTH--YDYPAV
32_PIL33242_Ganoderma_sinense               RD-QFANMVKTCHSAGVGVIV------------------DTLWNHMSGLDS----------------------------------------------ATGYAGS---SFTK--YNYPGI
32_KZT18190_Neolentinus_lepideus            RE-QFASMITRCHAAGVKVIA------------------DTLFNHMTAMDS----------------------------------------------GKGTAGS---PYSH--YDYPGI
32_KZV75308_Peniophora_sp_CONT              RD-SYKNMIDTCHAAGVKVIV------------------DTVINHMAGIDS----------------------------------------------GTGTAGT---SFTH--YTYDGY
32_KII86838_Plicaturopsis_crispa            RA-QFAAMVKTCSDAGVGVIA------------------DVLWNHMQGGDS----------------------------------------------ATGVGGT---VVTH--YNYPGM
32_KDQ08018_Botryobasidium_botryosum        RQ-QFSDMIQACHGAGVGVIV------------------DTIWNHMAGIDS----------------------------------------------GTGVGGT---CFTH--YNYPGL
32_KIY73061_Cylindrobasidium_torrendii      RD-QFSSMVSACHDAGVSVIA------------------DTILNHMAGTES----------------------------------------------GTGTGGS---SFTH--YNYPDI
32_KZW01365_Exidia_glandulosa               RA-ALASMIEACHKVGVKVIA------------------DAVINHMTGSD---A-------------------------------------------GTGTAGT---SYQH--YEYPGL
32_KDQ58178_Jaapia_argillacea               RS-QYQAMVQTCHAAGVLVIA------------------DTILNHMSGQD---N------------------------------------------GGTGVAGT---QFQH--YEYPGL
32_KZV75307_Peniophora_sp_CONT              RA-SFKNMVDTCHAAGVKVIA------------------DAVLNHMTGSDS----------------------------------------------GTGTAGT---SYTH--YVYNGF
32_KIK51212_Gymnopus_luxurians              RA-QFQNMINTCHAAGVKVIA------------------DTIFNHMTGSAS----------------------------------------------GSGVAGS---TYTE--YVYPGI
32_RDX41931_Polyporus_brumalis              RQ-QFQNMVNACHNAGVSVIV------------------DTIWNHMAGLDS----------------------------------------------GSGNAGS---SFTK--YNYPGI
32_TBU29254_Dichomitus_squalens             RD-QFANMVQACHAAGVGVIV------------------DTIWNHMSGVDS----------------------------------------------GNGYAGS---SFTK--YNYPGI
32_KIY45802_Fistulina_hepatica              RE-EFQSMITTCHNAGVKVIA------------------DVVMNDMTGYE--SG--------------------------------------------TGTAGS---SYTH--YDYPGI
32_KIL59382_Amanita_muscaria                RV-QFQQMVTQCHDAGVGVIA------------------DSILNHMTGMDS----------------------------------------------GTGVSGS---TFTH--YDYPGL
32_KIY53725_Fistulina_hepatica              KE-ELASMVSTCHDAGVSVII------------------DILFNAMAGID--SG--------------------------------------------TGIDGS---TFTH--YDYPDL
32_KIY47132_Fistulina_hepatica              RD-EFASMIQQCHDADVQIVV------------------DAVFNHMTAQQ--NG--------------------------------------------TGIAGT---KFTH--YYYPGN
32_RPD55342_Lentinus_tigrinus               RE-QFQNMVNACHNAGVSVIV------------------DTIWNHMAGLDS----------------------------------------------GSGTAGS---SFTK--YNYPGI
32_TCD61445_Steccherinum_ochraceum          RD-DLKNMIDTCHAAGVKVLV------------------DTVWNHMAGIDS----------------------------------------------GTGTAGD---SFTH--YSYPPT
32_KIM25602_Serendipita_vermifera           RE-EFASMIATCNAAGVQVIA------------------DAVTNHMAGGSS----------------------------------------------GVGTAGS---TFTH--YDYPGT
32_EJD37603_Auricularia_subglabra           RE-QLATMIDACHRVGVKVYA------------------DAVINHMTGSD---A-------------------------------------------GMGTAGS---SYEH--YNYPGI
32_KZT54766_Calocera_cornea                 RD-QFATMVKTCNAAGVQVIA------------------DTIMNHMSGQS---S-------------------------------------------GTGLGGT---IFSH--YSYPGL
32_KIY46805_Fistulina_hepatica              RA-QFERMVDACHSAGVKVLA------------------DTLFNHMTAKD--SG--------------------------------------------VGTAGS---SFTH--YNYPGV
32_PPQ69654_Psilocybe_cyanescens            RS-QFRNMITTCHAAGVRVIV------------------DTIFNHMAGTDS----------------------------------------------GTGTAGS---SFTH--YVYPGT
32_KZO93971_Calocera_viscosa                RD-QFAAMVKSCNAAGVQVIA------------------DTIMNHMSAQTS----------------------------------------------GTGVGGT---EYSH--YSYPGI
32_KZP07860_Fibularhizoctonia_sp_CBS_109695 RA-EYEAMVSACNAAGVGVIA------------------DAVLNHMSGSA--FG------------------------------------------EPGDAAGS---TFST--FAYPAV
32_PVG03687_Serendipita_vermifera           RE-QFAAMIKACNAAG--VIA------------------DAVTNHMSGAE---S-------------------------------------------GVGTANS---KFTH--YDYPGT
32_EIM87674_Stereum_hirsutum                EA-EFASMVSTCADAGVGIIV------------------DAVTNHMTAG-----------------------------------------------SGTGFAGN---TYSK--YDYPAV
32_CCA69087_Serendipita_indica              RE-EFIDMVNTCKTAGVQVIA------------------DAVVNHMSGAS---A-------------------------------------------GTSTLGT---NFTH--YDYPGT
32_CEL62035_Rhizoctonia_solani              RS-EFAAMIGKCKGAGVKVMV------------------DTIWNHMAGVD---N-------------------------------------------GVGTAGT---QFSH--YDYPGL
32_EIN05398_Punctularia_strigosozonata      RD-QFANMVSTCKSAGVNIIA------------------DVIWNHMAGTDS----------------------------------------------GSGVAGN---TFTH--YDYPGT
32_EJU02896_Dacryopinax_primogenitus        RA-QFANMVQACRTAGVQVIA------------------DTVLNHMSAQG---S-------------------------------------------GTGVGGT---VYSH--YSYPGI
32_KIO30302_Tulasnella_calospora            RD-KFANMISTCHAAGVGVIV------------------DTIWNHMAGISS----------------------------------------------GTGTAGT---AFST--FRSEDL
32_CEL62036_Rhizoctonia_solani              RS-DFAEMVGRCKNAGVRVSV------------------DMIWNHMAGVD---N-------------------------------------------GDGNAGT---HFSH--YNYPGL
32_KIO24533_Tulasnella_calospora            AD-QFRNMVNACKTAGVGVIV------------------DIVWNHMTGID---G-------------------------------------------GVGVAGS---SFSH--YDYPGI
32_EIM84100_Stereum_hirsutum                RD-EFSSMVTTCKNAGVGVLV------------------DAIWNHMAGSD---S-------------------------------------------GTGVAGT---SWTH--YNYPGY
32_KDN35973_Rhizoctonia_solani              RR-QFENMVAICKNAGVGIIA------------------DAVINHMSALN---N-------------------------------------------GTGVAGS---NFTH--YNYPGI
32_KIY48489_Fistulina_hepatica              SD-SFKSMVDTCHEAGVRVLS------------------DVILNHMAGFD---N-------------------------------------------GTGTAGD---TFTH--YNYTEY
32_KIY48486_Fistulina_hepatica              RD-EFQSMVETCHAAGVKVVA------------------DIVINHMCGFD---S-------------------------------------------GTGTAGD---TFTH--YSYVDY
32_KZP26813_Fibularhizoctonia_sp_CBS_109695 PD-EFDTMVSTCSKAGVSVIA------------------DVVLNHMTA-G--SG--------------------------------------------TGVGGN---AYTQ--YSYPAV
32_KDQ32476_Pleurotus_ostreatus             RE-QFRQMVTKCHAAGVKVIT------------------DTLFNHMSSLS----------------------------------------------SGKGTGGS---TFDK--YNYPGL
32_EIM84087_Stereum_hirsutum                AD-QFANMINSCKNAGVAVLL------------------DTCWNHMSGTY---E-------------------------------------------GNGVGGS---SFKQ--YEYPGF
32_KDQ32475_Pleurotus_ostreatus             RD-QFKRMVEKCHIAGVKVIT-----------------VDTLFNHISAIQ---G------------------------------------------PATGTAGS---TFSK--YDYPGL
32_EAQ88347_Chaetomium_globosum             RE-SFQSMVNTCHSAGVKVIV------------------DSVINHMSAGG-----------------------------------------------GTGTGGS---SYTK--YNYPG-
32_ORY89754_Leucosporidium_creatinivorum    RE-QFESMVKTCAEAGVQIIV------------------DAVLNHMASSG----------------------------------------------SGTGTGGS---SYSE--YDYPAV
32_EWZ34052_Fusarium_oxysporum              RR-SFRNMTDTCHRAGVKVIV------------------DTVINHMSARS-----------------------------------------------GTGTGGS---SYSK--YNYPG-
32_OBT91518_Pseudogymnoascus_verrucosus     RT-SFQNMINTCHGAGVKVVV------------------DTVINHMSAGS-----------------------------------------------GTGTGGT---SYTK--YNYPG-
32_KEY73157_Stachybotrys_chartarum          RA-AFQNMVNTCHSAGVLVVV------------------DTIINHMSAGS-----------------------------------------------GTGTGGS---SYTK--YNYPG-
32_OBT62577_Pseudogymnoascus_sp_23342_1_I1  RT-AFQNMVNTCHTAGVKVIA------------------DTVINHMSSGS-----------------------------------------------GTGTGGS---SYTK--YNYPG-
32_EON97874_Phaeoacremonium_minimum         RA-SFVNMVNTCHGAGVKVIA------------------DAVINHMSSGS-----------------------------------------------GTGTGGS---SYTK--YNYPG-
32_GAT55198_Mycena_chlorophos               RE-QFANMVSTCRSAGVGVFA------------------DVVMNHLTGGPNGSS------------------------------------------VGTGFAGS---SFSK--YTYPL-
32_OAG04271_Paraphaeosphaeria_sporulosa     RG-FFLNMVKTCHSAGVRVII------------------DIVINQMTAGS-----------------------------------------------GTGTGGS---SYTK--YNYPG-
32_EGG02960_Melampsora_larici_populina      PQ-QFVEMVNRCNAAGVGVIV------------------DAVVNHMTSGRPE-G--------------------------------------------FGTSGS---SFSH--YSYDDI
32_EGG02993_Melampsora_larici_populina      PD-EFADMVKRCNAVGVGIIV------------------DTVMNHMTATNVT-A--------------------------------------------LGTANS---SYSR--YSYENL
32_OAV91411_Puccinia_triticina              EA-EFDAMVQRCTSAGVGVIV------------------DVVLNHMSAGSTT-E------------------------------------------YRRGSAGS---SYRK--YDYPNL
32_EFP79978_Puccinia_graminis               EA-EFDRMVQRCGGAGVGVIV------------------DVVLNHMSAGSTT-E------------------------------------------YRRGSSGS---SYRK--YEYPDL
32_GAT51088_Mycena_chlorophos               QA-EFESMVSTCNAAGVGVIV------------------DVVFNHMTGSS-AST------------------------------------------TSTGFAGS---SYSK--YNYPAV
32_KNE94052_Puccinia_striiformis            EA-EFDAMVQRCASAGVGVIV------------------DVVFNHMSAGSIT-E------------------------------------------ARRGSAGS---SYRK--YEYSDA
32_KNZ54553_Puccinia_sorghi                 EA-EFDEMIERCGRAGVGVIA------------------DVVLNHMSAGSTT-----EDRRG--------------------------------------SGGS---TYRK--YHYPN-
42_AOF40721_Microbacterium_aurum            PGATYQELINAAHAKGIKIYQ------------------DVVYNHTSRWGEK-NLFVPTVYG------KRDSQWSWYYDAPVEGKVYNPTQT--DADGSTYNGD---LWSE--TQPAGQ
42_CAB06816_Streptomyces_lividans           AGASYQDLIDAAHAKGMKIYQ------------------DVVYNHSSRWGAR-GLFTPTVYG------VRDSQWSWYYDEKQPGFEYDGLTVEPKSGKSYYNGD---LWST--AEPTGN
42_AWA81565_Pecoramyces_ruminatium          EGATYQDLINECHKKGIKVIQ------------------DIVLNHTSNNGER-GLFPMISRDYHLTDGVTGNYAEFYLDDPNHI--------LPDD------------YED--LNPSTQ
```

```
1_AAA85446_Paenibacillus_polymyxa             ------------N----GF---------AK--------APFDKADWYHHN---------------------------------------GDITD---G----DY--------------
1_CAA49465_Thermoactinomyces_vulgaris         -----------------SF---------AK--------APFDKADWYHHY---------------------------------------GDTQD------------------------
1_BAD06003_Aspergillus_awamori                ------------NDVDYSV---------FD---------PFDSSSYFHPY---------------------------------------CLITD---W---DNL--------------
1_BAD06002_Aspergillus_awamori                ------------SSVDYSV---------FK---------PFSSQDYFHPF---------------------------------------CFIQN---Y---EDQ--------------
1_AAF14264_Aspergillus_flavus                 ------------SSVDYSV---------FK---------PFSSQDYFHPF---------------------------------------CLIQN---Y---EDQ--------------
1_BAD01051_Aspergillus_kawachii               ------------SSVDYSV---------FK---------PFSSQDYFHPF---------------------------------------CFIQN---Y---EDQ--------------
1_BAA22993_Aspergillus_kawachii               ------------NDVDYSV---------FD---------PFDSSSYFHPY---------------------------------------CLITD---W---DNL--------------
1_EAA64850_Aspergillus_nidulans               ------------ASVDYSA---------FN---------PFNSADYFHTP---------------------------------------CDITD---Y---DNQ--------------
1_AAF17100_Aspergillus_nidulans               ------------GSTDFSI---------FT---------PFDSASYFHSY---------------------------------------CAINN---Y---DNQ--------------
1_P56271_Aspergillus_niger                    ------------NDVDYSV---------FD---------PFDSSSYFHPY---------------------------------------CLITD---W---DNL--------------
1_CAK44871_Aspergillus_niger                  ------------SSVDYSV---------FK---------PFSSQDYFHPF---------------------------------------CFIQN---Y---EDQ--------------
1_CAK40249_Aspergillus_niger                  ------------THIDYST---------LT---------PFNSSSYYHPY---------------------------------------CKITD---W---NNF--------------
1_CAK41088_Aspergillus_niger                  ------------TTIDYNV---------FP---------QFNGSSYFHPY---------------------------------------CLITN---W---NNY--------------
1_CAA31218_Aspergillus_oryzae                 ------------SSVDYSV---------FK---------PFSSQDYFHPF---------------------------------------CFIQN---Y---EDQ--------------
1_BAA01255_Aspergillus_shirousami             ------------SSVDYSV---------FK---------PFSSQDYFHPF---------------------------------------CFIQN---Y---EDQ--------------
1_AEB80431_Aspergillus_tubingensis            ------------NDVDYSV---------FD---------PFDSSSYFHPY---------------------------------------CLITD---W---DNL--------------
1_BAA12010_Cryptococcus_sp_S_2                ------------NSVNYGS---------FT---------PFNSESYFHPF---------------------------------------CEI-D---Y---NNR--------------
1_ABG48762_Fusicoccum_sp_BCC4124              ------------SSVDYSR---------FN---------PFNSQSYFHSY---------------------------------------CEITD---Y---NNQ--------------
1_AAO12212_Lipomyces_kononenkoae              ------------QDVDYSL---------YP--------APFNTSSAFHQP---------------------------------------CPI-D---F---SNQ--------------
1_AAC49622_Lipomyces_spencermartinsiae        ------------ADVDYSE---------YF---------PYSSQDYFHSF---------------------------------------CWITD---Y---SNQ--------------
1_AAN75021_Lipomyces_starkeyi                 ------------ADVDYSE---------YF---------PYSSQDYFHSF---------------------------------------CWITD---Y---SNQ--------------
1_AFD54462_Malbranchea_cinnamomea             ------------ASIDYSQ---------FN---------PFNSSDYYHPP---------------------------------------CEI-N---Y---DDQ--------------
1_ABF72529_Ophiostoma_floccosum               ------------SNVDYSV---------FD---------AFPSSSYFHSY---------------------------------------CEISN---Y---DDQ--------------
1_EPS26265_Penicillium_oxalicum               ------------NTVDYSV---------FK---------PFSSSSYFHPY---------------------------------------CLISD---Y---SNQ--------------
1_ABO42285_Phanerochaete_chrysosporium        ------------LTPDLST---------Y----------MFKDQSQYHPY---------------------------------------CPI-D---W---NNI--------------
1_BAW15173_Pholiota_microspora                ------------TTPDYSN---------Y----------FFKDASLYHQY---------------------------------------CPI-D---W---GNT--------------
1_BAF98616_Pichia_burtonii                    ------------PSIDYSI---------YT---------PFNEQKYFHDY---------------------------------------CLITD---Y---ENQ--------------
1_AGJ52081_Rhizomucor_pusillus                ------------N--GYSG---------Y----------TFGDASLYHPK---------------------------------------CTI-D---Y---NDQ--------------
1_ADL28123_Rhizopus_oryzae                    ------------SGGDYSG---------Y----------TFGQSSEYHRA---------------------------------------CDI-N---Y---NDQ--------------
1_ADD80242_Saccharomycopsis_fibuligera        ------------DSVDYSE---------YT---------PFNDQKYFHNY---------------------------------------CLISN---Y---DDQ--------------
1_ABS76467_Saitozyma_flava                    ------------NSVNYGS---------FT---------PFNSESYFHPF---------------------------------------CEI-D---Y---NNR--------------
1_CAB11471_Schizosaccharomyces_pombe          ------------RNIDYGI---------YR---------PFNQSSHYHPM---------------------------------------CPIE----Q---DKP--------------
1_CAB40006_Schizosaccharomyces_pombe          ------------ADADYSS---------LN---------PFNKESYFHPY---------------------------------------CLI-D---WDITDNE--------------
1_CAA34162_Schwanniomyces_occidentalis        ------------SSVDYSS---------FT---------PFNQQSYFHDY---------------------------------------CLITN---Y---NDQ--------------
1_CAA51912_Schwanniomyces_occidentalis        ------------SSVAYSN---------YN---------PFNQQSYFHDY---------------------------------------CLITN---Y---DDQ--------------
1_ACN82436_Sclerotinia_sclerotiorum           ------------TCVDYSI---------YT---------PFNSKSYYHPF---------------------------------------CLI-D---Y---NNA--------------
1_CAA03110_Thermomyces_lanuginosus            ------------SEVDYSI---------FN---------PLNSQDYFHPF---------------------------------------CLIED---Y---DNQ--------------
1_BAG69580_Trichoderma_viride                 ------------IT-DDSP---------S----------PLNQQSSYHTK---------------------------------------CDI-D---F---NNQ--------------
1_CAJ21046_Valsaria_rubricosa                 ------------IA-NLSP---------P----------PLNEQSSYHTQ---------------------------------------CDI-D---Y---SSQ--------------
5_AWX66236_Alicyclobacillus_sp_18711          ------------RGNTYSS---------FKWHSYY--------------F---------------------------------------DG-VD---WDQSRQLSGKIYQIQGTKAWD
5_AAQ01675_Alkalimonas_amylolytica            ------------RQAHYTQQNWGYLWHDFDWDTA---------------F---------------------------------------NG-SD------------NQ--LYPGKWWG
5_CAL14744_Anoxybacillus_flavithermus         ------------RGNTYSS---------FKWRWYH--------------F---------------------------------------DG-TD---WDESRKLN-RIYKFRGTGKAWD
5_AEW07376_Bacillus_acidicola                 ------------RGNTYSS---------FKWRWYH--------------F---------------------------------------DG-VD---WDESRKLS-RIYKFRGIGKAWD
5_AAA22191_Bacillus_amyloliquefaciens         ------------RGNTYSD---------FKWHWYH--------------F---------------------------------------DG-AD---WDESRKI-SRIFKFRGEGKAWD
5_ABY86223_Bacillus_cereus                    ------------RGDPYSN---------FKWKWYH--------------F---------------------------------------DG-TD---WDEGRKL-NRIYKFRGIGKAWD
5_CAD26699_Bacillus_halmapalus                ------------RGNTYSD---------FKWRWYH--------------F---------------------------------------DG-VD---WDQSRQFQNRIYKFRGDGKAWD
5_AAA22226_Bacillus_licheniformis             ------------RGSTYSD---------FKWHWYH--------------F---------------------------------------DG-TD---WDESRKL-NRIYKFQ--GKAWD
5_AEM05860_Bacillus_licheniformis             ------------RGSTYSD---------FKWYWYH--------------F---------------------------------------DG-TD---WDESRKL-NRIYKFQ--GKAWD
5_AAK00598_Bacillus_megaterium                ------------RGDSYSN---------FKWKWYH--------------F---------------------------------------DG-TD---WDEGRKL-NRIYKFRGIGKAWD
5_AGN35141_Bacillus_paralicheniformis         ------------RGSTYSD---------FKWYWYH--------------F---------------------------------------DG-TD---WDESRKL-NRIYKFQ--GKAWD
5_AAR68734_Bacillus_sp                        ------------RGNTHSN---------FKWRWYH--------------F---------------------------------------DG-TD---WDQSRQLQNKIYKFRGTGKAWD
5_AAA22231_Bacillus_sp_707                    ------------RGNTHSS---------FKWRWYH--------------F---------------------------------------DG-VD---WDQSRRLNNRIYKFRGHGKAWD
5_BAF03567_Bacillus_sp_JAMB_204               ------------RGNTYSS---------FKWNWYH--------------F---------------------------------------DG-TD---YDQSRNL-NRIYKFRGTGKAWD
5_CAC39917_Bacillus_sp_KSM_K38                ------------RNNAYSD---------FKWRWFH--------------F---------------------------------------NG-VD---WDQRYQE-NHIFRFA--NTNWN
5_AAB18785_Bacillus_sp_MK_716                 ------------RGNTYSS---------FKWRWYH--------------F---------------------------------------DG-VD---WDESRKLS-RIYKFRGIGKAWD
5_AAA63900_Bacillus_sp_TS_23                  ------------RGNTYSS---------FKWRWYH--------------F---------------------------------------DG-TD---WDESRKLN-RIYKFRSTGKAWD
5_ABW87262_Bacillus_sp_YX_YX1                 ------------RGNTYSD---------FKWHWYH--------------F---------------------------------------DG-AD---WDESRKI-SRIFKFRGEGKAWD
5_AAF00567_Cytophaga_sp                       ------------RGTTYSN---------FKWQWFH--------------F---------------------------------------DG-TD---WDQSRSL-SRIFKFRGTGKAWD
5_AAC74994_Escherichia_coli                   ------------RAGQYSQ---------FIWDFKC--------------F---------------------------------------SG-ID---HIENPDE-DGIFKIVNDYTGEG
5_AFZ41193_Exiguobacterium_sp_DAU5            ------------RNNTYSP---------FKWRWYH--------------F---------------------------------------DG-TD---WDQSRSL-SRIYKFKSTGKAWD
5_AAA22235_Geobacillus_stearothermophilus     ------------RGNTYSS---------FKWRWYH--------------F---------------------------------------DG-VD---WDESRKLS-RIYKFRGIGKAWD
5_ABX83871_Geobacillus_thermodenitrificans    ------------RGNTYSS---------FKWRWYH--------------F---------------------------------------DG-VD---WDESRKLS-RIYKFRGIGKAWD
5_AFC87833_Geobacillus_thermoleovorans        ------------RGNTYSS---------FKWRWYH--------------F---------------------------------------DG-VD---WDESRKLS-RIYKFRGIGKAWD
5_ACL70573_Halothermothrix_orenii             ------------RNGEYSN---------FTWNGQC--------------F---------------------------------------DG-TD---WDDYSKE-SGKY--LFDEKSWD
5_CAQ30277_Nostoc_sp_PCC_7119                 ------------RQGKYSN---------FEWHWWH--------------F---------------------------------------DA-VD---YNEYNSG-DRSTVYLLEGKNFD
5_AAA27110_Salmonella_typhimurium             ------------RAGQYSN---------FIWDYHC--------------F---------------------------------------SG-ID---HIENPDE-DGIFKIVNDYTGDG
5_BAA24178_Streptococcus_equinus              ------------RHKAYND---------FEWHWYH--------------F---------------------------------------TG-TD---YDVKTGK-NGIFQIQGDNKGWA
5_AAA97431_Streptococcus_equinus              ------------ATVPYND---------FECHWYH--------------F---------------------------------------TG-TD---YDVKTGK-TGIFQIQGDNKGWA
5_AAN59233_Streptococcus_mutans               ------------RQDNYSD---------FKWHWYH--------------F---------------------------------------TG-VD---YDALHNE-NGIYMILGDNKGWA
5_CCD30600_uncultured_bacterium               ------------RKNKYSD---------FQWNWQC--------------F---------------------------------------DG-VD---WDEKTKT-SGKF--LFEGKSWD
5_ALP73597_Vibrio_alginolyticus               ------------RNDKYSN---------FHWTWYH--------------F---------------------------------------DG-VD---WDDAGEE-KAIFKFKGEGKAWD
5_ABK62854_Histoplasma_capsulatum             ------------RGERYSE---------MKYHWYH--------------F---------------------------------------SG-VD---FDARNKN-KGIYKILGPDKNKD
5_ABS11196_Paracoccidioides_brasiliensis      ------------RGNQYSK---------MKYRSEH--------------F---------------------------------------TG-VD---YDALTGR-NGIFKILGPQ-NKD
5_BAW15172_Pholiota_microspora                ------------RGDKYST---------LRWNHEH--------------F---------------------------------------TA-VD---WDQETRT-KAIYRIAEQG-RKG
15_AAB60935_Aedes_aegypti                     -------P--------F-------------------------NRSHFHAD---------------------------------------CQIND---YN---NA--------------
15_AAB60934_Aedes_aegypti                     -------P--------Y-------------------------GANDFNPH---------------------------------------CEINN---YN---DP--------------
15_AAA03322_Aedes_atropalpus                  -------P--------F-------------------------GPNDFNPR---------------------------------------CDITN---YN---DK--------------
15_AAA03323_Anopheles_merus                   -------P--------F-------------------------GWPDFNPP---------------------------------------CAIND---WG---NA--------------
15_AAN77138_Anthonomus_grandis                -------S--------Y-------------------------SSENFHAT---------------------------------------CDI-N---YN---DA--------------
15_AAN77139_Anthonomus_grandis                -------P--------Y-------------------------TIEHFHPT---------------------------------------CGM-D---YN---SP--------------
15_AAO13755_Asterias_rubens                   -------P--------Y-------------------------SALDFNWPQGRC--------------------------PTS------GDID----YS---NI--------------
15_AAY23288_Blattella_germanica               -------P--------Y-------------------------GPEDFHTP---------------------------------------CAINN---YN---DA--------------
15_BAB72257_Callosobruchus_chinensis          -------P--------Y-------------------------GGADFHKS---------------------------------------CPINN---YN---DP--------------
15_AAA03350_Culex_tarsalis                    -------P--------F-------------------------SALDFNPR---------------------------------------CPLNN---YN---NA--------------
15_AAF20998_Diabrotica_virgifera_virgifera    -------P--------F-------------------------GPNHFHSR---------------------------------------CDINN---YQ---ES--------------
15_AAC35243_Drosophila_ananassae              -------P--------Y-------------------------SNPDFHPT---------------------------------------CAINN---YN---DA--------------
15_BAB91544_Drosophila_bocki                  -------P--------Y-------------------------SSLDFNPT---------------------------------------CAISN---YN---DA--------------
15_BAA04582_Drosophila_erecta                 -------P--------Y-------------------------SSLDFNPT---------------------------------------CGISN---YN---DA--------------
15_BAB91509_Drosophila_kikkawai               -------P--------Y-------------------------SSLDFNPT---------------------------------------CAISN---YN---DA--------------
15_BAA95437_Drosophila_leontia                -------P--------Y-------------------------SSLDFNPT---------------------------------------CAISN---YN---DA--------------
15_BAA95441_Drosophila_lini                   -------P--------Y-------------------------SSLDFNPT---------------------------------------CAINN---YN---DA--------------
15_BAA04583_Drosophila_mauritiana             -------P--------Y-------------------------SSLDFNPT---------------------------------------CAINN---YN---DA--------------
15_AAK93479_Drosophila_melanogaster_Amyrel    -------P--------Y-------------------------TAQDFHPT---------------------------------------CEITD---WN---DR--------------
15_CAA28238_Drosophila_melanogaster           -------P--------Y-------------------------SSLDFNPT---------------------------------------CAISN---YN---DA--------------
15_AAC46839_Drosophila_pseudoobscura          -------P--------Y-------------------------SSLDFNPT---------------------------------------CAISN---YN---DA--------------
15_BAA04585_Drosophila_sechellia              -------P--------Y-------------------------SSLDFNPT---------------------------------------CAINN---YN---DA--------------
15_AAC46463_Drosophila_virilis                -------P--------F-------------------------SSLDFNPT---------------------------------------CAITN---YA---DP--------------
15_AAG60011_Drosophila_yakuba                 -------P--------Y-------------------------SSLDFNPT---------------------------------------CSINN---YN---DA--------------
15_CAA76926_Phaedon_cochleariae               -------P--------Y-------------------------GSGDFHGR---------------------------------------CTSNN---YQ---DP--------------
15_P56634_Tenebrio_molitor                    -------P--------Y-------------------------GSGDFHSP---------------------------------------CEVNN---YQ---DA--------------
15_AAA03708_Tribolium_castaneum               -------P--------Y-------------------------GSGDFHDS---------------------------------------CTVNN---YQ---DA--------------
15_AAF73435_Zabrotes_subfasciatus             -------P--------Y-------------------------GPDDFHPD---------------------------------------CMINN---YQ---DV--------------
24_ABL09312_Acarus_siro                       -------L--------L-------------------------VPNDFHGRESCH--------------------------TDN------MDIKN---YD---NP--------------
24_BAB85635_Anguilla_japonica                 -------P--------Y-------------------------SSWDFNDG-KCK--------------------------TNS------GEIEN---YH---DI--------------
24_AAL37207_Crassostrea_gigas                 -------P--------F-------------------------SSWDFNGGTECS--------------------------TGD------GGIHN---YN---DP--------------
24_AAL37183_Crassostrea_gigas                 -------P--------F-------------------------SSWDFNSGSECS--------------------------TGD------GNIHN---YN---DP--------------
24_AAD38942_Dermatophagoides_pteronyssinus    -------P--------F-------------------------GPNDFHGHESCP--------------------------TQD------LEIHD---YT---NP--------------
24_AAD38943_Euroglyphus_maynei                -------P--------F-------------------------GPNDFHGHESCQ--------------------------TKD------LEIHN---YS---DR--------------
24_AAC60246_Gallus_gallus                     -------P--------Y-------------------------SAWDFNDG-KCH--------------------------TAS------GDIEN---YG---DM--------------
24_ABO26610_Haliotis_discus_discus            -------P--------Y-------------------------GPTDFNDNSNCH--------------------------TGD------MQIHN---YN---NP--------------
24_BAM74656_Haliotis_discus_hannai            -------P--------Y-------------------------GPTDFNDNSNCH--------------------------TGD------MQIHN---YN---NP--------------
24_AAA51724_Homo_sapiens_pancreatic           -------P--------Y-------------------------SGWDFNDG-KCK--------------------------TGS------GDIEN---YN---DA--------------
24_AAH63129_Homo_sapiens_salivary             -------P--------Y-------------------------SGWDFNDG-KCK--------------------------TGS------GDIEN---YN---DA--------------
24_AAA37221_Mus_musculus                      -------P--------Y-------------------------SGFDFNDG-KCR--------------------------TAS------GGIEN---YQ---DA--------------
24_AAA37230_Mus_musculus                      -------P--------Y-------------------------SAWDFNDN-KCN-----------------------------------GEIDN---YN---DA--------------
24_H2N0D4_Oryzias_latipes                     -------P--------Y-------------------------SNLDFNDG-KCK--------------------------TGS------GNIEN---YG---DP--------------
24_CAA68065_Pecten_maximus                    -------P--------F-------------------------SAWDFNTGNECH--------------------------SSD------MNIHD---YN---NA--------------
24_CAA54524_Penaeus_vannamei                  -------P--------Y-------------------------SAFDFNDG-NCH--------------------------TGS------GNIEN---YG---DA--------------
24_CAB65552_Penaeus_vannamei                  -------P--------Y-------------------------SGFDFNDA-NCH--------------------------TGS------GDIEN---YG---DA--------------
24_AAF65827_Pseudopleuronectes_americanus     -------P--------F-------------------------SNCDFNDN-KCR--------------------------TGS------GNIEN---YG---DA--------------
24_AAA40725_Rattus_norvegicus                 -------P--------Y-------------------------SAWYFNDN-KCN-----------------------------------GEINN---YN---DA--------------
24_AAH88228_Rattus_norvegicus                 -------P--------Y-------------------------SGFDFNDG-KCK--------------------------TGS------GGIEN---YN---DA--------------
24_P83053_Struthio_camelus                    -------P--------Y-------------------------SGWDFNDG-KCR--------------------------TGS------GEIEN---YG---DA--------------
24_AAF02828_Sus_scrofa                        -------P--------Y-------------------------SAWDFNDG-KCK--------------------------TAS------GGIES---YN---DP--------------
24_CAC87125_Tetraodon_nigroviridis            -------P--------Y-------------------------SYLDFNDY-KCK--------------------------TSS------GNIES---YDA--DV--------------
32_AAA21016_Aeromonas_hydrophila              -------Q--------F-------------------------GYNDFHHS---------------------------------------GDITN---YG---DS--------------
32_BAA22082_Bacillus_sp_195                   -------I--------Y-------------------------QSQDFHS---CR-----------------------------------RNIAN---YQ---DR--------------
32_AAY89038_Bifidobacterium_breve             -------P--------Y-------------------------TKENFHD---CT-----------------------------------KNIGD---YT---NA--------------
32_CAB92963_Halomonas_meridiana               -------H--------Y-------------------------QRDDFHEP---------------------------------------CGIEQSDYAQ---NA--------------
32_BAJ52728_Kocuria_varians                   -------T--------Y-------------------------SRADFHD---HG-----------------------------------CEVQD---YT---DR--------------
32_AKG25402_Microbacterium_aurum              -------L--------Y-------------------------SDSDFHS---CK-----------------------------------RDIDT---YK---DR--------------
32_CAA41481_Pseudoalteromonas_haloplanktis    -------I--------Y-------------------------SPQDFHES---------------------------------------CTINNSDYGN---DR--------------
32_AAA86835_Pseudomonas_sp_KFCC10818          -------P--------Y-------------------------GVNDFN-S---------------------------------------CTSD--INYS---NR--------------
32_AAA96317_Streptomyces_albus                -------L--------Y-------------------------SAPDFDD---CT-----------------------------------AEITD---YQ---DR--------------
```

```
32_BAC73693_Streptomyces_avermitilis  -------L--------Y------------------------SSYDMDD---CT----------------------------------ATITD---YT---NR---------------
32_CAA40798_Streptomyces_griseus  -------I--------W------------------------SGADMDD---CR----------------------------------SEIND---YG---NR---------------
32_AAA26698_Streptomyces_hygroscopicus  -------F--------Y------------------------QDQTFHG---CR----------------------------------KSISD---YT---NR---------------
32_AAA88554_Streptomyces_limosus  -------I--------W------------------------SGADMDD---CR----------------------------------SEIND---YG---NR---------------
32_CAB06622_Streptomyces_lividans  -------L--------Y------------------------SSNDLDN---CT----------------------------------SQINN---YG---DR---------------
32_CAA73775_Streptomyces_sp_TO1  -------L--------Y------------------------SSYDFDD---ST----------------------------------SQVTN---YQ---DR---------------
32_AAA82875_Streptomyces_sp_WL6  -------Y--------F------------------------GDADFHT---CR----------------------------------TAIKD---YT---DR---------------
32_AAA26697_Streptomyces_thermoviolaceus  -------L--------Y------------------------SAPDFDD---CT----------------------------------AEITD---YQ---DR---------------
32_AAB36561_Streptomyces_venezuelae  -------L--------Y------------------------SGSDMDD---CR----------------------------------ATISN---YQ---DR---------------
32_ABF13430_Thermobifida_fusca  -------I--------Y------------------------QSQDFND---CR----------------------------------RDITN---WN---DK---------------
32_CAA41881_Thermomonospora_curvata  -------I--------Y------------------------QSQDFND---CR----------------------------------RDITN---WN---DK---------------
32_BAV60949_Pholiota_microspora  ----------------Y------------------------QNQDFHH---CG--------------------------LEPG----DDIVN---YD---NA---------------
32_KJA26527_Hypholoma_sublateritium  ----------------Y------------------------QNQDFHH---CG--------------------------LEPN----DDIVN---YD---NA---------------
32_KDR78015_Galerina_marginata  ----------------Y------------------------QFTDFHH---CG--------------------------LEPD----DDIVN---YD---NR---------------
32_PPQ83160_Psilocybe_cyanescens  ----------------Y------------------------QNQDFHH---CG--------------------------LEPG----DDIVN---YS---NR---------------
32_KTB32361_Moniliophthora_roreri  ----------------Y------------------------QNQDFHH---CG--------------------------LESG----DEIVN---YG---NR---------------
32_KDQ30003_Pleurotus_ostreatus  ----------------Y------------------------QTQDFHH---CG--------------------------LEPG----DDIVN---YS---NR---------------
32_RDX41646_Polyporus_brumalis  ----------------Y------------------------QNQDFHH---CG--------------------------LEPG----DDIVN---YD---NA---------------
32_OJT13448_Trametes_pubescens  ----------------Y------------------------QNQDFHH---CG--------------------------LEPG----DDIVN---YD---NA---------------
32_PFH45132_Amanita_thiersii  ----------------Y------------------------QVQDFHH---CG--------------------------LEPG----DDIVN---YG---NR---------------
32_KIM37787_Hebeloma_cylindrosporum  ----------------Y------------------------QDQNFHH---CG--------------------------LRPG----DRIVD---YT---NR---------------
32_PBK61492_Armillaria_solidipes  ----------------Y------------------------QYQDFHH---CT--------------------------LTST----GDIED---YT---SR---------------
32_ETW76368_Heterobasidion_irregulare  ----------------Y------------------------QTQDFHH---CG--------------------------LEPG----DGIVN---YD---NR---------------
32_RDB27268_Hypsizygus_marmoreus  ----------------Y------------------------RGQDFHH---CG--------------------------LTSG----DDIVN---FN---DR---------------
32_PPQ71147_Gymnopilus_dilepis  ----------------Y------------------------DYDNFHH---CG--------------------------LEPD----DNIVN---YD---NR---------------
32_OSD07916_Trametes_coccinea  ----------------Y------------------------QNQDFHH---CG--------------------------LEPG----DDIVN---YD---NA---------------
32_KZW01389_Exidia_glandulosa  ----------------Y------------------------QNQDFHH---CG--------------------------LESG----DDIVN---YS---NR---------------
32_EIW55835_Trametes_versicolor  ----------------Y------------------------QNQDFHH---CG--------------------------LEPG----DDIVN---YD---NA---------------
32_SJL11930_Armillaria_ostoyae  ----------------Y------------------------QYQDFHH---CT--------------------------LTST----GDIED---YT---SR---------------
32_PAV18085_Pyrrhoderma_noxium  ----------------Y------------------------QYQDFHH---CG--------------------------LEPN----DDIVN---YD---NR---------------
32_RPD65356_Lentinus_tigrinus  ----------------Y------------------------ENQDFHH---CG--------------------------LEPG----DDIVN---YD---NA---------------
32_PBK84861_Armillaria_gallica  --------------------------------------------DFHH---CT--------------------------LTST----GDIED---YT---SR---------------
32_KLO18298_Schizopora_paradoxa  ----------------Y------------------------DNSSFHH---CG--------------------------LEPD----DTIVN---YD---NR---------------
32_PPR08271_Panaeolus_cyanescens  ----------------Y------------------------QNQDIHH---CG--------------------------LEPG----DDIVN---FS---NR---------------
32_KIK53845_Gymnopus_luxurians  ----------------Y------------------------QTQDFHM---CG--------------------------LEPG----NIIVN---YD---NE---------------
32_KIJ56431_Sphaerobolus_stellatus  ----------------Y------------------------QAQDFHY---CG--------------------------LEPG----NNIVN---YD---NA---------------
32_EJD07843_Fomitiporia_mediterranea  ----------------Y------------------------EYQDFHH---CG--------------------------LEPG----DDIVN---YD---NR---------------
32_EPQ53992_Gloeophyllum_trabeum  ----------------Y------------------------EYQDFHH---DC--------------------------GEAD----GNIDN---YD---NR---------------
32_KIP11288_Phlebiopsis_gigantea  ----------------Y------------------------ESQDFHY---CG--------------------------LEPD----NNIVN---YD---NA---------------
32_KIY62009_Cylindrobasidium_torrendii  ----------------Y------------------------QTQDFHH---CG--------------------------LTSG----DDIVD---YS---NR---------------
32_KIJ69360_Hydnomerulius_pinastri  ----------------P----------------------YTS-DNFHY---CG--------------------------LEPD----NNIVN---WD---NE---------------
32_KZT20455_Neolentinus_lepideus  ----------------Y------------------------QNADFHH---CG--------------------------GEPD----DNIVN---YD---NR---------------
32_EGN97351_Serpula_lacrymans_var_lacrymans  ----------------T----------------------YSA-DNFHY---CG--------------------------LEPG----NTIVN---YD---NR---------------
32_KDQ06010_Botryobasidium_botryosum  ----------------Y------------------------QTQDFHH---CG--------------------------LQSG----DEIVN---YK---NR---------------
32_PPR00074_Panaeolus_cyanescens  ----------------Y------------------------GSQDFHY---CG---------------------------SPG----NDINN---YF---DR---------------
32_EKM53319_Phanerochaete_carnosa  ----------------Y------------------------EYQDFHH---CG--------------------------LEPD----DNIVD---YS---NA---------------
32_TBU45601_Dichomitus_squalens  ----------------Y------------------------QNQDFHH---CG--------------------------LEPG----DDIVN---YD---NR---------------
32_PSR82077_Phlebia_centrifuga  ----------------Y------------------------QNQDFHH---CG--------------------------LEPG----DDIVN---YD---NR---------------
32_KIM89391_Piloderma_croceum  ----------------Y------------------------EAQDFHY---CG--------------------------LEPN----NDIVD---YE---NR---------------
32_PIL31175_Ganoderma_sinense  ----------------Y------------------------QNQDFHH---CG--------------------------LEPG----DDIVK---YD---SR---------------
32_KIY70325_Cylindrobasidium_torrendii  ----------------Y------------------------QTQDFHH---CG--------------------------LTSD----DDIED---YT---NL---------------
32_KDQ58060_Jaapia_argillacea  ----------------Y------------------------NSTNFHY---CG--------------------------LEPD----NNIVN---YD---NR---------------
32_CEL62905_Rhizoctonia_solani  ----------------Y------------------------QTQDFHH---CG--------------------------LQPG----DDIVN---YN---NR---------------
32_GAT57350_Mycena_chlorophos  ----------------Y------------------------NTTNFHH---CG--------------------------LEPG----DDIVN---YN---NR---------------
32_KIM25608_Serendipita_vermifera  ----------------Y------------------------GTQDFHH---CG--------------------------LTSG----DDIVD---YS---SR---------------
32_GAW02849_Lentinula_edodes  ----------------Y------------------------EAQDFHY---CG--------------------------LEPG----NTIVN---YD---NE---------------
32_TCD63303_Steccherinum_ochraceum  ----------------Y------------------------QSQDFHH---CG--------------------------LQPD----DNIVD---YS---NR---------------
32_KZP22139_Fibularhizoctonia_sp_CBS_109695  ----------------G----------------------Y-SATSFHY---CG--------------------------LEPN----NAIVN---YD---IR---------------
32_KII90441_Plicaturopsis_crispa  ----------------P----------------------YTDNGDFHH---CN--------------------------NGND----DNTQN---WD---NS---------------
32_KDQ58058_Jaapia_argillacea  ----------------Y------------------------DTTNFHH---CG--------------------------LEPG----DDIEN---YD---NR---------------
32_OCH93218_Obba_rivulosa  ----------------Y------------------------SDSDFHS---CR--------------------------T--------TITD---WD---NE---------------
32_EMD36390_Gelatoporia_subvermispora  ----------------Y------------------------SDSDFHS---CR--------------------------T--------SITS---WD---DE---------------
32_EIW78746_Coniophora_puteana  ----------------P----------------------YTS-DNFHY---CG--------------------------TT-N----NAIED---WN---NE---------------
32_KII86244_Plicaturopsis_crispa  ----------------Y------------------------QNQDFHH---QEANESEIADPMPPRTPTKHFFVIFPQWLESG----DDIVN---YD---NR---------------
32_EKM75205_Agaricus_bisporus  ----------------Y------------------------QIQDFHH---CG--------------------------FHPS----DDIEN---FS---NR---------------
32_KZT36083_Sistotremastrum_suecicum  ----------------Y------------------------ASTDFHT---NC--------------------------GTPD----NQIDD---YN---NA---------------
32_KZS90559_Sistotremastrum_niveocremeum  ----------------Y------------------------ASTDFHT---NC--------------------------GTPD----NQIDD---YN---NA---------------
32_EFI98134_Schizophyllum_commune  ----------------Y------------------------QTQDFHH---CG--------------------------LESG----DDIVN---YS---NR---------------
32_BBC62173_Tricholoma_matsutake  ----------------Y------------------------QTQDFHH---CG--------------------------LTPG----DQIVD---YS---SR---------------
32_CCA69081_Serendipita_indica  ----------------Y------------------------QYQDFHH---CT--------------------------LTST----GDIVD---WT---SR---------------
32_EFI98135_Schizophyllum_commune  ----------------Y------------------------QTQDFHH---CG--------------------------LEPY----DDINN---YG---NR---------------
32_KII85613_Plicaturopsis_crispa  ----------------Y------------------------NTTNFHH---CG--------------------------LEPN----DRIVN---YN---NR---------------
32_EIN11730_Punctularia_strigosozonata  ----------------P----------------------YTDDGDFHH---CG--------------------------LEPG----DDIVN---YD---NA---------------
32_PIL33242_Ganoderma_sinense  ----------------Y------------------------QNQDFHS---CR----------------------------------HGINN---WN---NA---------------
32_KZT18190_Neolentinus_lepideus  ----------------Y------------------------QYQDFHH---CG--------------------------LEPG----DNIRD---YR---NR---------------
32_KZV75308_Peniophora_sp_CONT  ----------------Y------------------------QAQDFHY---CG--------------------------LEPG----NAIVN---YN---SA---------------
32_KII86838_Plicaturopsis_crispa  ----------------Y------------------------NVSNFHH---CG--------------------------LETN----DNIVN---YN---NR---------------
32_KDQ08018_Botryobasidium_botryosum  ----------------Y------------------------QNDNFHH---CG--------------------------LEPG----DDIVN---YK---NR---------------
32_KIY73061_Cylindrobasidium_torrendii  ----------------Y------------------------TTDDFHH---CG--------------------------LTSG----DDIVD---YT---SR---------------
32_KZW01365_Exidia_glandulosa  ----------------Y------------------------THDSFHH---CG--------------------------LEPG----DNIVN---YD---SR---------------
32_KDQ58178_Jaapia_argillacea  ----------------Y------------------------SNSDFHH---CG--------------------------LEPG----DEIVD---YT---KR---------------
32_KZV75307_Peniophora_sp_CONT  ----------------Y------------------------QAQDFHY---CG--------------------------LEPN----NEIVN---YD---NA---------------
32_KIK51212_Gymnopus_luxurians  ----------------Y------------------------QSQDFHY---CG--------------------------LETD----NLLTN---WF---DE---------------
32_RDX41931_Polyporus_brumalis  ----------------Y------------------------QNQDFHN---CR----------------------------------HGINN---WN---DA---------------
32_TBU29254_Dichomitus_squalens  ----------------Y------------------------QDSDFHA---CR----------------------------------HGIDQ---WN---NA---------------
32_KIY45802_Fistulina_hepatica  ----------------Y------------------------SGFDFHY---CG--------------------------TEGD-----AIVD---WS---NA---------------
32_KIL59382_Amanita_muscaria  ----------------Y------------------------QYNDFHH---CG--------------------------VEPY----DDIKN---YT---NR---------------
32_KIY53725_Fistulina_hepatica  -------CLHAGCNGSY------------------------DSDNFHY---CG--------------------------TTDN-----DIED---WN---NV---------------
32_KIY47132_Fistulina_hepatica  ----------------Y------------------------GYQNFHH---CP--------------------------PVNG----SYAMD---FN---NR---------------
32_RPD55342_Lentinus_tigrinus  ----------------Y------------------------QNQDFHT---CR----------------------------------HGINN---WN---SA---------------
32_TCD61445_Steccherinum_ochraceum  ----------------W------------------------DTSNFHY---CG---------------------------TPG----NDIQD---WQ---SR---------------
32_KIM25602_Serendipita_vermifera  ----------------Y------------------------QTQDFHH---CG--------------------------LAPD----DNIND---YT---SR---------------
32_EJD37603_Auricularia_subglabra  ----------------Y------------------------TTDSFHH---CG--------------------------LAPG----DNIAD---YD---NR---------------
32_KZT54766_Calocera_cornea  ----------------Q-------------------------FNNFHH---CG--------------------------LEPN----DVIAN---YN---NR---------------
32_KIY46805_Fistulina_hepatica  ----------------Y------------------------TPDDFHH---CN--------------------------SPGG-----QIQD---YN---NE---------------
32_PPQ69654_Psilocybe_cyanescens  ----------------Y------------------------QYQDFHH---CG--------------------------LSNG----DDIVN---YS---NR---------------
32_KZO93971_Calocera_viscosa  ----------------Q-------------------------FDNFHH---CG--------------------------LEPN----DIIVN---YD---NA---------------
32_KZP07860_Fibularhizoctonia_sp_CBS_109695  -------P--------W------------------------VADNFHY---CG--------------------------DGNDGSGDSNWVDD--YT---NR---------------
32_PVG03687_Serendipita_vermifera  ----------------Y------------------------QVQDFHH---CG--------------------------LAPN----DNIQD---YS---NR---------------
32_EIM87674_Stereum_hirsutum  -------P--------Y------------------------TSSSFHY---CN--------------------------GANG---AKDISN---YD---NA---------------
32_CCA69087_Serendipita_indica  ----------------Y------------------------SANDFHY---CG--------------------------LTPD----NNIAD---YT---NR---------------
32_CEL62035_Rhizoctonia_solani  ----------------Y------------------------NWNDFHH---CG--------------------------LTNN----DDIQN---WG---DN---------------
32_EIN05398_Punctularia_strigosozonata  ----------------Y------------------------SGFDFHY---CG--------------------------T-TG----DAILD---WG---NK---------------
32_EJU02896_Dacryopinax_primogenitus  ----------------Q-------------------------YQDFHH---CG--------------------------ISSE---LIPLVVN---YD---NR---------------
32_KIO30302_Tulasnella_calospora  ----------------C----------------------L--LTD------L---------------------------EDSD----GDIHD---YT---NR---------------
32_CEL62036_Rhizoctonia_solani  ----------------Y------------------------GWNDFHH---CG--------------------------T-NN----DDIQN---WD---DR---------------
32_KIO24533_Tulasnella_calospora  ----------------Y------------------------DKNDFHY---CG--------------------------TP-G----NDIQN---WN---DR---------------
32_EIM84100_Stereum_hirsutum  ----------------Y------------------------SSYDFHY---CG--------------------------TD-G----DAILD---WS---NK---------------
32_KDN35973_Rhizoctonia_solani  ----------------Y------------------------EENDFHH---CG--------------------------NPPG----DDISD---YN---NR---------------
32_KIY48489_Fistulina_hepatica  -----------------------------------------NSTDFHY---CG--------------------------TT-D----DAILD---WS---NA---------------
32_KIY48486_Fistulina_hepatica  -----------------------------------------DSSDFHY---CG--------------------------TT-D----DAIED---WS---NA---------------
32_KZP26813_Fibularhizoctonia_sp_CBS_109695  -------P--------Y------------------------TQSDFHA---VC-----------------------------------SLDD---YN---NS---------------
32_KDQ32476_Pleurotus_ostreatus  ----------------Y------------------------SYQDFHHK--CG--------------------------SSDE-----VIHD---FR---SR---------------
32_EIM84087_Stereum_hirsutum  ----------------Y------------------------SPEHFHH---CG--------------------------TK-G----DGIED---YS---DK---------------
32_KDQ32475_Pleurotus_ostreatus  ----------------Y------------------------SYKDFHHN--CG--------------------------TPNN-----DILN---FT---NR---------------
32_EAQ88347_Chaetomium_globosum  -------L--------Y------------------------SASDFDD---CT----------------------------------SQISD---YG---DR---------------
32_ORY89754_Leucosporidium_creatinivorum  -------P--------F------------------------TSDDFHWP-V-------------------------------------CDID---WY---NA---------------
32_EWZ34052_Fusarium_oxysporum  -------L--------Y------------------------SFFDFDD---CT----------------------------------SGVRD---YS---DR---------------
32_OBT91518_Pseudogymnoascus_verrucosus  -------L--------Y------------------------SSYDFDD---CT----------------------------------AQVSN---YA---DR---------------
32_KEY73157_Stachybotrys_chartarum  -------L--------Y------------------------SSYDFDD---CT----------------------------------SQINN---YA---DR---------------
32_OBT62577_Pseudogymnoascus_sp_23342_1_I1  -------L--------Y------------------------SSSDFDD---CT----------------------------------SAVSN---YA---DR---------------
32_EON97874_Phaeoacremonium_minimum  -------L--------Y------------------------SSYDFDD---CT----------------------------------SDINN---YG---DR---------------
32_GAT55198_Mycena_chlorophos  ----------------Y------------------------HEENFHY---CD--------------------------GTVA----ATITD---FN---NL---------------
32_OAG04271_Paraphaeosphaeria_sporulosa  -------L--------Y------------------------SFYDFDD---CT----------------------------------SQITN---YV---DR---------------
32_EGG02960_Melampsora_larici_populina  ----------------Y------------------------DYHDFHH---CG--------------------------RNGN----DHISD---YT---DR---------------
32_EGG02993_Melampsora_larici_populina  ----------------Y------------------------NFSDFHH---CG--------------------------RNGN----DGVYN---FT---DR---------------
32_OAV91411_Puccinia_triticina  ----------------Y------------------------TRNNFHK---CR--------------------------KRGN----LEISN---YK---DR---------------
32_EFP79978_Puccinia_graminis  ----------------Y------------------------TRNNFHK---CR--------------------------KRGN----LEITN---YM---NR---------------
32_GAT51088_Mycena_chlorophos  -------P--------Y------------------------TASQFHY---CN--------------------------GANG---AASISD---YT---NA---------------
32_KNE94052_Puccinia_striiformis  ---------------------------------------------------SR--------------------------KRGN----LEISN---YM---DR---------------
32_KNZ54553_Puccinia_sorghi  ------------AASV-----------VAWRS--------------------------------------------------------P-TT---WTVTS-----------------
42_AOF40721_Microbacterium_aurum  NCANWGVPMGY-SAEGYRT--------YNCQWPNATSGMFP--KEIFHQ--------------------------------------CWIGN---W----EG---------------
42_CAB06816_Streptomyces_lividans  TCVNWGRPTGAKSPEGYTL--------YDCQWPSPTSGMFP--KALYHN--------------------------------------CWIGN---W----EG---------------
42_AWA81565_Pecoramyces_ruminatium  ----------------YHL--------R-------DSAMKD-EDYAYRK--------------------------------------DVTIS---W----ES---------------
```

```
                                             CSR-V                                   CSR-II                                                          CSR-III
1_AAA85446_Paenibacillus_polymyxa           -NSNNQWKIENGDVAGLDDLNHEN-PATANELKNWIKWLLNETGIDGLRLDTVKHVPKG-FL-----KDFDQA------------A-NTFTMGEIFHG--DP----AYVGDYTR--YLD
1_CAA49465_Thermoactinomyces_vulgaris       --WNDQWWVENGDIAGLDDLNQDN-PAVATELKNWIAWLVQTTGVDGLRVDTVKHVPKW-FW-----REFDGA------------A-NTFTLGEVFHG--DP----AYVGDYTN--YLD
1_BAD06003_Aspergillus_awamori              -TMVQDCWEGDTI-VSLPDLNTTE-TAVRTIWYDWVADLVSNYSVDGLRIDSVLEVEPD-FF-----PGYQEA------------A-GVYCVGEVDNG--NP----ALDCPYQD--YLD
1_BAD06002_Aspergillus_awamori              -TQVEDCWLGDNT-VSLPDLDTTK-DVVKNEWFDWVGSLVSNYSIDGLRIDTVKHVQKD-FW-----PGYNKA------------A-GVYCIGEVLDG--DP----AYTCPYQN--VMD
1_AAF14264_Aspergillus_flavus               -TQVEDCWLGDNT-VSLPDLDTTK-DVVKNEWYDWVGSLVSNYSIDGLRIDTVKHVQKD-FW-----PGYNKA------------A-GVYCIGEVLDG--DP----AYTCPYQN--VMD
1_BAD01051_Aspergillus_kawachii             -TQVEDCWLGDNT-VSLPDLDTTK-DVVKNEWYDWVGSLVSNYSIDGLRIDTVKHVQKD-FW-----PGYNKA------------A-GVYCIGEVLDG--DP----AYTCPYQN--VMD
1_BAA22993_Aspergillus_kawachii             -TMVQDCWEGDTI-VSLPDLNTTE-TAVRTIWYDWVADLVSNYSVDGLRIDSVEEVEPD-FF-----PGYQEA------------A-GVYCVGEVDNG--NP----ALDCPYQK--YLD
1_EAA64850_Aspergillus_nidulans             -TQVEDCWLYTDA-VSLPDVDTTN-EEVKEIWYDWVGDLVSDYSIDGLRIDTARHVQKD-FW-----RDYNDA------------A-GVYCVGEVFQG--DP----DYTCGYQE--VMD
1_AAF17100_Aspergillus_nidulans             -WQVENCFLGDDT-VSLTDLNTQS-SEVRDIWYDWIEDIVANYSVDGLRIDTVKHVEKD-FW-----PGYIDA------------A-GVYSVGEIFHG--DP----AYTCPYQD--YMD
1_P56271_Aspergillus_niger                  -TMVEDCWEGDTI-VSLPDLDTTE-TAVRTIWYDWVADLVSNYSVDGLRIDSVLEVQPD-FF-----PGYNKA------------S-GVYCVGEIDNG--NP----ASDCPYQK--VLD
1_CAK44871_Aspergillus_niger                -TQVEDCWLGDNT-VSLPDLDTTK-DVVKNEWYDWVGSLVSNYSIDGLRIDTVKHVQKD-FW-----PGYNKA------------A-GVYCIGEVLDG--DP----AYTCPYQN--VMD
1_CAK40249_Aspergillus_niger                -TNAQLCQTGDNI-VALPDLYTEH-AEVQETLSNWAKEVISTYSIDGLRIDAAKHVNPG-FL-----KNFGDA------------L-DIFMTGEVLQQ--EV----STICDYQNN-YIG
1_CAK41088_Aspergillus_niger                -TDAQWCQTGDNY-TALPDLYTEH-TAVQNILMDWSKSVISNYSVDGLRIDAAKSLTPS-FL-----PTYAST------------V-GGFMTGEVMDS--NA----TNVCKYQTD-YLP
1_CAA31218_Aspergillus_oryzae               -TQVEDCWLGDNT-VSLPDLDTTK-DVVKNEWYDWVGSLVSNYSIDGLRIDTVKHVQKD-FW-----PGYNKA------------A-GVYCIGEVLDG--DP----AYTCPYQN--VMD
1_BAA01255_Aspergillus_shirousami           -TQVEDCWLGDNT-VSLPDLDTTK-DVVKNEWYDWVGSLVSNYSIDGLRIDTVKHVQKD-FW-----PGYNKA------------A-GVYCIGEVLDV--DP----AYTCPYQN--VMD
1_AEB80431_Aspergillus_tubingensis          -TMVQDCWEGDTI-VSLPDLNTTE-TAVRTIWYDWVADLVSNYSVDGLRIDSVEEVEPD-FF-----PGYQEA------------A-GVYCVGEVDNG--NP----ALDCPYQK--YLD
1_BAA12010_Cryptococcus_sp_S_2              -TSILDCWEGDEI-VPLVDLRTED-SDVQSIFNSWISNLIQTYNIDGLRIDSLQQSGSF-FF-----PGFNQA------------AGGMYMVGEVFNG--SP----SYVCPYQQA-GMP
1_ABG48762_Fusicoccum_sp_BCC4124            -DLVEDCWLGDST-VELVDVKTED-STVANMYNTWISQLVSNYSIDGLRIDTAKHVDKS-FF-----PGFESA------------A-GVFCTGEVYDG--DP----SYTCDYQN--YMD
1_AAO12212_Lipomyces_kononenkoae            -SSIEDCWLVTEPAPALVDLKNED-QVILDALINSVVDLVETYDIDGIRLDTARHVPKP-SL-----AKFQEK------------V-GVFVTGEALNQ--SV----PYVAQYQGP--LN
1_AAC49622_Lipomyces_spencermartinsiae      -TNVEECWLGDDS-VPLVDVNTQL-DTVKSEYQSWVKQLIANYSIDGLRIDTVKHVQMD-FW-----APFQEA------------A-GIYTVGEVFDG--DP----SYTCPYQE--NLD
1_AAN75021_Lipomyces_starkeyi               -TNVEQCWLGDDT-VPLVDVNTQL-DTVKSEYQSWVQELIANYSIDGLRIDTVKHVQMD-FW-----APFQEA------------A-GIYAVGEVFDG--DP----SYTCPYQE--NLD
1_AFD54462_Malbranchea_cinnamomea           -TSVEQCWLYTGA-NALPDLKTED-PHVSQVHNDWIADLVSKYSIDGLRIDTTKHVDKP-AI-----GSFNDA------------A-GVYAVGEVYHG--DP----AYTCPYQD--WVD
1_ABF72529_Ophiostoma_floccosum             -SNVEDCWLGDTT-VSLPDLNTEL-TSVRSIWNSWVAGLVANYSIDGLRIDTVKHVETS-FW-----PGYNDA------------A-GVYCVGEVFDG--DP----AYTCAYQN--YMD
1_EPS26265_Penicillium_oxalicum             -TNVEDCWLGDTT-VSLPDLDTTL-SSVQTIWYNWVSDLVSNYSIDGLRIDTVKHVEKS-FW-----PGYQSA------------A-GVYCVGEVFSG--DP----AYTCPYQN--YLD
1_AB042285_Phanerochaete_chrysosporium      -TSEQNCWLGDTK-VPLPDLNTQN-PTVVSGYSTWIKSLVETYNIDGLRIDAAKHVDAE-FW-----PQFCGA------------A-GVYCIGEVFGD--DI----DFASTWQGP--LD
1_BAW15173_Pholiota_microspora              -TSEQNCWLGDTK-VPLPDVDTQN-PTVVSQYGTWIANLVKEYSIDGLRIDAAKHVNID-FW-----PSFCKS------------A-GVFCIGEVFGG-ADI----EPIAMYQGPQALD
1_BAF98616_Pichia_burtonii                  -ALVEECWETS----TLPDLKTED-NEVLSVFESWVKDFVSNYTIDGLRVDSAKHMDTS-FY-----QPFTEA------------A-GVFLLGEVFDG--DS----AYTCPYQD--YIA
1_AGJ52081_Rhizomucor_pusillus              -TSIEQCWVAD----ELPDIDTEN-SDNVAILNDIVSGWVGNYSFDGIRIDTVKHIRKD-FW-----TGYAEA------------A-GVFATGEVFNG--DP----AYVGPYQK--YLP
1_ADL28123_Rhizopus_oryzae                  -NSIEQCWIS-----GLPDINTED-SAIVSKLNSIVSGWVSDYGFDGLRIDTVKHVRKD-FW-----DGYVSA------------A-GVFATGEVLSG--DV----SYVSPYQQ--HVP
1_ADD80242_Saccharomycopsis_fibuligera      -AQVQSCWEGDSS-VALPDLRTED-SDVASVFNSWVKDFVGNYSIDGLRIDSAKHVDQG-FF-----PDFVSA------------S-GVYSVGEVFQG--DP----AYTCPYQN--YIP
1_ABS76467_Saitozyma_flava                  -TSILDCWEGDEI-VPLVDLRTED-SDVQSIFNSWISNLIQTYNIDGLRIDSLQQSGSF-FF-----PGFNQA------------AGGMYMVGEVFNG--NP----SYVCPYQQA-GMP
1_CAB11471_Schizosaccharomyces_pombe        -LSLEQCWIGTED-MTLPDIDTEN-PQIIETLYNFIHDQVKQFKIDGLRVDATKHVRRT-FW-----PGFCES------------A-GVYCQGEEWTG--QA----DLFCEWQE--YMD
1_CAB40006_Schizosaccharomyces_pombe        -TNVMDCWQD-SG-VLLADLDVES-SDVSSYLSDHFKSLISKYDFDGLRIDAVKMMNYT-FF-----PDFVDA------------T-GVYSVGEVFSY--DP----DTMCSYMS--VLP
1_CAA34162_Schwanniomyces_occidentalis      -TNVEDCWEGDTE-VSLPDLSTED-NEVIGVFQTWVSDFVQNYSIDGLRIDSAKHVDTA-SL-----TKFEDA------------S-GVYNLGEVYQG--DP----TYTCPYQN--YMK
1_CAA51912_Schwanniomyces_occidentalis      -TNVEDCWEGDNT-VSLPDLRTED-SDVSSIFNLWVAELVSNYSIDGLRIDSAKHVDES-FY-----PSFQSA------------A-GVYLLGEVYDG--DP----AYTCPYQN--YMS
1_ACN82436_Sclerotinia_sclerotiorum         -TSVVDCWEGDNI-VSLPDLRTED-SDVYTEWNSWISQLVANYSIDGLRVDSAQQTGKA-FF-----PSFQNS------------A-GVYVVGEIFNG--DP----AYVCPYQN--YMN
1_CAA03110_Thermomyces_lanuginosus          -EEVEQCWLADTP-TTLPDVDTTN-PQVRTFFNDWIKSLVANYSIDGLRVDTVKHVEKD-FW-----PDFNEA------------A-A-CTVGEVFNG--DP----AYTCPYQE--VLD
1_BAG69580_Trichoderma_viride               -TSVENCWLA-----GLPDVDTQD-PTIRSLYQDWVSNLVSTYGFDGVRIDTVRHVEQD-YW-----PGFVNA------------S-GVYCIGEVFNG--DP----DFMQPYQS--LMP
1_CAJ21046_Valsaria_rubricosa               -SSIETCWIS-----GLPDLDTTD-STIRSLFQTWVHGLVSNYSFDGLRVDTVKHVEKD-YW-----PGFVSA------------A-GTYAIGEVFSG--DT----SYVAGYQS--VMP
5_AWX66236_Alicyclobacillus_sp_18711        W--EVDSENGNYDYLMGADIDYDH-PDVQTEVKNWGKWFVNTLNLDGVRLDAVKHIKFD-YM-----SSWLSSVKSTT---G---KSNLFAVGEYWNT--SL----GALENYEN-----
5_AAQ01675_Alkalimonas_amylolytica          N-----TFH--FPYLMGEDVDYNR-FEVQQEMKAWGEWIINHVGFSGFRMDAIAHVDTD-FT-----RDWINHVQWAT---S---E-DVFFVAEAWVS--DI-------NGYLD-----
5_CAL14744_Anoxybacillus_flavithermus       W--EVDTENGNYDYLMYADLDMDH-PEVVSELKNWGKWYVTTTNIDGFRLDAVKHIKYS-FF-----PDWLSYVRTQT---Q---K-PLFAVGEFWSY--DI----SKLHNYIT-----
5_AEW07376_Bacillus_acidicola               W--EVDTENGNYDYLMYADLDMDH-PEVVTELKNWGKWYVNTTNIDGFRLDAVKHIKFS-SF-----PDWLSYVRSQT---G---K-PLFTVGEYWSY--DI----NKLHNYIT-----
5_AAA22191_Bacillus_amyloliquefaciens       W--EVSSENGNYDYLMYADVDYDH-PDVVAETKKWGIWYANELSLDGFRIDAAKHIKFS-FL-----RDWVQAVRQAT---G---K-EMFTVAEYWQN--NA----GKLENYLN-----
5_ABY86223_Bacillus_cereus                  W--EVSSENGNYDYLMYADLDFDH-PDVANEMKKWGTWYAKELNLDGFRLDAVKHIDHE-YL-----RDWVNHVRQQT---G---K-DMFTVAEYWQN--DI----QTLNNYLV-----
5_CAD26699_Bacillus_halmapalus              W--EVDSENGNYDYLMYADVDMDH-PEVVNELRRWGEWYTNTLNLDGFRIDAVKHIKYS-FT-----RDWLTHVRNAT---G---K-EMFAVAEFWKN--DL----GALENYLN-----
5_AAA22226_Bacillus_licheniformis           W--EVSNENGNYDYLMYADIDYDH-PDVAAEIKRWGTWYANELQLDGFRLDAVKHIKFS-FL-----RDWVNHVREKT---G---K-EMFTVAEYWQN--DL----GALENYLN-----
5_AEM05860_Bacillus_licheniformis           W--EVSNENGNYDYLMYADIDYDH-PDVTAEIKRWGTWYANELQLDGFRLDAVKHIKFS-FL-----RDWVNHVREKT---G---K-EMFTVAEYWQN--DL----GALENYLN-----
5_AAK00598_Bacillus_megaterium              W--EVSSENGNYDYLMYADLDFDH-PDVANEMKKWGTWYANELNLDGFRLDAVKHIDHE-YL-----RDWVNHVRQQT---G---K-EMFAVAEYWQN--DI----QTLNNYLA-----
5_AGN35141_Bacillus_paralicheniformis       W--EVSNENGNYDYLMYADIDYDH-PDVTAEIKRWGTWYANELQLDGFRLDAVKHIKFS-FL-----RDWVNHVREKT---G---K-EMFTVAEYWQN--DL----GALENYLN-----
5_AAR68734_Bacillus_sp                      W--EVDIENGNYDYLMYADIDMDH-PEVINELRNWGVWYTNTLNLDGFRIDAVKHIKYS-YT-----RDWLTHVRNTT---G---K-PMFAVAEFWKN--DL----AAIENYLN-----
5_AAA22231_Bacillus_sp_707                  W--EVDTENGNYDYLMYADIDMDH-PEVVNELRNWGVWYTNTLGLDGFRIDAVKHIKYS-FT-----RDWINHVRSAT---G---K-NMFAVAEFWKN--DL----GAIENYLQ-----
5_BAF03567_Bacillus_sp_JAMB_204             W--EVSSEYGNYDYLLYADIDYDH-PDVVNEMKKWGTWYANELKLDGFRIDAAKHIKHS-FL-----GDWVTSVRTST---G---K-EMFTVAEYWQN--NL----GALQNYLN-----
5_CAC39917_Bacillus_sp_KSM_K38              W--RVDEENGNYDYLLGSNIDFSH-PEVQDELKDWGSWFTDELDLDGYRLDAIKHIPFW-YT-----SDWVRHQRNEA---D---Q-DLFVVGEYWKD--DV----GALEFYLD-----
5_AAB18785_Bacillus_sp_MK_716               W--EVDTENGNYDYLMYADLDMDH-PEVVTELKNWGKWYVNTTNIDGFRLDAVKHIKFS-FF-----PDWLSYVRSQT---G---K-PLFTVGEYWSY--DI----NKLHNYIT-----
5_AAA63900_Bacillus_sp_TS_23                W--EVDTENGNYDYLMFADLDMDH-PEVVTELKNWGTWYVNTTNIDGFRLDAVKHIKYS-FF-----PDWLTYVRNQT---G---K-NLFAVGEFWSY--DV----NKLHNYIT-----
5_ABW87262_Bacillus_sp_YX_YX1               W--EVSSENGNYDYLMYADVDYDH-PDVVAETKKWGIWYANELSLDGFRIDAAKHIKFS-FL-----RDWVQAVRQAT---G---K-EMFTVAEYWQN--NA----GKLENYLN-----
5_AAF00567_Cytophaga_sp                     W--EVSSENGNYDYLMYADIDYDH-PDVVNEMKKWGVWYANEVGLDGYRLDAVKHIKFS-FL-----KDWVDNARAAT---G---K-EMFTVGEYWQN--DL----GALNNYLA-----
5_AAC74994_Escherichia_coli                 WNDQVDDELGNFDYLMGENIDFRN-HAVTEEIKYWARWVMEQTQCDGFRLDAVKHIPAW-FY-----KEWIEHVQEVA---P---K-PLFIVAEYWSH--EV----DKLQTYID-----
5_AFZ41193_Exiguobacterium_sp_DAU5          S--EVSGENGNYDYLMYADVDFEH-PEVRQEMKNWGKWYADSLGLDGFRLDAVKHINHS-YL-----KEWVTSVRQTT---G---K-EMFTVAEYWKN--DL----GAINDYLA-----
5_AAA22235_Geobacillus_stearothermophilus   W--EVDTENGNYDYLMYADLDMDH-PEVVTELKSWGKWYVNTTNIDGFRLDAVKHIKFS-FF-----PDWLSDVRSQT---G---K-PLFTVGEYWSY--DI----NKLHNYIM-----
5_ABX83871_Geobacillus_thermodenitrificans  F--HFAYFEKAYYDSTENGDIDDH-PEVVTELKSWGKWYVNTTNIDGFRLDAVKHIDFS-FF-----PDWLSDVRSQT---G---K-PLFTVGEYWSY--DI----NKLHNYIM-----
5_AFC87833_Geobacillus_thermoleovorans      W--EVDTENGNYDYLMYADLDMDH-PEVVTELKNWGKWYVNTTNIDGFRLDAVKHIKFS-FF-----PDWLSYVRSQT---G---K-PLFTVGEYWSY--DI----NKLHNYIT-----
5_ACL70573_Halothermothrix_orenii           W-----TYNWDEDYLMGADVDYEN-EAVQNDVIDWGQWIINNIDFDGFRLDAVKHIDYR-FI-----DKWMSAVQNSS---N---R-DVFFVGEAWVE--DV----DDLKGFLD-----
5_CAQ30277_Nostoc_sp_PCC_7119               D--YVALEKGNFAYLMGCDLDFQN-EWVRGEVTYWGKWCLDTTKVDGFRIDAIKHISTW-FF-----PEWIDALERHA---G---K-DLFMVGEYWYN--DI----NTLLWYVD-----
5_AAA27110_Salmonella_typhimurium           WNDQVDDEMGNFDYLMGENIDFRN-HAVTEEIKYWARWVMEQTHCDGFRLDAVKHIPAW-FY-----KEWIEHVQAVA---P---K-PLFIVAEYWSH--EV----DKLQTYID-----
5_BAA24178_Streptococcus_equinus            NQDLVDGENGNYDYLMYADLDFKH-PEVIKNIYDWAEWFVETTGVCGFRLDAIKHIDSF-FM-----SNFIRDMKEKY---G---K-DFYVFGEFWNG--DE----KTNNDYLA-----
5_AAA97431_Streptococcus_equinus            NQDLVDGENGNYDYLMYADLDFKH-PEVIKNIYDWADWFVETTGVKGFRLDAIKHIDSF-FM-----GNFIRDMKTKS---G---N-DFYVFGEFWNG--DE----KSNNDYLA-----
5_AAN59233_Streptococcus_mutans             SQENIDQENGNYDYLMYDDIDFKH-PEVQEHLRDWVAWFLETSGVGGFRLDAIKHIDKT-FM-----AQFIRYIREHL---K---A-DLYVFGEYWKD--SH----FDITDYLH-----
5_CCD30600_uncultured_bacterium             Y-----TFIRDDDYLMGADVDYEN-LEVQEDVTNWGNWIINEIGFDGFRLDAVKHIDPR-FI-----NQFINDVQKSS---S---K-DLFFVGEAWIE--DI----NTLAKFLD-----
5_ALP73597_Vibrio_alginolyticus             W--EVSSEKGNYDYLMYADLDMDH-PEVKQELKDWGEWYINMTGVDGFRMDAVKHIKYQ-YL-----QEWIDHLRWKT---G---K-ELFTVGEYWNY--DV----NQLHNFIT-----
5_ABK62854_Histoplasma_capsulatum           WARDVSKENGNYDYLMFSNLDYSN-PEVREDVFKWIEWMGHQLPLSGLRLDAAKHVSSG-FL-----KDFVAHVRRTV---G---P-GWLLVAEYWKA--EV----RELLEYLG-----
5_ABS11196_Paracoccidioides_brasiliensis    WARDVSTENGNYDYLMFADLDHSN-PEVREDIKRWIEWLGNQLHLSGLRLDAAKHCSAG-FL-----RDFIAHIQQTV---G---A-GWFFVAEYWKA--EV----WELLNYLG-----
5_BAW15172_Pholiota_microspora              WSRNVDTEHGNYDYLLGTDIDHRH-PAVQEDLLAWGKWILETTGATGFRLDAIKHIDRK-FM-----VRWIEDIRLAI---G---GKSMFVVAEYWSG--SY----KAIVPYMH-----
15_AAB60935_Aedes_aegypti                   -TNV-----RNCELAALPDLDQSN-RFVQNKIIQYLNHLL-DLGVAGFRMDACKHMRPE-DL-----KSIYDRLKPVNAMFLFPPGARPFIFQEVIDL-GT-EG--VSAKEYTN---LG
15_AAB60934_Aedes_aegypti                   -YQV-----RNCWLVGLPDLALGT-QWVRDRIVDLMNKCI-GYGIAGFRVDAVKHMWPG-DL-----EHIYNRLNNLNTDHGFPGGARPFITQEVIDL-GG-EA--ISKYEYTH---LG
15_AAA03322_Aedes_atropalpus                -YQV-----RNCWLVGLPDLALGN-QWVRDRIVDLMNKCV-GYGVAGFRVDAVKHMWPG-DL-----EHIYSRLNNLNTDHGFPHGAKPFITQEVIDL-GG-EA--ITKYEYTH---LG
15_AAA03323_Anopheles_merus                 -EQI-----RNCELVGLHDLNQAV-PWVRDRVVDFLNHLI-ELGVAGFRVDAAKHMWPA-DL-----AVIFGRLNNLNTAYGFAPGSRAFLAQEVIDM-GAHEV--VRKFEYTF---LG
15_AAN77138_Anthonomus_grandis              -ASI-----RNCELSGLKDLDQSQ-DYVRGKIIEYMNHLI-SLGVAGFRVDAAKHMWPA-DL-----SAIFGSVNDLNTDF-FPSGSRAMYYQEVIDT-GS-DP--VYNTEYTG---FG
15_AAN77139_Anthonomus_grandis              -SAI-----RNCELSGLPDLNQTE-DYVREKIKDYMNHLI-EIGVAGFRIDAAKHMWPE-DL-----KIIYGSLNDLNTDY-FSAGSRPFFYQEVIDY-GN-DV--VSKKEYID---FG
15_AAO13755_Asterias_rubens                 -DSI-----RNCDLVGLKDLNVEK-SYVQDKLAEYLNVLV-DAGVAGFRIDAAKHMWPG-DL-----DAVLKKVNNLNTAFGFPPNARPFIFQEVINQ-GG-EV--ISSWEYRH---LG
15_AAY23288_Blattella_germanica             -CNV-----RNCELSGLHDLNLSR-ESVQKKMVAYLDTLI-DAGVAGLRVDAAKHMWPS-NL-----QILYNKVKNLNTSHGFPNNARPFFFQEVIDQ-GG-EA--IHATEYTE---FG
15_BAB72257_Callosobruchus_chinensis        -AEV-----RNCELVGLPDLDQGR-QYVKDKIVEYMNHLV-DLGIAGFRVDAAKHIWPA-DL-----QAMYASVKNLNTEF-FPENSRPFYYQEVIDF-GG-DA--IKREEYLG---FG
15_AAA03350_Culex_tarsalis                  -IEV-----RNCWLVGLPDLNQGV-QWVRDKIVELFNKLI-GMGVAGFRVDAVKHMWPG-DL-----QAIYSRLDPLPTSHGFPPNAQAFLTQEVIDL-GG-EA--VTRDEYTH---LG
15_AAF20998_Diabrotica_virgifera_virgifera  -HNI-----RNCALTGLPDLDQSH-EHVRKKIVEYLNHLV-DLGVAGFRVDAAKHMWPA-DL-----KAIYDSVKDLTG---NGLSGRPFVYQEVIDL-GG-EA--VKKTEYTS---FG
15_AAC35243_Drosophila_ananassae            -AQV-----RNCELVGLHDLNQGN-SYVRDKVVLFLNHLI-DLGVAGFRVDAAKHMWPA-DL-----GVIYSRLKNLNPSHGFVQGSKAFIIQEVIDM-GG-EA--ISKFEYTS---LG
15_BAB91544_Drosophila_bocki                -NQV-----RNCELVGLRDLNQGN-SYVQEKIAEFLNHLI-DLGVAGFRVDAAKHMWPA-DL-----GVIYGRLKNLNTDHGFSSGSKAYIVQEVIDM-GG-EA--ISKSEYTG---LG
15_BAA04582_Drosophila_erecta               -NQV-----RNCELVGLRDLNQGN-SYVRDKVVEFLDHLI-DLGVAGFRVDAAKHMWPA-DL-----AVIYGRLKNLNTDHGFASGSKAYIVQEVIDM-GG-EA--ISKSEYTG---LG
15_BAB91509_Drosophila_kikkawai             -NQV-----RNCELVGLRDLNQGN-SYVQEKIAEFLNHLI-DLGVAGFRVDAAKHMWPA-DL-----GVIYGRLKNLNTDHGFSSGSKAYIVQEVIDM-GG-EA--ISKSEYTG---LG
15_BAA95437_Drosophila_leontia              -NQV-----RNCELVGLRDLNQGN-SYVQEKIAEFLNHLI-DLGVAGFRVDAAKHMWPA-DL-----GVIYGRLKHLNTDHGFSSGSKAYIVQEVIDM-GG-EA--ISKSEYTG---LG
15_BAA95441_Drosophila_lini                 -NQV-----RNCELVGLRDLNQGN-SYVQEKVAEFLNHLI-DLGVAGFRVDAAKHMWPA-DL-----GVIYGRLKNLNTDHGFSSGSKAYIVQEVIDM-GG-EA--ISKSEYTG---LG
15_BAA04583_Drosophila_mauritiana           -NQV-----RNCELVGLRDLNQGN-SYVQDKVVEFLDHLI-DLGVAGFRVDAAKHMWPA-DL-----AVIYGRLKNLNTDHGFASGSKAYIVQEVIDM-GG-EA--ISKSEYTG---LG
15_AAK93479_Drosophila_melanogaster_Amyrel  -FQV-----QQCELVGLKDLDQSS-DWVRSKLIEFLDHLI-ELGVAGFRVDAAKHMASE-DL-----EYIYSSLSNLNIDHGFPHNSRPFIFQEVIDH-GH-ET--VSRDEYKD---LG
15_CAA28238_Drosophila_melanogaster         -NEV-----RNCELVGLRDLNQGN-SYVQDKVVEFLDHLI-DLGVAGFRVDAAKHMWPA-DL-----AVIYGRLKNLNTDHGFASGSKAYIVQEVIDM-GG-EA--ISKSEYTG---LG
15_AAC46839_Drosophila_pseudoobscura        -NQV-----RNCELVGLRDLNQGN-SYVQEKVSDFLNHLI-DLGVAGFRVDAAKHMWPA-DL-----GVIYGRLKNLNTDHGFASGSRPYIVQEVIDM-GG-EA--ISKTEYTG---LG
15_BAA04585_Drosophila_sechellia            -NQV-----RNCELVGLRDLNQGN-SYVQDKVVEFLDHLI-DLGVAGFRVDAAKHMWPA-DL-----AVIYGRLKNLNTDHGFASGSKAYIVQEVIDM-GG-EA--ISKSEYTG---VG
15_AAC46463_Drosophila_virilis              -TNV-----RNCELVGLRDLNQGN-SWVRDKIVDFLNHLT-DLGVAGFRVDAAKHMWPG-DL-----EAIYGRLNNLNTNHGFSSGAKPYIFQEVIDL-GG-EA--ISKSEYTG---LG
15_AAG60011_Drosophila_yakuba               -NQV-----RNCELVGLRDLNQGN-SYVQDKVVEFLDHLI-DLGVAGFRVDAAKHMWPA-DL-----AVIYGRLKTLNTDHGFNSGSKAYIVQEVIDM-GG-EA--ISKSEYTG---LG
15_CAA76926_Phaedon_cochleariae             -NNI-----RNCWLSGLPDLDQSK-DYVRDKILEYLNHLV-DLGVAGFRVDAAKHMWPA-DL-----QVIYGRVKDLNTDHGFSQGSRPFFYQEVIDL-GG-EG--VSKNEYTG---FG
15_P56634_Tenebrio_molitor                  -DNV-----RNCELVGLRDLNQGS-DYVRGVLIDYMNHMI-DLGVAGFRVDAAKHMSPG-DL-----SVIFSGLKNLNTDYGFADGARPFIYQEVIDL-GG-EA--ISKNEYTG---FG
15_AAA03708_Tribolium_castaneum             -SNV-----RNCELVGLADLNQGS-DYVRSKIIEYMNHLV-DLGVAGFRVDAAKHMWPA-DL-----EAIYGSLKNLNTDHGFLLGQKPFIFQEVIDL-GG-EA--ISKHEYTG---FG
15_AAF73435_Zabrotes_subfasciatus           -NNV-----RNCQLVGLPDLDQSK-QYVRDKIVGYLNHLV-DLGIAGFRVDAAKHMWPA-DL-----SAIYGSVKNLNSAY-FPGGSRPLFYQEVIDY-GT-EP--IKKGEYTG---FG
24_ABL09312_Acarus_siro                     -EEA-----RNCRLSGLRDLKQSS-EYVRQKQADFLNHLI-DLGVAGSRSDASKHMWPG-DL-----EAIYGKLHNLNTAY-FPANSRPFIYHEVIYY-GG-DG--IKSSDYTK---LG
24_BAB85635_Anguilla_japonica               -FQV-----RDCRLVSLLDLNLQK-DYVRDKVAEYMNTLI-DMGVAGFRVDACKHMWPG-DL-----QAVSSRLHNLNTSW-FPSNSKPFIYQEVIDL-GG-EP--ITASDYTG---IG
24_AAL37207_Crassostrea_gigas               -NEV-----RNCRLLGMADLKLSK-DYVRDTVAGYLNHLI-SLGVAGFRVDAAKHMWPG-DL-----RAVFERLHDLNTAY-FTAGTKPFIYLEVIDL-GN-EP--IKAAEYTG---IA
24_AAL37183_Crassostrea_gigas               -NEV-----RNCRLVSLVDLKLSK-DYVRDSIAGYLNHLI-SLGVAGFRVDAAKHMWPG-DL-----RAVFGRLHDLNSAY-FPSGTKPFIFQEVIDM-GH-EA--ISAAEYTG---IA
24_AAD38942_Dermatophagoides_pteronyssinus  -KEA-----RNCRLSGLRDLKQQS-EYVRQKQVDFLNHLI-DIGVAGFRSDASTHQWPD-DL-----RSIYSRLHNLNKEF-FPENSQPFIYHETIYY-GG-NG--INSNEYTS---LG
24_AAD38943_Euroglyphus_maynei              -NEA-----RNCRLSGLRDLKQQS-EYVRKKQVDFLNHLI-DIGVAGFRSDASTHQWPD-DL-----RSIYSQLHTLNKEF-FPENSHPFIYHETIYY-GG-NG--INSNEYTS---LG
24_AAC60246_Gallus_gallus                   -YQV-----RDCKLSSLLDLALEK-DYVRSTIAAYMNHLI-DMGVAGFRIDAAKHMWPG-DI-----RAFLDKLHDLNTQW-FSAGTKPFIYQEVIDL-GG-EP--ITGSQYFG---NG
24_AB026610_Haliotis_discus_discus          -EEV-----RNCRLVGLADLKAGK-DYVRSEIEGYLNHLV-DIGIAGFRVDAAKHMWPG-DL-----TAIFGSVKNLRSDV-FGGGKKPFVFQEVIDM-GG-EP--IKGDQYLG---SG
24_BAM74656_Haliotis_discus_hannai          -EEV-----RNCRLVGLADLKAGK-DYVRSEIEGYLNHLV-DIGIAGFRVDAAKHMWPG-DL-----TAIFGSVKNLRSDV-FGGGKKPFVFQEVIDM-GG-EP--IKGDQYLG---SG
24_AAA51724_Homo_sapiens_pancreatic         -TQV-----RDCRLTGLLDLALEK-DYVRSKIAEYMNHLI-DIGVAGFRLDASKHMWPG-DI-----KAILDKLHNLNSNW-FPAGSKPFIYQEVIDL-GG-EP--IKSSDYFG---NG
24_AAH63129_Homo_sapiens_salivary           -TQV-----RDCRLSGLLDLALGK-DYVRSKIAEYMNHLI-DIGVAGFRIDASKHMWPG-DI-----KAILDKLHNLNSNW-FPEGSKPFIYQEVIDL-GG-EP--IKSSDYFG---NG
24_AAA37221_Mus_musculus                    -AQV-----RDCRLSGLLDLALEK-DYVRTKVADYMNHLI-DIGVAGFRLDASKHMWPG-DI-----KAILDKLHNLNTKW-FSQGSRPFIFQEVIDL-GG-EA--VSSNEYFG---NG
24_AAA37230_Mus_musculus                    -YQV-----RNCRLTGLLDLALEK-DYVRTKVADYMNHLI-DIGVAGFRLDAAKHMWPR-DI-----KAVLDKLHNLNTKW-FSQGSRPFIFQEVIDL-GG-EA--IKGSEYFG---NG
24_H2N0D4_Oryzias_latipes                   -YQV-----RDCRLVGLLDLALEK-DYVRGKVADFMNKLI-DMGVAGFRVDACKHMWPG-DL-----DNVYRRLNNLNTKW-FPGGSRPFIFQEVIDL-GG-EP--ITTGEYVG---LG
24_CAA68065_Pecten_maximus                  -EEI-----RNCRLVSLADLKLSK-NYVREEITQYMNHLI-DLGVAGFRIDAAKHMWPG-DL-----RAMFGTLHDLNSAV-FGSGRKPFIFQEVIDM-GG-EP--ISASEYTG---IG
24_CAA54524_Penaeus_vannamei                -NQV-----RNCKLVGLNDLNQGT-DYVRGKIREFMNKLI-SYGVAGFRIDASKHMWPG-DM-----KAIFDSLDNLNTDF-FKAGARPFIFQEVIDL-GG-EA--ISSGEYVG---NG
24_CAB65552_Penaeus_vannamei                -NQV-----RNCKVVGMNDLNQGT-DYVRGKIRDYLNTLI-SFGVAGFRVDATKHMWPG-DM-----KAIFDSLDDLSPDI-YGVGARPFIVQEVIDL-GG-EA--ISSEEYVG---NG
24_AAF65827_Pseudopleuronectes_americanus   -NQV-----RDCRLVSLLDLALEK-DYVRGKVTDFLNVLI-DMGVAGFRVDACKHMWPG-DL-----AAVYGGLHNLNTKW-WSAGSRPFIFQEVIDL-GG-ES--ITTKEYVN---LG
24_AAA40725_Rattus_norvegicus               -NQV-----RNCRLSGLLDLALDK-DYVRTKVADYMNNLI-DIGVAGFRLDAAKHMWPG-DI-----KAVLDKLHNLNTKW-FSQGSRPFIFQEVIDL-GG-EA--IKGSEYFG---NG
24_AAH88228_Rattus_norvegicus               -AQV-----RDCRLSGLLDLALEK-DYVRTKVADYMNHLI-DIGVAGFRLDASKHMWPG-DI-----KAVLDKLHNLNTKW-FSEGSKPFIYQEVIDL-GG-EA--VSSNEYFG---NG
24_P83053_Struthio_camelus                  -SQV-----RDCRLVGLLDLALEK-DYVRSTVAGYMNHLI-DIGVAGFRLDAAKHMWPG-DI-----KAFLDKLHNLNTNW-FSSGSRPFIYQEVIDL-GG-EP--ITSSQYFG---NH
24_AAF02828_Sus_scrofa                      -YQV-----RDCQLVGLLDLALEK-DYVRSMIADYLNKLI-DIGVAGFRIDASKHMWPG-DI-----KAVLDKLHNLNTNW-FPAGSRPFIFQEVIDL-GG-EA--IQSSEYFG---NG
24_CAC87125_Tetraodon_nigroviridis          -NQV-----RDCRLENLLDLALEK-DYVRGKVADYMNKLV-SMGVAGFRVDACKHMWPG-DL-----SAVYGRLNNLNTKW-FPGGSRPFIFQEVIDL-GG-GV--ISSREYFY---LG
32_AAA21016_Aeromonas_hydrophila            -NNV-----WNGALYGLPDLNTGS-SYVQDQIATYMKTLL-GWGVAGFRIDAAKHMAPA-DV-----KAILDKA-----------GSPRAYLEVIGA-GG-ESPDIQPGRYTY---ID
32_BAA22082_Bacillus_sp_195                 -WEV-----QECNLVNLADLNTSS-SYVQGKIAAYLNDLV-SLGVDGLRIDAVKHIAAS-DM-----QGILSKVNDRA---------RLYIVQEVIR-A-NE---PIQPEEYTS---NG
32_AAY89038_Bifidobacterium_breve           -DEV-----QNCRLTSLQDLDTSQ-EYVQDKLADYMNRLL-DLGVYGFRVDAVKHIATA-DV-----AAIKAKLAEKS-GR---NADDIFFEQEVIG-NASE-AAEIQPSNYVA---NG
32_CAB92963_Halomonas_meridiana             -ESV-----RQCQLVGLPDLNTSD-PTVQSRIADYLDTLA-ALGVGGIRIDAAKHMAPS-DI-----AEILAQVD-----------APLYAFQEVIDL-GG-EA--ISATEYQG---TA
32_BAJ52728_Kocuria_varians                 -WEV-----QECDLVGLADLDTGA-DYVQSRLADYLDDLS-SLGVDGFRIDAAKHIAAA-DL-----EGILSRTDGIA---------AEDIVQEVIR-GAGE---PIQPEEYTD---LG
32_AKG25402_Microbacterium_aurum             -WVV-----QECNLVNLSDLNTGS-SSVQQKIADYLNKLI-DIGVKGFRFDAVKHIASA-DM-----NGIMSKVKNRD---------QLYIVQEVIR-AAGE---PIQPEEYTA---YG
32_CAA41481_Pseudoalteromonas_haloplanktis  -YRV-----QNCELVGLADLDTAS-NYVQNTIAAYINDLQ-AIGVKGFRFDASKHVAAS-DI-----QSLMAKVN-----------GSPVVFQEVIDQ-GG-EA--VGASEYLS---TG
32_AAA86835_Pseudomonas_sp_KFCC10818        -WSV-----QNCDLVGLNDLKTSS-EYVRQKIADYMNDAI-SMGVAGFRIDAAKHMPAE-DI-----AAIKSKLH-----------GTPYIFQEVIGA-EN-EP--VKPAEYTY---IG
32_AAA96317_Streptomyces_albus              -WNV-----QHCELVGLADLDTGE-EYVRQTIAGYMNDLL-SLGVDGFRIDAAKHIPAE-DL-----ANIKSRLSNP---------NAYWKQEVIY-GAGE---AVQPGEYTG---TG
```

```
                                                  CSR-V                        CSR-II                                       CSR-III
32_BAC73693_Streptomyces_avermitilis        -ANV-----QNCELVGLADLDTGE-EYVRKTIAGYMNTLL-GYGADGFRVDAVKHIPAA-DL-----ANIKSRLTNP----------SVYWKQEVIY-ASGE---AVQPTEYTG---NG
32_CAA40798_Streptomyces_griseus            -ANV-----QNCELVGLADLDTGE-PYVRDRIAAYLNDLL-LLGVDGFRIDAAKHMPAA-DL-----TAIKAKVGNG----------STYWKQEAIH-GAGE---AVQPSEYLG---TG
32_AAA26698_Streptomyces_hygroscopicus      -DDV-----QTCELVDLADLGTGS-DYVRTTIAGYL-GLR-SLGVDGFRIDAAKHISAT-DL-----AAVKGKMKDP-----------GYWVQEVIY-GAGE---AVRPDEYTG---IG
32_AAA88554_Streptomyces_limosus            -ANV-----QNCELVGLADLDTGE-SYVRDRIAAYLNDLL-SLGVDGFRIDAAKHMPAA-DL-----TAIKAKVGNG----------STYWKQEAIH-GAGE---AVQPSEYLG---TG
32_CAB06622_Streptomyces_lividans           -FNV-----QECELVGLADLDTGE-AYVRGKIAGYLTDLL-SLGVDGFRIDAAKHMAAA-DL-----AAIKSRLSNP----------NVYWKHEAIY-GAGE---AVSPTEYVG---SG
32_CAA73775_Streptomyces_sp_TO1             -WNV-----QHCELVGLADLDTGE-SYVRGGIARYMNDLL-SLGVDGFRIDAAKHIDAA-DL-----ANIKSRLTNP----------SVYWKQEVIY-GAGE---AVQPTEYTG---NG
32_AAA82875_Streptomyces_sp_WL6             -GDV-----QNCELVGLSDLDTGK-DEVRSTIAAYLDGLR-SMGVDGFRIDAAKHMAAD-DV-----AIQGQDERPP-----------GSGSPEVIQ-GGGE---AVQPEEYTE---IG
32_AAA26697_Streptomyces_thermoviolaceus    -WNV-----QHCELVGLADLDTGE-EYVRQTIAGYMNDLL-SLGVDGFRIDAATHIPAE-DL-----ANIKSRLSNP----------NAYWKQEVIY-GAGE---PPKPGEYTG---TG
32_AAB36561_Streptomyces_venezuelae         -ANV-----QNCELVQLPDLDTGE-DHVRGKIAGYLNDLA-SLGVDGFRIDAAKHMPAA-DL-----ANIKSRLTNP----------NVFWKLEAIH-GAGE---AVSPSEYLG---SG
32_ABF13430_Thermobifida_fusca              -WEV-----QHCELVGLADLKTSS-PYVQDRIAAYLNELI-DLGVAGFRIDAAKHIPEG-DL-----QAILSRLKNVHPAW---GGGKPYIFQEVIA---DS---TISTGSYTH---LG
32_CAA41881_Thermomonospora_curvata         -WEV-----QHCELVGLADLKTSS-PYVQDRIAAYLNELI-DLGVAGFRIDAAKHIPEG-DL-----QAILSRLKNVHPAW---GGGKPYIFQEVIA---DS---TISTGSYTH---LG
32_BAV60949_Pholiota_microspora             -VEV-----QTCELTNLADLATET-EYVRGRLAAYGNDLL-SLGVDGFRLDAAKHINTS-DI-----ANITSRLTT-----------KPYITQEVIFG-AGE-P--ITPAQYVG---NG
32_KJA26527_Hypholoma_sublateritium         -VEV-----FTCQLDNLADLFTET-EYVRSTLAAYGNDLL-SLGVDGFRLDAAKNINSS-DI-----ANITSRLTS-----------APYITQEVIFG-AGQ-P--VTPAMYVG---NG
32_KDR78015_Galerina_marginata              -LEV-----QTCELDNLADLATDT-EYVRARLAQFGSDLL-SLGADGFRLDAAKHIAAT-DL-----ANITSRLSG-----------GLYITQEVIWG-SGE-P--IQPSEYVG---IG
32_PPQ83160_Psilocybe_cyanescens            -LEV-----QTCELDNLADLATDT-EYVRSRLAAYGNDLL-SLGVDGLRLDAAKHIAAT-DL-----ANIISRLST-----------TPYITQEVIWG-SGE-P--IQPSEYTG---IG
32_KTB32361_Moniliophthora_roreri           -VEV-----QTCELVNLADLATDT-EYVRGRLAAYINDLL-SLGVDGLRLDAAKHIASS-DL-----ANILSRLNS-----------KPYITQEVIFG-NGE-P--IQPSEYVG---NG
32_KDQ30003_Pleurotus_ostreatus             -LEV-----QTCELVNLADLATDT-EYVRGRLAAYANDLR-SLGVDGFRLDAVKHIASG-DL-----ANILSRVTG-----------PFYVTQEVIFG-AGE-A--VQPSEYVD---IG
32_RDX41646_Polyporus_brumalis              -VEV-----QTCELVNLADLATDT-EYVRGRLAEYGNDLL-SLGADGLRLDASKHIPVG-DL-----ANILSRLNS-----------KPYITQEVIYG-AGE-P--ITPNQYVG---NG
32_OJT13448_Trametes_pubescens              -VEV-----QTCELVNLADLATDT-EYVRGRLAQYGNDLL-SLGADGLRLDASKHIPVG-DI-----ANILSRLNR-----------TVYITQEVIFG-SGE-P--ITPNQYTG---NG
32_PFH45132_Amanita_thiersii                -VEV-----QTCELVNLADLATDT-EYVRGRLAQYGNDLL-SLGVDGFRLDAAKHMPAG-DI-----SNILGRLSR-----------RPYITQEVIFG-AGE-P--IQPSEYVG---NG
32_KIM37787_Hebeloma_cylindrosporum         -VEV-----QTCQLGTLADLATET-EYVRARLAQYGNDLL-SLGVDGFRLDAAKHIAAT-DL-----SDIVSRLSR-----------KSYITQEIIWG-FGE-A--IQPSEYLG---IG
32_PBK61492_Armillaria_solidipes            -AQV-----QTCQLEGLADLATET-EYVRGRLAEYANDLL-SLGVDGFRLDAAKHIAAD-DI-----ANITSRFSS-----------SPYITQEVIWG-AGE-A--VQPSEYVG---IG
32_ETW76368_Heterobasidion_irregulare       -LEV-----QTCQLDNLADLATDT-EYVRERLAQYANDLL-SLGVDGLRLDAAKHIAAT-DI-----ANITSRFNS-----------APYITQEVIWG-SGE-P--IQPSEYTG---IG
32_RDB27268_Hypsizygus_marmoreus            -VQV-----QTCELVNLADLATDT-EYVRGRLAQYANDLI-SLGVDGLRLDAAKHMSAG-DI-----SNILGRLSA-----------RPYITQEVVWG-SGG-Q--VQPSEYTA---NG
32_PPQ71147_Gymnopilus_dilepis              -LEV-----QTCQLDNLADLGTDT-EFVRSRLAQYANDLL-SLGADGLRLDACKHMAAT-DV-----ANMTGRLSK-----------APYITQEVIWG-AGE-P--IQPSEYVG---IG
32_OSD07916_Trametes_coccinea               -VEV-----QTCELDNLADLATET-EYVRSRLAEYANDLL-SLGVDGLRLDAAKHINVT-DI-----ANILSRLDR-----------TVYITQEVIYG-AGE-P--ITPNQYTG---NG
32_KZW01389_Exidia_glandulosa               -VEV-----QTCELVNLADLATDT-DYVRGRLATYGNDLL-SLGADGFRLDAAKHIPAG-DI-----SNILGRLTT-----------RPYISQEVIWG-SGE-P--IQPSEYVG---NG
32_EIW55835_Trametes_versicolor             -VEV-----QTCELVNLADLATDT-EYVRGRLAQYGNDLL-SLGADGLRLDASKHIPVG-DI-----ANILSRLNR-----------TVYITQEVIFG-SGE-P--ITPNQYTG---NG
32_SJL11930_Armillaria_ostoyae              -AQV-----QTCQLEGLADLATET-EYVRGRLAEYANDLL-SLGVDGFRLDAAKHIAAD-DI-----ANITSRFSS-----------SPYITQEVIWG-AGE-A--VQPSEYVG---IG
32_PAV18085_Pyrrhoderma_noxium              -LEV-----QTCQLVGLADLATDT-EYVRSRLAEYGNDLL-SLGVDGMRLDAAKHIAAT-DL-----ANITSRLNG-----------APYLTQEVIYG-AGE-P--ITPAEYVG---IG
32_RPD65356_Lentinus_tigrinus               -VEV-----QTCELVNLADLATDT-EYVRGRLAEYGNDLL-SLGADGLRLDASKHIPVG-DI-----ANILSRLNS-----------TPYITQEVIYG-AGE-P--ITPNQYVG---NG
32_PBK84861_Armillaria_gallica              -AQV-----QTCQLEGLADLATET-EYVRGRLAEYANDLL-SLGVDGFRLDAAKHIAAD-DL-----ANITSRFSS-----------SPYITQEVIWG-AGE-A--VQPSEYVG---IG
32_KLO18298_Schizopora_paradoxa             -LEV-----QTCQLDGLADLATDT-EPIRERLAAYANDLI-SLGVSGLRLDAAKHMAAT-DV-----ANMTSRLVG-----------TPYVTQEVIWG-AGE-P--IQPSEYIG---IG
32_PPR08271_Panaeolus_cyanescens            -EEV-----QTCELENLADLATET-EYVRGRLAEYGNDLL-SLGVDGFRLDAAKHIASS-DI-----ANILSRLNG-----------HPYITQEVING-DDD-V--VLASEYVP---NG
32_KIK53845_Gymnopus_luxurians              -QEV-----WTCELDGLADLATDT-TYVRGRLAEYANDLM-SLGVDGLRLDAAKSIPPT-DI-----EAIVAQFTK-----------SPYLTQEVVWG-AGQ-P--VTPELYTG---IG
32_KIJ56431_Sphaerobolus_stellatus          -VEV-----QTCQLDNLADLATET-EYVRGRLAEYGNDLL-SLGVDGFRLDSAKHINTT-DI-----ANILSRLNS---------TSKPYITQEVIYG-AGE-P--ITPNQYTG---NG
32_EJD07843_Fomitiporia_mediterranea        -VEV-----QTCELVNLADLATET-EYVRSRLAEYGNDLL-SLGVDGLRLDAAKHIAAT-DL-----ANITSRLNR-----------PAYLTQEVIYG-EGE-P--ITPAEYVG---IG
32_EPQ53992_Gloeophyllum_trabeum            -TEV-----QVCQLDGLADLDTES-EDVRSTLAAYGNDLI-GLGVDGLRLDAAKHIAAT-SL-----ANITSRLTG-----------SPYITSETIWG-SGE-P--IQPSEYVG---IG
32_KIP11288_Phlebiopsis_gigantea            -VEV-----QTCQLDGLADLYTET-EYVRSRLAEYANDLL-SLGVDGLRLDAAKHIAAT-DI-----ANITSRLTK-----------APYITQEVIYG-AGE-P--ITPNQYTS---IG
32_KIY62009_Cylindrobasidium_torrendii      -AQI-----QTCELVNLADLATDT-EYVRARLATYVNDLV-SLGVDGLRLDAAKHISPD-DI-----ANILGRVSS-----------PGYITQEVIYD-SSG-PSGTLPADYVR---NG
32_KIJ69360_Hydnomerulius_pinastri          -VEV-----WTCQLDGLADLATDTE-YVQQMEAAYANDLM-SLGVKGLRLDAAKDINPA-DL-----ANITSRFNS-----------KPYITQEVIWG-SGQ-P--VTPALYTG---IG
32_KZT20455_Neolentinus_lepideus            -TEV-----QECQLEGLADLDTEA-EYVRSTLAAYGNHLL-GLGVDGLRLDAAKHIAAT-SL-----ANITSRLTG-----------SPYITCETIWG-SGE-P--IQPSEYVG---IG
32_EGN97351_Serpula_lacrymans_var_lacrymans -LEV-----QTCQLDGLADLATNTT-AVQKILAAYANDLI-SLGVEGLRLDAAKNIAAS-DI-----GNITSLING-----------SPYITQEVIWG-ADQ-P--IQPSEYVY---IG
32_KDQ06010_Botryobasidium_botryosum        -QEV-----QTCELVNLADLATES-DYVRGKLAGYGNDLL-SLGADGFRLDAAKHMATN-DI-----AAILGKLSR-----------RPYISQEVIYG-DGE-P--ITPSEYTQ---NG
32_PPR00074_Panaeolus_cyanescens            -YNV-----QNCELVNLSDLKTES-DYVRGKLAGYANDLL-SLGVEGLRLDAAKHIPSG-DI-----ANIISRLSR-----------RPYITQEVIFG-NGE-P--VTPNEYVG---NG
32_EKM53319_Phanerochaete_carnosa           -VEV-----QTCQLDNLADLFTES-EYVRSRLAAYVNDLL-SLGVDGLRLDAAKHINAN-DI-----ANITSRLRR-----------APYITQEVIFG-TGE-P--ITPIQYVN---IG
32_TBU45601_Dichomitus_squalens             -LEV-----QTCELDNLADLATNT-TYVQGRLAEYGNDLL-SLGADGFRLDAAKHIAAT-DL-----AQILSQLNS-----------KPYITQEVIFG-EGE-P--ITPNEYVG---NG
32_PSR82077_Phlebia_centrifuga              -LEV-----QTCELENLADLATDT-EYVRSRLATYANDLL-SLGVDGLRLDAAKHMAST-DI-----ANITSRFTR-----------APYITQEVIYG-AGE-P--ITPNEYT------
32_KIM89391_Piloderma_croceum               -LEV-----QTCQLVGLADLATDTDDYVRGRLAEYANDLL-SLGVDGLRLDAAKHIAAT-DL-----ANITSRFTS-----------TPYITQEVIWG-SGE-P--IQPSEYVD---IG
32_PIL31175_Ganoderma_sinense               -AEV-----QTCELVNLADLRTET-EYVRGRLAEYGNDLL-SLGVDGLRLDAAKHIDTK-DI-----AAIVSQLKT-----------TPYITQEVVYG-TGE-P--VTPNEYEG---NG
32_KIY70325_Cylindrobasidium_torrendii      -AQI-----QTCELVNLADLATET-EYVRATLSTYVNDLV-SLGVDGLRLDAAKHIASS-DI-----SNILSRVNG-----------SVYITQEVIYD-ASG-PAGSLPADYVG---NG
32_KDQ58060_Jaapia_argillacea               -LEV-----QTCQLDGLADLATET-EYVRVRLAAYANDLI-SLGVDGLRLDAAKHMAAT-DV-----ANITSRLDG-----------APYITQEVIWG-AGE-P--IQPSEYVG---IG
32_CEL62905_Rhizoctonia_solani               -AEV-----QTCELVNLSDLATET-EYVRGKLAAYANDLL-SLGVVGLRLDAAKHIASG-DI-----ANILGRLTS-----------RPYITQEVIFG-SGE-P--ILPSEYTG---NG
32_GAT57350_Mycena_chlorophos               -LEV-----QTCELVNLADLATDT-EYVRAKLASYGNKLL-SLGVDGLRLDAAKHIAAT-DL-----ANITSRLVG-----------SPYLTQEVIFG-PGE-P--ISPLEYVG---IG
32_KIM25608_Serendipita_vermifera           -AQV-----QTCELVNLADLATET-EYVRARLATYLNDLV-SLGVDGFRLDAAKHMAAS-DI-----GNILSRVSS-----------VSYVSQEVIYG-SGE-P--VQPSEYVG---NG
32_GAW02849_Lentinula_edodes                -VEV-----WTCELEGLADLATDT-TYVQGRLAEYANDMA-SLGIDGLRLDAAKNIAPA-DI-----EAIVAQFDV-----------SLYLTQEVIWG-SGQ-P--VTPELYTG---IG
32_TCD63303_Steccherinum_ochraceum          -LEV-----QTCQLDGLADLATET-EYVRSRLAGYANDLL-SLGVDGLRLDAAKHIAAV-DL-----ANITSRLHG-----------SPYLSQEVIYG-AGE-P--ISPNEYVD---IG
32_KZP22139_Fibularhizoctonia_sp_CBS_109695 -LEV-----QTCQLDGLADLATNT-TAVQQRLAEYANDLI-SLGVDGLRLDAAKHIAATPDL-----TAIKALITG-----------SPYITQEVIWG-AGE-P--IQPSEYIG---IG
32_KII90441_Plicaturopsis_crispa            -TDV-----WDCQLGGLSDLATDTSENVRSKEAGYVNDMI-SLGVAGLRLDAAKSINPA-DI-----ANISSRFSS-----------KVYITQEVIWG-ANE-A--VDPSMYVD---NG
32_KDQ58058_Jaapia_argillacea               -VEV-----QTCQLVGLADLATET-EYVRARLAAYANDLL-GVGVDGLRLDAAKHMAAA-DV-----ANMTTRLHG-----------TPYITQEVIYG-AGE-P--SSPLNML------
32_OCH93218_Obba_rivulosa                   -TQA-----WDCELSGLADLATET-SHVQSVLSAYANDLL-SLGVTGLRLDAAKDIDPS-NI-----ASILALLDS-----------QPYITQEVIWG-EGQ-P--VTPQLYVG---NG
32_EMD36390_Gelatoporia_subvermispora       -EQV-----WNCELDGLADLATET-SHVQSVLSAYANDLL-SLGVTGLRLDAAKDIDPA-NI-----ATILSLLDS-----------QPYITQEVIWG-EGQ-P--VTPQLYVG---NG
32_EIW78746_Coniophora_puteana              -TQV-----WTCELENLADLATDTQ-YVQQIEANYVNDLL-SLGVKGLRLDAAKDIDPA-NI-----AAIAGMFTS-----------KPYITQEVIWG-DGQ-P--VTPQLYTG---NG
32_KII86244_Plicaturopsis_crispa            -VEV-----QTCELVNLADLATDT-EYVRGRLATYANDLL-SLGVDGFRLDAAKHIPAT-DI-----SNILGRLTS-----------SPYITQEVIWG-SGE-P--IQPSEYVS---NG
32_EKM75205_Agaricus_bisporus               -QEV-----QTCELVNLADLATDT-EYVRGRLAQYGNDLL-SLGVDGMRLDAAKHISAG-DL-----SNILGRLSR-----------RPYISQEVIFG-GGE-P--IQPSEYTG---NG
32_KZT36083_Sistotremastrum_suecicum        -TEV-----WTCELDGLADLATDTDATVKSRLAVYANDLL-SLGVDGFRLDASKSINYN-DI-----KIILGRVANT---Q---AGGRPYVTQEVIWG-AGQ-P--VTPAEYVQ---NG
32_KZS90559_Sistotremastrum_niveocremeum    -TEV-----WTCELDGLADLATDTDATVKSRLAVYANDLL-SLGVDGFRLDASKSINYN-DI-----KIILGRVANT---Q---AGGRPYITQEVIWG-AGQ-P--VTPAEYVQ---NG
32_EFI98134_Schizophyllum_commune           -EEV-----QTCELVNLADLATDT-DYVRGKLADYANDLI-SLGVDGLRLDASKHIPTD-DI-----AAIISRLNS-----------SVYITQEVIYG-DGE-P--ITPAEYVQ---NG
32_BBC62173_Tricholoma_matsutake            -QQV-----QTCQLDNLADLATET-EYVRGRLATYGNDLL-SLGIDGFRLDSAKHIAAG-DI-----SNILSRLSS-----------RPYITQEVIWG-PNE-A--VQPSEYVG---NG
32_CCA69081_Serendipita_indica              -AQV-----QTCELVNLSDLATET-EYVRAKLAAYMNDLV-SLGVDGFRIDAARHVATE-DI-----ANILSRVSA-----------AGYVSQEVSFDGDQN-A--VKPLEYVA---NG
32_EFI98135_Schizophyllum_commune           -KEV-----QTCELVNLADLATDT-VYVQARLAAYGNDLL-SLGVDGLRLDAAKHIPTG-EV-----ATIIDMLAS-----------HPYITQEVTSS-PDD-V--VKPEEYVQ---NG
32_KII85613_Plicaturopsis_crispa            -LEV-----QTCDLVGLPDLVTGD-TYVQGRLAQYGDDLV-SLGVQGFRLDAAKHIATT-DL-----ASILAMMKT----------KPCYITQETIYG-SGE-P--IIPSEYLP---NG
32_EIN11730_Punctularia_strigosozonata      -VEV-----QTCELDNLADLATET-DHVRQTLAAYGNDLL-SLGVSGLRLDASKQ-DVS-DI-----AAILAMLDS-----------TPYITQEVIYG-EGE-P--ITPNQYTG---NG
32_PIL33242_Ganoderma_sinense               -TEV-----QTCELAGLADLATET-EYVRSRLAQFGNDLL-SLDVDGLRLDAAKHMAVG-DI-----SNILSRLNK--------NGKNIYITQEVVFG-AGQ-P--VTPNEYTQ---TG
32_KZT18190_Neolentinus_lepideus            -QEV-----QTCQLDNLADLATDT-EYVRARLAAYANDLL-FLGVDGMRLDAAKHIAAT-DL-----GNITSRFMG-----------KPFITQEVIYG-EGE-P--IQPSEYTT---IG
32_KZV75308_Peniophora_sp_CONT              -TEV-----QTCQLSNLADLDTGA-EYPRSRLAEYVNDLL-SLGVDGLRIDAAKHISVT-DL-----ANITSRLNG-----------SPYITQEVVAG---G-A--VTPQMYVD---LG
32_KII86838_Plicaturopsis_crispa            -IEV-----QTCQLDSLADLATNS-TYVQAQLAAYGDDLV-SLGVQGFRLDAAKHMNTT-DI-----AAILAAMKT----------KPGYITQETIYG-SGE-P--VLPTEYLQ---NG
32_KDQ08018_Botryobasidium_botryosum        -QEV-----QTCELVNLADLATET-DYVRGKLAAYGNDLL-SLGADGFRLDAAKHIPQT-DI-----AAILGKLTR-----------PPYISQEVIFG-AGE-P--ITPNEYTP---NG
32_KIY73061_Cylindrobasidium_torrendii      -EQV-----QTCELVNLADLATDT-EYVRGRLAEYINDLL-SLDVDGLRLDAAKHIATD-DI-----ADILGRIDG-----------SPYISQEVIYG-GGE-P--VQPSEYVQ---NG
32_KZW01365_Exidia_glandulosa               -EEV-----QTCQLVNLADLDTGK-DEVQTAIAGYLSDLM-SLGVDGLRLDASKHMAAT-EI-----NGILGKMTT----------SPQYVTQEVIF-GA---GEPILPSEYTG---NG
32_KDQ58178_Jaapia_argillacea               -AEV-----QTCELVNLADLATET-EHVRAALAAYGNDLL-SLGVDGLRLDAAKHVAAA-DL-----INITSRFIR-----------KP-YMTQEVIW-GQ---SDAVQPGEYVG---IG
32_KZV75307_Peniophora_sp_CONT              -VEV-----QTCQLDNLADLDTGA-EYPRSRLAEYVNDLQ-SLGVDGFRLDAAKHISVA-DL-----TNIASRFTG-----------TPYLTQEVIYG-AGE-P--ITPDQYVG---IG
32_KIK51212_Gymnopus_luxurians              -EEV-----WTCQLEGLADLATDT-EYVRSRLAEYANDLI-SLGVDGLRLDAAKSIPPS-DI-----ANITSRLTP-----------IPYITQEVVWG-DDQ-P--VTPELYTD---IG
32_RDX41931_Polyporus_brumalis              -TEI-----QTCELAGLADLATET-EYVRSRLAQYGNDLL-SLGVDGLRLDAAKHMPVA-DI-----SNILSRLNT--------NGKSLYITQEVVYG-GGQ-P--VTPNQYTQ---TG
32_TBU29254_Dichomitus_squalens             -TSI-----QTCQLAGLADLATEK-EHVRSRLAQYGNDLL-SLNVDGLRIDAAKHMAVG-DI-----SNILSRLNK--------QGKNLYVTQEVVYG-AGQ-P--VTPNQYTQ---MG
32_KIY45802_Fistulina_hepatica              -TQV-----QTCQLDNLADLKTES-EYVRSRLAEHANDLL-SLDLDGFRIDAAKHIAVT-DL-----QNITSRFSP----------TPSYITQETYG-S----SGTIHPSDYTV---IG
32_KIL59382_Amanita_muscaria                -YEL-----QNCQLVGLADLNTGS-SYVQQSIINYVNDLR-TMGVDGFRLDAAKHIDTN-EI-----ATMLASVKG-----------NPYITQEVTYSGVGD----VQPGEYTQ---NG
32_KIY53725_Fistulina_hepatica              -WQV-----QNCQLDNLADLKTES-ESVREKLAAHANDLL-AISADGFRMDSAKEIPVE-DI-----TNITSRFIV----------KPSYITQETYF-A----SGNLYPSNYTV---NG
32_KIY47132_Fistulina_hepatica              -TSV-----QECELLGLADLATET-EDVQSRLAAYGNDLL-SLSVDGLRLDAAKHIPTG-DI-----ANILSRLNGT---------KRPYITQEITY-N---GAEAVRPSEYVQ---NG
32_RPD55342_Lentinus_tigrinus               -SEI-----QTCELAGLADLATDT-EYVRSRVAQYGNDLL-SLGVDGLRLDAAKHMPVA-DI-----SNILSRLNT--------NGKTLYITQEVVYG-GGQ-P--VTPNEYTQ---TG
32_TCD61445_Steccherinum_ochraceum          -TQV-----QTCELSNLADLATET-ETVRAKLAAFGNDLM-SLGVDGLRLDAAKHMPTA-DI-----SNIVGRLNP----------QPQYITQEVVAG-DGD-T--IQGSEYAG---IG
32_KIM25602_Serendipita_vermifera           -DQV-----QKCQLSGLADLATDT-DYVQGRIATYLKDLL-SLGVTGLRMDAAKHIATA-DI-----ANIFGRVGS-----------IPYVTQEVIYG-NNE-P--VQPSEYVQ---NG
32_EJD37603_Auricularia_subglabra            -QQV-----QTCQLVNLADLATDT-PNVQASIAAYLSDLL-SLGIDGFRVDAAKHIAAT-DI-----SAILARVSP----------APSYITQEVIF-GL---GEPITPNEYID---NG
32_KZT54766_Calocera_cornea                 -LEV-----QTCELLGMADLDTGS-EYVRSLLATYVNDLV-SLGVTGFRLDASKHIAAR-DI-----ANIVHRFEG-----------KH-YITQEVIY-GY---GEPIHPNEYVG---NG
32_KIY46805_Fistulina_hepatica              -YQV-----QNCELLGLADLKTES-EKVRATLAAYANDLV-SLGVDGFRLDAAKHIPNA-DI-----QNILSRLTT----------KPAYVTQETQF-A----SGHINPSDYIG---NG
32_PPQ69654_Psilocybe_cyanescens            -LEV-----QTCELVNLSDLATEA-EYVRSRLAAYGNDLL-SLGVDGLRLDAAKHIAAN-DL-----ANIVSRLSS-----------RPYITSEVIWG-GGE-P--IQPSEYTG---IG
32_KZO93971_Calocera_viscosa                -LEV-----QTCELLGLADLDTGS-EYVRELLAAYANDLA-SLGVTGFRLDASKHISST-DI-----ASIVKRFVG-----------KPYITQEVIYG-AGE-P--IQPNDYTG---NG
32_KZP07860_Fibularhizoctonia_sp_CBS_109695 -TNV-----QQCELGGLEDLATDT-ASVRTIQAAYLNDLM-SLGVKGFRFDAATWIAAA-DL-----AAIVGQLKT----------KATYITQETHW-I---EDCPILPTEYTS---IG
32_PVG03687_Serendipita_vermifera           -DQV-----QKCQLEGLADLATDT-DYVQGRIAEYLRDLL-SLGVTGLRMDAAKHIASS-DL-----SNILGRVGP-----------VP-YITQEVIY-GN---NEPVQPSEYVQ---NG
32_EIM87674_Stereum_hirsutum                -TDV-----WFCELDSLADLAQEE-SSVRDSIAGYLQNLL-DIGVAGFRLDAAKSIPPA-NI-----SAILGSLSG-----------SYYDTQEVYY-GAGE---AVSPADYVG---NG
32_CCA69087_Serendipita_indica              -DQV-----QKCQLVGLADLATDT-DYVQGRLADYLKDLL-STGVTGLRIDAAKHIASS-DI-----TNILSRVGP-----------VP-YITQEVIY-GD---NEPIQPSEYVA---NG
32_CEL62035_Rhizoctonia_solani              -EQL-----WNCELSNLADLNSNS-VEVQKKLAQFTNDLL-SLGVDGLRLDAAKHIHPW-DI-----GQVINQLSR----------KPDYITQEIID-TS---GS--MVGMYTG---IG
32_EIN05398_Punctularia_strigosozonata      -TQL-----QTCQLENLADLNTET-EYVRSRLAQYGNDLL-SLGVAGFRLDAAKHVNID-SL-----SNIVSRLSS-----------KPYITQEA-LG-SGQ-P--IPPESYNT---LG
32_EJU02896_Dacryopinax_primogenitus        -GEV-----QTCELLGLADLDTGS-EYVRKLLAAYANDLA-SLGVTGFRLDASKHIAAT-DI-----ANIVRRFVG-----------KP-YITQEVIY-GY---GEPIQPNEYTG---NG
32_KIO30302_Tulasnella_calospora            -TEV-----QFCELVNLADLDTAS-PAVRAKLAAYGNDLI-SLGVDGFRLDAAKHIPSG-DI-----AAILSALSK-----------NVYVTQEVSWG-SGE-P--IQPSEYTT---AG
32_CEL62036_Rhizoctonia_solani              -NQL-----WNCQLVNLADLNTSS-VYVQDKLADFTNDVL-GLGVDGLRLDAAKHMSPD-DI-----RAIMGKLSR----------RPSYISQEIVD-TG---GS--MVGMYTD---IG
32_KIO24533_Tulasnella_calospora            -TQL-----QNCELVNLADLKTES-SKVRNTLITHTNSLI-DLGVAGFRVDASKHVSTN-DI-----KYLQAKLKK----------QGLYYTHEVVY-EG---GGGVTSSEYMA---YG
32_EIM84100_Stereum_hirsutum                -TQI-----QFCQLENLADLATET-DYVRKTLVDEANDML-SLGVAGFRLDAAKHIPVT-DL-----QYIVSQLST-----------TPYITQEVI--GT---GQPIPESDYAS---LG
32_KDN35973_Rhizoctonia_solani              -TQV-----QTCELLNLADLATDT-ERVRSRLAEYLNDLI-SLGVVGLRIDAAKHIAAG-DI-----FDILSRIKS-----------NV-YITQEVIF-GT---GEPILPSEYVE---NG
32_KIY48489_Fistulina_hepatica              -TQI-----RLCQLENLADLDTEA-DNVQTKLAAQLDEFI-NLGVDGFRLDAAKHIYPS-SI-----ETILGKVSG----------SYSYITQEVDFDTG---GDGVWPSEYID---IG
32_KIY48486_Fistulina_hepatica              -WQI-----RNCQLENLADLNTTS-DKVRSTLAGHLDDLV-DLGVDGFRIDAAKHILPS-DI-----EAILAEMSG----------SVSYITQESEYSTG---GSGVYPSEYIS---IG
32_KZP26813_Fibularhizoctonia_sp_CBS_109695 -TNV-----WNCQLDGLADLATES-SNVQTKETAYANNLI-SKGVKGFRLDSAKSMDPA-NI-----KAVLSGVSS-----------EVYVTQEVVY------GGAVDPSEYTG---NG
32_KDQ32476_Pleurotus_ostreatus             -KEV-----QTCELLGLADLATGN-ENAQRRLKAYANDLR-SLGVDGFRLDAAKHIPAD-DL-----KSILSSVPGF-------GTPSFYVTQEVVG-GTKDDDSEVKATEYIQ---NG
32_EIM84087_Stereum_hirsutum                -YQV-----QNCQLANLADLKTED-DYVRKTIMQHANDML-DMGVAGFRLDAAKHIPME-DL-----NAIFGGLSR-----------KPFITSEVI--GS---GPPIPQTDYTG---LG
32_KDQ32475_Pleurotus_ostreatus             -NQL-----QKCELLGLADLDTKS-DVVRARLVEYAKDLR-SLGVDGFRIDAAKHIPAD-EL-----EAILSKVPGF-------RSPSLYITQEVFG-GTQP-GEEVRATEYLG---NG
32_EAQ88347_Chaetomium_globosum             -WSV-----QHCELVGLADLDTNE-DYVRKTIAGYMNDLL-SLGVDGFRIDAAKHIATE-DL-----TDIKSRLTNS----------GVYWKQEVIY-GAGE---AVQPTEYTR---NG
32_ORY89754_Leucosporidium_creatinivorum    -TSV-----YDCQLSNLQDLKSES-TTVQVAQAAYLNDLL-GLGVAGFRLDAAKSMRPE-NI-----QSIIDRLDS-----------KPLLAQEVNS-D---STQPVTPNMYSS---IG
32_EWZ34052_Fusarium_oxysporum              -RNV-----QHCELLGLADLDTHE-EYPRRAISGYMNDLL-SLGVDGFRIDAAKHMAAG-DL-----ANIKSRLANP----------SAYWKQEVIY-GAGE---AVYPTEYIG---NG
32_OBT91518_Pseudogymnoascus_verrucosus     -FNV-----QHCELVGLSDLDTNE-EYPRKAISGYINDLI-SLGVDGFRVDGAKHIATE-DL-----ANIKSRLANP----------AAYWKQEVIY-GAGE---AVQPTEYTG---NG
32_KEY73157_Stachybotrys_chartarum          -WNV-----QHCELVGLADLDTSE-TYVRQTIASYMNDLL-SLGVDGFRVDAAKHIATE-DL-----ANIKSRLANA----------GIYWKQEVIY-GSGE---AVQPTEYTS---NG
32_OBT62577_Pseudogymnoascus_sp_23342_1_I1  -YNV-----QHCELVGLADLDTNE-DYPRQAIAKYIDDLI-SLGVDGFRIDGAKHIATE-DL-----ANIKSRLANP----------SAYWKQEVIY-GAGE---AVQPTEYTS---NG
32_EON97874_Phaeoacremonium_minimum         -WNV-----QHCELVGLADLDTPE-EYPRKAISGYLNDLL-SLGVDGFRIDAAKHMATE-DL-----ANIKSRLTNP----------STYWKQEVIY-GSGE---AVQPTEYTG---NG
32_GAT55198_Mycena_chlorophos               -TQV-----RTCELVGLSDLDQSQ-PAVQATLVAYMNDLL-SLGVSGFRVDAARHMDPN-DL-----KTMFSQLNG-----------APYITQEVTS-G-----GASTPDEYTS---NG
32_OAG04271_Paraphaeosphaeria_sporulosa     -WNV-----QHCELVGLADLDTNE-DYVRGAIAGYMNDLL-SLGVDGFRIDAAKHTATE-DL-----ANMKSRLANS----------SVYWKQEVIY-GAGE---AVQPTEYTG---NG
32_EGG02960_Melampsora_larici_populina      -YEV-----QNCELLGLADLATET-TKVRDTIGTYLDSLA-RIGVTGFRMDASKHMPAT-DI-----RAILQRVTNR----------GRMHIVQEVIF-GFDE---PIRPEEYIM---NG
32_EGG02993_Melampsora_larici_populina      -YEV-----QNCELLGLADLATET-TKVRDTLGAYLEHLV-SLGVSGFRLDAAKHMPAS-DI-----QAILQRVSNH---------ESLHVSQEITI-GSNE---PIQPEEYVI---NG
32_OAV91411_Puccinia_triticina              -YEV-----QNCELVGLADLKTSQ-RHVQTQLKNYLTSLV-DKGVAGFRIDAAKHIPAC-DL-----LDILKLF-------------SVRVVQEVMY-TGGE---PIRPEEYSA---IG
32_EFP79978_Puccinia_graminis               -YEV-----QNCELVGLADLKTSQ-RHVQVQLKSYLTRLV-DKGVAGFRIDAAKHIPAS-EL-----LDILKLI-------------PIRVVQEVMY-TGGE---PIRPEEYSA---IG
32_GAT51088_Mycena_chlorophos               -TNRATVLTDWQKLSGLADLAQEQ-AGVRESIAAYLQVLI-NAGVAGFRVDSAKSMPPA-NI-----SAIFSLLSG-----------SFYDTQEVVY-GAGN-SPLVEPDQYTG---NG
32_KNE94052_Puccinia_striiformis            -YEV-----QNCELVGLADLKTSQ-RHVQNQLRRYLARLV-EKGVAGLRVDAAKHIPVR-DL-----LDILRFV-------------PLRVVQEIMY-TGGE---PIRPEEYST---VG
32_KNZ54553_Puccinia_sorghi                 ------EKVQNCELVGLADLRTSQ-GQVQDEQQRYLLRL-VDKQVAGFRIDAAKHIPAS-DL-----LNILKPIPLRT---V---QEVMYVGGEPIRP--EEYAPIGRVHVFQA-----
42_AOF40721_Microbacterium_aurum             -SDAKDCWIHE----DLADLNTES-AVVQDYLIDTYNKYI-DMGVDGFRIDTAVHIPRV-MWNRYFLPALQEHAVAVH---GE-KGKDFYVFGEVAQF-VHD----KW-----N---RG
42_CAB06816_Streptomyces_lividans           -EDSRSCWLHE----DLADFNTEN-PQVQNYLIGAYDKYI-DMGVDGFRVDTAVHIPRT-TWNRRFLPAIQERVAQQH---GAEAAKNFFVFGEVAAF-VND----KW-----N---RG
42_AWA81565_Pecoramyces_ruminatium          -FAETTGQFAG----DCEELNTEL-PFVYEYLVKVYKKYM-DMGVDAFRIDTVKHISRL-TLNSVFIPEFKKYAKEI-------GNDGFYMVGEVAGR-SSN----VF-----N---HD
```

| | |
|---|---|
| 1_AAA85446_Paenibacillus_polymyxa | AA-L---DFPMYYTIKD------------------------------------VFGHD--------QSMRKI---------------KD------------------------------ |
| 1_CAA49465_Thermoactinomyces_vulgaris | AV-L---DFPMYYTIKN------------------------------------VFGQD--------QSMRTI---------------AD------------------------------ |
| 1_BAD06003_Aspergillus_awamori | GV-L---NYPIYWQLLY------------------------------------AFES-------SSGSISDL---------------YN------------------------------ |
| 1_BAD06002_Aspergillus_awamori | GV-L---NYPIYYPLLN------------------------------------AFKS-------TSGSMDDL---------------YN------------------------------ |
| 1_AAF14264_Aspergillus_flavus | GV-L---NYPIYYPLLN------------------------------------AFKS-------TSGSMDDL---------------YN------------------------------ |
| 1_BAD01051_Aspergillus_kawachii | GV-L---NYPIYYPLLN------------------------------------AFKS-------TSGSMDDL---------------YN------------------------------ |
| 1_BAA22993_Aspergillus_kawachii | GV-L---NYPIYWQLLY------------------------------------AFES-------SSGSISNL---------------YN------------------------------ |
| 1_EAA64850_Aspergillus_nidulans | GV-L---NYPIYYPLLR------------------------------------AFSS-------TSGSLSDL---------------AN------------------------------ |
| 1_AAF17100_Aspergillus_nidulans | GV-M---NYPIYYPLLN------------------------------------AFKS-------SSGSMSDL---------------YN------------------------------ |
| 1_P56271_Aspergillus_niger | GV-L---NYPIYWQLLY------------------------------------AFES-------SSGSISNL---------------YN------------------------------ |
| 1_CAK44871_Aspergillus_niger | GV-L---NYPIYYPLLN------------------------------------AFKS-------TSGSMDDL---------------YN------------------------------ |
| 1_CAK40249_Aspergillus_niger | SL-P---NYPVYYAMLK------------------------------------AFTL---------GNTSAL---------------AT------------------------------ |
| 1_CAK41088_Aspergillus_niger | SL-P---NYPLYYSMIT------------------------------------AFLN---------GEPATL---------------LE------------------------------ |
| 1_CAA31218_Aspergillus_oryzae | GV-L---NYPIYYPLLN------------------------------------AFKS-------TSGSMDDL---------------YN------------------------------ |
| 1_BAA01255_Aspergillus_shirousami | GV-L---NYPIYYPLLN------------------------------------AFKS-------TSGSMDDL---------------YN------------------------------ |
| 1_AEB80431_Aspergillus_tubingensis | GV-L---NYPIYWQLLY------------------------------------AFES-------SSGSISNL---------------YN------------------------------ |
| 1_BAA12010_Cryptococcus_sp_S_2 | GV-L---NYPMFFYITN------------------------------------AFQT-------SSGSMSQL---------------AQ------------------------------ |
| 1_ABG48762_Fusicoccum_sp_BCC4124 | SV-L---NYPIYYPLVR------------------------------------AFTS-------SSGSISDL---------------VN------------------------------ |
| 1_AAO12212_Lipomyces_kononenkoae | SA-I---NYPLWYALVD------------------------------------SFMGR--------TTFDYL---------------ES------------------------------ |
| 1_AAC49622_Lipomyces_spencermartinsiae | GV-L---NYPVYYPVVS------------------------------------AFQR-------VGGSISSL---------------VD------------------------------ |
| 1_AAN75021_Lipomyces_starkeyi | GV-L---NYPVYYPVVS------------------------------------AFES-------VSGSVSSL---------------VD------------------------------ |
| 1_AFD54462_Malbranchea_cinnamomea | GV-L---NFPVYYPLID------------------------------------AFKS-------PSGTMWSL---------------VD------------------------------ |
| 1_ABF72529_Ophiostoma_floccosum | GV-L---NYPIYYQLLS------------------------------------AFES-------TSGSISNL---------------YN------------------------------ |
| 1_EPS26265_Penicillium_oxalicum | GV-L---NYPIYYQLLG------------------------------------AFKS-------TSGSISSL---------------YN------------------------------ |
| 1_ABO42285_Phanerochaete_chrysosporium | AI-L---QYPMYNALVE------------------------------------AFAIP------GPQNMSAM---------------SD------------------------------ |
| 1_BAW15173_Pholiota_microspora | SV-L---NFPLYTALTS------------------------------------AFSIP------GPQNMSAL---------------TQ------------------------------ |
| 1_BAF98616_Pichia_burtonii | GV-S---NYPLYYPVLR------------------------------------FFQN-------SKSTSGEL---------------LE------------------------------ |
| 1_AGJ52081_Rhizomucor_pusillus | PL-I---NYPMYYALND------------------------------------VFVSK------SKGFSRIS----------------E------------------------------ |
| 1_ADL28123_Rhizopus_oryzae | SL-I---NYPLYYPVYD------------------------------------VFTKS------RT-MSRLS---------------SG------------------------------ |
| 1_ADD80242_Saccharomycopsis_fibuligera | GV-S---NYPLYYPTTR------------------------------------FFKT-------TDSTSSEL---------------TQ------------------------------ |
| 1_ABS76467_Saitozyma_flava | GV-L---NYPMFFYITN------------------------------------AFQT-------SSGSMSQL---------------AQ------------------------------ |
| 1_CAB11471_Schizosaccharomyces_pombe | GL-H---NFPVQGVAAE------------------------------------SVIPL------NDRALRKT---------------AI------------------------------ |
| 1_CAB40006_Schizosaccharomyces_pombe | GV-T---NYFLQLYINF------------------------------------SFTA-------TGAGFTLI---------------PT------------------------------ |
| 1_CAA34162_Schwanniomyces_occidentalis | GV-T---NYPLYYPVYR------------------------------------FFSD-------TSATSSEL---------------TS------------------------------ |
| 1_CAA51912_Schwanniomyces_occidentalis | GV-T---NYPLYYPMLR------------------------------------FFQG-------TSNSVDEL---------------NA------------------------------ |
| 1_ACN82436_Sclerotinia_sclerotiorum | GV-L---NYPAYYWITQ------------------------------------AFQS-------TSGSISNL---------------VN------------------------------ |
| 1_CAA03110_Thermomyces_lanuginosus | GV-L---NYPIYYPALD------------------------------------AFKS-------VGGNLGGL---------------AQ------------------------------ |
| 1_BAG69580_Trichoderma_viride | GL-L---NYAIFYPLNA------------------------------------FYQQT------GSS--QAL---------------VD------------------------------ |
| 1_CAJ21046_Valsaria_rubricosa | GL-L---NYPIYYPLIR------------------------------------VFAQG------ASF--TDL---------------VN------------------------------ |
| 5_AWX66236_Alicyclobacillus_sp_18711 | -----------KTNW-S--------------------------------------------------M----SLFDVPLHMNFQAAANG------------------------------ |
| 5_AAQ01675_Alkalimonas_amylolytica | -----------AVNTPH--------------------------------------------------L----RAFDFNLREDFVALSSG------------------------------ |
| 5_CAL14744_Anoxybacillus_flavithermus | -----------KTNG-S--------------------------------------------------M----SLFDAPLHNNFYIASKS------------------------------ |
| 5_AEW07376_Bacillus_acidicola | -----------KTNG-T--------------------------------------------------M----SLFDAPLHSKFYTASKS------------------------------ |
| 5_AAA22191_Bacillus_amyloliquefaciens | -----------KTSF-N--------------------------------------------------Q----SVFDVPLHFNLQAASSQ------------------------------ |
| 5_ABY86223_Bacillus_cereus | -----------KVNY-N--------------------------------------------------Q----SVFDAPLHYNFHYASTG------------------------------ |
| 5_CAD26699_Bacillus_halmapalus | -----------KTNW-N--------------------------------------------------H----SVFDVPLHYNLYNASNS------------------------------ |
| 5_AAA22226_Bacillus_licheniformis | -----------KTNF-N--------------------------------------------------H----SVFDVPLHYQFHAASTQ------------------------------ |
| 5_AEM05860_Bacillus_licheniformis | -----------KTNF-N--------------------------------------------------H----SVFDVPLHYQFHAASTQ------------------------------ |
| 5_AAK00598_Bacillus_megaterium | -----------KVNY-N--------------------------------------------------Q----SVFDAPLHYNFHYASKG------------------------------ |
| 5_AGN35141_Bacillus_paralicheniformis | -----------KTNF-N--------------------------------------------------H----SVFDVPLHYQFHAASTQ------------------------------ |
| 5_AAR68734_Bacillus_sp | -----------KTSW-N--------------------------------------------------H----SVFDVPLHYNLYNASNS------------------------------ |
| 5_AAA22231_Bacillus_sp_707 | -----------KTNW-N--------------------------------------------------H----SVFDVPLHYNLYNASKS------------------------------ |
| 5_BAF03567_Bacillus_sp_JAMB_204 | -----------KTGY-N--------------------------------------------------Q----SVFDVPLHYNFQAASSQ------------------------------ |
| 5_CAC39917_Bacillus_sp_KSM_K38 | -----------EMNW-E--------------------------------------------------M----SLFDVPLNYNFYRASQQ------------------------------ |
| 5_AAB18785_Bacillus_sp_MK_716 | -----------KTNG-T--------------------------------------------------M----SLFDAPLHNKFYTASKS------------------------------ |
| 5_AAA63900_Bacillus_sp_TS_23 | -----------KTNG-S--------------------------------------------------M----SLFDAPLHNNFYTASKS------------------------------ |
| 5_ABW87262_Bacillus_sp_YX_YX1 | -----------KTSF-N--------------------------------------------------Q----SVFDVPLHFNLQAASSQ------------------------------ |
| 5_AAF00567_Cytophaga_sp | -----------KVNY-N--------------------------------------------------Q----SLFDAPLHYNFYAASTG------------------------------ |
| 5_AAC74994_Escherichia_coli | -----------QVEG-K--------------------------------------------------T----MLFDAPLQMKFHEASRM------------------------------ |
| 5_AFZ41193_Exiguobacterium_sp_DAU5 | -----------KTGY-T--------------------------------------------------H----SVFDVPLHYNFQAAGNG------------------------------ |
| 5_AAA22235_Geobacillus_stearothermophilus | -----------KTNG-T--------------------------------------------------M----SLFDAPLHNKFYTASKS------------------------------ |
| 5_ABX83871_Geobacillus_thermodenitrificans | -----------KTNG-T--------------------------------------------------M----SLFDAPLHFHDRTASKS------------------------------ |
| 5_AFC87833_Geobacillus_thermoleovorans | -----------KTNG-T--------------------------------------------------M----SLFDAPLHSKFYTASKS------------------------------ |
| 5_ACL70573_Halothermothrix_orenii | -----------TVGNPD--------------------------------------------------L----RVFDFPLRSFFVDMLNG------------------------------ |
| 5_CAQ30277_Nostoc_sp_PCC_7119 | -----------AVRG-K--------------------------------------------------M----SVFDVPLHYNFHQASKS------------------------------ |
| 5_AAA27110_Salmonella_typhimurium | -----------QVDG-K--------------------------------------------------T----MLFDAPLQMKFHEASRQ------------------------------ |
| 5_BAA24178_Streptococcus_equinus | -----------TTGH-R--------------------------------------------------F----DLIDVRLHQNLFEASKE------------------------------ |
| 5_AAA97431_Streptococcus_equinus | -----------STDY-R--------------------------------------------------F----DLVDVRLHQNLFEASKA------------------------------ |
| 5_AAN59233_Streptococcus_mutans | -----------SVDL-Q--------------------------------------------------F----DLIDVMLHMSLFEAGQK------------------------------ |
| 5_CCD30600_uncultured_bacterium | -----------TVNNDD--------------------------------------------------L----KVFDFPLRSSFEDMING------------------------------ |
| 5_ALP73597_Vibrio_alginolyticus | -----------KTSG-S--------------------------------------------------M----SLFDAPLHMNFYNASKS------------------------------ |
| 5_ABK62854_Histoplasma_capsulatum | -----------RMDY-I--------------------------------------------------V----SLFDAPLVRRFSDISRS------------------------------ |
| 5_ABS11196_Paracoccidioides_brasiliensis | -----------RMGH-L--------------------------------------------------V----SLFDAPLVHQFSYISTT------------------------------ |
| 5_BAW15172_Pholiota_microspora | -----------AFKG-E--------------------------------------------------A----AFFDVPLHMNLNRASTQ------------------------------ |
| 15_AAB60935_Aedes_aegypti | VV-T---EFNWCIVVGG------------------------------------VFRG---------------------------T--TNADALEL------------------------ |
| 15_AAB60934_Aedes_aegypti | TV-T---EFRYSAEIGR------------------------------------VFHG---------------------------R--DRLAHLSN------------------------ |
| 15_AAA03322_Aedes_atropalpus | TV-T---EFRFSAEIGR------------------------------------VFRG---------------------------Y--DRLAHLSN------------------------ |
| 15_AAA03323_Anopheles_merus | TV-T---EFMFSHYLGR------------------------------------AFGG---------------------------N--DALRWLSN------------------------ |
| 15_AAN77138_Anthonomus_grandis | RV-C---EFKYGMELAK------------------------------------CFRG---------------------------S--NPLKYLTN------------------------ |
| 15_AAN77139_Anthonomus_grandis | RV-C---EFKYGNLIGP------------------------------------CFKG---------------------------Q--CPFNLLNT------------------------ |
| 15_AAO13755_Asterias_rubens | RV-T---EFKYGTEVSL------------------------------------GFRG---------------------------D--KVLSHFNS------------------------ |
| 15_AAY23288_Blattella_germanica | RV-T---EFKYGRELSN------------------------------------AFRG---------------------------N--NAIKWLKN------------------------ |
| 15_BAB72257_Callosobruchus_chinensis | NV-L---EFRHGCELSK------------------------------------AFQG---------------------------S--NQLKNLKN------------------------ |
| 15_AAA03350_Culex_tarsalis | TV-T---EFRFSAEIGR------------------------------------VFRG---------------------------H--DRLANLRN------------------------ |
| 15_AAF20998_Diabrotica_virgifera_virgifera | AV-L---EFKYGSELGN------------------------------------AFQG---------------------------R--NDLHWLKS------------------------ |
| 15_AAC35243_Drosophila_ananassae | LV-T---EFRHSNSIGK------------------------------------VFGG---------------------------K--DQLRWLTN------------------------ |
| 15_BAB91544_Drosophila_bocki | AI-T---EFRHSDSIGK------------------------------------VFRG---------------------------K--DQLRYLSN------------------------ |
| 15_BAA04582_Drosophila_erecta | AI-T---EFRHSDSIGK------------------------------------AFRG---------------------------K--DQLQYLRN------------------------ |
| 15_BAB91509_Drosophila_kikkawai | AI-T---EFRHSDSIGK------------------------------------AFRG---------------------------K--NQLRYLSN------------------------ |
| 15_BAA95437_Drosophila_leontia | AI-T---EFRHSDSIGK------------------------------------AFRG---------------------------K--DQLRYLSN------------------------ |
| 15_BAA95441_Drosophila_lini | AI-T---EFRHSDSIGK------------------------------------AFRG---------------------------K--DQLRYLSN------------------------ |
| 15_BAA04583_Drosophila_mauritiana | AI-T---EFRHSDSIGK------------------------------------VFRG---------------------------K--DQLQYLTN------------------------ |
| 15_AAK93479_Drosophila_melanogaster_Amyrel | AV-T---EFRFSEEIGN------------------------------------AFRG---------------------------N--NALKWLQS------------------------ |
| 15_CAA28238_Drosophila_melanogaster | AI-T---EFRHSDSIGK------------------------------------VFRG---------------------------K--DQLQYLTN------------------------ |
| 15_AAC46839_Drosophila_pseudoobscura | AI-T---EFRHSDSIGK------------------------------------VFRG---------------------------K--DKLTYMSN------------------------ |
| 15_BAA04585_Drosophila_sechellia | AI-T---EFRHSDSIGK------------------------------------VFRG---------------------------K--DQLQYLTN------------------------ |
| 15_AAC46463_Drosophila_virilis | AI-T---EFRHSDSIGK------------------------------------VFRG---------------------------K--DQLRYLTN------------------------ |
| 15_AAG60011_Drosophila_yakuba | AV-T---EFRHSDSIGK------------------------------------VFRG---------------------------K--DQLQYLTN------------------------ |
| 15_CAA76926_Phaedon_cochleariae | TV-L---EFKYGTELGN------------------------------------AFQG---------------------------N--NALHNLEN------------------------ |
| 15_P56634_Tenebrio_molitor | CV-L---EFQFGVSLGN------------------------------------AFQG---------------------------G--NQLKNLAN------------------------ |
| 15_AAA03708_Tribolium_castaneum | TV-I---EFQYGLSLGN------------------------------------AFQG---------------------------G--NQLANLAN------------------------ |
| 15_AAF73435_Zabrotes_subfasciatus | RV-L---DFVHGGQLTN------------------------------------VFRG---------------------------Q--NQLKNLQS------------------------ |
| 24_ABL09312_Acarus_siro | RA-I---EFHFYRDIAN------------------------------------VVRR---------------------------H--NQLKTVKN------------------------ |
| 24_BAB85635_Anguilla_japonica | RV-T---EFKYSSKLGT------------------------------------VIRK---------------------------WNGEKMAYLRT------------------------ |
| 24_AAL37207_Crassostrea_gigas | RV-T---DFIYGIKIAE------------------------------------VFRH---------------------------H--NQAKWLRT------------------------ |
| 24_AAL37183_Crassostrea_gigas | RV-T---NFIYGIKLAD------------------------------------VFRH---------------------------H--NQAKWLKT------------------------ |
| 24_AAD38942_Dermatophagoides_pteronyssinus | RI-I---EFRFYKEITN------------------------------------VFRG---------------------------N--NPLHWLKN------------------------ |
| 24_AAD38943_Euroglyphus_maynei | RI-I---EFRFYKEITN------------------------------------VFRG---------------------------N--NQLRWLKN------------------------ |
| 24_AAC60246_Gallus_gallus | RV-T---EFKYGAKLGT------------------------------------VIRK---------------------------WNGEKMAYLKN------------------------ |
| 24_ABO26610_Haliotis_discus_discus | RV-T---NFIFGAKLAQ------------------------------------VFRK---------------------------Q--NAMKYLSN------------------------ |
| 24_BAM74656_Haliotis_discus_hannai | RV-T---NFIFGAKLAQ------------------------------------VFRK---------------------------Q--NAMKYLSN------------------------ |
| 24_AAA51724_Homo_sapiens_pancreatic | RV-T---EFKYGAKLGT------------------------------------VIRK---------------------------WNGEKMSYLKN------------------------ |
| 24_AAH63129_Homo_sapiens_salivary | RV-T---EFKYGAKLGT------------------------------------VIRK---------------------------WNGEKMSYLKN------------------------ |
| 24_AAA37221_Mus_musculus | RV-T---EFKYGAKLGK------------------------------------VMRK---------------------------WDGEKMSYLKN------------------------ |
| 24_AAA37230_Mus_musculus | RV-T---EFKYGAKLGT------------------------------------VIRK---------------------------WNGEKMSYLKN------------------------ |
| 24_H2N0D4_Oryzias_latipes | RV-T---EFKYGARLGE------------------------------------LFRK---------------------------WNGQKLSYTKN------------------------ |
| 24_CAA68065_Pecten_maximus | RV-T---NFIFGVKLGQ------------------------------------VFRN---------------------------E--NKASNLHN------------------------ |
| 24_CAA54524_Penaeus_vannamei | RV-T---EFRYGKYLGE------------------------------------AFRG---------------------------N--NQLKYLNN------------------------ |
| 24_CAB65552_Penaeus_vannamei | RV-T---EFRYGKYLGE------------------------------------AFRG---------------------------N--NQLKYLNN------------------------ |
| 24_AAF65827_Pseudopleuronectes_americanus | RV-T---EFKYGAKLGT------------------------------------VFRK---------------------------WNGEKLCYTRN------------------------ |
| 24_AAA40725_Rattus_norvegicus | RV-T---EFKYGAKLGT------------------------------------VIRK---------------------------WNGEKMSYLKN------------------------ |
| 24_AAH88228_Rattus_norvegicus | RV-T---EFKYGAKLGK------------------------------------VLRK---------------------------WDGEKMAYLKN------------------------ |
| 24_P83053_Struthio_camelus | RV-T---EFKYGAKLGT------------------------------------VIRK---------------------------WNGEKMAYLKN------------------------ |
| 24_AAF02828_Sus_scrofa | RV-T---EFKYGAKLGT------------------------------------VVRK---------------------------WSGEKMSYLKN------------------------ |
| 24_CAC87125_Tetraodon_nigroviridis | RV-T---EFKYGAKLGT------------------------------------VFRK---------------------------WNNEKLMYTKN------------------------ |
| 32_AAA21016_Aeromonas_hydrophila | TV-T---EFKYGTDLAA------------------------------------NFN----------------------------G---QIKNLKT------------------------ |
| 32_BAA22082_Bacillus_sp_195 | DI-H---EFAFARKLKE------------------------------------AFNG---------------------------G---TINWLT------------------------- |
| 32_AAY89038_Bifidobacterium_breve | KV-S---EFNFTNHLSV------------------------------------AFAG---------------------------D---ITDASK------------------------- |
| 32_CAB92963_Halomonas_meridiana | DI-T---EFRYGASLGD------------------------------------IFNN---------------------------Q---ALANLQQ------------------------ |
| 32_BAJ52728_Kocuria_varians | DV-H---EFSYARKLKE------------------------------------SFGG---------------------------G---RIDWLL------------------------- |
| 32_AKG25402_Microbacterium_aurum | DV-H---EFAYARKLKE------------------------------------AFGG---------------------------S---RIDWLI------------------------- |
| 32_CAA41481_Pseudoalteromonas_haloplanktis | LV-T---EFKYSTELGN------------------------------------TFRN---------------------------G---SLAWLSN------------------------ |
| 32_AAA86835_Pseudomonas_sp_KFCC10818 | DV-T---EFFFARTLGP------------------------------------KFKH---------------------------G---GIKDLQG------------------------ |
| 32_AAA96317_Streptomyces_albus | DV-Q---EFRYAYDLKR------------------------------------VFTQ---------------------------E---HLAYLK------------------------- |

| | |
|---|---|
| 32_BAC73693_Streptomyces_avermitilis | DV-Q---EFRYAYDLKR------------------------------------VFNN---------------------------E---NLAYLK------------------------- |
| 32_CAA40798_Streptomyces_griseus | DV-Q---EFRYARDLKR------------------------------------VFQN---------------------------E---NLAHLK------------------------- |
| 32_AAA26698_Streptomyces_hygroscopicus | DV-D---EFRYGTHLKS------------------------------------AFQS---------------------------G---NIAQLK------------------------- |
| 32_AAA88554_Streptomyces_limosus | DV-Q---EFRYARDLKR------------------------------------VFQN---------------------------E---NLAHLK------------------------- |
| 32_CAB06622_Streptomyces_lividans | DV-Q---EFRYARDLKR------------------------------------VFNG---------------------------E---NLAYLK------------------------- |
| 32_CAA73775_Streptomyces_sp_TO1 | DV-Q---EFPYAYDLKR------------------------------------VFNN---------------------------E---NLAYLK------------------------- |
| 32_AAA82875_Streptomyces_sp_WL6 | DV-D---EFRYGGHLKS------------------------------------AFQG---------------------------G---GIAQLK------------------------- |
| 32_AAA26697_Streptomyces_thermoviolaceus | DV-Q---EFRYAYDLKR------------------------------------VFTQ---------------------------E---HLAYLK------------------------- |
| 32_AAB36561_Streptomyces_venezuelae | DV-Q---EFRYARDLKR------------------------------------VLQG---------------------------E---KLSYLK------------------------- |
| 32_ABF13430_Thermobifida_fusca | SV-T---EFQYHRDISH------------------------------------AFAN---------------------------G---NIAHLT------------------------- |
| 32_CAA41881_Thermomonospora_curvata | SV-T---EFQYHRDISH------------------------------------AFAN---------------------------G---NIAHLT------------------------- |
| 32_BAV60949_Pholiota_microspora | DV-Q---EFRYTTAVQQ------------------------------------TFSS---------------------------G---GISNLQN------------------------ |
| 32_KJA26527_Hypholoma_sublateritium | DV-Q---EFRYTSALQS------------------------------------AFSG---------------------------G---GISSLQN------------------------ |
| 32_KDR78015_Galerina_marginata | DV-Q---EFRYTSAIKS------------------------------------AFSG---------------------------G---GISGLQN------------------------ |
| 32_PPQ83160_Psilocybe_cyanescens | DV-Q---E--YTSALKD------------------------------------AFSG---------------------------G---GISSLQN------------------------ |
| 32_KTB32361_Moniliophthora_roreri | DV-Q---EFRYTTTLKS------------------------------------TFLG---------------------------G---GISSLQN------------------------ |
| 32_KDQ30003_Pleurotus_ostreatus | DV-Q---EFRYTSELKN------------------------------------AFSG---------------------------G---GIANLQD------------------------ |
| 32_RDX41646_Polyporus_brumalis | DV-Q---EFRYTSTLKD------------------------------------SFLG---------------------------G---TISNLQS------------------------ |
| 32_OJT13448_Trametes_pubescens | DV-Q---EFRYTSALKD------------------------------------AFSS---------------------------S---GISNLQD------------------------ |
| 32_PFH45132_Amanita_thiersii | DV-Q---EFRYTTALRD------------------------------------AFSG---------------------------G---GISSLQN------------------------ |
| 32_KIM37787_Hebeloma_cylindrosporum | DV-Q---EFRYTTALKD------------------------------------AFSG---------------------------Q---GISSLQN------------------------ |
| 32_PBK61492_Armillaria_solidipes | DV-Q---EFRYTTALQS------------------------------------AFGG---------------------------G---SISGLEN------------------------ |
| 32_ETW76368_Heterobasidion_irregulare | DV-Q---EFRYTTALQD------------------------------------AFSS---------------------------S---GISGLQN------------------------ |
| 32_RDB27268_Hypsizygus_marmoreus | DV-Q---EFRYTSALRD------------------------------------AFLG---------------------------G---GISNLQS------------------------ |
| 32_PPQ71147_Gymnopilus_dilepis | DV-Q---EFRYTTTLMN------------------------------------AFLN---------------------------N---GIASLQN------------------------ |
| 32_OSD07916_Trametes_coccinea | N----------------------------------------------------AFLG---------------------------G---GISSLQS------------------------ |
| 32_KZW01389_Exidia_glandulosa | DV-Q---EFRYTTALKN------------------------------------AFQS---------------------------N---GISSLQN------------------------ |
| 32_EIW55835_Trametes_versicolor | DV-Q---EFRYTSALKD------------------------------------AFSG---------------------------S---GISSLQD------------------------ |
| 32_SJL11930_Armillaria_ostoyae | DV-Q---EFRYTTALQS------------------------------------AFGG---------------------------G---SISGLEN------------------------ |
| 32_PAV18085_Pyrrhoderma_noxium | DV-Q---EFRYTTAVKN------------------------------------GFLS---------------------------D---GISGLQN------------------------ |
| 32_RPD65356_Lentinus_tigrinus | DV-Q---EFRYTSTLKD------------------------------------SFLG---------------------------G---TISNLQS------------------------ |
| 32_PBK84861_Armillaria_gallica | DV-Q---EFRYTTALQT------------------------------------AFTG---------------------------G---SISGLEN------------------------ |
| 32_KLO18298_Schizopora_paradoxa | DV-M---EFRYTTALLN------------------------------------SFLG---------------------------SNGANISGLQD------------------------ |
| 32_PPR08271_Panaeolus_cyanescens | DV-Q---EFRYTSAVRD------------------------------------AFSG---------------------------N-GDGIASLQS------------------------ |
| 32_KIK53845_Gymnopus_luxurians | NV-Q---EFRYTTALFN------------------------------------AFSG---------------------------G---GISNLQN------------------------ |
| 32_KIJ56431_Sphaerobolus_stellatus | DV-Q---EFRYTSTLQS------------------------------------AFSG---------------------------S--QALSSLQN------------------------ |
| 32_EJD07843_Fomitiporia_mediterranea | -------MFRYTTALKN------------------------------------AFLS---------------------------D---GIASLQN------------------------ |
| 32_EPQ53992_Gloeophyllum_trabeum | DA-Q---EFRYTTALKN------------------------------------AFTG---------------------------G---GISSLED------------------------ |
| 32_KIP11288_Phlebiopsis_gigantea | DV-Q---EFRYTTAFQN------------------------------------AFTN---------------------------G---SISSLQS------------------------ |
| 32_KIY62009_Cylindrobasidium_torrendii | DV-Q---EFRYTSTVRD------------------------------------AFNN---------------------------G---DISGLRD------------------------ |
| 32_KIJ69360_Hydnomerulius_pinastri | DV-M---EFRYTSALQT------------------------------------GFSG---------------------------G---GINTLEN------------------------ |
| 32_KZT20455_Neolentinus_lepideus | DA-Q---EFRYTTALQN------------------------------------AFTD---------------------------T---GISGLKD------------------------ |
| 32_EGN97351_Serpula_lacrymans_var_lacrymans | DV-M---EFRYTSALQS------------------------------------AFGG---------------------------G---GISSLQD------------------------ |
| 32_KDQ06010_Botryobasidium_botryosum | DV-Q---EFRYTYTVKS------------------------------------AFES---------------------------S---GITNLQK------------------------ |
| 32_PPR00074_Panaeolus_cyanescens | DV-Q---EFRYTSALKD------------------------------------AFQN---------------------------G---NIASLQN------------------------ |
| 32_EKM53319_Phanerochaete_carnosa | DV-Q---EFRYTTAVQN------------------------------------AFSG---------------------------S---GISGLQS------------------------ |
| 32_TBU45601_Dichomitus_squalens | DV-Q---EFRYTTALKN------------------------------------AFLG---------------------------G---GISSLES------------------------ |
| 32_PSR82077_Phlebia_centrifuga | ----------------------------------------------------------------------------------------------------------------------- |
| 32_KIM89391_Piloderma_croceum | SV-Q---EFRYTNSLKD------------------------------------AFLG---------------------------G---GISSLEN------------------------ |
| 32_PIL31175_Ganoderma_sinense | DV-Q---EFRYTTALLK------------------------------------AFSG---------------------------E---GISSLQS------------------------ |
| 32_KIY70325_Cylindrobasidium_torrendii | NV-Q---EFRFTYTVRD------------------------------------AFNN---------------------------G---DISGLED------------------------ |
| 32_KDQ58060_Jaapia_argillacea | DV-Q---EFRYTTALQQ------------------------------------AFTS---------------------------T---GISTLEN------------------------ |
| 32_CEL62905_Rhizoctonia_solani | DV-Q---EFRYTSTIQN------------------------------------AFQS---------------------------G---GISSLNG------------------------ |
| 32_GAT57350_Mycena_chlorophos | DV-Q---EFRYTSTLKS------------------------------------AFLG---------------------------G---GINTLQS------------------------ |
| 32_KIM25608_Serendipita_vermifera | AV-Q---EFRYTSTIKD------------------------------------AFQS---------------------------S---GISSLQN------------------------ |
| 32_GAW02849_Lentinula_edodes | NV-Q---EFRYTTALQS------------------------------------AFLG---------------------------G---GISNLEN------------------------ |
| 32_TCD63303_Steccherinum_ochraceum | AV-QDIFRFRYTSTLQS------------------------------------AFLE---------------------------G---GISSLKD------------------------ |
| 32_KZP22139_Fibularhizoctonia_sp_CBS_109695 | NV-M---EFRYTTALQN------------------------------------AFTN---------------------------G---SINSLES------------------------ |
| 32_KII90441_Plicaturopsis_crispa | DV-M---EFRYTTALQT------------------------------------AFTN---------------------------S---GISNLQN------------------------ |
| 32_KDQ58058_Jaapia_argillacea | ES-V---IFRYTSALQQ------------------------------------AFSY---------------------------S---GISNLEF------------------------ |
| 32_OCH93218_Obba_rivulosa | DV-Q---EFRYTSALQS------------------------------------AFTG---------------------------G---GISSLES------------------------ |
| 32_EMD36390_Gelatoporia_subvermispora | DV-Q---EFRYTSALQS------------------------------------AFTG---------------------------G---GISSLES------------------------ |
| 32_EIW78746_Coniophora_puteana | DV-M---EFRYTSALQS------------------------------------AFLG---------------------------G---GIDSLQD------------------------ |
| 32_KII86244_Plicaturopsis_crispa | DV-Q---EFRYTSAVKD------------------------------------AFLG---------------------------G---GISGLQD------------------------ |
| 32_EKM75205_Agaricus_bisporus | DV-Q---EFRYTTTIRD------------------------------------AFSG---------------------------G---GISSLQN------------------------ |
| 32_KZT36083_Sistotremastrum_suecicum | DV-M---EFRFASTIQT------------------------------------ALSS---------------------------S---GLSGMVN------------------------ |
| 32_KZS90559_Sistotremastrum_niveocremeum | DV-M---EFRFASTIQT------------------------------------ALSS---------------------------S---GLSGMVN------------------------ |
| 32_EFI98134_Schizophyllum_commune | DV-Q---EFRYTHTIQD------------------------------------AFTN---------------------------S---GLDQLQD------------------------ |
| 32_BBC62173_Tricholoma_matsutake | -------VFRYTTALKN------------------------------------AFLG---------------------------SGGSSISELQD------------------------ |
| 32_CCA69081_Serendipita_indica | AV-Q---EY-------D------------------------------------AFTS---------------------------S---GISQLQT------------------------ |
| 32_EFI98135_Schizophyllum_commune | DV-Q---EFRYTRALQS------------------------------------AFLG---------------------------G---GIDQLKD------------------------ |
| 32_KII85613_Plicaturopsis_crispa | DA-Q---EFRYTASLKS------------------------------------AFAN---------------------------G---AINGLQY------------------------ |
| 32_EIN11730_Punctularia_strigosozonata | DV-Q---EFRYTTALQD------------------------------------AFGG---------------------------S---GISSLED------------------------ |
| 32_PIL33242_Ganoderma_sinense | DV-Q---EFRFTDALRD------------------------------------AFTH---------------------------S--SQLSDLQN------------------------ |
| 32_KZT18190_Neolentinus_lepideus | DV-Q---EFRYTFLIKD------------------------------------AFTT---------------------------S---GIAGLQN------------------------ |
| 32_KZV75308_Peniophora_sp_CONT | NV-Q---EFRYAEELKK------------------------------------AFSS---------------------------S---GISELQD------------------------ |
| 32_KII86838_Plicaturopsis_crispa | DA-Q---EFRYASTLYY------------------------------------AFGD---------------------------G---PISGLQN------------------------ |
| 32_KDQ08018_Botryobasidium_botryosum | DV-Q---EFRYTSALKS------------------------------------AFEG---------------------------Q---GISSLPN------------------------ |
| 32_KIY73061_Cylindrobasidium_torrendii | NV-Q---EFRYTSTLQS------------------------------------AFGN---------------------------G---DISGLQD------------------------ |
| 32_KZW01365_Exidia_glandulosa | NV-Q---EFRFTSTLQD------------------------------------AFSS---------------------------G---GISS--------------------------- |
| 32_KDQ58178_Jaapia_argillacea | NV-Q---EFRYTTALKE------------------------------------AFSM---------------------------T---GIAN--------------------------- |
| 32_KZV75307_Peniophora_sp_CONT | NV-Q---EFRYTTELQN------------------------------------AFGG---------------------------S---GISALQD------------------------ |
| 32_KIK51212_Gymnopus_luxurians | NV-Q---EFRYTTAVMN------------------------------------AFSG---------------------------D---GIADLQN------------------------ |
| 32_RDX41931_Polyporus_brumalis | -------AFRFMDALRD------------------------------------AFTG---------------------------K--SNLSDLKD------------------------ |
| 32_TBU29254_Dichomitus_squalens | HV-Q---EFRFKDALTA------------------------------------AFTG---------------------------S--SRLSDLQN------------------------ |
| 32_KIY45802_Fistulina_hepatica | DA-Q---EFGFMSAMKS------------------------------------AFEN---------------------------D---ASTTYSSLS---------------------- |
| 32_KIL59382_Amanita_muscaria | DV-Q---EFRYPATLNK------------------------------------AFSS---------------------------G---NISALQD------------------------ |
| 32_KIY53725_Fistulina_hepatica | DA-Q---EFNFPTTVKA------------------------------------AFTN---------------------------N---ETSI-SELE---------------------- |
| 32_KIY47132_Fistulina_hepatica | -------MFRYPLAFLQ------------------------------------AFSN---------------------------D---SVGI----S---------------------- |
| 32_RPD55342_Lentinus_tigrinus | -------IFRFMDALRD------------------------------------AFTG---------------------------K--SNLSDLKD------------------------ |
| 32_TCD61445_Steccherinum_ochraceum | DV-Q---EFRWTTAIQK------------------------------------AFSG---------------------------G---GISSLQS------------------------ |
| 32_KIM25602_Serendipita_vermifera | NV-Q---EFRYTQAVQS------------------------------------AFMG---------------------------D---GIIWLQE------------------------ |
| 32_EJD37603_Auricularia_subglabra | NV-Q---EFRYTSTLRD------------------------------------AFSGV-------------------------SA---GIAQ--------------------------- |
| 32_KZT54766_Calocera_cornea | DV-Q---EFRYTSALQN------------------------------------AFLS---------------------------S---GIAS--------------------------- |
| 32_KIY46805_Fistulina_hepatica | DA-Q---EFNYPVTLKN------------------------------------AFLG---------------------------N---GATI-SQLK---------------------- |
| 32_PPQ69654_Psilocybe_cyanescens | DV-Q---EFRYTTALKD------------------------------------AFSG---------------------------G---GISNLQN------------------------ |
| 32_KZO93971_Calocera_viscosa | DV-Q---EFRYTSALQN------------------------------------AFLS---------------------------S---GIASLTS------------------------ |
| 32_KZP07860_Fibularhizoctonia_sp_CBS_109695 | DA-Q---EFRYVVALKA------------------------------------AFLG---------------------------S---SSSI----S---------------------- |
| 32_PVG03687_Serendipita_vermifera | NV-Q---EFRYTDTLKW------------------------------------AFTE---------------------------G---GIDS--------------------------- |
| 32_EIM87674_Stereum_hirsutum | DV-I---EFRVPETVLT------------------------------------YFTS---------------------------Q---GIANMVT------------------------ |
| 32_CCA69087_Serendipita_indica | AV-Q---EFRYTNTLKW------------------------------------AFTE---------------------------G---GIDT--------------------------- |
| 32_CEL62035_Rhizoctonia_solani | DI-Q---EFRYPEALRD------------------------------------AFNW---------------------------N---GIAG--------------------------- |
| 32_EIN05398_Punctularia_strigosozonata | DV-T---VSDYYGDLQK------------------------------------AFTS---------------------------T---NLASLNG------------------------ |
| 32_EJU02896_Dacryopinax_primogenitus | EYYL---LFRYTSALQS------------------------------------AFLS---------------------------N---GIVS--------------------------- |
| 32_KIO30302_Tulasnella_calospora | DV-Q---EFRYLSALKD------------------------------------AFTS---------------------------SNLASLANIAS------------------------ |
| 32_CEL62036_Rhizoctonia_solani | DV-Q---EFRYPEALKA------------------------------------AFTG---------------------------G---GIAG--------------------------- |
| 32_KIO24533_Tulasnella_calospora | DV-Q---EFRYTSLLSN------------------------------------AFNS---------------------------G---NIAQ--------------------------- |
| 32_EIM84100_Stereum_hirsutum | TV-S---VSDYQVAVET------------------------------------AFKT---------------------------G---GIDT--------------------------- |
| 32_KDN35973_Rhizoctonia_solani | DV-Q---EFRYASALRN------------------------------------AFQA---------------------------D---GIAK--------------------------- |
| 32_KIY48489_Fistulina_hepatica | QV-N---AFEWAYYGLT------------------------------------AFLA---------------------------D---GLTA--------------------------- |
| 32_KIY48486_Fistulina_hepatica | QV-N---AFEFAYYMRS------------------------------------AFFY---------------------------D---GLSA--------------------------- |
| 32_KZP26813_Fibularhizoctonia_sp_CBS_109695 | DV-M---EFRFASALQS------------------------------------AFLG---------------------------S---GISS--------------------------- |
| 32_KDQ32476_Pleurotus_ostreatus | DV-Q---VFEYSKKIRE------------------------------------AFMK---------------------------G---SFTI--------------------------- |
| 32_EIM84087_Stereum_hirsutum | LV-S---ASDYQDMLQK------------------------------------ALRS---------------------------D---GLVS--------------------------- |
| 32_KDQ32475_Pleurotus_ostreatus | DV-H---VFDYANEIKA------------------------------------AFLS---------------------------G---NIAS--------------------------- |
| 32_EAQ88347_Chaetomium_globosum | DV-Q---EFRYAYDLKR------------------------------------VFND---------------------------E---KLAYLK------------------------- |
| 32_ORY89754_Leucosporidium_creatinivorum | GV-L---EFRAHYSLVS------------------------------------AFVK---------------------------G---NGIK--------------------------- |
| 32_EWZ34052_Fusarium_oxysporum | DV-Q---EFRYAYGLKR------------------------------------VFNN---------------------------E---KLAYLR------------------------- |
| 32_OBT91518_Pseudogymnoascus_verrucosus | DV-Q---EFRYAYDLKR------------------------------------VFNS---------------------------E---KLAYLS------------------------- |
| 32_KEY73157_Stachybotrys_chartarum | DV-Q---EFRYAYDLKR------------------------------------VFDN---------------------------E---RLAYLS------------------------- |
| 32_OBT62577_Pseudogymnoascus_sp_23342_1_I1 | DV-Q---EFRYAYGLKS------------------------------------VFNN---------------------------E---KLAYLA------------------------- |
| 32_EON97874_Phaeoacremonium_minimum | DV-Q---EFRYAYDLKR------------------------------------VLNS---------------------------E---KLAYLK------------------------- |
| 32_GAT55198_Mycena_chlorophos | DI-I---EFDATAFIQA------------------------------------AFTG---------------------------S---SGSVSNLVT---------------------- |
| 32_OAG04271_Paraphaeosphaeria_sporulosa | DV-Q---EFRYAFDLKP------------------------------------VFNN---------------------------E---KLAYLR------------------------- |
| 32_EGG02960_Melampsora_larici_populina | NV-H---VFQGASDLKR------------------------------------LFKT---------------------------D---GMAHLVH------------------------ |
| 32_EGG02993_Melampsora_larici_populina | NV-Q---IFQGASDLER------------------------------------LFKT---------------------------D---GMAYLVQ------------------------ |
| 32_OAV91411_Puccinia_triticina | RV-H---IFQAAYDLKR------------------------------------MFLS---------------------------D---GIAYLGQ------------------------ |
| 32_EFP79978_Puccinia_graminis | RV-H---IFQAAYDLKR------------------------------------MFLS---------------------------D---GIAYLGH------------------------ |
| 32_GAT51088_Mycena_chlorophos | DV-I---EFRVXXXXTTRKKWCTARV----------------TRLSSNQISTLAMEI---------------------------E---GIANMVT------------------------ |
| 32_KNE94052_Puccinia_striiformis | RV-H---IFQAAYDLKR------------------------------------MFLS---------------------------D---GIAYLGH------------------------ |
| 32_KNZ54553_Puccinia_sorghi | -----------AYDLKR--------------------------------------------------MFLVRTLTYLPWPDSFYNQSDGIAYLGHVSTPLSTILMTYTYHPVLIFLASY |
| 42_AOF40721_Microbacterium_aurum | SV-N---HSAPFYTWKERREYSTND----VQAVIEQYQYENLMGGDGQPRSTNARLNGNNYHTPDHSQFSGMNIIDMRMHMNFDNASNA------------------------------ |
| 42_CAB06816_Streptomyces_lividans | SV-T---HSAQFYTWKERKEYSADD----AKAALEMYEYEQQQGTGNQPTSANAFLEGNSYHVPDHSRFSGMNVIDMRMHMNFGDASNA------------------------------ |
| 42_AWA81565_Pecoramyces_ruminatium | VV-Q---SSPLYWTWNEPKDYGWNYESTDGKDNLEKTRQE-WTDGATPRKSNNAFLNGNNYHTPDYSEFSGLNIIDFTMHHSFENANNA------------------------------ |

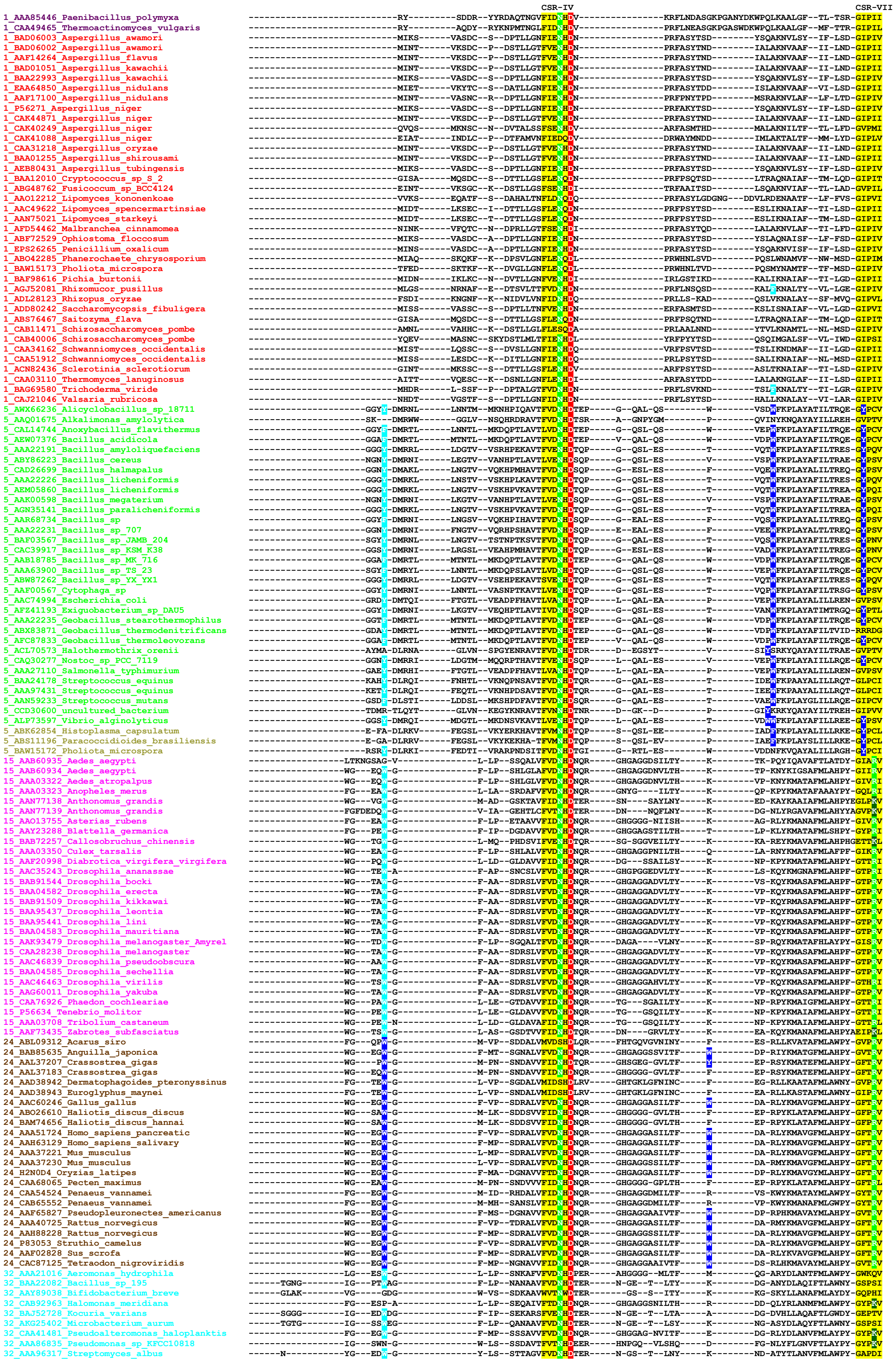
CSR-IV
CSR-VII

CSR-IV (FVDNHDTER block) · CSR-VII (final block)

| | |
|---|---|
| 32_BAC73693_Streptomyces_avermitilis | ------N-----------YG---EGW-G---------------Y-LN--SSVAGVFVDNHDTER-----N-GS--T--LNY-----K--------DG-ANYTLANVFMLAYPY-GAPDI |
| 32_CAA40798_Streptomyces_griseus | ------N-----------FG---EDW-G---------------Y-MA--SGKSAVFVDNHDTER-----G-GD--T--LNY-----K--------NG-SAYTLAGVFMLAWPY-GSPDV |
| 32_AAA26698_Streptomyces_hygroscopicus | ------S-----------VA---D---G---------------K-LW--QRQARTFVDNWDTER-----N-GS--T--LTY-----K--------DG-AAYTLANVFMLASPY-GSPNV |
| 32_AAA88554_Streptomyces_limosus | ------N-----------FG---EDW-G---------------Y-MA--SGKSAVFVDNHDTER-----G-GD--T--LNY-----K--------NG-SAYTLAGVFMLAWPY-GSPDV |
| 32_CAB06622_Streptomyces_lividans | ------N-----------FG---EAW-G---------------H-LP--SDEAAVFVTNHDTER-----N-GE--T--LTY-----K--------DG-ATYTLAHVFMLAWPY-GSPDV |
| 32_CAA73775_Streptomyces_sp_TO1 | ------N-----------YG---EAW-G---------------Y-LN--SSVAGVFVDNHDTDA-----N-GS--T--LNY-----R--------DG-ANYTLANVFMLAWPY-GAPDI |
| 32_AAA82875_Streptomyces_sp_WL6 | ------A-----------VA---D---R---------------K-LG--SASARTFVDNWDTER-----N-GS--T--LTY-----K--------DG-AAYTLANVFMLASPY-GSPNV |
| 32_AAA26697_Streptomyces_thermoviolaceus | ------N-----------YG---EDW-G---------------Y-LS--STTAGVFVDNHDTER-----N-GS--T--LNY-----K--------ND-ATYTLANVFMLAWPY-GAPDI |
| 32_AAB36561_Streptomyces_venezuelae | ------N-----------FG---EAW-G---------------H-MP--SGQSGVFVDNHDTER-----G-GD--T--LSY-----K--------DG-ANYTLASVFMLAWPY-GSPDV |
| 32_ABF13430_Thermobifida_fusca | ------G-----------LG---S---G---------------L-TP--SDKAVVFVVNHDTQR-----Y-EP--I--LTH-----T--------DG-ARYDLAQKFMLAHPY-GTPKV |
| 32_CAA41881_Thermomonospora_curvata | ------G-----------LG---S---G---------------L-TP--SDKAVVFVVNHDTQR-----Y-EP--I--LTH-----T--------DR-ARYDLAQKFMLAHPY-GTPKV |
| 32_BAV60949_Pholiota_microspora | -------------------L---DN-QG---------------W-VS--GTQANVFVANHDTER-----N-G----ASLNF-----N--------SPSNTYILATVFSLAHPY-GTPTV |
| 32_KJA26527_Hypholoma_sublateritium | -------------------L---DS-QG---------------W-VA--GTSANVFVANHDTER-----N-G----GSLNM-----N--------SPSNTYITATIFSLAHPY-GTPTV |
| 32_KDR78015_Galerina_marginata | -------------------L---DS-QG---------------W-VS--GSKANVFVANHDTER-----N-G----NSLNF-----K--------SPSNTYITATIFSLAHPY-GTPTV |
| 32_PPQ83160_Psilocybe_cyanescens | -------------------I---DN-EG---------------W-VS--GSQANVFVANHDTER-----N-G----NSLNI-----N--------SASNTYITATIFSLAHPY-GTPTI |
| 32_KTB32361_Moniliophthora_roreri | -------------------L---DN-RG---------------W-VS--GSQANVFVTNHDTER-----N-G----DSLNS-----N--------SPSNTYVTATIFSLAHPY-GTPTV |
| 32_KDQ30003_Pleurotus_ostreatus | -------------------L---EN-RG---------------W-VT--GSKANVFVTNHDTER-----N-G----ASLNA-----N--------SPSNTYVTATIFSLAHPY-GTPTI |
| 32_RDX41646_Polyporus_brumalis | -------------------F---DN-LG---------------W-VD--GSVANVFVTNHDTER-----N-G----NSLNN-----N--------SPSNTYVSAMVFSLAHPY-GTPTI |
| 32_OJT13448_Trametes_pubescens | -------------------F---EN-RG---------------W-VP--GTGANVFVTNHDTER-----N-G----ASLNN-----N--------SPSNTYVTAMIFSLAHPY-GTPSI |
| 32_PFH45132_Amanita_thiersii | -------------------L---DN-RG---------------W-VS--GSQANVFVANHDTER-----N-G----GSLNI-----N--------SASNAYILATLFSLAHPY-GTPTI |
| 32_KIM37787_Hebeloma_cylindrosporum | -------------------L---DN-RD---------------W-VA--GSQANVFVANHDTER-----N-G----NSLTY-----S--------SPSNTYITATIFSLAHPY-GTPSV |
| 32_PBK61492_Armillaria_solidipes | -------------------L---D---R---------------W-IS--GSQANVFVANHDTER-----N-G----NSLNV-----N--------SANNAYVIATIFSLAHPY-GTPTI |
| 32_ETW76368_Heterobasidion_irregulare | -------------------L---DS-NG---------------W-VS--GSGANVFVADHDTER-----N-G----NSLNA-----N--------SPSNTYYLAHIFSLAHPY-GTPTV |
| 32_RDB27268_Hypsizygus_marmoreus | -------------------L---DT-RG---------------W-VS--GSQANVFVANQDTER-----S-G----ASLNI-----N--------SPSNTYITATIFSLAHPY-GTPTV |
| 32_PPQ71147_Gymnopilus_dilepis | -------------------L---DN-QG---------------W-VS--GSQANIFVTNHDTER-----N-G----NSLNI-----N--------SPSNTYITATIFSLAHPY-GRPTV |
| 32_OSD07916_Trametes_coccinea | -------------------F---DN-LG---------------W-VP--GTGANVFVTNHDTER-----N-G----NSLND-----N--------SPSNTYVTAMIFSLAHPY-GTPTI |
| 32_KZW01389_Exidia_glandulosa | -------------------P---D--AS---------------W-VS--SSTANVFVANHDTER-----N-G----NSLSS-----T--------SPSNTYVLATIFSLAHPY-GTPTI |
| 32_EIW55835_Trametes_versicolor | -------------------F---EN-RG---------------W-VP--GTGANVFVVNHDTER-----N-G----NSLNN-----N--------SPSNTYVTAMIFSLAHPY-GTPSI |
| 32_SJL11930_Armillaria_ostoyae | -------------------L---D---R---------------W-IS--GSQANVFVANHDTER-----N-G----NSLNV-----N--------SANNAYVIATIFSLAHPY-GTPTI |
| 32_PAV18085_Pyrrhoderma_noxium | -------------------L---DN-QG---------------W-VS--GSVANVFVANHDTER-----N-G----NSLNS-----N--------SPSNAYTLAMIFSLAHPY-GTPTI |
| 32_RPD65356_Lentinus_tigrinus | -------------------F---DN-LG---------------W-VD--GSVANVFVTNHDTER-----N-G----NSLNN-----N--------SPSNTYVSAMVFSLAHPY-GTPTI |
| 32_PBK84861_Armillaria_gallica | -------------------L---DIGIG---------------W-IS--GSQANVFVANHDTER-----N-G----DSLNV-----N--------SANNAYVIATIFSLAHPY-GTPTI |
| 32_KLO18298_Schizopora_paradoxa | -------------------L---DD-QG---------------W-VP--GDLANVFVADHDTER-----N-Q----GSLNA-----N--------SPSNTYLLAHIFSLAHPF-GTPTV |
| 32_PPR08271_Panaeolus_cyanescens | -------------------L---DD-RG---------------W-VS--GTSANVFVANHDTER-----N-G----GSLRW-----D--------SPSNTYITATVFSLAHPY-GTPTV |
| 32_KIK53845_Gymnopus_luxurians | -------------------L---NN-QG---------------W-IA--SSDANVFVTNHDTER-----D-G----GALTY-----Q--------SPSNEYVTGMIFSLAYPYGNTVTI |
| 32_KIJ56431_Sphaerobolus_stellatus | -------------------L---DN-LG---------------W-VP--GTGANVFVANHDTER-----N-G----NSLNY-----N--------SASNTYVLATIFSLAHPY-GTPSV |
| 32_EJD07843_Fomitiporia_mediterranea | -------------------L---DD-QG---------------W-VP--GDDANVFVTNHDTER-----N-G----NSLND-----N--------SPSNTYTLAMIFSLAHPY-GTPTI |
| 32_EPQ53992_Gloeophyllum_trabeum | -------------------L---DD-QG---------------W-VA--SNKANVFVANHDTER-----N-G----NSLNY-----N--------SGNNAYVLATIFSLAHPY-GTPTV |
| 32_KIP11288_Phlebiopsis_gigantea | -------------------L---DG-LG---------------W-VP--GTSANVFVANHDTER-----G-G----NSLNN-----N--------SPSNLYTLAMILSLAHPY-GTPTI |
| 32_KIY62009_Cylindrobasidium_torrendii | -------------------L---ES-KG---------------W-IA--GSTANVFVANHDTER-----D-G----SSLKY-----D--------SSNNVYVTATIFSLAHPY-GTPTI |
| 32_KIJ69360_Hydnomerulius_pinastri | -------------------L---DD-QG---------------W-VP--GTGANVFVADHDTER-----G-G----SSLNY-----Q--------SPSNTYTLAHVFSLAHPY-GTPTV |
| 32_KZT20455_Neolentinus_lepideus | -------------------L---DD-QG---------------W-VD--SDKANVFVANHDTER-----N-G----NTLNY-----N--------SAHNTYVLATIFSLAHPY-GTPTV |
| 32_EGN97351_Serpula_lacrymans_var_lacrymans | -------------------L---DN-QGMS--------FVLLCW-VP--GTSANVFVADHDTER-----G-G----SSLNY-----N--------SPSNTYTLAHVFSLAHSY-GTPTV |
| 32_KDQ06010_Botryobasidium_botryosum | -----------------------IDSYG---------------W-VS--SAEANVFVANHDTER-----N-G----QSLSY-----K--------SPNNIYTLAHVFMLAWPL-GTPTV |
| 32_PPR00074_Panaeolus_cyanescens | -------------------L---EN-RG---------------W-VS--GPGANVFVDNHDTER-----N-G----ASLSY-----K--------SSNNAYVLATVFKLAYPY-GAPTI |
| 32_EKM53319_Phanerochaete_carnosa | -------------------F---DN-LG---------------W-VP--GTSANVFVSNHDTER-----N-G----NSLNA-----N--------SPSNIYVTAMVFSLAHPY-GMPTI |
| 32_TBU45601_Dichomitus_squalens | -------------------F---DN-LG---------------W-VD--GSVANVFVTNHDTER-----N-G----NSLNN-----N--------SPSNTYVTATIFSLAHPY-GTPTI |
| 32_PSR82077_Phlebia_centrifuga | ------------------------Y-IG---------------W-VS--GTQANVFVTNHDTER-----N-G----NSLNN-----N--------SPSNTYVTAMIFSLAHPF-GTPTI |
| 32_KIM89391_Piloderma_croceum | -------------------L---DN-QG---------------W-VP--GTKANVFVTNHDTERVQILLN-G----NSLNS-----N--------SPSNTYITAMIFSLAHPY-GTPTI |
| 32_PIL31175_Ganoderma_sinense | -------------------F---DN-LG---------------W-VK--GSGANVFVTNHDTER-----N-G----ASLNN-----N--------SPSNTYVSALIFSLAHPY-GTPTI |
| 32_KIY70325_Cylindrobasidium_torrendii | -------------------L---ES-KG---------------W-IS--GSDANVFVANHDTER-----D-G----SSLNS-----N--------SANNVYVTATIFSLAHPY-GSPTV |
| 32_KDQ58060_Jaapia_argillacea | -------------------L---NS-QG---------------W-VD--TSVANVFVTNHDTER-----N-G----NSLNY-----N--------SSSNTYVLATVFSLAHPY-GTPSI |
| 32_CEL62905_Rhizoctonia_solani | -------------------L---EN-RG---------------W-IA--SGGANVFVANHDTER-----N-G----ASLTY-----K--------SG-SIYTLAHVFMLAYPY-GTPTV |
| 32_GAT57350_Mycena_chlorophos | -------------------F---DN-LG---------------F-VP--GSGANVFVTNHDTER-----N-G----LALNY-----Q--------SPSNTYVLAMIFSLAHPY-GTPTI |
| 32_KIM25608_Serendipita_vermifera | -------------------L---ES-RG---------------W-VA--GSSANVFVANHDTER-----G-G----SSLSY-----K--------SSSNTYTLAMVFSLAHSY-GTPSI |
| 32_GAW02849_Lentinula_edodes | -------------------L---SS-QG---------------W-ID--SSDANVFVTNHDTER-----N-G----GSLNY-----E--------SPSNTYTTAMIFSLAYPF-GNVTI |
| 32_TCD63303_Steccherinum_ochraceum | -------------------L---DS-LG---------------W-IP--GDRANVFVANHDTER-----N-G----ASLNE-----N--------SPSNTYVTATIFSLAHPY-GTPTL |
| 32_KZP22139_Fibularhizoctonia_sp_CBS_109695 | -------------------L---DS-QG---------------W-VP--GVDANVFVADHDTER-----G-T----NSLNS-----N--------SPDNTYVTATIFSLGYPY-GTPTV |
| 32_KII90441_Plicaturopsis_crispa | -------------------L---DD-QG---------------W-IA--GDVANVFVADHDTER-----S-G----ASLTY-----S--------SSDNTYTTAHIFSLAHPY-GTPTV |
| 32_KDQ58058_Jaapia_argillacea | -------------------L---NS-QG---------------L-VD--GSVANVFVTNHDTER-----N-G----AALTY-----K--------SSSNTYVLATIFSLAHPY-GTPTI |
| 32_OCH93218_Obba_rivulosa | -------------------F---DNLVG---------------W-VS--GSQANVFVTNHDTER-----A-N----SSLNA-----L--------AGSNAYVNAMIFSLAHPY-GTPTI |
| 32_EMD36390_Gelatoporia_subvermispora | -------------------F---NN-LG---------------W-VN--GSQANVFVTNHDTER-----A-N----SSLNA-----L--------AGSNAYVNAMIFSLAHPY-GTPTI |
| 32_EIW78746_Coniophora_puteana | -------------------L---DN-QG---------------W-VA--GTSANVFVADHDTER-----S-G----ASLNY-----S--------SPSNAYVLAHIFSLAHPY-GTPTV |
| 32_KII86244_Plicaturopsis_crispa | -------------------L---ES-KG---------------W-VS--GSQANVFVTNHDTER-----N-G----DSLNI-----N--------SASNTYVTATIFSLAHPY-GTPTI |
| 32_EKM75205_Agaricus_bisporus | -------------------L---DN-RG---------------W-VA--GNRANVFVANHDTER-----N-G----GSLRY-----D--------SSSNTYITATIFSLAHPY-GTPTI |
| 32_KZT36083_Sistotremastrum_suecicum | -------------------S---INTGG---------------W-VS--SSQANIFVADHDTER-----S-G----GSLNY-----N--------SPSNTYTLAHVFMLAYPY-GTPTV |
| 32_KZS90559_Sistotremastrum_niveocremeum | -------------------S---INTGG---------------W-VS--SSQANIFVADHDTER-----S-G----GSLNY-----N--------SPSNTYTLAHVFMLAYPY-GTPTV |
| 32_EFI98134_Schizophyllum_commune | -------------------L---DS-KG---------------W-VA--GSSANVFVANHDTER-----G-G----DSLNY-----N--------SPNNIYTTAMIFSLAHPY-GTPSI |
| 32_BBC62173_Tricholoma_matsutake | -------------------L---EN-QG---------------W-IP--GVQANVFVANHDTER-----N-G----NSLNI-----N--------SPSNTYILATILSLAHPY-GTPTV |
| 32_CCA69081_Serendipita_indica | -------------------L---GS-AS---------------W-VPNTGYGANVFVANHDTER-----A-G----GSLRY-----D--------SSSNTYTLAMVLSLAHPY-GVPTI |
| 32_EFI98135_Schizophyllum_commune | -------------------L---DG-KG---------------W-VP--ASAANVFVVNHDTER-----Q-G----DSLNS-----K--------SPSNTYITATVFSLAHPY-GMPSV |
| 32_KII85613_Plicaturopsis_crispa | -------------------L---DS-QG---------------W-VP--GKQANVFVANHDTER-----N-G----WSLNY-----N--------SPKNTYITATVFSLAHPY-GNPTV |
| 32_EIN11730_Punctularia_strigosozonata | -------------------L---DN-RG---------------W-VP--GSGANVFVANHDTER-----N-G----NSLNY-----N--------SPDNEYWLAMVFSLAHSY-GTPTI |
| 32_PIL33242_Ganoderma_sinense | -------------------L---DN-RG---------------W-VP--SAQANVLVANHDTER-----N-S----GNLNT-----N--------SPSNIYVLANIFALAHPY-GTPTI |
| 32_KZT18190_Neolentinus_lepideus | -------------------L---DD-RG---------------W-VD--SPVANVFVANHDTER-----N-G----GGLNY-----K--------AANNMYVLATVFSLAHSY-GTPTL |
| 32_KZV75308_Peniophora_sp_CONT | -------------------L---ST-FG---------------W-VG--ASDANVFVANHDTER-----G-G----NTLNA-----N--------SPSNTYVLATVFSLAHPY-GTPTI |
| 32_KII86838_Plicaturopsis_crispa | -------------------L---DS-MG---------------W-VS--GSQANVFVTNHDTER-----N-G----YALNY-----L--------SANNTYITGTVFSLAHPY-GTPTV |
| 32_KDQ08018_Botryobasidium_botryosum | ------------------SL---SPSNG---------------W-VP--SDHANVFVVNHDTER-----N-G----QALTY-----K--------DPNNAYTLAHVFMLAYPF-GTPTV |
| 32_KIY73061_Cylindrobasidium_torrendii | -------------------L---EN-NG---------------W-VS--GSDANVFVANHDTER-----G-G----SSLNY-----K--------SASNTYTTATIFSLAHPY-GTPTI |
| 32_KZW01365_Exidia_glandulosa | -----LV--D--------TI---PGE-S---------------W-LA--SGDANVFVANHDTER-----N-DA--S--LNY-----L--------SPSNTYTLAHVFMLAYPY-GTPSV |
| 32_KDQ58178_Jaapia_argillacea | -----LKG-----------L---EGR-G---------------W-IA--SNDANVFVANHDTER-----N-GG--G--LNY-----K--------AASNMYILATIFSLAYPY-GRPSV |
| 32_KZV75307_Peniophora_sp_CONT | -------------------L---SS-FG---------------W-IS--GLDANVFVANHDTER-----N-G----NSLNH-----N--------SPNNAYVLATIFSLAYPY-GTPSV |
| 32_KIK51212_Gymnopus_luxurians | -------------------L---DD-QG---------------W-ID--SSVANVFVTNHDTER-----Y-G----PSLNY-----T--------SPHNTYVTALIFSLASTYGNTVTI |
| 32_RDX41931_Polyporus_brumalis | -------------------L---DN-RG---------------W-VP--GSQANVFVANHDSER-----N-S----GTLTA-----N--------SPSNIYTLASIFSLAHPY-GTPTV |
| 32_TBU29254_Dichomitus_squalens | -------------------L---DN-RG---------------W-VP--SAKANVFVANHDTER-----D-T----HDLNY-----N--------SPSNTYMLANIFALAHPY-GTPTV |
| 32_KIY45802_Fistulina_hepatica | ----TLDG-----------V---GST-T---------------W-VT--SDSANIFVADQDTER-----N-GG--S--LPP-----S----------HDGYANAHVFMLGFNY-GHPTV |
| 32_KIL59382_Amanita_muscaria | -------------------L---DS-RG---------------W-VP--GNKANVFVANHDTER-----S-EG--LDSLNI-----S--------SPSNTYILATIFSLGYPY-GHPSI |
| 32_KIY53725_Fistulina_hepatica | ----YL-------------N---ETN-G---------------W-VE--SDFANIFVADHDTER-----N-NG--S--LSA-----I--------SDWNTYVNAHVFMLAYPY-GHPTV |
| 32_KIY47132_Fistulina_hepatica | ----SLRD-----------M---GSL-G---------------W-VP--SWDANIFVANHDTER-----S-GT--S--LNF-----S--------SPANAYVLAHVLSLAHPY-GRPTV |
| 32_RPD55342_Lentinus_tigrinus | -------------------L---DN-RG---------------W-VP--GSQANVFVANHDSER-----S-S----GTLTS-----T--------SPSNAYTLASIFSLAHPY-GTPTV |
| 32_TCD61445_Steccherinum_ochraceum | ------------------FT---D--SQ---------------W-TD--SAHANVFVANHDTER-----G-NA--AASLNV-----N--------SPSNTYTLANIFALAHPY-GTPTV |
| 32_KIM25602_Serendipita_vermifera | -------------------L---EN-RG---------------W-IA--GSFANVFVANHDTER-------G----SSLTY-----K--------SPANTYTLAQVFSLAHPYGAKTTI |
| 32_EJD37603_Auricularia_subglabra | -----LLDEG--------VI---PGA-G---------------W-LA--SGDANVFVANHDTER-----N-SE--S--LSI-----L--------SPSDTYKLAHVFMLAHPY-GTPTV |
| 32_KZT54766_Calocera_cornea | -----LMT-----------L---PNQ-D---------------W-VP--SSSANVFVANHDTER-----N-GE--S--LDY-----N--------SPSNTYWLAHVFMLTWPF-GTPTV |
| 32_KIY46805_Fistulina_hepatica | ----DL-------------S---SRS-G---------------W-LP--SGSANVFVANHDTER-----S-GL--S--LDH-----S--------SPSNTYANAHVFSLAYPY-GHPTV |
| 32_PPQ69654_Psilocybe_cyanescens | -------------------F---EN-RG---------------W-VS--GSQANVFVANHDTER-----N-G----ASLTI-----K--------SSSNTYITSHIFSLAHPY-GTPTV |
| 32_KZO93971_Calocera_viscosa | -------------------L---PS-QD---------------W-VS--SSSANVFVANHDTER-----N-G----QSLDY-----N--------SPSNTYYLAHVYMLTWPF-GTPTV |
| 32_KZP07860_Fibularhizoctonia_sp_CBS_109695 | ----TLEN-----------L---NSQ-G---------------W-VD--GSVANVFVATHDTER-----D-GT--S--LIY-----S--------SPDNTYITASVFSLAHSY-GTPTI |
| 32_PVG03687_Serendipita_vermifera | -----LQD-----------F---GNR-G---------------W-LA--GSTANVFVANHDTER-----G--S--S--LSY-----K--------YPSNLYTLASVFSLAHPYGANPTI |
| 32_EIM87674_Stereum_hirsutum | ----PSPM-----------G---SAW-G---------------F-ID--SDVANSIVANQDTER-----S-AT--S--LNY-----E--------SANNAYVLSAIFILSFNYGGAKTV |
| 32_CCA69087_Serendipita_indica | -----LQD-----------F---GNR-G---------------W-LD--SKSANVFVANHDTER-----D--S--S--LNY-----K--------SAMNTYYLATIFSLAHPYGANPTV |
| 32_CEL62035_Rhizoctonia_solani | -----LRG-----------I---ENR-G---------------W-LS--SNSANVFITNHDQEH-----D-GA--V--LSY-----K--------SGSNVYTLAHVFMLSYPY-GTPTI |
| 32_EIN05398_Punctularia_strigosozonata | -------------------I---EK-KF---------------A-VA--SSGASVTVATHDSEH-----G-S----GSLTY-----Q--------SANNSYTLAHVFMLAYPY-GTPTI |
| 32_EJU02896_Dacryopinax_primogenitus | -----LIT-----------L---PLK-D---------------W-VP--SSSANVFVANHDTER-----N-GN--S--LNY-----R--------SPSNMYWLANVFMLTWPF-GTPTV |
| 32_KIO30302_Tulasnella_calospora | -------------------N---PTSQG---------------W-VP--SGEANIFVANHDTER-----S-G----NSIAY-----K--------WDRNTYTLATIFSLAYPY-GTPTI |
| 32_CEL62036_Rhizoctonia_solani | -----LRG-----------I---ENR-G---------------W-LA--SNSANVFVTNHDQER-----G-NT--A--LTY-----K--------SGS-VYTLAHVFMLSYPY-GTPTI |
| 32_KIO24533_Tulasnella_calospora | -----LKG-----------L---KDL-G---------------W-LP--SDKANVFVANHDRER-----S-G---Y--LSY-----K--------SPSKRYTLAHVFSLAYPY-GSPTV |
| 32_EIM84100_Stereum_hirsutum | -----LEN-----------V---EST-----------------SDVT--SSAASVWVANMDSER-----G-NN--GGGLNY-----K--------DG-AIYTLAHVFSLTHNY-GTPSV |
| 32_KDN35973_Rhizoctonia_solani | -----LQD-----------V---RQS-A---------------W-ID--SKGANVFVANHDTER-----N-EQ--S--LSY-----R--------SG-SIYTLAHIFMLAYPY-GTPTI |
| 32_KIY48489_Fistulina_hepatica | -----YEN-----------V---DTI-G---------------WNLT--SSQANTFVVNHDTER-----D-NT--T--L-T-----Y--------RD-PSYTIAHVLMLAHPY-GRPTV |
| 32_KIY48486_Fistulina_hepatica | -----FES-----------V---ESL-G---------------WNLE--SQYANTFIANHDTER-----D-ED--T--L-Y-----Y--------GD-ASYTIAHVLMMAHPY-GTPTV |
| 32_KZP26813_Fibularhizoctonia_sp_CBS_109695 | -----LEN-----------L---NNQ-G---------------W-VA--SSSANVFVADHDTER-----S-SS--S--LNY-----S--------CPDNTYTNAHVFMLGYDY-GTPTV |
| 32_KDQ32476_Pleurotus_ostreatus | -----LKD-----------L----GR-D---------------W-VP--SAHANVFVVNHDKER-----D-GE--A--LNA-----N--------APSNIYILAHIFSLAYPY-GRVSV |
| 32_EIM84087_Stereum_hirsutum | -----LQD-----------I---ENK-V---------------GGLR--SDQANTWVNNMDSER-----G-LD--GGGITY-----K--------EG-KLYALAHVFALTHPF-GTPTV |
| 32_KDQ32475_Pleurotus_ostreatus | -----LKG-----------L---DSR-G---------------W-IP--SASANVFVVNHDNER-----L-DE--S--LNA-----H--------SPSNTYTLGHVFSLAYPY-GHISV |
| 32_EAQ88347_Chaetomium_globosum | ------D-----------YG---ESW-G---------------Y-MS--SSVAAVFVDNHDTER-----N-GQ--T--LNY-----K--------DN-AKYTLATVFMLAYPY-GAPDI |
| 32_ORY89754_Leucosporidium_creatinivorum | ----SLLS-----------W---PDS-S---------------W-VD--SSSAVVFVANHDTER-----TDGT--T--LNW-----A--------SSNNAYALASVFLLAQPY-GQPTI |
| 32_EWZ34052_Fusarium_oxysporum | ------N-----------YG---EGW-G---------------F-MS--SSIAAVFVDNHDTER-----N-GA--T--LSY-----K--------DN-AKYTLANVFMLAYPY-GATDV |
| 32_OBT91518_Pseudogymnoascus_verrucosus | ------N-----------FG---EGW-G---------------Y-LG--SSVAAVFVDNHDTER-----N-GA--T--LNY-----K--------DN-AKYTLASVFMLAHPY-GAPDV |
| 32_KEY73157_Stachybotrys_chartarum | ------N-----------YG---EGW-G---------------Y-MS--SSIAGVFVDNHDTER-----N-GQ--T--LSY-----K--------DD-AKYTLANVFMLAYPY-GAPDI |
| 32_OBT62577_Pseudogymnoascus_sp_23342_1_I1 | ------N-----------YG---EGW-G---------------Y-MS--SGVSAVFVDNHDTER-----N-GA--T--LNY-----K--------DN-AKYTLANVFMLAHPY-GAPDI |
| 32_EON97874_Phaeoacremonium_minimum | ------N-----------YG---EGW-G---------------Y-LG--NSVAAVFVDNHDTER-----N-GA--T--LNY-----K--------DN-AKYTLANVFMLAYPY-GATDI |
| 32_GAT55198_Mycena_chlorophos | ----PVPL-----------N---TPW-D---------------I-VN--STSANVIMANQDTER-----S-GA--S--LTS-----E--------SPNNAYILSAIFLLGFNY-GTPTI |
| 32_OAG04271_Paraphaeosphaeria_sporulosa | ------N-----------YG---EGW-G---------------Y-MS--SSVAGVFVDNHDTER-----N-GE--T--LRY-----K--------DN-AKYTLATVFMLAYPY-GAPDI |
| 32_EGG02960_Melampsora_larici_populina | ----PVG-----------WG---SAWGN---------------GYLA--SEHSTVFVTNHDLER-----S-GG--S--LVA---------------ADPQYLLAHVFILTWSY-GQVDI |
| 32_EGG02993_Melampsora_larici_populina | ----PLA-----------WG---PAWGK---------------GYVS--SDQSTIFVTNHDLER-----N-GG--S--LSP---------------ADPRYLLANVFILTWTY-GQVDI |
| 32_OAV91411_Puccinia_triticina | ----PVP-----------WG---PQWGD---------------GYLP--SSHSQIFVTNWDLER-----N-DN--S--LRI-----D--------QDPGPYLLCQVFILTWGY-GQADV |
| 32_EFP79978_Puccinia_graminis | ----PVP-----------WG---PQWGD---------------GYLP--SSHSQIFVTNWDLER-----N-EH--G--IRI-----D--------KDPGAYLLCQVFILTWGY-GQVDV |
| 32_GAT51088_Mycena_chlorophos | ----PTPM-----------G---AAW-G---------------F-LA--SSSANAIMANQDSER-----NPGE--S--LNY-----E--------AANNAYVLGAIFLLAFNY-GTPTV |
| 32_KNE94052_Puccinia_striiformis | ----PVP-----------WG---PQWGD---------------GYLP--SSQSQIFVTNWDLER-----N-EH--S--IRI-----D--------QHAGPYLLCQVFILTWGY-GQVDV |
| 32_KNZ54553_Puccinia_sorghi | REITPLDNGIVFRRKAYSLGATMGRW-ERRDVEEEEMCLLMIR--MKNNSTHSQIFVTNWDLER-----N--EHGLRM--------D--------QNPGAYVLSQVFILTWGY-GQVDV |
| 42_AOF40721_Microbacterium_aurum | ----------------------------FHAG------MDSDD--VTNDATYNAVYVDSHDYGP-----G--KS---NVRFDGGTAA-----------WAENMSL--MW-TFR-GIPVL |
| 42_CAB06816_Streptomyces_lividans | ----------------------------FHNG------KDSDD--SYNDATYNVVYVDSHDYGP-----N--KS---SERFSGGTDA-----------WAENMSL--MW-TFR-GIPTL |
| 42_AWA81565_Pecoramyces_ruminatium | ----------------------------YNVG------RAEDE--YVNDSTWNVVYVDSHDYGP-----N--MGGNDYSRYSGGTDA-----------WAENLNL--MF-TWR-GIPCI |

| | CSR-VII |
|---|---|
| 1_AAA85446_Paenibacillus_polymyxa | YQGTEQGYSGG----DDP--ANRENM------------------------------------------NF-------NAN--HDLYQYIA-KLNYVRNNHP-AL--------------- |
| 1_CAA49465_Thermoactinomyces_vulgaris | YQGTEQGFSGG----ADP--YNRDDM------------------------------------------VF-------NDK--HELYGYIA-KLNYIRNTHV-AL--------------- |
| 1_BAD06003_Aspergillus_awamori | YAGEEQHYSGG----DVP--YNREATWLS---------------------------------------GY-------DTS--AELYTWIA-TTNAIRKLAI-SA--------------- |
| 1_BAD06002_Aspergillus_awamori | YAGQEQHYAGG----NDP--ANREATWLS---------------------------------------GY-------PTD--SELYKLIA-SANAIRNYAI-SK--------------- |
| 1_AAF14264_Aspergillus_flavus | YAGQEQHYAGG----NDP--ANREATWLS---------------------------------------GY-------PTD--SELYKLIA-SANAIRNYAI-SK--------------- |
| 1_BAD01051_Aspergillus_kawachii | YAGQEQHYAGG----NDP--ANREATWLS---------------------------------------GY-------PTD--SELYKLIA-SANAIRNYAI-SK--------------- |
| 1_BAA22993_Aspergillus_kawachii | YAGEEQHYSGG----DVP--YNREATWLS---------------------------------------GY-------DTS--AELYTWIA-TTNAIRKLAI-SA--------------- |
| 1_EAA64850_Aspergillus_nidulans | YAGQEQHYSGA----GDP--ANREATWLS---------------------------------------GY-------DST--SELYQFIS-KTNQIRNHAI-WQ--------------- |
| 1_AAF17100_Aspergillus_nidulans | YAGQEQHYSGS----NDP--YNREPVWWS---------------------------------------SY-------STS--SELYKFIA-TTNKIRKLAI-SK--------------- |
| 1_P56271_Aspergillus_niger | YAGEEQHYAGG----KVP--YNREATWLS---------------------------------------GY-------DTS--AELYTWIA-TTNAIRKLAI-AA--------------- |
| 1_CAK44871_Aspergillus_niger | YAGQEQHYAGG----NDP--ANREATWLS---------------------------------------GY-------PTD--SELYKLIA-SANAIRNYAI-SK--------------- |
| 1_CAK40249_Aspergillus_niger | YQGQEQHLDGP----GSP--ENREAIWLS---------------------------------------EY-------NTD--AELYKLIG-KLNAIRKHAY-RL--------------- |
| 1_CAK41088_Aspergillus_niger | YQGLEQAIA--------Y--SNRAALWLT---------------------------------------DF-------DTN--ATLYKHIK-KLNAIRKHAI-NL--------------- |
| 1_CAA31218_Aspergillus_oryzae | YAGQEQHYAGG----NDP--ANREATWLS---------------------------------------GY-------PTD--SELYKLIA-SANAIRNYAI-SK--------------- |
| 1_BAA01255_Aspergillus_shirousami | YAGQEQHYAGG----NDP--ANREATWLS---------------------------------------GY-------PTD--SELYKLIA-SANAIRNYAI-SK--------------- |
| 1_AEB80431_Aspergillus_tubingensis | YAGEEQHYSGG----DVP--YNREATWLS---------------------------------------GY-------DTS--AELYTWIA-TTNAIRKLAI-SA--------------- |
| 1_BAA12010_Cryptococcus_sp_S_2 | YYGQEQHLSGS----GVP--LNREALWTS---G-----------------------------------GY-------DTS--SPLYEMIT-TVNQLRTLAI-KQ--------------- |
| 1_ABG48762_Fusicoccum_sp_BCC4124 | YQGQEQHYAGA----SDP--DNREAVWLS---------------------------------------GY-------NAD--ADLYKLTA-SVNQIRNRAI-AV--------------- |
| 1_AAO12212_Lipomyces_kononenkoae | YYGFEQRFDGG----FDP--VNREPMWTS---------------------------------------GY-------NTS--TPLYNYLA-RLNAIRKYAA-SI--------------- |
| 1_AAC49622_Lipomyces_spencermartinsiae | YYGQEQGLNGG----NDP--YNREALWPT---------------------------------------GY-------STT--STFYEYIA-SLNQIRNHAI-YI--------------- |
| 1_AAN75021_Lipomyces_starkeyi | YYGQEQGLNGG----NDP--YNREALWLT---------------------------------------GY-------STT--STFYKYIA-SLNQIRNQAI-YK--------------- |
| 1_AFD54462_Malbranchea_cinnamomea | YAGQEQQYSGD----SDP--YNREALWLS---------------------------------------GF-------NTD--APLYKHIA-ACNRIRSHAV-SN--------------- |
| 1_ABF72529_Ophiostoma_floccosum | YSGQEQHYSGG----ADP--ANREATWLS---------------------------------------GY-------STT--ATLYKHIK-TTNQIRSLII-GK--------------- |
| 1_EPS26265_Penicillium_oxalicum | YAGQEQHYSGG----NDP--ANREATWLS---------------------------------------GY-------SKN--AQLYQHIA-STNKIRSLAI-SK--------------- |
| 1_ABO42285_Phanerochaete_chrysosporium | YYGQEQGFSGN----ADP--YNREPLWTS---------------------------------------KY-------ENS---STYQFVT-KLNTLRNFLV-SS--------------- |
| 1_BAW15173_Pholiota_microspora | YYGQEQYFSGN----ADP--LNREPLWPS---------------------------------------KY-------AKT---DAYNLMA-TLNKFRNFLV-NT--------------- |
| 1_BAF98616_Pichia_burtonii | YYGQEQGLLGA----EDP--ENREALWLA---------------------------------------GY-------DTD--SAYYKFIS-KLNQARNQAA-NK--------------- |
| 1_AGJ52081_Rhizomucor_pusillus | YYGSEQGFSGG----ADP--ANREVLWTA---------------------------------------NY-------DTS--SDLYQFIK-TVNSVRMKSN------------------ |
| 1_ADL28123_Rhizopus_oryzae | YYGTEQSFKGG----NDP--NNREVLWTT---------------------------------------GY-------STT--SDMYKFVT-TLVKARKGSN------------------ |
| 1_ADD80242_Saccharomycopsis_fibuligera | YYGQEQGLSGK----SDP--NNREALWLS---------------------------------------GY-------NKE--SDYYKLIA-KANAARNAAV-YQ--------------- |
| 1_ABS76467_Saitozyma_flava | YYGQEQHLSGS----GVP--LNREALWTS---G-----------------------------------GY-------DSS--SPLYKMIT-TVNQLRTLAI-KQ--------------- |
| 1_CAB11471_Schizosaccharomyces_pombe | FYGQEQMFNGS----HDP--VNRPALWDQ---------------------------------------GY-------NTD--GPLYQYTS-KVNKIRRDLI-NS--------------- |
| 1_CAB40006_Schizosaccharomyces_pombe | FYGQEQGFNGG----EDP--ANRPALWLT---------------------------------------DY-------DQS--NPYYTVIK-TMVAFRKFVI-TQ--------------- |
| 1_CAA34162_Schwanniomyces_occidentalis | YYGQEQGLNGG----SDP--ANREALWLS---------------------------------------GY-------NTD--SEYYELIS-KLNQIRNQAI-KK--------------- |
| 1_CAA51912_Schwanniomyces_occidentalis | YYGQEQGYSGS----SDP--NNREALWLS---------------------------------------GY-------STS--NGYYKLIS-SVNQIRNQAI-YK--------------- |
| 1_ACN82436_Sclerotinia_sclerotiorum | YQGQEQHLTGS----SVP--NNREALWSNS--N-----------------------------------SY-------SQS--ATYYRFIA-SVNQIRNQAI-YV--------------- |
| 1_CAA03110_Thermomyces_lanuginosus | YTGQEQHYAGD----HDP--TNREAVWLS---------------------------------------GY-------NTD--AELYQFIK-KANGIRNLAI-SQ--------------- |
| 1_BAG69580_Trichoderma_viride | YYGSEQAFSGS----NDP--ANREDLWRS---------------------------------------GY-------NTE--TDMYNAIS-KLTFAKHTAG-GL--------------- |
| 1_CAJ21046_Valsaria_rubricosa | YYGTEQGYSGS----SDP--ANREDLWRS---------------------------------------GY-------STT--GDIYTTIA-ALSAARTAAG-GL--------------- |
| 5_AWX66236_Alicyclobacillus_sp_18711 | FYGDYYGIP-------S---QS-------V-------------------------------------------------S---AKSTWLD-KQLSARKSYAYGT--------------- |
| 5_AAQ01675_Alkalimonas_amylolytica | FARDYDEF---------------------------------------------------------------------------GMAATLD-KLIEARRYFAYGP--------------- |
| 5_CAL14744_Anoxybacillus_flavithermus | FYGDYYGIP-------K---YN-------I-------------------------------------------------P---ALKSKLD-PLLIARRDYAYGT--------------- |
| 5_AEW07376_Bacillus_acidicola | FYGDYYGIP-------Q---YN-------I-------------------------------------------------P---SLKSKID-PLLIARRDYAYGT--------------- |
| 5_AAA22191_Bacillus_amyloliquefaciens | FYGDMYGTKGT----SP---KE-------I-------------------------------------------------P---SLKDNIE-PILKARKEYAYGP--------------- |
| 5_ABY86223_Bacillus_cereus | FYGDYYGTKGN----SN---HE-------I-------------------------------------------------P---ALKDKID-PILTARKNFAYGT--------------- |
| 5_CAD26699_Bacillus_halmapalus | FYGDYYGIP-------T---HS-------V-------------------------------------------------P---AMKAKID-PILEARQNFAYGT--------------- |
| 5_AAA22226_Bacillus_licheniformis | FYGDMYGTKGD----SQ---RE-------I-------------------------------------------------P---ALKHKIE-PILKARKQYAYGA--------------- |
| 5_AEM05860_Bacillus_licheniformis | FYGDMYGTKGA----SQ---RE-------I-------------------------------------------------P---ALKHKIE-PILKARKQYAYGA--------------- |
| 5_AAK00598_Bacillus_megaterium | FYGDYYGTKGN----SN---YE-------I-------------------------------------------------P---ALKDKID-PILTARKNYAYGT--------------- |
| 5_AGN35141_Bacillus_paralicheniformis | FYGDMYGTKGA----SQ---RE-------I-------------------------------------------------P---ALKHKIE-PILKARKQYAYGA--------------- |
| 5_AAR68734_Bacillus_sp | FYGDYYGIP-------T---HG-------V-------------------------------------------------P---SMKSKID-PLLQARQTYAYGT--------------- |
| 5_AAA22231_Bacillus_sp_707 | FYGDYYGIP-------T---HG-------V-------------------------------------------------P---AMRSKID-PILEARQKYAYGK--------------- |
| 5_BAF03567_Bacillus_sp_JAMB_204 | FYGDLYGTKGT----SG---RE-------I-------------------------------------------------P---ALKTKIE-PLLKARKDFAYGT--------------- |
| 5_CAC39917_Bacillus_sp_KSM_K38 | FYGDYYGIP-------N---DN-------I-------------------------------------------------S---AKKDMID-ELLDARQNYAYGT--------------- |
| 5_AAB18785_Bacillus_sp_MK_716 | FYGDYYGIP-------Q---YN-------I-------------------------------------------------P---SLKSKID-PLLIARRDYAYGT--------------- |
| 5_AAA63900_Bacillus_sp_TS_23 | FYGDYYGIP-------K---YN-------I-------------------------------------------------P---GLKSKID-PLLIARRDYAYGT--------------- |
| 5_ABW87262_Bacillus_sp_YX_YX1 | FYGDMYGTKGT----SP---KE-------I-------------------------------------------------P---SLKDKLE-PILKARKEYAYGP--------------- |
| 5_AAF00567_Cytophaga_sp | FYGDMYGTKGT----TT---RE-------I-------------------------------------------------P---ALKSKIE-PLLKARKDYAYGT--------------- |
| 5_AAC74994_Escherichia_coli | FYPDLYGAHYE----DVG--GDGQTYPIDM-------------------------------------------------P----IIEQLD-ELILARQRFAHGV--------------- |
| 5_AFZ41193_Exiguobacterium_sp_DAU5 | FYGDYYGTKGT----TN---RE-------I-------------------------------------------------P---NMSGSLQ-PILKARKDFAYGT--------------- |
| 5_AAA22235_Geobacillus_stearothermophilus | FYGDYYGIP-------Q---YN-------I-------------------------------------------------P---SLKSKID-PLLIARRDYAYGT--------------- |
| 5_ABX83871_Geobacillus_thermodenitrificans | YGGDYYGIP-------Q---YN---------------------------------------------------------P---SLKSKID-PLLIARRDYAYGT--------------- |
| 5_AFC87833_Geobacillus_thermoleovorans | FYGDYYGIP-------Q---YN-------I-------------------------------------------------P---SLKSKID-PLLIARRDYAYGT--------------- |
| 5_ACL70573_Halothermothrix_orenii | YWKDYYIW---------------------------------------------------------------------------EMKEGLD-KLLTARRYYAYGP--------------- |
| 5_CAQ30277_Nostoc_sp_PCC_7119 | FHADYYGAEYE----DWG--KDGNRYNIFM-------------------------------------------------P---SHRWIID-KLLYARKHYAYGP--------------- |
| 5_AAA27110_Salmonella_typhimurium | FYPDLYGASYE----DSG--ENGETCRVDM-------------------------------------------------P----VINQLD-RLILARQRFAHGI--------------- |
| 5_BAA24178_Streptococcus_equinus | FYGDYYGISGE----FA---Q-------------------------------------------------------------QDFQDDID-KLLFLRQAAVYGK--------------- |
| 5_AAA97431_Streptococcus_equinus | FYGDYYGISGQ----FA---Q-------------------------------------------------------------ESFQTVID-KLIELRKNAVYGQ--------------- |
| 5_AAN59233_Streptococcus_mutans | FYGDYYGISGE----FA---Q-------------------------------------------------------------ESFQTVLD-KLLYIRQYHVYGS--------------- |
| 5_CCD30600_uncultured_bacterium | YWKDYYIY---------------------------------------------------------------------------NMKNELD-KLLEVRKYFAYGP--------------- |
| 5_ALP73597_Vibrio_alginolyticus | FYADYYGAQYS----DKG--HD-------I-------------------------------------------------N--MVKVPYIE-ELVTLRKDYAYGK--------------- |
| 5_ABK62854_Histoplasma_capsulatum | FYGDLYGVCGG----CKG--IL-------K-------------------------------------------------P---SCNDKLA-DLALARNLYAYGR--------------- |
| 5_ABS11196_Paracoccidioides_brasiliensis | FYGDLHGLCGG----CEG--IL-------R-------------------------------------------------P---SCNGQLA-NLSLARKLYAYGQ--------------- |
| 5_BAW15172_Pholiota_microspora | FYGDLYHNKES----YN-----------------------------------------------------------------ENTARNIS-LLIGARKKFAYGA--------------- |
| 15_AAB60935_Aedes_aegypti | MSSYN--FSDP---------DQGPPQD---------------------TVQVIRSP-GFAVNNSC-STGW--------VC--EHRWPEIR-KMIQFKNFV-AGS--------------- |
| 15_AAB60934_Aedes_aegypti | MSSFF--FDNG---------DQGPPQD---------------------GNGNLIPP-SINADNSC-GNGW--------AC--EHRWRQIY-NMVGFRNAV-RGT--------------- |
| 15_AAA03322_Aedes_atropalpus | MSSFF--FTNG---------DQGPPQD---------------------GGGNLVPP-SINPDNSC-GNGW--------AC--EHRWRQIY-NMVGFRNAV-RGT--------------- |
| 15_AAA03323_Anopheles_merus | MSSFA--FTDF---------DQGPPSD---------------------AQGNLLSP-IINPDKTC-GGGW--------VC--EHRWRQMY-SMIHFRNLA-WGT--------------- |
| 15_AAN77138_Anthonomus_grandis | MSSFY--FDSS---------DQAPPAN---------------------G-DDVLSP-GFNDDGTC-TNGW--------VC--QHRWSPIF-NMVEFRNTV-SGT--------------- |
| 15_AAN77139_Anthonomus_grandis | MSGFE--FDGR---------DSGPPHD---------------------DNFNILSP-VYT-ETGC-SNGW--------TC--EHRWTSVV-GMVQFRNVV-AGT--------------- |
| 15_AAO13755_Asterias_rubens | MSSFS--FNDP---------DSSPPRD---------------------PQGNTLSP-VINPDETC-GNGW--------VC--EHRWRQIK-NMVDFRNVV-VGQ--------------- |
| 15_AAY23288_Blattella_germanica | MSSYS--FSNS---------DQGPPQD---------------------AEGNIISP-SINNDGAC-NNSW--------IC--EHRWRQIY-NMIKFRNIV-TDT--------------- |
| 15_BAB72257_Callosobruchus_chinensis | FSGYS--YDSN---------DQGPPGY---------------------G--DEILG-AEIKDNSC-SNGW--------VC--EHRWSQIY-NMVEFRNVV-QGT--------------- |
| 15_AAA03350_Culex_tarsalis | MSSFY--FDDT---------EQGPPQD---------------------ANGNLIPP-VINERGLC-DNGY--------VC--EHRWRQMF-NMVEFSNVV-RGT--------------- |
| 15_AAF20998_Diabrotica_virgifera_virgifera | MSSYA--FDHR---------DQGPPVK---------------------Q------A-GFNTDNTC-TNGW--------IC--EHRWRQIY-NMVGFRNAV-DGT--------------- |
| 15_AAC35243_Drosophila_ananassae | MSSFA--FENT---------DQGPPTV---------------------DGQTIASP-TFNSDDSC-GGGW--------IC--EHRWRQIY-NMVAFRNSV-GLD--------------- |
| 15_BAB91544_Drosophila_bocki | MSSFS--FSDT---------DQGPPTT---------------------DGHNIASP-SFNSDNSC-SGGW--------VC--EHRWRQIY-NMVAFRNAV-GSD--------------- |
| 15_BAA04582_Drosophila_erecta | MSSFS--FSDT---------DQGPPTT---------------------DGHNIASP-VFNSDHSC-SGGW--------VC--EHRWRQIY-NMVAFRNAV-GSD--------------- |
| 15_BAB91509_Drosophila_kikkawai | MSSFS--FSDT---------DQGPPTT---------------------DGHNIASP-SFNSDNSC-SGGW--------VC--EHRWRQIY-NMVAFRNAV-GSD--------------- |
| 15_BAA95437_Drosophila_leontia | MSSFS--FSDT---------DQGPPTT---------------------DGHNIASP-SFNSDNSC-SGGW--------VC--EHRWRQIY-NMVAFRNAV-GSD--------------- |
| 15_BAA95441_Drosophila_lini | MSSFS--FSDT---------DQGPPTT---------------------DGHNIASP-SFNSDNSC-SGGW--------VC--EHRWRQIY-NMVAFRNAV-GSD--------------- |
| 15_BAA04583_Drosophila_mauritiana | MSSFS--FSDT---------DQGPPTT---------------------DGHNIASP-VFNSDNSC-SGGW--------VC--EHRWRQIY-NMVAFRNTV-GSD--------------- |
| 15_AAK93479_Drosophila_melanogaster_Amyrel | MSSFA--FDDH---------DTPPPQD---------------------AQERIISP-EFDADGAC-VNGW--------IC--EHRWRQIY-AMVGFKNAV-RDT--------------- |
| 15_CAA28238_Drosophila_melanogaster | MSSFS--FTDT---------DQGPPTT---------------------DGHNIASP-IFNSDNSC-SGGW--------VC--EHRWRQIY-NMVAFRNTV-GSD--------------- |
| 15_AAC46839_Drosophila_pseudoobscura | MSSFS--FTDT---------DQGPPTT---------------------DGQNIASP-TFNSDKSC-GGGW--------VC--EHRWRQIY-NMVAFRNAA-GDA--------------- |
| 15_BAA04585_Drosophila_sechellia | MSSFS--FSDT---------DQGPPTT---------------------DGHNIASP-VFNSDNSC-SGGW--------VC--EHRWRQIY-NMVAFRNAV-GLD--------------- |
| 15_AAC46463_Drosophila_virilis | MSSFA--FDDT---------DQGPPTT---------------------DGHNIASP-QFNSDNSC-SGGW--------VC--EHRWRQIY-NMVAFRNAV-GDA--------------- |
| 15_AAG60011_Drosophila_yakuba | MSSFS--FSDT---------DQGPPTT---------------------DGHNIASP-VFNSDNSC-SGGW--------VC--EHRWRQIY-NMVAFRNAV-GSD--------------- |
| 15_CAA76926_Phaedon_cochleariae | MSSFS--FDYN---------DQGPPTQ---------------------G------P-GFNSVRNL--HQW--------VGGANTGWRQILRVMVGFRNAV-DGT--------------- |
| 15_P56634_Tenebrio_molitor | MSSFD--FTDN---------DQGPPQD---------------------GSGNLISP-GINDDNTC-SNGY--------VC--EHRWRQVY-GMVGFRNAV-EGT--------------- |
| 15_AAA03708_Tribolium_castaneum | MSSFA--FDNN---------DQGPPQD---------------------GAGNLISP-SINDDGTC-GNGY--------VC--EHRWRQIF-NMVGFRNAV-QET--------------- |
| 15_AAF73435_Zabrotes_subfasciatus | FSGYY--FNDN---------KQGPPGQ---------------------D--NIC---------AE-GSGW--------VC--EHRWRQIA-NMVGFRNAV-SGT--------------- |
| 24_ABL09312_Acarus_siro | MSSYH--WDQKIEDGKDKNDWIGPPSD---------------------GSGNILSV-TPQPDDTC-NKEW--------IC--EHRWRQIY-NMVHFRNVA-GNE--------------- |
| 24_BAB85635_Anguilla_japonica | MSSFR--WNRHIVDGKDQNDWMGPPSY---------------------PDGSTKPV-LINPDTTC-GDNW--------VC--EHRWRQIR-NMVIFRNVV-NNQ--------------- |
| 24_AAL37207_Crassostrea_gigas | MSSYR--WNRDFHDGRDHNSWMGPPHN---------------------GDMSIKSP-SIQPDMSC-GNGW--------IC--EHRWRQIY-NMVAFRNVV-MGT--------------- |
| 24_AAL37183_Crassostrea_gigas | MSSYH--WNRDFHGGEDHNNWQGPPHN---------------------GDMSIKGP-SIQSDMSC-SNGW--------IC--EHRWRQIY-NMVAFRNVV-MGT--------------- |
| 24_AAD38942_Dermatophagoides_pteronyssinus | MSSYF--WNQIIKDGKDVNDWVGPPSD---------------------KNGNILSV-HPNPDMTC-NHEW--------IC--EHRWREIY-NMVKFRMIA-GQE--------------- |
| 24_AAD38943_Euroglyphus_maynei | MSSYF--WNQIIRDGKDVNDWVGPPTD---------------------QHGNILSV-HPNADLTC-NHEW--------IC--EHRWRQIY-NMVRFKMIA-GQE--------------- |
| 24_AAC60246_Gallus_gallus | MSSYR--WPRYFENGVDVNDWVGPPSN---------------------SDGSTKSV-TINADTTC-GNDW--------VC--EHRWRQIR-NMVIFRNVV-DGQ--------------- |
| 24_ABO26610_Haliotis_discus_discus | MSSYN--FNQAN-------TDQGPPQN---------------------GDMSTKPV-TI-NGMVC-GNGW--------TC--EHRWRQMY-NMVAFRNIA-GYS--------------- |
| 24_BAM74656_Haliotis_discus_hannai | MSSYN--FNQAN-------TDQGPPQN---------------------GDMSTKPV-TI-NGMVC-GNGW--------TC--EHRWRQMY-NMVAFRNIA-GYS--------------- |
| 24_AAA51724_Homo_sapiens_pancreatic | MSSYR--WPRQFQNGNDVNDWVGPPNN----------------------NGVIKEV-TINPDTTC-GNDW--------VC--EHRWRQIR-NMVIFRNVV-DGQ--------------- |
| 24_AAH63129_Homo_sapiens_salivary | MSSYR--WPRYFENGKDVNDWVGPPND----------------------NGVTKEV-TINPDTTC-GNDW--------VC--EHRWRQIR-NMVNFRNVV-DGQ--------------- |
| 24_AAA37221_Mus_musculus | MSSYY--WPRNFQNGKDVNDWVGPPNN----------------------NGKTKEV-SINPDSTC-GNDW--------IC--EHRWRQIR-NMVAFRNVV-NGQ--------------- |
| 24_AAA37230_Mus_musculus | MSSYR--WNRNFQNGKDQNDWIGPPNN----------------------NGVTKEV-TINADTTC-GNDW--------VC--EHRWRQIR-NMVAFRNVV-NGQ--------------- |
| 24_H2N0D4_Oryzias_latipes | MSSYS--WDRNFVNGKDENDWIGPPSN---------------------GDGSTKPV-PINPDQTC-GDGW--------VC--EHRWRQIM-NMVQFRNVV-NGQ--------------- |
| 24_CAA68065_Pecten_maximus | MSSYN--FDRSN-------TDQGPPHN---------------------GDN-INDV-TINADLTC-GNGW--------TC--EHRWREIY-NMVAFRNIV-MGQ--------------- |
| 24_CAA54524_Penaeus_vannamei | MSSYY--WDQWWENGQDKNDWIGPPHD---------------------GSFNIISP-SFNADGSC-GNGW--------IC--EHRWRQIY-NMVEFRNVA-HGT--------------- |
| 24_CAB65552_Penaeus_vannamei | MSSYY--WDQCWENGQDKNDWVGPPHD---------------------DSFNIISP-SFNADGSC-GNGW--------MC--EHRWRQIY-NMVEFRNVA-HGT--------------- |
| 24_AAF65827_Pseudopleuronectes_americanus | MSSFR--WNRHIVNGKDQNDWMGPPSH---------------------SDGSTKSV-PINPDQTC-GDGW--------VC--EHRWRQIT-NMVKFRNVV-DGQ--------------- |
| 24_AAA40725_Rattus_norvegicus | MSSYR--RTRNFQNGKDVNDWIGPPNN----------------------NGVTKEV-TINPDTTC-GNDW--------VC--EHRWRQIR-NMVAFRNVV-NGQ--------------- |
| 24_AAH88228_Rattus_norvegicus | MSSFH--WPRYFENGKDVNDWVGPPNN----------------------NGATKEV-TINSDSTC-GNDW--------VC--EHRWRQIR-NMVAFRNVV-NGQ--------------- |
| 24_P83053_Struthio_camelus | MSSFR--WPRHFENGKDVNDWYGPPSN---------------------SDGSTKEV-TINADSTC-GNDW--------VC--EHRWRQIR-NMVIFRNVV-DGE--------------- |
| 24_AAF02828_Sus_scrofa | MSSYR--WARNFVNGQDVNDWIGPPNN----------------------NGVIKEV-TINADTTC-GNDW--------VC--EHRWRQIR-NMVWFRNVV-DGQ--------------- |
| 24_CAC87125_Tetraodon_nigroviridis | MSSFR--WNRHIVNGKDQNNWMGPPSH---------------------PDGSTKSV-PINPDETC-GDGW--------VC--EHRWRQIK-NMVIFRNVV-NGQ--------------- |
| 32_AAA21016_Aeromonas_hydrophila | MSAYR--FENM-----------GTYET---------------------DKGA-------PGSTPCTDSQW--------NC--EQRRPTIM-NMVLFRNRT-EGQ--------------- |
| 32_BAA22082_Bacillus_sp_195 | HSGYS--FSNN---------DAGPALA---------------------GNGEVIDP-VCGQ------NGW--------TC--KHAQTGIE-NMVGFRTQT-YGT--------------- |
| 32_AAY89038_Bifidobacterium_breve | YSGYY--FDNS---------DDGAPGA---------------------TETSVPDM-ECPTDGSMTSGTW--------QC--AERWTAIR-GMIGFHNAV-NGT--------------- |
| 32_CAB92963_Halomonas_meridiana | MSSYA--FSN-S--------DQGPPQA---------------------PVYQ-------QGEAQC-GEAW--------VC--EHRWPEIA-NMVAFRQQA-EGA--------------- |
| 32_BAJ52728_Kocuria_varians | FSGYS--VDDY---------DAGPPTD---------------------ADGRVVDA-VCGE------GPW--------TC--THARTEVT-GMVGFRDAV-GDT--------------- |
| 32_AKG25402_Microbacterium_aurum | HSGYE--FSND---------KQAPVVN---------------------GQGRVVDP-QPGQ------NGW--------TF--EHAETGIK-NMVGFRVTT-YGT--------------- |
| 32_CAA41481_Pseudoalteromonas_haloplanktis | MSSYD--FHGDT--------DAGGPNV---------------------PVHN-------NGNLECFASNW--------KC--EHRWSYIA-GGVDFRNNTADNW--------------- |
| 32_AAA86835_Pseudomonas_sp_KFCC10818 | MSGYY--FND-F--------DAGPPAT---------------------GIHT-------GNACGFDGGDW--------VC--EHRWRGVA-NMVAFRNHTAAEW--------------- |
| 32_AAA96317_Streptomyces_albus | NSGYE--WSDP---------DAGPPDG---------------------GHV----D-ACWQ------NGW--------KC--QHKWPEIA-SMVAFRNAT-RGE--------------- |

| | CSR-VII |
|---|---|
| 32_BAC73693_Streptomyces_avermitilis | NSGYE--WSDA---------DAGPPGG---------------------GTV----N-ACWQ------DGW--------KC--QHAWPEIK-AMVAFRNAT-RGE---------------- |
| 32_CAA40798_Streptomyces_griseus | HSGYE--FTDH---------DAGPPNG---------------------GTV----N-ACYS------DGW--------KC--QHAWPELS-SMVGLRNTA-SGQ---------------- |
| 32_AAA26698_Streptomyces_hygroscopicus | YSGYE--WTDK---------DAA-AGG---------------------------------S------TGW--------TD--DAAKREIT-GMVGFRNAV-GSA---------------- |
| 32_AAA88554_Streptomyces_limosus | HSGYE--FTDH---------DAGPPNG---------------------GTV----N-ACYS------DGW--------KC--QHAWPELS-SMVGLRNTA-SGQ---------------- |
| 32_CAB06622_Streptomyces_lividans | HSGYE--FTDH---------DAGPPNG---------------------GQV----N-ACYS------DGW--------KC--QHAWREIS-SMVAFRNTA-RGQ---------------- |
| 32_CAA73775_Streptomyces_sp_TO1 | NSGYE--WSDA---------AAGPPN----------------------ATV----N-ACWQ------DGW--------KC--QHKWPEII-RMVGFRNAT-RGQ---------------- |
| 32_AAA82875_Streptomyces_sp_WL6 | YSGYE--WSDA---------DAGPPSG---------------------------------A------DGW--------TD--THAQQTIT-GLVGFRNAV-GSA---------------- |
| 32_AAA26697_Streptomyces_thermoviolaceus | NSGYE--WSDP---------DARPPDG---------------------GHV----D-ACWQ------NGW--------KC--QHKWPEIA-SMVAFRNAT-RGE---------------- |
| 32_AAB36561_Streptomyces_venezuelae | HSGYE--WTDK---------DAGPPNN---------------------GQV----N-ACYT------DGW--------KC--QHAWREIS-SMVAFRNTA-RGQ---------------- |
| 32_ABF13430_Thermobifida_fusca | MSSYT--WSGD--------DKAGPPMH---------------------SDGTTRPT-DCSA------DRW--------LC--EHR--AVA-GMVGFHNAV-AGQ---------------- |
| 32_CAA41881_Thermomonospora_curvata | MSSYT--WSGD--------DKAGPPMH---------------------SDGTTRPT-DCSA------DRW--------LC--EHR--AVA-GMVGFHNAV-AGQ---------------- |
| 32_BAV60949_Pholiota_microspora | LSSYS--Y-ST--------IDDGAPNS---------------------GAGTCSTN-GGS-------GGW--------HC--QHRLPAIA-GMVGFRNNV-G------------------ |
| 32_KJA26527_Hypholoma_sublateritium | LSSYS--F-VT--------TDDGAPNG---------------------GVGTCSGN-AGT-------GGW--------YC--QHRFVPFV-GMTGFRNNV-G------------------ |
| 32_KDR78015_Galerina_marginata | LSSYS--F-ST--------TDDGAPNG---------------------GVGICSAT-GGS-------GGW--------LC--QHRLVAIS-GMVGFRNNV-G------------------ |
| 32_PPQ83160_Psilocybe_cyanescens | LSSYS--F-ST--------TDDGAPNG---------------------GTGACTAT-GGS-------GGW--------LC--QHRFVAIS-GMVGFRNNV-G------------------ |
| 32_KTB32361_Moniliophthora_roreri | LSSYS--F-SN--------IDDGSPNG---------------------GAGTCTAT-GGS-------NGW--------LC--QHRWTAIS-GMVGFRNTV-G------------------ |
| 32_KDQ30003_Pleurotus_ostreatus | LSSYS-GF-TN--------TDAGAPNG---------------------GVGTCSGT-GGA-------NGW--------FC--QHRWVAFS-GMVGFRNTV-G------------------ |
| 32_RDX41646_Polyporus_brumalis | LSSYS-GF-TN--------TDAGAPNG---------------------GVGTCSGS-GGS-------NGW--------FC--QHRWDAIA-GMVGFRNNV-G------------------ |
| 32_OJT13448_Trametes_pubescens | LSSYD-GF-TN--------TDLGAPNG---------------------GTGTCSGS-GGT-------NGW--------LC--QHRWTAVA-GMVGFRNQV-G------------------ |
| 32_PFH45132_Amanita_thiersii | LSSYH--F-ST--------TDDGAPNG---------------------GSGTCSTT-GGS-------GNW--------LC--QHRFIAIT-GMVGFRNTV-G------------------ |
| 32_KIM37787_Hebeloma_cylindrosporum | LSSYS--F-SS--------YDTGAPNG---------------------GAGACTSN-GGS-------GGW--------LC--QHRHVAIS-GMVGFRNNV-G------------------ |
| 32_PBK61492_Armillaria_solidipes | LSSYQ--Y-ST--------TDDGAPNN---------------------GVGTCSGT-GGA-------NGW--------YC--QHRWIAFT-GMVGFRNNV-G------------------ |
| 32_ETW76368_Heterobasidion_irregulare | LSSYS-GF-SD--------TDVGAPNS---------------------GVGTCSGS-TGT-------NGW--------LC--QHRWTGIA-GMVGFRNNV-S------------------ |
| 32_RDB27268_Hypsizygus_marmoreus | LSSFT--F-SN--------KDAGPPNG---------------------GSGTCSST-GGS-------GGW--------LC--QHRYIPIS-GMVGFRNNV-G------------------ |
| 32_PPQ71147_Gymnopilus_dilepis | LSSYS--F-SG--------YDDGAPNN--GKYLQYYKGNHTHDH-RNTGAGTCSAT-GGS-------SGW--------LC--QHRYVAIS-GMVGFRNNV-G------------------ |
| 32_OSD07916_Trametes_coccinea | LSSYS-GF-TN--------TDAGAPNG---------------------GTGTCSGS-GGE-------NGW--------LC--QHRWTAVA-GMVGFRNNV-G------------------ |
| 32_KZW01389_Exidia_glandulosa | LSSYS--F-SN--------TDAGAPNN---------------------GVGTCSGS-GGA-------NGW--------LC--QHRWPAFA-GLVAFRNAV-G------------------ |
| 32_EIW55835_Trametes_versicolor | LSSYT-GF-TN--------TDLGAPNG---------------------GTGTCSGS-GGT-------NGW--------LC--QHRWTAVA-GMVGFRNQV-G------------------ |
| 32_SJL11930_Armillaria_ostoyae | LSSYQ--Y-ST--------TDDGAPNN---------------------GVGTCSGT-GGA-------NGW--------YC--QHRWIAFT-GMVEFRNNV-G------------------ |
| 32_PAV18085_Pyrrhoderma_noxium | LSSYS--F-SD--------TDAGSPNS---------------------GTGSCSTD-GGS-------NGW--------LC--QHRWPAVV-GMLGFRNTV-RGT---------------- |
| 32_RPD65356_Lentinus_tigrinus | LSSYD-GF-TN--------TDAGAPNG---------------------GTGTCSGS-GGS-------NGW--------LC--QHRWTAIA-GMVGFRNNV-G------------------ |
| 32_PBK84861_Armillaria_gallica | LSSYQ--Y-LT--------TDDGAPNN---------------------GVGTCLGT-GGA-------NGW--------YC--QHRWIAFT-GMVEFRNNV-G------------------ |
| 32_KLO18298_Schizopora_paradoxa | LSSYV-GF-TD--------SDLGAPNG---------------------NSGTCSEN-GGS-------DGW--------IC--QHRWTAIV-GMVGFRNEV-G------------------ |
| 32_PPR08271_Panaeolus_cyanescens | ISSYS--F-TD--------TDAGAPNR----------------------AGTCSGN-GGS-------NGW--------LC--QHRMLAIS-GMVGFRNAV-G------------------ |
| 32_KIK53845_Gymnopus_luxurians | LSSYQ--F-SS--------FDDGAPDG---------------------GAGECSGT-GGT-------GGW--------LC--QHRWIAVS-GMVGFRNHV-G------------------ |
| 32_KIJ56431_Sphaerobolus_stellatus | LSSYT--F-SD--------TDDGSPNG---------------------GTGTCSAT-GGT-------NGW--------LC--QHRWPAFV-GMVTFRNSV-S------------------ |
| 32_EJD07843_Fomitiporia_mediterranea | LSSYS-GF-TN--------TDAGAPNG---------------------GAGTCSTD-GGA-------NGW--------LC--QHRWPAIT-GMVGFRNNI-SVA---------------- |
| 32_EPQ53992_Gloeophyllum_trabeum | LSSYS--F-ST--------TDDGSPNS---------------------GAGTCSGT-GGA-------NGW--------LC--QHRWIAFS-GMTGFRNNV-G------------------ |
| 32_KIP11288_Phlebiopsis_gigantea | LSSYS-GF-TD--------YDAGAPNS---------------------GAGTCTTG-GGS-------NGW--------LC--QHRWNAVA-GMTGFHNTV-G------------------ |
| 32_KIY62009_Cylindrobasidium_torrendii | LSGFS--F-TN--------KDDGSPNG---------------------GVGTCSGT-GGT-------NGW--------LC--QHRWVAFS-GMVGFRNNA-K------------------ |
| 32_KIJ69360_Hydnomerulius_pinastri | LSSYE--F-ST--------YDQGAPNG---------------------GAGTCSGN-TGE-------NGW--------LC--QHRWSAIA-GMVGFRNNV-G------------------ |
| 32_KZT20455_Neolentinus_lepideus | LSSYE--F-ST--------TDDGAPNS---------------------GTGTCSGT-GGE-------NGW--------LC--QHRWVAIS-GMVGFRNNA-G------------------ |
| 32_EGN97351_Serpula_lacrymans_var_lacrymans | LSSYQ--F-SS--------YDDGAPNG---------------------GNGTCYGA-GGV-------NGW--------LC--QHRWSAIA-GMVGFHNNV-G------------------ |
| 32_KDQ06010_Botryobasidium_botryosum | LSGYQ--F-NG--------VDDGAPNS---------------------GKGQCSGT-GGS-------GSW--------LC--QHRWPAIA-GLVGLRNKA-Y------------------ |
| 32_PPR00074_Panaeolus_cyanescens | LSSFS--F-SD--------HDAGAPNG---------------------GYGRCSAT-GGS-------DGW--------VC--QHRWIAIS-GMVGFRNTV-G------------------ |
| 32_EKM53319_Phanerochaete_carnosa | LSSYS-DF-TD--------YDAGAPNG---------------------GAGTCTTG-GGT-------NGW--------LC--QHRWNAIA-GMVGFHNQV-G------------------ |
| 32_TBU45601_Dichomitus_squalens | LSSYD-GF-TN--------TDAGAPNG---------------------GTGTCSGT-GGE-------NGW--------LC--QHRWVAFT-GMVGFRNNV-G------------------ |
| 32_PSR82077_Phlebia_centrifuga | LSSYT-GF-TD--------TDAGAPNG---------------------GAGTCTGT-GGT-------NGW--------LC--QHRWVAIS-GMVGFHNNV-G------------------ |
| 32_KIM89391_Piloderma_croceum | LSSYY-GF-TD--------TDAGAPNG---------------------GVGTCTGT-CGR-------NGW--------LC--QHRWTAIS-GMTGFRNTV-G------------------ |
| 32_PIL31175_Ganoderma_sinense | LSSYS-GF-TD--------TSVGAPNG---------------------GVGTCIGS-GGK-------NGW--------LC--QHRWIAVA-GMVGFRNNV-G------------------ |
| 32_KIY70325_Cylindrobasidium_torrendii | LSSFS--F-SS--------SDDGAPNG---------------------GVGTCSGT-GGA-------NGW--------LC--QHRWTAFS-GMVGFRNNA-G------------------ |
| 32_KDQ58060_Jaapia_argillacea | LSSYT-FESDN--------TDQGAPNG---------------------GAGTCYGT-GGV-------NGW--------LC--QHRWIAFS-GMVGFRNNV-G------------------ |
| 32_CEL62905_Rhizoctonia_solani | LSSYT--F-SN--------NDAGSPSN---------------------GAGSCSGS-GGA-------NGW--------QC--QHRWSAVA-GMVKWRNGV-T------------------ |
| 32_GAT57350_Mycena_chlorophos | LSSYS--F-ST--------TDDGAPNN---------------------GVGTCSAT-GGS-------GGW--------LC--QHRILSVA-GMVGFRNVV-G------------------ |
| 32_KIM25608_Serendipita_vermifera | LSGYS--F-SD--------NDAGAPNS---------------------GAGTCSGS-TGS-------NGW--------LC--QHRWPAVA-GMVGFHNVA-G------------------ |
| 32_GAW02849_Lentinula_edodes | LSSYE--F-AT--------TDDGAPNG---------------------GTGECSGT-GGT-------DGW--------IC--QHRWVAVS-GMVGFKNNV-G------------------ |
| 32_TCD63303_Steccherinum_ochraceum | LSSYS-GF-ED--------SDAGAPNG---------------------GTGTCAGT-GGT-------NGW--------FC--QHRWIAFS-GMVGFRNAV-G------------------ |
| 32_KZP22139_Fibularhizoctonia_sp_CBS_109695 | LSGYT-I--TT--------YDAGGPNG---------------------GAGTCSGT-GGT-------NGW--------LC--QHRWTAFS-GMTGFHNNV-G------------------ |
| 32_KII90441_Plicaturopsis_crispa | LSSYE--F-SG--------YDDGAPES---------------------NAGACSGN-GGT-------DGW--------LC--QHRWIAIS-GMVGFRNNA-G------------------ |
| 32_KDQ58058_Jaapia_argillacea | LSSFE-FPDDN--------TDQGSPNG---------------------GSGTCSGT-GGA-------NGW--------LC--QHRWIAFS-GMVGFRNNV-S------------------ |
| 32_OCH93218_Obba_rivulosa | LSSYQ-GF-TD--------YDAGAPNG---------------------GAGTCSGS-GGT-------NGW--------MC--QHRWTGVA-GMVGFHNNV-G------------------ |
| 32_EMD36390_Gelatoporia_subvermispora | LSSYE-GF-TD--------YDAGAPNS---------------------GAGTCSGS-GGT-------NGW--------LC--QHRWTGVA-GMVGFHNNV-G------------------ |
| 32_EIW78746_Coniophora_puteana | LSSFE--I-TD--------FDQGAPDN---------------------GTGTCTAS-GGQ-------DGW--------LC--QHRWPAIA-GMVGFRASA-G------------------ |
| 32_KII86244_Plicaturopsis_crispa | LSSYS--F-SD--------QDAGAPNG---------------------GAGTCSGT-GGS-------NGW--------LC--QHRWVAFT-GMVGFHNNV-G------------------ |
| 32_EKM75205_Agaricus_bisporus | ISSYN--I-PN--------NDAGAPNG---------------------GSGSCSTT-GGA-------NGW--------LC--QHRFVAIT-GMVGFRNNV-G------------------ |
| 32_KZT36083_Sistotremastrum_suecicum | LSGYT--F-SN--------TDQGSPNN---------------------GSGTCYGA-GGT-------NGW--------LC--QHRWSAIA-GLVGFRNHV-GL----------------- |
| 32_KZS90559_Sistotremastrum_niveocremeum | LSGYT--F-SN--------TDQGSPNN---------------------GTGTCYGA-GGT-------NGW--------LC--QHRWSAIA-GLVGFRNYV-GP----------------- |
| 32_EFI98134_Schizophyllum_commune | LSSFE--F-SN--------SDDGAPNG---------------------NAGTCSDD-GGA-------SGW--------IC--QHRWPAVR-NMVAFRNEV-G------------------ |
| 32_BBC62173_Tricholoma_matsutake | LSSYS--F-SN--------TDDGAPDG---------------------GASCILLS-SNG-------GGW--------LC--QHRFVAIK-GMVGFRNSV-G------------------ |
| 32_CCA69081_Serendipita_indica | LSSYS--F-SN--------NDNGAPNS---------------------NYGTCSGS-TGS-------NGW--------LC--QHRWPAVS-GLVGFHNTV-G------------------ |
| 32_EFI98135_Schizophyllum_commune | LSSYE--F-SD--------HDAGAPGN---------------------GGGTCSQK-GGA-------SGW--------LC--QHRYPAIS-GMVGFRNMV-G------------------ |
| 32_KII85613_Plicaturopsis_crispa | LSSYS--F-SK--------IDDPAPNN---------------------GAGTCAST-GGS-------GGW--------LC--QHRLPAVA-GMVGFRNTV-G------------------ |
| 32_EIN11730_Punctularia_strigosozonata | LSSYS--Y-ST--------TDDGAPNG---------------------GVGTCSGT-AGT-------NGW--------LC--QHRWV--G-GMVGFHNNV-G------------------ |
| 32_PIL33242_Ganoderma_sinense | LSGYS--F-SL------SNTNAGAPNG---------------------GAGTCSGN-GGA-------NGW--------IC--QHRWQAIA-GMVGFRNAV-G------------------ |
| 32_KZT18190_Neolentinus_lepideus | LSSYD--F-SD--------QDAGAPNG---------------------GAGTCSTS-GGA-------NGW--------LC--QHRWTAFT-GMVGFRNVV-G------------------ |
| 32_KZV75308_Peniophora_sp_CONT | LSSYS--F-SN--------SDDGAPNG---------------------GAGTCSGN-GGQ-------NGW--------LC--QHRWAATS-GMVKWRNAV-G------------------ |
| 32_KII86838_Plicaturopsis_crispa | LSSYS--F-TD--------IDVGAPNN---------------------GTGTCATA-GGS-------GGW--------LC--QHRYPAMA-GMVGFRNAV-G------------------ |
| 32_KDQ08018_Botryobasidium_botryosum | LSSYQ--F-GG--------NDDGSPNN---------------------GNGQCSGT-GGT-------NGW--------LC--QHRWPAIA-GLVGLRNKA-F------------------ |
| 32_KIY73061_Cylindrobasidium_torrendii | LSSYE--F-SN--------SDDGAPNS---------------------GAGTCSGA-GGE-------NGW--------LC--QHRWTAVA-GMVGFRNEV-G------------------ |
| 32_KZW01365_Exidia_glandulosa | LSSYE--Y-AG--------YDDGAPNS---------------------ATGTCFGE-G-GV------NSW--------LC--QHRFPPIA-GLTAFRGAV-S-G---------------- |
| 32_KDQ58178_Jaapia_argillacea | LSGYD--F-QN--------IDVGSPNN---------------------GAGTCSNN-G-GA------AGW--------LC--QHRWTAFA-GMVGWRNKA-GKA---------------- |
| 32_KZV75307_Peniophora_sp_CONT | LSSYT--Y-ST--------TDDGAPNG---------------------GTGTCSGT-GGE-------NGW--------LC--QHRWVAFS-GLVKWRNAV-G------------------ |
| 32_KIK51212_Gymnopus_luxurians | LSSYS--F-SD--------INAGAPNE-----------------------GNCSGT-GGSG------DGW--------IC--QHRWVAIT-GMTAFRNNV-G------------------ |
| 32_RDX41931_Polyporus_brumalis | LSSYS--F-SN--------ADAGPPNG---------------------GAGNCNQ----G-------SGF--------IC--QHRWQEIV-GMVKFRNTV-G------------------ |
| 32_TBU29254_Dichomitus_squalens | LSSYS--F-SS--------GDTGAPNG---------------------GTGVCSGN-GGS-------NGW--------LC--QHRWQAIS-GMVGFRNTV-G------------------ |
| 32_KIY45802_Fistulina_hepatica | MSSYS--Y-SS--------GDDGAPNN---------------------GYGTCEGA--NGT------NGF--------YC--QHRWTQVT-GMAGFRSNV-GD----------------- |
| 32_KIL59382_Amanita_muscaria | LSSYR--F-AD--------KGEGAPNG---------------------GAGACSGN-GGS-------GSW--------LC--QHRFTPVS-GMVGFRNNV-G------------------ |
| 32_KIY53725_Fistulina_hepatica | LSSYQ--F-NN--------TDEGAPNN---------------------GTGTCNGT--TGL------NGW--------YC--QHRWTEIT-GMVAFREIV-GD----------------- |
| 32_KIY47132_Fistulina_hepatica | LSSYA--F-DT--------TDDGAPMD---------------------GQGTCQVT--SGA------NRW--------LC--QHRWPAIA-GMVGFRNNV-QN----------------- |
| 32_RPD55342_Lentinus_tigrinus | LSSYS--F-SN--------ADAGPPNG---------------------GSGNCNQ----G-------SGF--------IC--QHRWQEIA-GMVKFRNTV-G------------------ |
| 32_TCD61445_Steccherinum_ochraceum | LSSYT--Y-SS--------VDDGAPNG---------------------GTGSCYGA-GGV-------NGW--------LC--QHRWTAIA-GMTGFRNTV-G------------------ |
| 32_KIM25602_Serendipita_vermifera | LSSYL--F-EG--------HEDGAPNS---------------------LHGTCTGN-TGA-------NGW--------LC--QHRWSPIA-GLVGFRNAV-G------------------ |
| 32_EJD37603_Auricularia_subglabra | LSSYE--F-ND--------TDAGAPNG---------------------AVGSCFGE-G-GV------NGW--------LC--QHRFPPIA-GLTAFRFAV-S-G---------------- |
| 32_KZT54766_Calocera_cornea | LSSYQ--F-SD--------SDAGAPAN---------------------GEGSCSGT-G-GT------NGW--------LC--QHRNPIIA-NLLPLHNDF-TSS---------------- |
| 32_KIY46805_Fistulina_hepatica | LSGYA--F-SS--------FDDDAPTG---------------------GAGMCSAH--GGM------SGW--------LC--QHRWPQMA-GMVGFRKNV-GD----------------- |
| 32_PPQ69654_Psilocybe_cyanescens | LSSYS--F-SG--------HDDGAPNG---------------------GVGACSAT-GGS-------V---------------------------------------------------- |
| 32_KZO93971_Calocera_viscosa | LSSYQ--F-SD--------SDAGAPVN---------------------GAGSCSGS-GGS-------NGW--------LC--QHRNPVIA-NLLPLHNAF-T------------------ |
| 32_KZP07860_Fibularhizoctonia_sp_CBS_109695 | LSSYS--F-SD--------TNAGAPNS---------------------NTGVCSGS--GGL------DGW--------NC--QHRYVAIT-GMVGFHNTA-SA----------------- |
| 32_PVG03687_Serendipita_vermifera | LSSYM--F-DG--------RDDGAPNN---------------------WQGTCTGN-T-GA------NGW--------LC--QHRWSPIA-GMVGFRNTV-GSA---------------- |
| 32_EIM87674_Stereum_hirsutum | YSGYN--F-TS--------YDQGAPQD---------------------SNGNTNAV-TCDS------NGW--------RC--EHRWQAIA-NMVG------------------------- |
| 32_CCA69087_Serendipita_indica | LSSYF--F-EA--------RDDGAPNN---------------------WQGACQGI-S-GD------KGW--------LC--QHRWPGIS-GLVGFRNTV-GDS---------------- |
| 32_CEL62035_Rhizoctonia_solani | LSSYT--F-ND--------KNEGAPNG---------------------AYGTCYGA-G-GV------NGW--------LC--QHRWTPIA-GMIGFYNQV-GTA---------------- |
| 32_EIN05398_Punctularia_strigosozonata | LSSYT--F-DN--------TDAGGPNG---------------------NYGTCYGS-GGV-------NGW--------LC--QHRWTPIA-GMAAFRNTV-G------------------ |
| 32_EJU02896_Dacryopinax_primogenitus | LSSYD--F-TD--------HDAGAPNN---------------------GAGTCSGT-G-GS------NGWQILTVCIRLC--QHRSPVIA-NLLPLHNAL-IKP---------------- |
| 32_KIO30302_Tulasnella_calospora | LSGYY--F-TN--------NDDGAPND---------------------GAGSCND-----G------SGW--------IC--QHRWSAVT-GMVKFFNQV-K------------------ |
| 32_CEL62036_Rhizoctonia_solani | LSSYE--F-DN--------GNSGAPNG---------------------AYGTCSGA-G-GA------GGW--------LC--QHRWNAIA-GMIGFYNQV-GTA---------------- |
| 32_KIO24533_Tulasnella_calospora | FSGYS--F-AN--------SDDGAPNG---------------------NYGTCNDN-GSGA------NGW--------QC--QHRWTAIA-GMVSFYNNV-GSN---------------- |
| 32_EIM84100_Stereum_hirsutum | LSSYE--F-SD--------NDAGAPNS---------------------GAGSCSGN-N-GT------NGW--------LC--QHRWTAIA-NLVKFRTAA-GST---------------- |
| 32_KDN35973_Rhizoctonia_solani | LSSYT--F-SD--------YDIGAPIN---------------------GTGACAGS-K-GA------NGW--------LC--QHRWAAIQ-GMVKFRNGI-QSL---------------- |
| 32_KIY48489_Fistulina_hepatica | LSSYT--F-DD--------TNAGPPNN---------------------GTGECTGN-G-GE------DDW--------LC--QHRYQAIA-GMVPWRLAA-GNE---------------- |
| 32_KIY48486_Fistulina_hepatica | LSSYS--F-TD--------INAGPPNN---------------------GNGTCSGN-G-GS------GDW--------LC--QHRYMAIA-NMVPWRAAV-GSE---------------- |
| 32_KZP26813_Fibularhizoctonia_sp_CBS_109695 | LSGYK--F-SG--------YDDGAPNN---------------------GTGTCTGN--TGT------GGW--------IC--QHRWTPVI-GMVGFHNNA-GT----------------- |
| 32_KDQ32476_Pleurotus_ostreatus | LSSYEGGL-GD--------KDVGGPNQ---------------------GNAKCSGSARNGN------GGW--------MC--QHRWVAIA-GMTRFRNAV-QD----------------- |
| 32_EIM84087_Stereum_hirsutum | LSSYE--F-ND--------VNAGAPNG---------------------GTGTCDGN-A-GS------NGW--------IC--QHRWTAIS-NLVKFRSAT-SGA---------------- |
| 32_KDQ32475_Pleurotus_ostreatus | LSSYESGI-DN--------HDPGPPNG---------------------GEW---------P------NRW--------LC--QHRWTAIA-GMTRFRNAV-RN----------------- |
| 32_EAQ88347_Chaetomium_globosum | NSGYE--FSDS---------DAGPPNN---------------------GVV----D-ACWQ------DSW--------KC--QHAWSEVL-GMVAFRNAV-RGQ---------------- |
| 32_ORY89754_Leucosporidium_creatinivorum | HSGYN--F-TS--------FGTGAPLD---------------------SDDNALPV-TCFS------DNW--------RC--EQRWDAIS-GMVKFHNAV-GD----------------- |
| 32_EWZ34052_Fusarium_oxysporum | HSGYE--FSDK---------NAGPPNN---------------------GAV----N-ACWQ------DGW--------KC--QHAWPEII-RMVAFRNAV-RGQ---------------- |
| 32_OBT91518_Pseudogymnoascus_verrucosus | HSGYE--FSDN---------DAGPPNN---------------------GAV----S-ACWQ------NGW--------KC--QHAWAEIM-RMVAFRNAV-RGQ---------------- |
| 32_KEY73157_Stachybotrys_chartarum | NSGYE--FSDT---------DAGPPNN---------------------GVV----S-ACWQ------DSW--------KC--QHAWPEIM-RMVAFRNAV-RGQ---------------- |
| 32_OBT62577_Pseudogymnoascus_sp_23342_1_I1 | NSGYE--FTDV---------DAGPPNG---------------------GAV----T-ACWQ------DGW--------KC--QHAWAEIS-SMVAFRNAV-RGE---------------- |
| 32_EON97874_Phaeoacremonium_minimum | HSGYE--FSDT---------DAGPPNN---------------------GAV----S-ACWQ------DGW--------KC--QHAWPEIL-RMVAFRNAV-RGQ---------------- |
| 32_GAT55198_Mycena_chlorophos | FSGYN--FSGD--------FNLGAPQN---------------------ASGFTEAI-TCFQ------NGF--------RC--EHRFIAIA-NMNLFHNAV-EG----------------- |
| 32_OAG04271_Paraphaeosphaeria_sporulosa | KSGYE--FSDT---------DAGPPYD---------------------GAV----N-ACCQ------DSW--------KC--QHAWPEIM-RMVAFRNVV-RGQ---------------- |
| 32_EGG02960_Melampsora_larici_populina | LSGYN--F-TN--------FDDGPSQF----------------------------RVHCGQ------FGW--------RC--EHRHPMIV-GAIQIRGRS-GSQ---------------- |
| 32_EGG02993_Melampsora_larici_populina | LSGYN--F-TE--------TGDGPRQL----------------------------RVECGQ------FGW--------RC--DHRHPMIV-GAVKIRGVS-DSE---------------- |
| 32_OAV91411_Puccinia_triticina | FSGYN--F-ST--------YDEGPQDP----------------------------VAECGR------LGW--------RC--EHRHPMVV-GAIQLGAAA-SGQ---------------- |
| 32_EFP79978_Puccinia_graminis | FSGYH--F-SA--------YDDGPQDP----------------------------VAQCGR------FGW--------RC--EHRHPMVV-GAIQLGAAA-SDQ---------------- |
| 32_GAT51088_Mycena_chlorophos | YSGYN--Y-TS--------YDQGAPQS---------------------SSGYTNAV-SCXX------XXS--------RP--LFRLAPTA-MPFTIRTLV-RLSALGRPTPSREARSRP |
| 32_KNE94052_Puccinia_striiformis | FSGYN--F-TA--------YDEGPQDP----------------------------VAQCGR------FGW--------RC--EHRHAMIV-GAIQLGAAA-SDE---------------- |
| 32_KNZ54553_Puccinia_sorghi | FSGYNFTHY---------------------------------------------------------------------------DDPPLD-PVAQCG-RFGWRC---------------- |
| 42_AOF40721_Microbacterium_aurum | YYGSEIEFQAG----KQI--DCGPTCPLATTGRAYY-GDKIAGTVVASDFGKVASA-TGAV-------AD-------TLN--QPLVKHLQ-TLNQIRRAVP-AL---------------- |
| 42_CAB06816_Streptomyces_lividans | YYGSEIEFQKG----RKI--DCGPTCPLATTGRAYY-GDHLAGDVTASDFSRVDSA-TGAV-------AT-------TLA--QPLVKHVQ-RLNQIRRAVP-AL---------------- |
| 42_AWA81565_Pecoramyces_ruminatium | YYGSEVEFKKG----KKI--DDGPNVPLETTGRAYF-GDYIEGSVTTSDFGVYSNA-SGPI-------AD-------TLN--SPLSQHIR-RLNLIRRRIP-AL---------------- |

```
1_AAA85446_Paenibacillus_polymyxa            ------------------------------------------------QN------GSQREKWVDDSFYSFQRSK----NGD------EAIVFINNSWNSQT-RTI-GN----------
1_CAA49465_Thermoactinomyces_vulgaris        ------------------------------------------------RK------GTQAEKWVDDTFYAFQRSY----NGD------EVVVMINNSWNSQT-RTV-PN----------
1_BAD06003_Aspergillus_awamori               ------------------------------------------------DS--DYITYANDPIYTDSNTIAMRKGT----SGS------QVITVLSNKGSSGS-SYT-LTL----------
1_BAD06002_Aspergillus_awamori               ------------------------------------------------DT--GFVTYKNWPIYKDDTTIAMRKGT----DGS------QIVTILSNKGASGD-SYT-LSL----------
1_AAF14264_Aspergillus_flavus                ------------------------------------------------DT--GFVTYKNWPIYKDDTTIAMRKGT----DGS------QIVTILSNKGASGD-SYT-LSL----------
1_BAD01051_Aspergillus_kawachii              ------------------------------------------------DT--GFVTYKNWPIYKDDTTIAMRKGT----DGS------QIVTILSNKGASGD-SYT-LSL----------
1_BAA22993_Aspergillus_kawachii              ------------------------------------------------DS--DYITYKNDPIYTDSNTIAMRKGT----SGS------QIITVLSNKGSSGS-SYT-LTL----------
1_EAA64850_Aspergillus_nidulans              ------------------------------------------------NE--TYLSYKNYAIYNENNVLAMRKGF----DGS------QIITILTNAGADAG-SST-VSV----------
1_AAF17100_Aspergillus_nidulans              ------------------------------------------------DS--SYLTSRNTPFYSDSNYIAMRKGS----GGS------QVLTLLNNIGTSIG-SYT-FDL----------
1_P56271_Aspergillus_niger                   ------------------------------------------------DS--AYITYANDAFYTDSNTIAMAKGT----SGS------QVITVLSNKGSSGS-SYT-LTL----------
1_CAK44871_Aspergillus_niger                 ------------------------------------------------DT--GFVTYKNWPIYKDDTTIAMRKGT----DGS------QIVTILSNKGASGD-SYT-LSL----------
1_CAK40249_Aspergillus_niger                 ------------------------------------------------DN--HYPDVETYPIFEGGSELGFRKGI----EGR------QVVMLLSTQGTNSS-AYN-LSM----------
1_CAK41088_Aspergillus_niger                 ------------------------------------------------DS--SYISSKTYPIYQGGSELAFWKGN----NGR------QVIMVLSTAGSNGS-AYT-LTL----------
1_CAA31218_Aspergillus_oryzae                ------------------------------------------------DT--GFVTYKNWPIYKDDTTIAMRKGT----DGS------QIVTILSNKGASGD-SYT-LSL----------
1_BAA01255_Aspergillus_shirousami            ------------------------------------------------DT--GFVTYKNWPIYKDDTTIAMRKGT----DGS------QIVTILSNKGASGD-SYT-LSL----------
1_AEB80431_Aspergillus_tubingensis           ------------------------------------------------DS--DYITYKNDPIYTDSNTIAMRKGT----SGS------QIITVLSNKGSSGS-SYT-LTL----------
1_BAA12010_Cryptococcus_sp_S_2               ------------------------------------------------NG--GFVTYKIQVPYTDSNHIVTRKGN----SGY------QIVGVYTNVGSAGS-SST-LSI---------S
1_ABG48762_Fusicoccum_sp_BCC4124             ------------------------------------------------DA--TYLTYKNWVIYSDTTTIAMRKGY----DGK------QVITVLSNKGASGD-AYT-LNL----------
1_AAO12212_Lipomyces_kononenkoae             ------------------------------------------------TGTQVFYSDDTVFLGSGVSHMAMQRGP--------------LVIVLTNVGQHII-DNT--GY---------T
1_AAC49622_Lipomyces_spencermartinsiae       ------------------------------------------------DD--TYLTYQNWVIYSDSTTIAMRKGF----TGN------QIITVLSNLGSSGS-SYT-LTL----------
1_AAN75021_Lipomyces_starkeyi                ------------------------------------------------DD--TYLTYQNWVIYSDSTTIAMRKGF----TGN------QIITVLSNLGTSGS-SYT-LTL----------
1_AFD54462_Malbranchea_cinnamomea            ------------------------------------------------DD--AYITTPTDIKYSDDHTLALVKGA--------------VTTVLTNAGANAG-ETT-VTV----------
1_ABF72529_Ophiostoma_floccosum              ------------------------------------------------DS--SWATSANSPFYQDSNTIAMLKGSA---SGS------KVLTVLSNKGASGS-SYT-LSL----------
1_EPS26265_Penicillium_oxalicum              ------------------------------------------------DA--NYITSKNNAFYTDSNTIAMKKGS----SGS------QVVTVLSNRGSSGS-SYT-LSL----------
1_ABO42285_Phanerochaete_chrysosporium       ------------------------------------------------TD--NWLNSTTEVLTTTDYGLAIMKGP--------------VISIMTNIGSPVR-AFE-PVL----------
1_BAW15173_Pholiota_microspora               -------------------------------------------------T--DWVHKEAQILTTSPYGMAIMKGP--------------VISIVTNIGSPPQ-NGT-HIA----------
1_BAF98616_Pichia_burtonii                   ------------------------------------------------DN--SYPSTVSSIIYSDDHTIATKKAN--------------LVAIFTNVGASAS-P-S-VTL----------
1_AGJ52081_Rhizomucor_pusillus               -------------------------------------------------K------TVYMDIYVGDNAYAFKHGD------A------LVVL--NNYGSGST-N-Q-VSF----------
1_ADL28123_Rhizopus_oryzae                   -------------------------------------------------S------TVNMGIAQTDNVYVFQRGG------S------LVVV--NNYGQGST-N-T-ITV----------
1_ADD80242_Saccharomycopsis_fibuligera       ------------------------------------------------DS--SYATSQLSVIFSNDHVIATKRGS--------------VVSVFNNLGSSGS-S-D-VTI----------
1_ABS76467_Saitozyma_flava                   ------------------------------------------------NG--GFVTYKIQVPYTDSNHIVTRKGN----SGY------QIVGVYTNVGSAGA-SST-LSL---------S
1_CAB11471_Schizosaccharomyces_pombe         ------------------------------------------------EDGEIYIRSITHAIMIGDHVMVMYKGP--------------VITFITNYGAVDK-EYL-IKM----------
1_CAB40006_Schizosaccharomyces_pombe         ------------------------------------------------DP--DWVTSTYQSIESAVDHYVGQKND--------------VLVMFNNMGVTNN-L----TI---------Y
1_CAA34162_Schwanniomyces_occidentalis       ------------------------------------------------DS--AYSTYKSSVVSSSDHYIATRKGS----DAN------QLISIFNNLGSNGS-Q-D-ITV----------
1_CAA51912_Schwanniomyces_occidentalis       ------------------------------------------------DS--KYTTYWSDVLYASGHVIALQRGA----DDQ------RIVSVFNNLGSSGS-Q-T-VTF----------
1_ACN82436_Sclerotinia_sclerotiorum          ------------------------------------------------DP--TYLTYKAYPVYSDGTTIVMRKGF----TGK------QIIAVFSNKGASGS-SYT-LTL---------T
1_CAA03110_Thermomyces_lanuginosus           ------------------------------------------------NP--EFTSSKTKVIYQDDSTLAINRGG--------------VVTVLSNEGASGE-TGT-VSI----------
1_BAG69580_Trichoderma_viride                ------------------------------------------------AD------NDHKHLYVEPTAYAWSRAG------G------KLVAFTTNSGGGSS-A-Q-FCF----------
1_CAJ21046_Valsaria_rubricosa                ------------------------------------------------AG------NDHVHLYTTDNAYAWSRAS------G------KLIVVTSNRGSSDS-S-T-ICF----------
5_AWX66236_Alicyclobacillus_sp_18711         ------------------------------------------------QH--DYLDNQ--------DVIGWTREGD---SAHAG---SGLATVMSDG-PGGS----------------K
5_AAQ01675_Alkalimonas_amylolytica           ------------------------------------------------GH--EYSGNTE-------AVYAYVREGL---SNVPG---TGLVMLMSGR-NWGG---Q-QS---------F
5_CAL14744_Anoxybacillus_flavithermus        ------------------------------------------------QH--DYIDSA--------DIIGWTREGV---AEKAN---SGLAALITDG-PGGS----------------K
5_AEW07376_Bacillus_acidicola                ------------------------------------------------QH--DYLDHS--------DIIGWTREGV---TEKPG---SGLAALITDG-PGGS----------------K
5_AAA22191_Bacillus_amyloliquefaciens        ------------------------------------------------QH--DYIDHP--------DVIGWTREGD---SSAAK---SGLAALITDG-PGGS----------------K
5_ABY86223_Bacillus_cereus                   ------------------------------------------------QR--DYFDHP--------DVIGWTREGD---SVHAN---SGLATLISDG-PGGT----------------K
5_CAD26699_Bacillus_halmapalus               ------------------------------------------------QH--DYFDHH--------NIIGWTREGN---TTHPN---SGLATIMSDG-PGGE----------------K
5_AAA22226_Bacillus_licheniformis            ------------------------------------------------QH--DYFDHH--------DIVGWTREGD---SSVAN---SGLAALITDG-PGGA----------------K
5_AEM05860_Bacillus_licheniformis            ------------------------------------------------QH--DYFDHH--------NIVGWTREGD---SSVAN---SGLAALITDG-PGGT----------------K
5_AAK00598_Bacillus_megaterium               ------------------------------------------------QR--DYFDHP--------DVIGWTREGD---SVHAN---SGLATLISDG-PGGA----------------K
5_AGN35141_Bacillus_paralicheniformis        ------------------------------------------------QH--DYFDHH--------NIVGWTREGD---SSVAN---SGLAALITDG-PGGT----------------K
5_AAR68734_Bacillus_sp                       ------------------------------------------------QH--DYFDHH--------DIIGWTREGD---SSHPN---SGLATIMSDG-PGGN----------------K
5_AAA22231_Bacillus_sp_707                   ------------------------------------------------QN--DYLDHH--------NIIGWTREGN---TAHPN---SGLATIMSDG-AGGS----------------K
5_BAF03567_Bacillus_sp_JAMB_204              ------------------------------------------------QR--DYIDNQ--------DVIGWTREGN---TSKAK---SGLAALITDG-PGGA----------------K
5_CAC39917_Bacillus_sp_KSM_K38               ------------------------------------------------QH--DYFDHW--------DVVGWTREGS---SSRPN---SGLATIMSNG-PGGS----------------K
5_AAB18785_Bacillus_sp_MK_716                ------------------------------------------------QH--DYLDHS--------DIIGWTREGV---TEKPG---SGLAALITDG-PGGS----------------K
5_AAA63900_Bacillus_sp_TS_23                 ------------------------------------------------QR--DYIDHQ--------DIIGWTREGI---DTKPN---SGLAALITDG-PGGS----------------K
5_ABW87262_Bacillus_sp_YX_YX1                ------------------------------------------------QH--DYIDHP--------DVIGWTREGD---SSAAK---SGLAALITDG-PGGS----------------K
5_AAF00567_Cytophaga_sp                      ------------------------------------------------QR--DYIDNP--------DVIGWTREGD---STKAK---SGLATVITDG-PGGS----------------K
5_AAC74994_Escherichia_coli                  ------------------------------------------------QT--LFFDHP--------NCIAFSRSGT---DEFPG-----CVVVMSNG-DDGE----------------K
5_AFZ41193_Exiguobacterium_sp_DAU5           ------------------------------------------------QH--DYINHQ--------DVIGWTREGV---TDRAK---SGLATILSDG-PGGS----------------K
5_AAA22235_Geobacillus_stearothermophilus    ------------------------------------------------QH--DYLDHS--------DIIGWTREGV---TEKPG---SGLAALITDG-PGGS----------------K
5_ABX83871_Geobacillus_thermodenitrificans   ------------------------------------------------GH--DYLDHSH-------DIIGWTREGV---TEKPG---SGLAALITDG-PGGS----------------K
5_AFC87833_Geobacillus_thermoleovorans       ------------------------------------------------QH--DYLDHS--------DIIGWTREGV---TEKPG---SGLAALITDG-PGGS----------------K
5_ACL70573_Halothermothrix_orenii            ------------------------------------------------GY--EVDNNDA-------DIYSYVRSGF---PDVAG---DGLVLMISDG-TSGN---V-AG---------K
5_CAQ30277_Nostoc_sp_PCC_7119                ------------------------------------------------QY--NYLDHW--------NTIGWTRLGD---ADHPQ----GMAVIMSDG-SEGI----------------K
5_AAA27110_Salmonella_typhimurium            ------------------------------------------------QT--LFFDHP--------NCIAFSRSGT---EENPG-----CVVVLSNG-DDGE----------------K
5_BAA24178_Streptococcus_equinus             ------------------------------------------------EM--NYFDNP--------NCIGWSYLGD---KEHPT----SLAVLINNA-HSTA----------------K
5_AAA97431_Streptococcus_equinus             ------------------------------------------------EM--DYFDQA--------NCIGWTCLGD---DEHPT----ALAGLINNS-KATS----------------K
5_AAN59233_Streptococcus_mutans              ------------------------------------------------QE--DYFDYA--------NCIGWTCLGD---EEHPD----GVAVIISNG-EANC----------------K
5_CCD30600_uncultured_bacterium              ------------------------------------------------GY--EVDNNDE-------DVYSYVREGL---EEVEG---DGLVLMISDG-ESGE---I-IS---------K
5_ALP73597_Vibrio_alginolyticus              ------------------------------------------------QH--SYLDHW--------DVIGWTREGD---AKHPH---S-MAVIMSDG-PGGS----------------K
5_ABK62854_Histoplasma_capsulatum            ------------------------------------------------QR--DYFDKR--------HCIGFVRFGD---SGHPD----GLACVLSNG-PAAT----------------K
5_ABS11196_Paracoccidioides_brasiliensis     ------------------------------------------------QR--DYFNKR--------NCIGFVRMGD---SAHPH----GLACVLSNG-PADK----------------K
5_BAW15172_Pholiota_microspora               ------------------------------------------------TE--DYFAHP--------NCIGFVRKGD---ATHTG-----CAVILSNK-EDGPSTYL-HE---------L
15_AAB60935_Aedes_aegypti                    -------------------------------------------------------SLDHIQA-T-QNTFAFCRGE------------KGF-IVFNNS--E-N-TIT-QQY--------HT
15_AAB60934_Aedes_aegypti                    -------------------------------------------------------GINDWWSNG-NQQIAFCRGG------------SGF-VAFNLE--G-Y-DMN-ENL--------QT
15_AAA03322_Aedes_atropalpus                 -------------------------------------------------------NLNDWWTNG-NMQIAFCRGA------------NGF-VAFNLE--S-Y-DMN-ETL--------QT
15_AAA03323_Anopheles_merus                  -------------------------------------------------------PLQHWWDNG-NNQIAFARGN------------VGF-VAFNNE--P-F-DMN-VIL--------QT
15_AAN77138_Anthonomus_grandis               -------------------------------------------------------ELTNWWSGG-DNQIAFSRGD------------KGL-IAISIN--G---DIN-SNI--------PT
15_AAN77139_Anthonomus_grandis               -------------------------------------------------------DMQQYWSNG-N-QIAFSRGN------------KGF-FATTIG--G---DIN-QGI--------PT
15_AAO13755_Asterias_rubens                  -------------------------------------------------------PLTNWWDNG-NHMIAFGRGD------------KGF-LVINNE---HG-NLY-ETL--------QT
15_AAY23288_Blattella_germanica              -------------------------------------------------------AVNNWWDNG-DKQIAFSRGD------------KGF-IAFNNQ--YNT-HLM-HTL--------QT
15_BAB72257_Callosobruchus_chinensis         -------------------------------------------------------PLTNWWTDG-NQQIAFSRGN------------KGF-VAFTVS--G---DIS-ADL--------QT
15_AAA03350_Culex_tarsalis                   -------------------------------------------------------GVNDWWDNG-ANQMAFCRGN------------RGF-IAFNLE--S-F-NLS-QTL--------QT
15_AAF20998_Diabrotica_virgifera_virgifera   -------------------------------------------------------GINNWWSNG-HQQIAFGRGN------------KGF-IAFTLY--G---DIK-QSL--------QT
15_AAC35243_Drosophila_ananassae             -------------------------------------------------------PVQNWWDNG-NNQIAFSRGN------------KGF-VAFNND--N-S-DLN-QSL--------KT
15_BAB91544_Drosophila_bocki                 -------------------------------------------------------AIQNWWDNG-SNQIAFSRGS------------RGF-VAFNND--N-Y-DLN-SSL--------QT
15_BAA04582_Drosophila_erecta                -------------------------------------------------------AIQNWWDNG-SNQIAFSRGS------------RGF-VAFNND--N-Y-DLN-SSL--------QT
15_BAB91509_Drosophila_kikkawai              -------------------------------------------------------AIQNWWDNG-SNQIAFSRGS------------RGF-VAFNND--N-Y-DLN-SSL--------QT
15_BAA95437_Drosophila_leontia               -------------------------------------------------------AIQNWWDNG-SNQIAFSRGS------------RGF-VAFNND--N-Y-DLN-SSL--------QT
15_BAA95441_Drosophila_lini                  -------------------------------------------------------AIQNWWDNG-SNQIAFSRGS------------RGF-VAFNND--N-Y-DLN-SSL--------QT
15_BAA04583_Drosophila_mauritiana            -------------------------------------------------------AIQNWWDNG-SNQISFSRGS------------RGF-VAFNND--N-Y-DLN-SSL--------QT
15_AAK93479_Drosophila_melanogaster_Amyrel   -------------------------------------------------------EITGWWDNG-DNQISFCRGN------------KGF-LAINNN--L-Y-DLS-QDL--------NT
15_CAA28238_Drosophila_melanogaster          -------------------------------------------------------EIQNWWDNG-SNQISFSRGS------------RGF-VAFNND--N-Y-DLN-SSL--------QT
15_AAC46839_Drosophila_pseudoobscura         -------------------------------------------------------DLQNWWSNG-SNQISFSRGN------------RAF-VAFNND--N-Y-DLN-SSL--------QT
15_BAA04585_Drosophila_sechellia             -------------------------------------------------------AIQNWWDNG-SNQISFSRGS------------RGF-VAFNND--N-Y-DLN-SSL--------QT
15_AAC46463_Drosophila_virilis               -------------------------------------------------------QLQNWWDNG-SNQISFSRGS------------QGF-VAFNND--N-Y-DLN-SSL--------QT
15_AAG60011_Drosophila_yakuba                -------------------------------------------------------AIQNWWDNG-SNQIAFSRGS------------RGF-VAFNND--N-Y-DLN-SSL--------QT
15_CAA76926_Phaedon_cochleariae              -------------------------------------------------------SISNWWSDG-NQQIAFGRGD------------KGF-VAFTLA--G---DIN-GNL--------QT
15_P56634_Tenebrio_molitor                   -------------------------------------------------------QVENWWSND-DNQIAFSRGS------------QGF-VAFTNG--G---DLN-QNL--------NT
15_AAA03708_Tribolium_castaneum              -------------------------------------------------------GIENWWSDG-NQQIAFGRGN------------KGF-VAFTIG--Y---DLN-QHF--------ET
15_AAF73435_Zabrotes_subfasciatus            -------------------------------------------------------DMTNWWTDG-YQQIAFGRGN------------KGF-VAFSLS--G---DIK-ADL--------QT
24_ABL09312_Acarus_siro                      -------------------------------------------------------AVSHWWDNG-DYQIAFGRGS------------KAF-IAINLQDG--Q-GLN-RKL--------AT
24_BAB85635_Anguilla_japonica                -------------------------------------------------------PISHWWDNG-SNQVAFGRGN------------RGF-IVFNND---DR-HLD-MTL--------NT
24_AAL37207_Crassostrea_gigas                -------------------------------------------------------TMTNWWDNG-DYAIAFSRGN------------KGF-IVINAG---TS-DIN-VNL--------QT
24_AAL37183_Crassostrea_gigas                -------------------------------------------------------TLTNWWDNG-DYAIAFSRGN------------KGF-IVINAG---TS-DIN-VNL--------QT
24_AAD38942_Dermatophagoides_pteronyssinus   -------------------------------------------------------PVHNWWDNG-DYQIAFSRGN------------RAF-IAINLQKNQ-Q-NLQ-QKL--------HT
24_AAD38943_Euroglyphus_maynei               -------------------------------------------------------PVRNWWDNG-DYQIAFSRGS------------HAF-IAINLQKNGDK-NLR-QRL--------HT
24_AAC60246_Gallus_gallus                    -------------------------------------------------------PESNWWDNG-SNQVAFGRGD------------RGF-IVFNND---DW-YMN-VDL--------QT
24_ABO26610_Haliotis_discus_discus           -------------------------------------------------------GLSNWWSGS-DYQIAFSRGN------------KAF-IAFNLE---GY-DLS-KSL--------NT
24_BAM74656_Haliotis_discus_hannai           -------------------------------------------------------GLSNWWSGS-DYQIAFSRGN------------KAL-IAFNLE---GY-DLS-KSL--------NT
24_AAA51724_Homo_sapiens_pancreatic          -------------------------------------------------------PETNWYDNG-SNQVAFGRGN------------RGF-IVFNND---DW-SFS-LTL--------QT
24_AAH63129_Homo_sapiens_salivary            -------------------------------------------------------PETNWYDNG-SNQVAFGRGN------------RGF-IVFNND---DW-TFS-LTL--------QT
24_AAA37221_Mus_musculus                     -------------------------------------------------------PEANWWDND-SNQVAFGRGN------------KGL-IVFNND---DW-ALS-ETL--------QT
24_AAA37230_Mus_musculus                     -------------------------------------------------------PESNWWDNN-SNQVAFSRGN------------RGF-IVFNND---DW-ALS-ATL--------QT
24_H2N0D4_Oryzias_latipes                    -------------------------------------------------------PHANWWDNG-NNQVAFGRGN------------RGF-IVFNND---DW-ALD-VTL--------NT
24_CAA68065_Pecten_maximus                   -------------------------------------------------------NLQHWWDNG-NYQIAFGRGN------------KGF-IAMNMD---NH-NLD-QTL--------QT
24_CAA54524_Penaeus_vannamei                 -------------------------------------------------------DMNDWWDNG-SNQIAFCRGN------------KGF-LAINND---GW-DLK-ETL--------QT
24_CAB65552_Penaeus_vannamei                 -------------------------------------------------------DMNDWWDNG-SNQIAFCRGD------------KGF-LAINND---GW-DLK-ETL--------QT
24_AAF65827_Pseudopleuronectes_americanus    -------------------------------------------------------PHSNWWDNQ-SNQVAFGRGN------------RGF-IVFNND---DW-DLD-ETL--------NT
24_AAA40725_Rattus_norvegicus                -------------------------------------------------------PEANWWDNG-SNQVAFSRGN------------RGF-IVFNND---DW-ALS-STL--------QT
24_AAH88228_Rattus_norvegicus                -------------------------------------------------------PEANWWDNG-SNQVAFGRGN------------KGF-IVFNND---DW-DLS-TTL--------QT
24_P83053_Struthio_camelus                   -------------------------------------------------------PESNWWDNN-SNQVAFGRGS------------KGF-IVFNND---DW-HMN-VDL--------YT
24_AAF02828_Sus_scrofa                       -------------------------------------------------------PEANWWANG-SNQVAFGRGN------------RGF-IVFNND---DW-QLS-STL--------QT
24_CAC87125_Tetraodon_nigroviridis           -------------------------------------------------------PHSNWWDNN-SNQVAFGRGN------------RGF-IIFNND---DW-DLD-VTL--------NT
32_AAA21016_Aeromonas_hydrophila             -------------------------------------------------------PVSNWWDNG-NNQIAFSRGT------------RAS-SPSTTRAA--------------------
32_BAA22082_Bacillus_sp_195                  -------------------------------------------------------AVVNKWDNG-SSAIAFGRGD------------KGY-VAINRGS----ALT-RTF--------QT
32_AAY89038_Bifidobacterium_breve            -------------------------------------------------------DVTNWKAYD-TNVLGFGRGD------------VGY-LALNNSAD----DSK-QTF--------QT
32_CAB92963_Halomonas_meridiana              -------------------------------------------------------EITHWWDNG-HHQIAFSREA------------QGF-IAINREQQ----ALT-HTF--------QT
32_BAJ52728_Kocuria_varians                  -------------------------------------------------------PVTDAWTGG-EQSLAFGRGE------------KGF-LAINRAPS----GLD-ASW--------QT
32_AKG25402_Microbacterium_aurum             -------------------------------------------------------DIVNKWTSADGSAIGFGRGS------------KGY-VAINNGSS----SVQ-REF--------TT
32_CAA41481_Pseudoalteromonas_haloplanktis   -------------------------------------------------------AVTNWWDNT-NNQISFGRGS------------SGH-MAINKEDS----TLT-ATV--------QT
32_AAA86835_Pseudomonas_sp_KFCC10818         -------------------------------------------------------RVTNWWDDG-YNQVAFGRGG------------LGF-VVINRDDN--K-GIN-QGF--------AT
32_AAA96317_Streptomyces_albus               -------------------------------------------------------PVTDWWDDG-ADAIAFGRGS------------KGF-VAINHESA----TVQ-RTY--------QT
```

```
32_BAC73693_Streptomyces_avermitilis             ------------------------------------------------------SVTNWWDNG-GDAIAFGRGA------------KGY-VAINHESG----SLT-RTY--------QT
32_CAA40798_Streptomyces_griseus                 ------------------------------------------------------PVTNWWDNG-GDQIAFGRGD------------KAY-VAINHEGS----ALN-RTF--------QS
32_AAA26698_Streptomyces_hygroscopicus           ------------------------------------------------------ELTNWWDNG-GRPLAFARSD------------KGF-VALNNGDA----ALT-QTF--------AT
32_AAA88554_Streptomyces_limosus                 ------------------------------------------------------PVTNWWDNG-GDQIAFGRGD------------KAY-VAINHEGS----ALN-RTF--------QS
32_CAB06622_Streptomyces_lividans                ------------------------------------------------------GVTDWWDNG-GDQIAFGRGS------------KAY-VAINHEGT----SLT-RTF--------QT
32_CAA73775_Streptomyces_sp_TO1                  ------------------------------------------------------AVTDWWDNG-GDAIAFGRGS------------KGF-VAINHESA----TIS-RTY--------QT
32_AAA82875_Streptomyces_sp_WL6                  ------------------------------------------------------ELTDWWDNG-GSALAFGRGD------------KGF-VALNNADD----ALT-ETF--------TT
32_AAA26697_Streptomyces_thermoviolaceus         ------------------------------------------------------PVTDWWDDG-ADAIAFGRGS------------KGF-VAINHESA----TVQ-RTY--------QT
32_AAB36561_Streptomyces_venezuelae              ------------------------------------------------------AVTNWWDNG-NNAIAFGRGS------------KAY-VAINHETS----ALT-RTF--------QT
32_ABF13430_Thermobifida_fusca                   ------------------------------------------------------GIGSAVTDG-NGRLAFARGS------------AGY-AAFNATNT----AWT-RTF--------TT
32_CAA41881_Thermomonospora_curvata              ------------------------------------------------------GIGSAVTDG-NGRLAFARGS------------AGY-AAFNATNT----AWT-RTF--------TT
32_BAV60949_Pholiota_microspora                  ----------------------------------------------------SAAITDWVSPQ-AQQIAFGRGA------------LGF-VAINNADS----SWT-SAF--------TT
32_KJA26527_Hypholoma_sublateritium              ----------------------------------------------------SSALTNWVSPQ-SQQIAFGRGA------------LGF-VAINNVDS----TWT-ATF--------TT
32_KDR78015_Galerina_marginata                   ----------------------------------------------------TSAITNWVSPQ-SEQIAFGRGA------------LGF-VAINNADF----AWS-TSF--------TT
32_PPQ83160_Psilocybe_cyanescens                 ----------------------------------------------------TSALTNWVSPQ-SQQIAFGRGA------------LGF-VAINNADS----AWS-TTF--------TT
32_KTB32361_Moniliophthora_roreri                ----------------------------------------------------SAAINNWVSPQ-SQQIAFGRGS------------AGF-VAINNADS----TWS-ASF--------ST
32_KDQ30003_Pleurotus_ostreatus                  ----------------------------------------------------SAGITNWVSPQ-SQQIAFGRGS------------AGF-VAINNADS----SWT-ATF--------AT
32_RDX41646_Polyporus_brumalis                   ----------------------------------------------------NAALTNWVSPQ-GSQIAFGRGA------------LGF-VAINNADS----AWS-ATF--------ST
32_OJT13448_Trametes_pubescens                   ----------------------------------------------------SAALNNWVAPQ-SQQIAFGRGS------------LGF-VAINNADS----AWT-STF--------TT
32_PFH45132_Amanita_thiersii                     ----------------------------------------------------TASMTNWVSPQ-SQQIAFGRGS------------AGF-IVINNAGS----SWT-STF--------TT
32_KIM37787_Hebeloma_cylindrosporum              ----------------------------------------------------SAALTNWVSPQ-SQQIAFGRGS------------LGF-VAINNADY----AWS-ATF--------AT
32_PBK61492_Armillaria_solidipes                 ----------------------------------------------------SAALTDWVSPQ-SSQIAFGRGS------------AGF-VAINNAAS----DWS-SSF--------TT
32_ETW76368_Heterobasidion_irregulare            ----------------------------------------------------SAALNNWVAPS-SQQIAFGRGS------------IGF-VAINNVDS----SWT-ATF--------TS
32_RDB27268_Hypsizygus_marmoreus                 ----------------------------------------------------SAGITNWMSAQ-SQRIAFGRGS------------AGY-VAINNVDS----AWT-TTF--------ST
32_PPQ71147_Gymnopilus_dilepis                   ----------------------------------------------------SAPMTHWVSPQ-SQQIAFGRGS------------LGF-VAINNADT----AWN-AVF--------TT
32_OSD07916_Trametes_coccinea                    ----------------------------------------------------SAAMTNWVAPQ-SQQIAFGRGS------------LGF-VAINNADA----DWS-TTF--------ET
32_KZW01389_Exidia_glandulosa                    ----------------------------------------------------SAALTNWRSDS-SQRIAFGRGA------------LGF-VAINNQDD----AWS-ATF--------ST
32_EIW55835_Trametes_versicolor                  ----------------------------------------------------SAALNNWVAPQ-SQQIAFGRGS------------LGF-VAINNADS----AWT-STF--------TT
32_SJL11930_Armillaria_ostoyae                   ----------------------------------------------------STALTDWVSPQ-SSQIAFGRGS------------AGF-VAINNAAS----DWS-SSF--------TT
32_PAV18085_Pyrrhoderma_noxium                   ---------------------------------------------------SSNALSNWVSPS-SNQIAFSRGS------------VAF-VAINNEDS----SWT-AEF--------NT
32_RPD65356_Lentinus_tigrinus                    ----------------------------------------------------SAALTNWVSPQ-GSQIAFGRGA------------LGF-VAINNADS----DWS-ATF--------ST
32_PBK84861_Armillaria_gallica                   ----------------------------------------------------SAALTDWVSPQ-SSQIAFGRGS------------AGF-VAINNAAS----DWS-SSF--------KT
32_KLO18298_Schizopora_paradoxa                  ----------------------------------------------------AASMTNWVSAS-SQQIAFGRGS------------AGF-VAINNADS----TWT-TTF--------ET
32_PPR08271_Panaeolus_cyanescens                 ----------------------------------------------------ISEITNWVSPQ-SQQIAFGRGS------------TGF-VAINNADS----DWT-ATF--------IT
32_KIK53845_Gymnopus_luxurians                   ----------------------------------------------------SAALTNWVSPQ-SEQIAFGRGS------------LGF-VAINNADS----AWS-ATF--------TT
32_KIJ56431_Sphaerobolus_stellatus               -----------------------------------------------------GNVTNFVSGG-SQQIAFGRGS------------TGF-LAINNQDS----TWT-STF--------QT
32_EJD07843_Fomitiporia_mediterranea             ---------------------------------------------------SPNALTNWVSPS-SQRIAFARGA------------AGF-VAINNEDS----AWS-MTF--------NS
32_EPQ53992_Gloeophyllum_trabeum                 ----------------------------------------------------SAALTNWVSPG-SNQIAFGRGS------------AGF-VVINNQAS----SWT-GTF--------AT
32_KIP11288_Phlebiopsis_gigantea                 ----------------------------------------------------TAAITNWQSPQ-ASQIAFGRGA------------LGF-VAINNADT----AWS-ATF--------TT
32_KIY62009_Cylindrobasidium_torrendii           ----------------------------------------------------STAVTNWQSPQ-GNRIAFERAG------------TGF-VAINNDGS----AWT-GTF--------TT
32_KIJ69360_Hydnomerulius_pinastri               ----------------------------------------------------SAALENWVSPA-ADQIAFGRGS------------LGF-VAINNDDY----PWT-ATF--------TT
32_KZT20455_Neolentinus_lepideus                 ----------------------------------------------------SAALTDWVAPS-SQQIAFGRES------------AGF-VAINNEDT----SWT-TTF--------ST
32_EGN97351_Serpula_lacrymans_var_lacrymans      ----------------------------------------------------TAALGDWVSPS-SQQIAFGRGA------------LGF-VAINNEDS----TWA-TTF--------TT
32_KDQ06010_Botryobasidium_botryosum             ----------------------------------------------------GTAVGNFVSGN-SQQIAFSRGA------------VAF-VIINNDGG----SWS-RTF--------TT
32_PPR00074_Panaeolus_cyanescens                 ----------------------------------------------------SGSLVNWVSPA-ANRIAFGRGS------------AGY-VAINNADS----AWS-ATF--------RT
32_EKM53319_Phanerochaete_carnosa                ----------------------------------------------------TAAMNNWVSPQ-SEQIAFGRGS------------LGF-VAINNADA----VWT-TTF--------AT
32_TBU45601_Dichomitus_squalens                  ----------------------------------------------------DAEVTNWVSPQ-SEQIAFGRGA------------LGF-VAINNADS----DWS-ATF--------ST
32_PSR82077_Phlebia_centrifuga                   ----------------------------------------------------SAALVNWVSPQ-SQQIAFGRGA------------LGF-VAINNADS----DWT-ATF--------TT
32_KIM89391_Piloderma_croceum                    ----------------------------------------------------EAPITNWVSPQ-SNQIAFGRGS------------AGF-VAINNDDF----AWS-TTF--------TT
32_PIL31175_Ganoderma_sinense                    ----------------------------------------------------VAALTNWVSPQ-SQQIAFGRGD------------RGF-VAINNADS----AWS-TTF--------ST
32_KIY70325_Cylindrobasidium_torrendii           ----------------------------------------------------STALTNWQSPQ-GDRIAFERDG------------TGF-VAINNDNA----AWT-GTF--------TT
32_KDQ58060_Jaapia_argillacea                    ----------------------------------------------------DASLTDWVAPT-SQQIAFGRGS------------LGF-VAINNADS----AWT-ATF--------ST
32_CEL62905_Rhizoctonia_solani                   -----------------------------------------------------GSVNNWVSGT-NQQIAFGRGS------------SGF-VVINNADS----AWT-RTF--------TT
32_GAT57350_Mycena_chlorophos                    ----------------------------------------------------TAALVNWASPQ-ASQIAFGRGA------------LGF-IAINNADA----AWT-GTF--------TT
32_KIM25608_Serendipita_vermifera                ----------------------------------------------------SAAINNWVSGT-SQQIAFGRGS------------AGF-VVINNADS----SWS-ATF--------TT
32_GAW02849_Lentinula_edodes                     ----------------------------------------------------SAALNDWVSPQ-SEQIAFGRGA------------LGF-VAINNADT----VWS-ATF--------ST
32_TCD63303_Steccherinum_ochraceum               ----------------------------------------------------SAPLTDWVSPQ-PQLIAFGRGS------------LGF-VAINNIDS----PWS-ATF--------QT
32_KZP22139_Fibularhizoctonia_sp_CBS_109695      ----------------------------------------------------SSALTNWVSPQ-ADRIAFERQA------------LGY-LAINNDNA----AWV-GTF--------TT
32_KII90441_Plicaturopsis_crispa                 ----------------------------------------------------SEDLTDWVSPS-SQQIAFGRGS------------TGF-VAINNEDS----DWS-NTF--------ST
32_KDQ58058_Jaapia_argillacea                    ----------------------------------------------------SAPLTNWVAVG-AQQIAFGRGS------------AGF-VAINNADT----PWS-FQF--------QT
32_OCH93218_Obba_rivulosa                        ----------------------------------------------------DADMEGWVAEG-TNQIAFGRGS------------LGF-VAINNADT----VWS-TTF--------ST
32_EMD36390_Gelatoporia_subvermispora            ----------------------------------------------------DADMEGWIAEG-SNQIAFGRGS------------LGF-AAINNADT----IWS-TTF--------ST
32_EIW78746_Coniophora_puteana                   ----------------------------------------------------SANLTDWSSPS-SSQIAFGRGS------------NAF-VAINNEDS----DWS-TTF--------QT
32_KII86244_Plicaturopsis_crispa                 ----------------------------------------------------SAALTNWVSPQ-SDRIAFERTG------------LGY-VAINNDDS----AWG-ATF--------ST
32_EKM75205_Agaricus_bisporus                    ----------------------------------------------------SATLNNWVSPQ-SQQIAFGRGS------------LGY-VAINNANS----QWS-STF--------TT
32_KZT36083_Sistotremastrum_suecicum             ----------------------------------------------------SSAVTNYVAPS-SQQVAFGRGA------------LGF-VAINNQDS----TWT-SSF--------TT
32_KZS90559_Sistotremastrum_niveocremeum         ----------------------------------------------------SSAVTNYVAPS-SQQVAFGRGA------------LGF-VAINNQDS----TWT-SSF--------TT
32_EFI98134_Schizophyllum_commune                ----------------------------------------------------DAELTDWVSPQ-GSQIAFGRGS------------AGY-VAINNADS----EWS-TTF--------ST
32_BBC62173_Tricholoma_matsutake                 ----------------------------------------------------SAGITNWMSSG-PQQIAFGRGS------------AGF-MAMNNMDS----SWT-ATF--------VT
32_CCA69081_Serendipita_indica                   ----------------------------------------------------TDALNNWVSPA-SQQIAFGRGS------------SGF-VVINNADS----SWT-SSF--------TT
32_EFI98135_Schizophyllum_commune                ----------------------------------------------------DVPMSDWVSPS-SQRIAFGRGA------------LGF-VAINNVDQ----EWT-TTF--------AT
32_KII85613_Plicaturopsis_crispa                 ----------------------------------------------------AGSLNNFVSGT-SSQIAFGRGS------------AGH-VAINNGDK----DWS-VTL--------AT
32_EIN11730_Punctularia_strigosozonata           ----------------------------------------------------DAAVTDWVSPS-SDQIAFGRGS------------SGF-VAINNEDS----AWS-TTF--------QT
32_PIL33242_Ganoderma_sinense                    ----------------------------------------------------NAAMTSWTAPE-GQRIAFGRGA------------LGF-VAINNEDS----EWS-TTF--------AT
32_KZT18190_Neolentinus_lepideus                 ----------------------------------------------------KESITNWVAPT-EQQIAFGRGS------------KGF-VAINNADT----AWT-ANF--------TT
32_KZV75308_Peniophora_sp_CONT                   ----------------------------------------------------TAALNNWVAPS-SNQIAFGRGS------------VGF-VAINNQDS----QWS-TSF--------KT
32_KII86838_Plicaturopsis_crispa                 ----------------------------------------------------SGALQNFVTGT-SSQIAFGRGS------------AGL-VAINNDNS----AWT-VTL--------ST
32_KDQ08018_Botryobasidium_botryosum             ----------------------------------------------------GTPVANQVTGS-SQQIAFSRGA------------VAF-VAINNDGN----PWS-KTF--------AT
32_KIY73061_Cylindrobasidium_torrendii           ----------------------------------------------------DAELTNWQSPQ-GSQIAFERDG------------AGF-VAINNSDS----DWE-GTF--------TT
32_KZW01365_Exidia_glandulosa                    ------------------------------------------------------DVSNIQKGT-AQQLAFSRGT------------TGF-VAINNEDA----TWT-GSF--------ST
32_KDQ58178_Jaapia_argillacea                    ------------------------------------------------------PVTNWFASG-SQQIAFGRGS------------VAF-IVINNADA----HWS-GKF--------NT
32_KZV75307_Peniophora_sp_CONT                   ----------------------------------------------------TAVLNNWVAPS-SNQIAFGRGT------------VGF-VAINNQDG----SWS-TSF--------KT
32_KIK51212_Gymnopus_luxurians                   ----------------------------------------------------TAEMTNWVSPQ-SNQIAFGRGN------------LGF-IAINNDDF----EWS-TTF--------TT
32_RDX41931_Polyporus_brumalis                   ----------------------------------------------------TAPMTNWVAPD-SQRIAFGRGS------------LGF-VAINNEDS----AWT-ATF--------TT
32_TBU29254_Dichomitus_squalens                  ----------------------------------------------------NVDITNWTAPA-AQRIAFGRGS------------LGF-VAINNEDS----EWW-MKF--------HT
32_KIY45802_Fistulina_hepatica                   -----------------------------------------------------AGVSNWVSPQ-NDRIAFGRGT------------LGF-VAINNNDD----DWT-DTF--------NT
32_KIL59382_Amanita_muscaria                     ----------------------------------------------------SSPVTNWVSPQ-SNQIAFGRGS------------LGY-LAINNADT----PWT-SSF--------ST
32_KIY53725_Fistulina_hepatica                   -----------------------------------------------------AELTNWVSPQ-YDRIAFGRGT------------LGF-VGINNDDG----SWS-GTF--------TT
32_KIY47132_Fistulina_hepatica                   -----------------------------------------------------ASLTNWASPS-PQQIAFGRDR------------TGY-VAINNADT----HWR-TQF--------NT
32_RPD55342_Lentinus_tigrinus                    ----------------------------------------------------TAPMTNWVAPD-SQRIAFGRGS------------LGF-VAINNEDS----AWS-ATF--------KT
32_TCD61445_Steccherinum_ochraceum               ----------------------------------------------------SAALTNWVSPS-SDRIAFGRGN------------VGF-VIINNDDA----AWG-ATF--------TT
32_KIM25602_Serendipita_vermifera                ----------------------------------------------------DAPLGNWQTGT-HQQIAFGRGS------------SGF-VAINNEDG----EWS-PTF--------ST
32_EJD37603_Auricularia_subglabra                ------------------------------------------------------AVQNVQKGT-AQQVAFARGA------------TGF-VALNNEDA----VWS-GAF--------ST
32_KZT54766_Calocera_cornea                      ---------------------------------------------------TPL--DNVVTGT-SQQLAYGRGS------------TGF-IAINNGDN----GWS-TTF--------TT
32_KIY46805_Fistulina_hepatica                   -----------------------------------------------------APMTDWVSPA-NDRIAFGRGN------------FGF-VAINNDDA----AWP-ATF--------VT
32_PPQ69654_Psilocybe_cyanescens                 -----------------------------------------------------------------------------------------------------------------------
32_KZO93971_Calocera_viscosa                     ---------------------------------------------------SSTPLDHVVTGT-SQQLAYGRGS------------TGY-VAINNDDN----EWS-ATF--------ET
32_KZP07860_Fibularhizoctonia_sp_CBS_109695      -----------------------------------------------------SAVVNWAAGS-DQQIAFGRGS------------VAF-VAINNDVQ----PWS-QAF--------AT
32_PVG03687_Serendipita_vermifera                ------------------------------------------------------VLQNWQTGI-NQQIAFSRGS------------SGF-VAINNGDE----DWS-KTF--------TT
32_EIM87674_Stereum_hirsutum                     ---------------FNN-----------------------------AVG-QSSALTNIQEGT-NNQIAFGRGS------------IGF-VAINNAAS----SWS-ATF--------ST
32_CCA69087_Serendipita_indica                   ------------------------------------------------------EITNWQTGT-HQQIAFGRGN------------LGF-VAINNESA----QWS-AEF--------TT
32_CEL62035_Rhizoctonia_solani                   ------------------------------------------------------GLTNWAQGS-NQQIAFGRGD------------AGF-VAINNEDW----AWT-STF--------QT
32_EIN05398_Punctularia_strigosozonata           ----------------------------------------------------SAAVSNWQQGS-TNQIAFGRGS------------AGF-VAINNESG----AWS-KTF--------TT
32_EJU02896_Dacryopinax_primogenitus             ---------------------------------------------------ASL--DHVMTGT-AQQLAYGRGS------------TGF-VAINNDDS----AWS-ATF--------ST
32_KIO30302_Tulasnella_calospora                 ----------------------------------------------------GTSLNAWVSDQ-ASQIAFGRGS------------SGF-VAINYSSS----IWT-KTF--------TT
32_CEL62036_Rhizoctonia_solani                   ------------------------------------------------------GLGNWVQGS-NQQIAFGRGS------------SGF-VAINNEDW----QWT-QTF--------QT
32_KIO24533_Tulasnella_calospora                 ------------------------------------------------------ALKNWKQGD-SNQIAFGRGS------------VGY-VIINNSNN----KWK-TTF--------TT
32_EIM84100_Stereum_hirsutum                     ------------------------------------------------------SLTNWQTGS-NMQIAYERDA------------VGF-VIINNTDG----EWD-NTF--------TT
32_KDN35973_Rhizoctonia_solani                   ---------------------------------------------------RGNPHSNASTPL-CSRTVYGRSRNWIQSGKRGRCCIRF-IPSFRRTK----SWG-DYI--------RC
32_KIY48489_Fistulina_hepatica                   ------------------------------------------------------SMTNWTSAS-SQQIAFGRGS------------VGF-VAINNASS----EWS-TTF--------ST
32_KIY48486_Fistulina_hepatica                   ------------------------------------------------------SMVNWQMGT-SGQIAFGRGS------------TGF-VAINNASS----DWS-STF--------TT
32_KZP26813_Fibularhizoctonia_sp_CBS_109695      -----------------------------------------------------KNVTDWFSPN-SELIAFGRGS------------SGF-VAINNQNT----DWDSASF--------ST
32_KDQ32476_Pleurotus_ostreatus                  -----------------------------------------------------APLENLQSPR-DDRIAFSRGK------------SGF-VAINNSDN----PWS-ATL--------NT
32_EIM84087_Stereum_hirsutum                     ------------------------------------------------------PLTNWQSGS-PDQIAYERAG------------KGF-VVINNSDG----DWS-NSW--------TC
32_KDQ32475_Pleurotus_ostreatus                  -----------------------------------------------------TKLENWQSPQ-GDRIAFSRGN------------AGF-VAINNSDS----PWI-STL--------LT
32_EAQ88347_Chaetomium_globosum                  ------------------------------------------------------AVADWWDNG-NNQIAFGRGN------------KGF-VAINKEGS----SLT-RTF--------QT
32_ORY89754_Leucosporidium_creatinivorum         -----------------------------------------------------SAVTNSLATS-TNRSSFGRGS------------IGH-VAINFESS----SWD-ATL--------DT
32_EWZ34052_Fusarium_oxysporum                   ------------------------------------------------------GLTNWWDNG-NNAIGFGRGS------------MGY-VAINHESS----SVT-HTY--------QT
32_OBT91518_Pseudogymnoascus_verrucosus          ------------------------------------------------------ALTNWWDNG-NNAIGFGRGS------------KGY-VAINHESG----SVT-QTF--------QT
32_KEY73157_Stachybotrys_chartarum               ------------------------------------------------------AVTNWWDNG-NNQIAFGRGS------------RGF-IAINKESS----SLT-RTF--------QT
32_OBT62577_Pseudogymnoascus_sp_23342_1_I1       ------------------------------------------------------ALSNWWDNG-NNAIGFGRGS------------KGY-VAINHESG----SVT-QTY--------QT
32_EON97874_Phaeoacremonium_minimum              ------------------------------------------------------GLTDWWDNG-NNAIGFGRGD------------KGY-VAINHESG----SVT-RTY--------QT
32_GAT55198_Mycena_chlorophos                    -----------------------------------------------------QHLRDVAVGT-TQQVAFGRGT------------NGF-LVLNNDAT----VWT-QTF--------NT
32_OAG04271_Paraphaeosphaeria_sporulosa          ------------------------------------------------------GVTNWWDNG-NNQIAFGWGN------------QGF-VAINKEDS----LLT-QTL--------QT
32_EGG02960_Melampsora_larici_populina           ------------------------------------------------------PVRNIITSG-SNRTAYSRGS------------KGF-IALNNEPE----EWILPPG--------TQ
32_EGG02993_Melampsora_larici_populina           ------------------------------------------------------PVENLITSG-VNRTAYSRGS------------RAF-VALNNIPE----EWIFQSG--------TV
32_OAV91411_Puccinia_triticina                   ------------------------------------------------------PVHNIITSG-AHRLAFSRGS------------KVF-VALNNDDS----PWLLRDV--------LV
32_EFP79978_Puccinia_graminis                    ------------------------------------------------------PVHNIITSG-AHRLAFSRGS------------KVF-VAINNDDS----AWVLRDV--------VV
32_GAT51088_Mycena_chlorophos                    PSGRTTLSRSMSITIFLSALEARLAVEEAPSPLPSTCKSRRSSAKRSTSP-ARSRSSTIEGSS-NQQIAFGRGS------------IGF-VAINNAGS----SWS-QTF--------ST
32_KNE94052_Puccinia_striiformis                 ------------------------------------------------------PVQNIVTSG-AHRLAFSRGS------------KVF-VAINNDDS----DWLLEDV--------VV
32_KNZ54553_Puccinia_sorghi                      -----------------------------------------------E-----HRHP--------MIVGAVRLGA---AASGQ----PVQNIITVS-RGNQ---V-LSSLMRERKATEN
42_AOF40721_Microbacterium_aurum                 -----------------------------------------------QKGQ-------YSTEGVSGNMAFKRRFT---EGS------VD-SFVAVA-ISGG-A----------------
42_CAB06816_Streptomyces_lividans                -----------------------------------------------QMGQ-------YSTDGVTGDMAFKRRYT---DGG------TD-SFALVT-VSGG-A----------------
42_AWA81565_Pecoramyces_ruminatium               -----------------------------------------------QKGQ-------YA--SVDGQMAFKRRYT---DNN------TD-SFACVS-ITGG-A----------------
```

```
1_AAA85446_Paenibacillus_polymyxa          --FDNL-SNGTRLTNQL---SN----------------D--S-----VQI--NNGSITVTLA--P--K---EVKVFTK-
1_CAA49465_Thermoactinomyces_vulgaris      --IDRV-ANGQTLYNRM---GT----------------D--K-----VTV--NNGSITATLG--P--K---EVKIFTK-
1_BAD06003_Aspergillus_awamori             -SGSGY-TSGTELIEAY---TC----------------T--S-----VTV-DSNGDIPVPMA--S--G---LPRVLLPA
1_BAD06002_Aspergillus_awamori             -SGAGY-TAGQQLTEVI---GC----------------T--T-----VTV-GSDGNVPVPMA--G--G---LPRVLYPT
1_AAF14264_Aspergillus_flavus              -SGAGY-TAGQQLTEVI---GC----------------T--T-----VTV-GSDGNVPVPMA--G--G---LPRVLYPT
1_BAD01051_Aspergillus_kawachii            -SGAGY-TAGQQLTEVI---GC----------------T--T-----VTV-GSDGNVPVPMA--G--G---LPRVLYPT
1_BAA22993_Aspergillus_kawachii            -SGSGY-TSGTKLIEAY---TC----------------T--S-----VTV-DSNGDIPVPMA--S--G---LPRVLLPA
1_EAA64850_Aspergillus_nidulans            -PNTGF-TAGAAVTEIY---TC----------------E--D-----ITV-SGSGEVSVPME--S--G---LPRVLYPK
1_AAF17100_Aspergillus_nidulans            -YDHGY-NSGANLVELY---TC----------------S--S-----VQV-GSNGAISIPMT--S--G---LPRVLVPA
1_P56271_Aspergillus_niger                 -SGSGY-TSGTKLIEAY---TC----------------T--S-----VTV-DSSGDIPVPMA--S--G---LPRVLLPA
1_CAK44871_Aspergillus_niger               -SGAGY-TAGQQLTEVI---GC----------------T--T-----VTV-GSDGNVPVPMA--G--G---LPRVLYPT
1_CAK40249_Aspergillus_niger               --PVSF-TGGTVVTEIL---NC----------------V--N-----YTV-NTQSELVVPMD--K--G---EPRVFFPA
1_CAK41088_Aspergillus_niger               --PVSY-GASEVVTEVL---NC----------------V--N-----YTV-NTYSQLVVDMD--K--G---EPRVFFPA
1_CAA31218_Aspergillus_oryzae              -SGAGY-TAGQQLTEVI---GC----------------T--T-----VTV-GSDGNVPVPMA--G--G---LPRVLYPT
1_BAA01255_Aspergillus_shirousami          -SGAGY-TAGQQLTEVI---GC----------------T--T-----VTV-GSDGNVPVPMA--G--G---LPRVLYPT
1_AEB80431_Aspergillus_tubingensis         -SGSGY-TSGTKLIEAY---TC----------------T--S-----VTV-DSNGDIPVPMA--S--G---LPRVLLPA
1_BAA12010_Cryptococcus_sp_S_2             SSETGF-QASEPVMDVL---SC----------------T--L-----YHT-GTDGSLSFTMT--G--G---LPRVFYNA
1_ABG48762_Fusicoccum_sp_BCC4124           -SNTGW-TSGTEVVEIL---TC----------------A--T-----TTV-TSGNTVTVPMA--N--G---LPRIYFPL
1_AAO12212_Lipomyces_kononenkoae           VTGSQF-SAGDSLTDLV---SC----------------T--K-----VKVVGANGTFTSPSN--G--G---KARIWIKS
1_AAC49622_Lipomyces_spencermartinsiae     -SNTGY-TASSVVYEIL---TC----------------T--A-----VTV-DLSGNLAVPMS--G--G---LPRVFYPE
1_AAN75021_Lipomyces_starkeyi              -SNTGY-TASSVVYEIL---TC----------------T--A-----VTV-DSSGNLAVPMS--S--G---LPKVFYQE
1_AFD54462_Malbranchea_cinnamomea          -EATGY-ASGEQVTDVL---SC----------------E--S-----IAA-SDGGRLSVTLN--Q--G---LPRVFFPT
1_ABF72529_Ophiostoma_floccosum            -GSTGY-SSGASLVELY---SC----------------T--T-----VTV-DSSGNVPVPMA--S--G---LPRVLVPS
1_EPS26265_Penicillium_oxalicum            -SGSGY-EAGTKLVEMY---TC----------------T--A-----VTV-DSNGNIAVSMT--S--G---LPRVFMLA
1_ABO42285_Phanerochaete_chrysosporium     -D------AMANVPVIL---TC----------------T--Q-----HVV-GSNGTVEVEYSK-G--G---VPTILVPT
1_BAW15173_Pholiota_microspora             -VSSPY-LPSTAMVNIL---TC----------------A--Q-----WAV-GAKGMLDAQYTL-G--G---VPNILIPL
1_BAF98616_Pichia_burtonii                 -SQSGF-QKGDQVVDLL---TC----------------S--S-----TTV-GD-SGLEVTMA--L--G---VPKVYASS
1_AGJ52081_Rhizomucor_pusillus             SVSGKF-DSGASLMDIV---SN----------------I--T-----TTV-SSDGTVTFNLK--D--G---LPAIFTSA
1_ADL28123_Rhizopus_oryzae                 K-AGSF-SNGDTLTDVF---SN----------------K--S-----VTV-Q-NNQITFQLQ--N--G---NPAIFQKN
1_ADD80242_Saccharomycopsis_fibuligera     -SNTGY-SSGEDLVEVL---TC----------------S--T-----VSG---SSDLQVSIQ--G--G---QPQIFVPA
1_ABS76467_Saitozyma_flava                 SSETGF-QASEPVMDVL---SC----------------T--L-----YHT-GSDGSLSFTMT--G--G---LPRVFYNA
1_CAB11471_Schizosaccharomyces_pombe       -------PGSETMIDLL---TC----------------T--L-----IEV-EG-EVMRTSIK--K--G---EPKILYPY
1_CAB40006_Schizosaccharomyces_pombe       EVETNY-TANEVVSDVF---GH----------------R--T-----LTV-GADKTLTASMT--N--G---YPLIMYPH
1_CAA34162_Schwanniomyces_occidentalis     -SNTGY-SSGDKVIDII---SC----------------N--S-----VLA-GDSGSLSVSIS--G--G---MPQVYAPS
1_CAA51912_Schwanniomyces_occidentalis     -S-TKY-SGGEKVVDVL---TC----------------Q--T-----SYA-NSDSTLTVSIS--G--G---APRIYAPA
1_ACN82436_Sclerotinia_sclerotiorum        SSQTGF-TSNLQVVEVL---TC----------------T--T-----STT-NGSGNLAVSMA--G--G---VPRIFYPK
1_CAA03110_Thermomyces_lanuginosus         -PGTGF-EAGTELTDVI---SC----------------K--T-----VTA-GDSGAVDVPLS--G--G---LPSVLYPS
1_BAG69580_Trichoderma_viride              --GTQV-PNG-SWTNVFDGGNG----------------P--T-----YTA-DGNGQLCLTTT--N--G---EPIVLLSS
1_CAJ21046_Valsaria_rubricosa              --STQQ-ASGTTWTSTI---TG----------------N--S-----YTA-DSNGQICVQLS--S--G---GPEALVVS
5_AWX66236_Alicyclobacillus_sp_18711       TMYVGTAHAGQVFKDITG-NRT----------------D--T-----VTI-NSAGNGTFPCN--G--G---SVSIWVKQ
5_AAQ01675_Alkalimonas_amylolytica         SINSHQA--NTTFYDYTG-NVA----------------G--T-----VTT-NAQGYGSFPVN--M--TESTGWSVWVPQ
5_CAL14744_Anoxybacillus_flavithermus      WMYVGKQHAGKTFYDLTG-NRS----------------D--T-----VTI-NADGWGEFKVN--G--G---SVSIWVPK
5_AEW07376_Bacillus_acidicola              WMYVGKQHAGKVFYDLTG-NRS----------------D--T-----VTI-NSDGWGEFKVN--G--G---SVSVWVP-
5_AAA22191_Bacillus_amyloliquefaciens      RMYAGLKNAGETWYDITG-NRS----------------D--T-----VKI-GSDGWGEFHVN--D--G---SVSIYVQK
5_ABY86223_Bacillus_cereus                 WMDVGKNNAGEVWHDITG-NQT----------------N--T-----VTI-NKDGWGQFQVS--G--G---SVSIYVQQ
5_CAD26699_Bacillus_halmapalus             WMYVGQNKAGQVWHDITG-NKP----------------G--T-----VTI-NADGWANFSVN--G--G---SVSIWVKR
5_AAA22226_Bacillus_licheniformis          RMYVGRQNAGETWHDITG-NRS----------------E--P-----VVI-NSEGWGEFHVN--G--G---SVSIYVQR
5_AEM05860_Bacillus_licheniformis          RMYVGRQNAGETWHDITG-NRS----------------D--S-----VVI-NAEGWGEFHVN--G--G---SVSIYVQR
5_AAK00598_Bacillus_megaterium             WMDVGKNNAGEIWYDITG-NQT----------------N--T-----VTI-NKDGWGQFQVS--G--G---SVSIYVQR
5_AGN35141_Bacillus_paralicheniformis      RMYVGRQNAGETWHDITG-NRS----------------D--S-----VVI-NAEGWGEFHVN--G--G---SVSIYVQR
5_AAR68734_Bacillus_sp                     WMYVGKHKAGQVWRDITG-NRS----------------G--T-----VTI-NADGWGNFTVN--G--G---AVSVWVKQ
5_AAA22231_Bacillus_sp_707                 WMFVGRNKAGQVWSDITG-NRT----------------G--T-----VTI-NADGWGNFSVN--G--G---SVSIWVNK
5_BAF03567_Bacillus_sp_JAMB_204            RMYVGTQNAGEVWYDITG-NRT----------------D--K-----VTI-GSDGWAAFNVN--G--G---SVSVYVQQ
5_CAC39917_Bacillus_sp_KSM_K38             WMYVGRQNAGQTWTDLTG-NNG----------------A--S-----VTI-NGDGWGEFFTN--G--G---SVSVYVNQ
5_AAB18785_Bacillus_sp_MK_716              WMYVGKQHAGKVFYDLTG-NRS----------------D--T-----VTI-NSDGWGEFKVN--G--G---SVSVWVPR
5_AAA63900_Bacillus_sp_TS_23               WMYVGKKHAGKVFYDLTG-NRS----------------D--T-----VTI-NADGWGEFKVN--G--G---SVSIWVAK
5_ABW87262_Bacillus_sp_YX_YX1              RMYAGLKNAGETWYDITG-NRS----------------D--T-----VKI-GSDGWGEFHVN--G--G---SVSIYVQK
5_AAF00567_Cytophaga_sp                    RMYVGTSNAGEIWYDLTG-NRT----------------D--K-----ITI-GSDGYATFPVN--G--G---SVSVWVQQ
5_AAC74994_Escherichia_coli                TIHLGENYGNKTWRDFLG-NRQ----------------E--R-----VVT-DENGEATFFCN--G--G---SVSVWVIE
5_AFZ41193_Exiguobacterium_sp_DAU5         WMYVGKQNAGEVWKDMTG-NNG----------------R--L-----VTI-NADGWGEFFVN--G--G---SVSIYTQQ
5_AAA22235_Geobacillus_stearothermophilus  WMYVGKQHAGKVFYDLTG-NRS----------------D--T-----VTI-NSDGWGEFKVN--G--G---SVSVWVPR
5_ABX83871_Geobacillus_thermodenitrificans WMYVGKQHAGGNRTTVGG-MET----------------A--L-----TNG-SSCDRQEGYPC--V--F---YVSVWVPR
5_AFC87833_Geobacillus_thermoleovorans     WMYVGKQHAGKVFYDLTG-NRS----------------D--T-----VTI-NSDGWGEFKVN--G--G---SVSVWVPR
5_ACL70573_Halothermothrix_orenii          WINSRQP--DTEFYDLTG-HIK----------------E--H-----VTT-DSEGYGNFKVI--K--SEDKGWSIWVPV
5_CAQ30277_Nostoc_sp_PCC_7119              WMEVGKPNTK--FIDLTE-HIK----------------E--A-----VYT-NEWGWGEFRCL--G--G---SVSVWVQA
5_AAA27110_Salmonella_typhimurium          TLLLGDNYANKTWRDFSG-NRD----------------E--Y-----VVT-NDQGEATFFCN--A--G---SVSVWVIE
5_BAA24178_Streptococcus_equinus           RMFVGKKWAGKTFTDYLG-NQN----------------S--T-----IVI-DEEGYGSFPVG--A--E---SVSAYIPQ
5_AAA97431_Streptococcus_equinus           RMFVGEKWACKLFTDALG-NQA----------------A--H-----VQI-DEQGYGDFLVG--E--K---SVSAWIPL
5_AAN59233_Streptococcus_mutans            RMNMGEFNRNKVFVDYLN-NCT----------------E--E-----VIL-DDQGWGDFPVQ--E--A---SLSAWVNK
5_CCD30600_uncultured_bacterium            RINSGQP--NTTFYDYTG-HVQ----------------G--K-----IIT-DERGYGDFKVR--K--SENEGWSVWVPV
5_ALP73597_Vibrio_alginolyticus            WMYTGKPSAR--YVDKLG-IRT----------------E--E-----VWT-DANGWAEFPVN--G--G---SVSVWVSV
5_ABK62854_Histoplasma_capsulatum          RMYVGKAHADSVWTEIYG-YCR----------------S--R-----VKI-DSRGYGVFGVV--A--R---NVSVWVRE
5_ABS11196_Paracoccidioides_brasiliensis   RMNVGKSHAGSVWTEVYG-TCR----------------S--R-----VTI-DRRGYGVFGVV--S--Y---SVSVWVRE
5_BAW15172_Pholiota_microspora             RMNVGIENAGKTYRSIMT--QH----------------G--R-----VQI-DASGWGIFSCF--A--N---TVQVWTTS
15_AAB60935_Aedes_aegypti                  CLPQG------QYCDIISGEVS------------QSTCTGTV-----VNV-DANGDAEITLPK-N-----SVVAIYIPS
15_AAB60934_Aedes_aegypti                  CLSAG------TYCDVISGSKE------------GGSSLVLQ-----CKS-V-----EMDVPI-SIFRPKVMMVF--WP
15_AAA03322_Aedes_atropalpus               CLPAG------TYCDVISGSKE------------GGSCTGAT-----VQV-GGDGRANIQIPA-H--GDDGVLAIHINA
15_AAA03323_Anopheles_merus                GLPAG------IYCDVISGARE------------GETCTGLQ-----VIV-EPNGFASISIRA-N--AEDGVIAIHSEV
15_AAN77138_Anthonomus_grandis             GLPDG------TYCDVISGSLS------------NGSCTGKT-----VTV-SG-GSAYISISS-G--DTNAAVAIHVNA
15_AAN77139_Anthonomus_grandis             TLADG------TYCDVMTGKLS------------NGSCTGKT-----VTV-SG-GNVQVQLSA-G--GDEIAMAIHVES
15_AAO13755_Asterias_rubens                GLPQG------TYCNIYLGDYV------------HGMCTGPS-----VFV-DYQGFATFNIQGGG--NEDPVVALHIRS
15_AAY23288_Blattella_germanica            PLPAG------LYCDITSGSKN------------GSSCTGKT-----VTV-ENDGTAYIQILS-N--EQDGVLALHLES
15_BAB72257_Callosobruchus_chinensis       SLPAG------SYCDLITGISS------------NNSCTGKV-----VTV-DGSGKAHIDV---S--GVDGVLAIHVNS
15_AAA03350_Culex_tarsalis                 CLPAG------TYCDVISGVKQ------------GSTCTGGT-----VTV-GANGLAQITIQT-SAW--DGVLAIHADS
15_AAF20998_Diabrotica_virgifera_virgifera SLPAG------TYCDVISGSLE------------KGSCTGKT-----VNV-DSSGKAAISLST-N--EEDGVVAIHVNA
15_AAC35243_Drosophila_ananassae           ELPKG------SYCDVISGAKK------------GSVCTGKT-----VVI-ESNGMARININR-S--EYDGVLAIHINA
15_BAB91544_Drosophila_bocki               GLPAG------TYCDVISGTKN------------GSSCTGKT-----VTV-GSDGRASIYLGN-S--EDDGVLAIHVNA
15_BAA04582_Drosophila_erecta              GLPAG------TYCDVISGSKS------------GSSCTGRT-----VSV-GSDGRASISVGR-S--EDDGVLAIHVNA
15_BAB91509_Drosophila_kikkawai            GLPAG------TYCDVISGTKN------------GSSCTGKT-----VTV-GSDGRASIYLGN-S--EDDGVLAIHVNA
15_BAA95437_Drosophila_leontia             GLPAG------TYCDIISGTKN------------GSSCTGKT-----VTV-GSDGRASIYLGN-S--EDDGVLAIHVNA
15_BAA95441_Drosophila_lini                GLPAG------TYCDVISGTKN------------GSSCTGKT-----VTV-GSDGRASIYLGN-S--EDDGVLAIHVNA
15_BAA04583_Drosophila_mauritiana          GLPAG------TYCDVISGSKS------------GSSCTGKT-----VSV-GSDGRASIYIGS-S--EDDGVLAIHVNA
15_AAK93479_Drosophila_melanogaster_Amyrel CLPAG------TYCDVISGSLI------------DGSCTGKS-----VTV-NENGYGYIHIGS-D--DFDGVLALHVDA
15_CAA28238_Drosophila_melanogaster        GLPAG------TYCDVISGSKS------------GSSCTGKT-----VTV-GSDGRASINIGS-S--EDDGVLAIHVNA
15_AAC46839_Drosophila_pseudoobscura       GLPAG------TYCDVISGEKS------------GSSCTGKT-----VTV-GSDGRASISISS-S--ADDGVLAIHVNA
15_BAA04585_Drosophila_sechellia           GLPAG------TYCDVISGSKS------------GSSCTGKT-----VSV-GSDGRASIYIGS-S--EDDGVLAIHVNA
15_AAC46463_Drosophila_virilis             GLSAG------TYCDVISGTKS------------GSGCTGKT-----ITV-GSDGRANIYIGS-S--EEDGVVAIHVNA
15_AAG60011_Drosophila_yakuba              GLPAG------TYCDVISGSKS------------GSSCTGKT-----VSV-GSDGRASIYLGS-S--EDDGVLAIHVNA
15_CAA76926_Phaedon_cochleariae            SLPAG------SYCDIVSGKLE------------NGSCTGKT-----VNV-DGNGQAYITLSS-G--EDDGFLAIHVGA
15_P56634_Tenebrio_molitor                 GLPAG------TYCDVISGELS------------GGSCTGKS-----VTV-GDNGSADISLGS-A--EDDGVLAIHVNA
15_AAA03708_Tribolium_castaneum            GLPAG------SYCDVISGNAE------------NGSCSGKT-----ITV-GGDGYADISLGA-N--EDDGVIAIHVNA
15_AAF73435_Zabrotes_subfasciatus          SLPPG------TYCDVITGDIS------------NNSCTGKT-----VTV-RGDGKATIHLSS-G--EPDGILAIHVSA
24_ABL09312_Acarus_siro                    GLPQG------TYCDLVTGNLA------------GGKCTGGT-----VTV-DGSGNADINIAK-T--AEDPFVAIHVEA
24_BAB85635_Anguilla_japonica              GLPGG------TYCDIISGQKE------------GSRCSGKQ-----VTV-DSDGRAHFNISN-M--DEDPFIAIHVDS
24_AAL37207_Crassostrea_gigas              GLSQG------TYCDVISGNYE------------NGSCTGNE-----VHV-GGDGHAHFHISS-G--SEDPVVAIHIGA
24_AAL37183_Crassostrea_gigas              GLSQG------TYCDVISGNYD------------NGRCTGNE-----VHV-GGDGHAHFHISS-G--SDDPVVAIHIGA
24_AAD38942_Dermatophagoides_pteronyssinus GLPAG------TYCDIISGNLI------------DNKCTGKS-----IHV-DKNGQADVYVGH-D--EFDAFVAYHIGA
24_AAD38943_Euroglyphus_maynei             GLPAG------TYCDIISGDLI------------HNKCTGKS-----IQV-DKNGLADIYVGH-D--ELDAFVAYHIGA
24_AAC60246_Gallus_gallus                  GLPAG------TYCDVISGQKE------------GSACTGKQ-----VYV-SSDGKANFQISN-S--DEDPFVAIHVDA
24_ABO26610_Haliotis_discus_discus         GLPSG------SYCDVISGNLE------------NGGCTGKT-----IDV-DGSGHASIHISS-S--DKDPMVAIHVGA
24_BAM74656_Haliotis_discus_hannai         GLPSG------SYCDVISGNLE------------NGGCTGKT-----IDV-DGSGHASIHISS-S--DKDPMVAIHVGA
24_AAA51724_Homo_sapiens_pancreatic        GLPAG------TYCDVISGDKI------------NGNCTGIK-----IYV-SDDGKAHFSISN-S--AEDPFIAIHAES
24_AAH63129_Homo_sapiens_salivary          GLPAG------TYCDVISGDKI------------NGNCTGIK-----IYV-SDDGKAHFSISN-S--AEDPFIAIHAES
24_AAA37221_Mus_musculus                   GLPAG------TYCDVISGDKV------------DGNCTGIK-----VYV-GNDGKAHFSISN-S--AEDPFIAIHAES
24_AAA37230_Mus_musculus                   GLPAG------TYCDVISGDKV------------DGNCTGLR-----VNV-GSDGKAHFSISN-S--AEDPFIAIHADS
24_H2N0D4_Oryzias_latipes                  GLPGG------TYCDVISGNKD------------GGSCTGKQ-----ITV-GGDGRAHFYINN-S--EEDPFIAIHADS
24_CAA68065_Pecten_maximus                 GLPAG------TYCDVISGSYD------------GSSCSGTE-----IQV-GNDGNAHFSISN-S--SDDPMIAIHVGA
24_CAA54524_Penaeus_vannamei               CLPAG------TYCDVISGSKN------------GGSCTGKS-----VTV-GGDGKAYIEITT-M--EDDGVLAIHANS
24_CAB65552_Penaeus_vannamei               CLPAG------TYCDVISGSKD------------GGSCTGKS-----VTV-GGDG------------------------
24_AAF65827_Pseudopleuronectes_americanus  GMPGG------TYCDVISGQKE------------GGRCTGKQ-----IQV-GGDGRAHFKISS-R--DEDPFVAIHAES
24_AAA40725_Rattus_norvegicus              GLPAG------TYCDVISGDKV------------NGNCTGLK-----VNV-GSDGKAHFSISN-S--AEDPFIAIHADS
24_AAH88228_Rattus_norvegicus              GLPAG------TYCDVISGDKV------------DGNCTGIK-----VYV-GSDGNAYFSISN-S--AEDPFIAIHVES
24_P83053_Struthio_camelus                 GLPAG------TYCDVISGQKE------------GSRCTGIQ-----VYV-SGNGKANFQISN-N--AEDPFIAIHVGA
24_AAF02828_Sus_scrofa                     GLPGG------TYCDVISGDKV------------GNSCTGIK-----VYV-SSDGTAQFSISN-S--AEDPFIAIHAES
24_CAC87125_Tetraodon_nigroviridis         GLPAG------TYCDVISGQKE------------AGRCTGKQ-----IHV-GSDGRAHFRISN-R--DEDPFVAIHVES
32_AAA21016_Aeromonas_hydrophila           -----------RWWPRCRRSRPAS----------T-------------------------ATS-W-----GAMTTAAAV
32_BAA22082_Bacillus_sp_195                SLPAG------NYCNVIVGLPN------------STGCSAGG----VVTVDAAGTFTATVDQ--N-----SAFALHVG-
32_AAY89038_Bifidobacterium_breve          SLPAG------EYCNVYAT----------------GDCS--A----TVTVKDDGTFDATLAK--N-----SAIAIYAGA
32_CAB92963_Halomonas_meridiana            DMADG------RYQNVTAEGQC--------------------------IV-VEDGQLTLSVPA-M-----SAAALHVGA
32_BAJ52728_Kocuria_varians                SLPAG------EYCDVYSGVPT------------ASGCSGET-----LTV-AADGTVSGHVAP-T-----SALALHVDA
32_AKG25402_Microbacterium_aurum           SLPDG------TYYNVIKATKA------------GNSWS--G----ETITVAGGKFTATLGA--K-----DAIALHVDA
32_CAA41481_Pseudoalteromonas_haloplanktis DMASG------QYCNVLKGELSAD----------AKSCSGEV-----ITV-NSDGTINLNIGA-W-----DAMAIHKNA
32_AAA86835_Pseudomonas_sp_KFCC10818       GMPAG------EYCDIISGDFNSQ----------TGTCSGTT-----ISV-DSSGYAHFTVAS-H-----NAAAIHVGA
32_AAA96317_Streptomyces_albus             SLPAG------TYCDVQSN----------------------T----TVTVDSAGRFTAALGP--D-----TALALHTGR
```

| | |
|---|---|
| 32_BAC73693_Streptomyces_avermitilis | SLTAG------TYCNVQNN----------------------T----GVTVDSSGRFTATLGA--N-----TALALYSGK |
| 32_CAA40798_Streptomyces_griseus | GLPGG------AYCDVQSG----------------------R----SVTVGSDGTFTATVAA--G-----TALALHTGA |
| 32_AAA26698_Streptomyces_hygroscopicus | SLPAG------TYCDVVHA-AS--------------SCDG-D----TVTV-GDTEAQV--DA--A-----KSVALHVGA |
| 32_AAA88554_Streptomyces_limosus | GLPGG------AYCDVQSG----------------------R----SVTVGSDGTFTATVAA--G-----TALALHTGA |
| 32_CAB06622_Streptomyces_lividans | SLPAG------DYCDVQTG----------------------K----GVTVDGAGRFTATLGA--G-----TAVALHVGA |
| 32_CAA73775_Streptomyces_sp_TO1 | SLPPG------PYCNVQNN----------------------T----TCSV-NGSGQLTATLAA-N-----TALAVYAGK |
| 32_AAA82875_Streptomyces_sp_WL6 | SLPAG------TYCNVAAASPD--------------DCDG-N----TVTV-GDDGAVQATVPA-R-----GALALHTGA |
| 32_AAA26697_Streptomyces_thermoviolaceus | SLPAG------TYCDVQSN----------------------T----TVTVDSAGRFTAALGP--D-----TALALHNGR |
| 32_AAB36561_Streptomyces_venezuelae | SLPAG------SYCDVQSN----------------------T----PVTVNSSGQFTATLAA--N-----TAVALHVNA |
| 32_ABF13430_Thermobifida_fusca | SLPDG------VYCDVANGTFV------------DGVCDGPS------YQVSGGKFTATVPA--N-----GAVALHVEA |
| 32_CAA41881_Thermomonospora_curvata | SLPDG------VYCDVANGTFV------------DGVCDGPS------YQVSGGKFTATVPA--N-----GAVALHVEA |
| 32_BAV60949_Pholiota_microspora | SLPAG------SYCDVISGKN--S----------SGTCSGSS-----FVVS-GGTVSATIPA--R-----SAIALHTGS |
| 32_KJA26527_Hypholoma_sublateritium | SLPNG------SYCDVISGAS--S----------SGTCTGFS-----ITVS-GGTFTATVPA--R-----SAIAIHTGA |
| 32_KDR78015_Galerina_marginata | SLPPG------SYCDVISGR-S-S----------SGSCTGTG-----FTVL-GGTFTATVPA--R-----GGIAIHTGA |
| 32_PPQ83160_Psilocybe_cyanescens | SLPAG------SYCDVISGI-S-S----------SGACTGTG-----ITVS-GGTFTANVPA--R-----SAIAIHTGA |
| 32_KTB32361_Moniliophthora_roreri | SLADG------SYCDVISGR-S-S----------DGACTGVG-----VTVS-SGVFNANVPP--R-----SAIAIHTGQ |
| 32_KDQ30003_Pleurotus_ostreatus | SLPAG------AYCDVISGS-A-N----------AGTCSGLS-----ITVA-GGSFSATVPA--R-----SAIAIHTGA |
| 32_RDX41646_Polyporus_brumalis | SLPDG------TYCDVISGAS--S----------SGSCTGSS-----FTVS-GGSFSATVPA--R-----SSIAIHTGQ |
| 32_OJT13448_Trametes_pubescens | SLPDG------SYCDVVSGQT--S----------GTNCTGSS-----FTVS-SGSLSATVPA--R-----SAIAVHTGQ |
| 32_PFH45132_Amanita_thiersii | SLPDG------SYCDVITGK-S-S----------GGACTGKS-----VTVS-GGRFSATVNA--H-----DALAIHIGA |
| 32_KIM37787_Hebeloma_cylindrosporum | SLPDG------SYCDVISGK-N-S----------SGSCTGTS-----MTVA-GGFFTATVPA--R-----SAIAIHTGS |
| 32_PBK61492_Armillaria_solidipes | SLADG------SYCDVVSGT-S-S----------NGTCTGSG-----FTVS-GGSFTATVPA--H-----SAVAIHTGA |
| 32_ETW76368_Heterobasidion_irregulare | SLPAG------TYCDVVSGGS--S----------SGNCTGSS-----FTVSSSGTFAATIGS--R-----SAIAIHTGA |
| 32_RDB27268_Hypsizygus_marmoreus | SLPAG------TYCDVISGK-A-S----------SGSCTGAA-----ITVS-GGSFTATVAA--R-----NAIAIHTGA |
| 32_PPQ71147_Gymnopilus_dilepis | SLPSG------SYCDVISGS-S-S----------SGLCTGLG-----FTVS-EGTFTATVPA--R-----GAIAIHTGA |
| 32_OSD07916_Trametes_coccinea | SLPDG------SYCDVVSGTS--S----------DGSCTGSS-----FNVS-SGSLTATVPA--R-----SAIAVHTGA |
| 32_KZW01389_Exidia_glandulosa | SLPAG------TYCDVVAGK-P-T----------GTTCAGAA-----YTVS-GGTFSATISG--R-----NALALYVGA |
| 32_EIW55835_Trametes_versicolor | SLPDG------TYCDVVSGQT--S----------GTTCTGSS-----FTVS-SGSLSATVPA--R-----SAIAVHTGQ |
| 32_SJL11930_Armillaria_ostoyae | SLADG------SYCDVIGGT-S-S----------DGTCTGSG-----FIVS-GGSFTATVPA--H-----SAVAIHTGV |
| 32_PAV18085_Pyrrhoderma_noxium | GLPEG------EYCDVIAGAPQ-T----------AGTCRGQS-----FNVSSTGTFSTTISA--R-----NAVAIHIGA |
| 32_RPD65356_Lentinus_tigrinus | SLPDG------SYCDVISGAS--S----------SGSCTGSS-----FTVS-GGSFSATVPA--R-----SSIAIHTGA |
| 32_PBK84861_Armillaria_gallica | SLADG------SYCDVVSGT-S-S----------NGICTGSG-----FTVS-GGSFTATVPA--H-----SAVAIHIGA |
| 32_KLO18298_Schizopora_paradoxa | SMPEG------SYCDVVSGSS--V----------AGICSGYS-----FTVSSDGTSNVVVPA--R-----TAIAIHTGA |
| 32_PPR08271_Panaeolus_cyanescens | SLPDG------NYCDVIHGT-A-S----------GGTCSGPS-----VSVS-GGKFTAIVAA--R-----NAIAFHTSA |
| 32_KIK53845_Gymnopus_luxurians | SLPAG------SYCDVISGTS--S----------SGKCTGTG-----ITVS-GGTFTATVPA--R-----SAIAIHTGA |
| 32_KIJ56431_Sphaerobolus_stellatus | SLPAG------TYCDVVAGPL--S----------NGACQGAS-----ITVSSCGTFTATISA--R-----NALAIHIGA |
| 32_EJD07843_Fomitiporia_mediterranea | GLPAG------SYCDVVAGVP--N----------GAVCAGPS-----YTVASDGTFTPIISA--R-----NALAIHIGA |
| 32_EPQ53992_Gloeophyllum_trabeum | SLPAG------TYCDVVHGNS--S----------GGVCSASA-----ITVASDGTFSATVNP--Y-----DALAIHTGS |
| 32_KIP11288_Phlebiopsis_gigantea | SLPAG------TYCDVISGTL--S----------SGACTGSA-----FTVS-GGKFTANVPA--R-----YALAIHTAA |
| 32_KIY62009_Cylindrobasidium_torrendii | SLAAG------TYCDVISGT-A-T----------NGTCTGAS-----YTVS-GGSFTATVAA--R-----SAVAIHTGA |
| 32_KIJ69360_Hydnomerulius_pinastri | PLPAG------TYCDVISGPPS-G-----------STCSGAS-----YTVASGGTFSATVLA--R-----NAIALHTGA |
| 32_KZT20455_Neolentinus_lepideus | SLPAG------AYCDVINGAA--S----------NGTCSGTV-----YTVASDGTFSATINA--Y-----DAIALHTGA |
| 32_EGN97351_Serpula_lacrymans_var_lacrymans | SLPAG------SYCDVISGPPT-S----------SGTCAGAS-----YTVASDGTFSATVAT--A-----NAIALHTGA |
| 32_KDQ06010_Botryobasidium_botryosum | GLPNG------SYCDVANGA-L-S----------GSSCTGPS-----YTVT-GGSLTVTVPA--R-----SAIALHTGA |
| 32_PPR00074_Panaeolus_cyanescens | SLPDG------TYCDVISGK-S-S----------GSSCTGAS-----YTVS-GGSFSANVPA--R-----SAVALHTGQ |
| 32_EKM53319_Phanerochaete_carnosa | SLPDG------TYCDVISGIS--S----------SGVCTGTA-----FTVS-DGKFTAGVQA--R-----NAIAIHSGA |
| 32_TBU45601_Dichomitus_squalens | SLPSG------SYCDVISGSS--S----------GSTCTGSS-----FTVS-GGSFTATVPA--R-----SAIAIHTGQ |
| 32_PSR82077_Phlebia_centrifuga | SLPAG------SYCDVISGTS--S----------SGKCTGLG-----FTVS-GGTFSATVPA--R-----SAIAIHTGA |
| 32_KIM89391_Piloderma_croceum | SFADG------SYCDVISGNS--A----------SGSCTGTS-----FSVS-NGSLTATVPA--R-----SAIAIYTGA |
| 32_PIL31175_Ganoderma_sinense | SLPNG------SYCDVVHGSA--F----------DGFCTGSS-----FTVS-GGSFSATVPA--R-----DSIAIHTGA |
| 32_KIY70325_Cylindrobasidium_torrendii | SLADG------SYCDVITGT-A-S----------SGSCTGDS-----YTVS-GGSFSATVAA--R-----SAVAIHTGA |
| 32_KDQ58060_Jaapia_argillacea | PLPAG------SYCNVIDGAA--S----------GTTCTGSA-----FIVASDGTFTATIGA--R-----NALALHTGA |
| 32_CEL62905_Rhizoctonia_solani | SLAAN------SYCDVVSGD-V-S----------GGKCTGAS-----YTVS-GGSFTAMVPA--K-----SAIALFTGA |
| 32_GAT57350_Mycena_chlorophos | SLPNG------VYCDVISGK-S-T----------GGACSGST-----FVVS-AGKFTATVSA--R-----NAVALHIGA |
| 32_KIM25608_Serendipita_vermifera | SLPSG------TYCDVYTGP-K-S----------GSSCSGSS-----YTVS-GGSFSATVAA--R-----TALALHTGA |
| 32_GAW02849_Lentinula_edodes | SLPAG------SYCDVISGTS--S----------SGNCTGTG-----ITVS-GGLFTASVPA--R-----SAIAIHTGA |
| 32_TCD63303_Steccherinum_ochraceum | SLSDG------PYCDVVSGS-S-F----------NGQCSAAS-----FNVT-GGAFTAIVPA--R-----SAVAIHAGS |
| 32_KZP22139_Fibularhizoctonia_sp_CBS_109695 | TLAAG------SYCDVITGSS--V----------SGACTGTG-----ITVS-GGSFSATVPA--R-----SAIAIHTGQ |
| 32_KII90441_Plicaturopsis_crispa | SLAAG------SYCDVISGTAS-S-----------GSCSGTA-----ITVGSDGTFTATVPS--R-----SAIAIHTGA |
| 32_KDQ58058_Jaapia_argillacea | SLPAG------SYCDIIHGVL--S----------GNTCTGPS-----FVVASDGIFTATVAP--R-----DAVALHIGA |
| 32_OCH93218_Obba_rivulosa | SLPDG------TYCDVISGSS--V----------NGACTGTA-----ITVS-GGTFDTNVQA--W-----SSLAIHTGS |
| 32_EMD36390_Gelatoporia_subvermispora | SMPDG------TYCDVISGAS--V----------SGACTGVA-----VIVS-GGQFTANVQA--W-----SALAIHTGS |
| 32_EIW78746_Coniophora_puteana | SLAAG------SYCEVTSGPPS-S----------NGSCAGAA-----FTVGSDGTFTGTISS--R-----AAIALHTGA |
| 32_KII86244_Plicaturopsis_crispa | GLADG------KYCDVISGA-L-S----------SGACTGSF-----FTVS-GGSFTATVPA--R-----SALAIHTGA |
| 32_EKM75205_Agaricus_bisporus | SLPDG------TYCDVIRGT-V-S----------NGRCSGPS-----YTVS-GGRFTATVLQ--R-----DAIAIHTGA |
| 32_KZT36083_Sistotremastrum_suecicum | SLPAG------SYCDVASGSAA-S----------GGKCSGAA-----YTVS-GGKVSFTVPA--R-----TAIALHTGQ |
| 32_KZS90559_Sistotremastrum_niveocremeum | SLPAG------SYCDVASGSAA-S----------GGKCSGAA-----YVVS-GGKVSLTVPA--R-----TAIALHTGQ |
| 32_EFI98134_Schizophyllum_commune | SLADG------SYCDVISGD-A-S----------DGSCSGNT-----ITVS-GGSFDATVPA--R-----SALAIHTGA |
| 32_BBC62173_Tricholoma_matsutake | SLADG------TYCDVIKGE-S-A----------SGTS---T-----HTVR-GGLFTATIGA--R-----DALAIHTGA |
| 32_CCA69081_Serendipita_indica | SLPDG------TYCDVYTGP-K-S----------GSNCSGAA-----YTVS-GGVFSATVAA--R-----TALALHTGA |
| 32_EFI98135_Schizophyllum_commune | SLPDG------KYCDVVDAQ-V-V----------AGKCSGTT-----YTVS-GGKFEGKVPA--R-----SALALHVGA |
| 32_KII85613_Plicaturopsis_crispa | ALGNG------AYCDVVGGALTVD----------GMGCTGSA-----FIVY-AGAVSFTVPA--R-----NAVAIHVGA |
| 32_EIN11730_Punctularia_strigosozonata | SLAAG------TYCDVIAGPDS-T----------AGSCTNST-----ITVASDGTFTATVGA--Q-----NAVAIHTGA |
| 32_PIL33242_Ganoderma_sinense | TLPDG------SYCDVVGGS-L-S----------NGQCTGST-----FAVS-GGSFSATVPA--R-----GAIAVHAGA |
| 32_KZT18190_Neolentinus_lepideus | SLSAG------TYCDVIHGT-A-A----------PGKCDNPV-----YTVADDGTFAATVAP--R-----DALALHVGA |
| 32_KZV75308_Peniophora_sp_CONT | SLPDG------SYCDVVHETN--A----------KGPCTGTT-----YTVS-GGSFSATIAA--R-----EALAIYVAA |
| 32_KII86838_Plicaturopsis_crispa | LLKDG------AYCDVVSGAVA-N----------AYGCTGSG-----YTVS-SGSVTLTVPA--R-----DAVALHIAA |
| 32_KDQ08018_Botryobasidium_botryosum | SLPDG------SYCDVANGA-A-S----------GSGCTGPS-----YTIS-GGNLNVNIPA--H-----SAIALHTGA |
| 32_KIY73061_Cylindrobasidium_torrendii | SLADG------TYCDVITGA-S-S----------DGSCSGDS-----YTVS-GGSFSATVAS--R-----SAVAIHVGA |
| 32_KZW01365_Exidia_glandulosa | GLAAG------NYCNVYEGARN-T----------TGDCAASF-----VTVDDQGAASLNIAP--R-----SAVAIYVGA |
| 32_KDQ58178_Jaapia_argillacea | SLPAG------TYCDVIHGTIT------------PGRCDQPV-----YTIAKDGSFEADIKP--R-----DALALHTGI |
| 32_KZV75307_Peniophora_sp_CONT | SLPDG------SYCDVVHESN--A----------SGSCTGTT-----YTVS-GGSFSATIGG--R-----DALAIYAA- |
| 32_KIK51212_Gymnopus_luxurians | SLGDG------TYCDAISGVV--S----------SSSCTGTN-----FTVS-GGSFDATVPA--R-----SAIAIHTGA |
| 32_RDX41931_Polyporus_brumalis | SLPDG------TYCDVIKGQ-L-S----------NGQCSGTT-----FTVN-GGSFKATVPQ--R-----GAIAIHTGA |
| 32_TBU29254_Dichomitus_squalens | SLPDG------SYCDVISGKRS-S----------NGKCNGST-----ITVS-QGSFTATVPA--R-----GSIAIHAGA |
| 32_KIY45802_Fistulina_hepatica | SLPDG------TYCEVTTTGLD-AVF--------MGACNGTS-----VTVS-NGSFSAIVPS--N-----GAFAIHTGA |
| 32_KIL59382_Amanita_muscaria | SLPNG------IYCDVISGS-M-S----------NGSCSGRN-----ATVS-DGSFNATISP--R-----NALALHTTA |
| 32_KIY53725_Fistulina_hepatica | SLPDG------TYCEVTSTGTD-PVDS-------DGVCTGES-----ATVL-AGALTATIYA--R-----SSFAIHTGA |
| 32_KIY47132_Fistulina_hepatica | SLPDG------HYCNVVDGVSQ-N----------GVSCTGSSYLRSSSFIVHNGAFTATLYP--R-----TALAMHTGS |
| 32_RPD55342_Lentinus_tigrinus | SLPDG------TYCDVIKGQ-L-S----------NGQCSGST-----FTVS-GGSFTATVPQ--R-----GAIAIHTGA |
| 32_TCD61445_Steccherinum_ochraceum | GLPDG------VYTDVISGS--------------------AN-----FTVS-GGQLKTAVPI--R-----DAIALHVGA |
| 32_KIM25602_Serendipita_vermifera | GLPDG------TYCDVYAGP-K-G----------NNGCAGAT-----YTVS-GGSFSAKLPG--R-----SAIALHTQA |
| 32_EJD37603_Auricularia_subglabra | GLPQG------NYCNVFDGARN-S----------TGDCALGI-----VAVDAEGYAALNVPP--R-----AAVAIYVGA |
| 32_KZT54766_Calocera_cornea | MLADG------RYCDLVTG-GS-F----------GTQCTGAS-----YDVAS-SAFTATIPP--R-----SAVVLYTGS |
| 32_KIY46805_Fistulina_hepatica | SLPNG------KFCEVSAVGVH-AVTS-------EESCGGVS-----VVVN-DGSFSATVPP--R-----GAFAIHTGA |
| 32_PPQ69654_Psilocybe_cyanescens | ------------------------------------------------------------------------------- |
| 32_KZO93971_Calocera_viscosa | LLPDG------QYCDLITGGT--L----------EAECSGAS-----YDVA-AGSFSATIPS--R-----SAIALFAGS |
| 32_KZP07860_Fibularhizoctonia_sp_CBS_109695 | GLPAG------IYCDVIHGSVV------------SGVCSSVA-----YTVSSSGILNITVSA--R-----DAIAVHTNA |
| 32_PVG03687_Serendipita_vermifera | GLPDA------TYCDVYAGPMT------------SSGCAGTS-----YKVT-NGSVNITIPG--R-----TAVALHTGA |
| 32_EIM87674_Stereum_hirsutum | SLPDG------TYCDIIHDTDT-D----------PTVCSSTT-----YTVS-GGSFTATVGA--Y-----DAIAIYTSS |
| 32_CCA69087_Serendipita_indica | SLPDG------SYCNVNGGA-P------------RDGCMGSP-----ITIS-GGKFTANITP--R-----TALAVHVGA |
| 32_CEL62035_Rhizoctonia_solani | SLSSG------TYCDVASGKKE------------GNNCTGGR-----VTISNGSFTVT-VPG--R-----SAVAYHVGA |
| 32_EIN05398_Punctularia_strigosozonata | SLPDG------TYCNVIDGAI--S----------SGACSAST-----FTVA-GGKFTATVRG--Y-----WAIALHTGA |
| 32_EJU02896_Dacryopinax_primogenitus | SLHDG------RYCDLIAG-GL-S----------GAECAGSS-----YDVSR-ESFSATIPP--R-----SAIALYVGS |
| 32_KIO30302_Tulasnella_calospora | SLPDG------SYCDVVHGSL---------------------------IVVSGGKITMAVPT--Y-----DALAVYVGA |
| 32_CEL62036_Rhizoctonia_solani | SLPSG------TYCDVVSGRKN------------GGECTGGK-----VTISNGSFTVT-IPS--R-----TAVAYHIGA |
| 32_KIO24533_Tulasnella_calospora | GLPDG------KYCDVISGKKS------------GSSCTSKV-----VTVRGGKLTNYGVGQ--H-----NAVAIHIGA |
| 32_EIM84100_Stereum_hirsutum | GLADG------TYCDVISGAVS------------GSSCTGTT-----ITVSG-GKVTASVPS--R-----GAVAFYSGS |
| 32_KDN35973_Rhizoctonia_solani | -------------WELLATWEL-G----------AGKCDSAI-----FLGSC-MDCKHNNAD--R-------IHIF--- |
| 32_KIY48489_Fistulina_hepatica | SLAAG------TYCDVITGGSN------------SSGCAGTE-----ITVGSDGTFSASVPA--I-----GALAIYTGS |
| 32_KIY48486_Fistulina_hepatica | SLAEG------TYCDVISGAST------------NGTCTGTS-----ISVGGDGSFTASVPA--I-----SAIAIYTGA |
| 32_KZP26813_Fibularhizoctonia_sp_CBS_109695 | SMDAG------SYCDVTTGNMT------------SNACTGTK-----VTVTSGGAITITVPS--Y-----GAIAIHSGA |
| 32_KDQ32476_Pleurotus_ostreatus | GLPKG------SYCDVVSGGAK-AS-----------KCTGPT------------------------------------- |
| 32_EIM84087_Stereum_hirsutum | GLPDG------IYCDVVSGSKD------------GSQCTGSS-----INV-SG-GTCSGTAPA-R-----NALAVYTDS |
| 32_KDQ32475_Pleurotus_ostreatus | GLPKG------WYCDVVSGGSS-GRSNGRSEGGKGGGCTGRT------------------------------------- |
| 32_EAQ88347_Chaetomium_globosum | SLPAG------TYCNVQAN----------------------T----AVTVDGSGQFTATLGS--N-----TALAIYVGK |
| 32_ORY89754_Leucosporidium_creatinivorum | DVADG------TYCEITSGCAS-K-----------------------ITVS-GGSFTVTIPA--Y-----GSVAFYTGS |
| 32_EWZ34052_Fusarium_oxysporum | SLPAG------TYCNVQSN----------------------T----YVTVDSSGRFAATLSS--N-----TALAIYVGK |
| 32_OBT91518_Pseudogymnoascus_verrucosus | SLSAG------TYCNVQSN----------------------T----YVTVNSSGQFTATLAS--D-----TALAIYAGK |
| 32_KEY73157_Stachybotrys_chartarum | SLPAG------TYCNVQSN----------------------T----AVTVNSSGQFTATLSS--N-----TALAIYVGK |
| 32_OBT62577_Pseudogymnoascus_sp_23342_1_I1 | SLPAG------TYCNVQSK----------------------T----SVTVNSSGQFTATLGT--N-----TALAIYAGK |
| 32_EON97874_Phaeoacremonium_minimum | SLPAG------TYCNVQSN----------------------T----YVTVDSSGQFTATLGS--D-----IALAIYAGK |
| 32_GAT55198_Mycena_chlorophos | SLPAG------DYCDIIHDTDT-S----------PTTCSGPT-----ITVASDGTFSASVNA--F-----DALALYSSA |
| 32_OAG04271_Paraphaeosphaeria_sporulosa | SLPAG------KYCNVQSN----------------------S----AVTVNSSGHFTATLGP--N-----TALAIYAGK |
| 32_EGG02960_Melampsora_larici_populina | ---IGM-KPG-DYPDVLGSNQ----IV-------VRVGGR---------G--RLIDSIVIPG--F-----SAVGIHL-- |
| 32_EGG02993_Melampsora_larici_populina | ---IGM-NEG-NYTDVLGSNS----TT-------VQVCPQ---------G--KLIGNLVVPG--F-----SAVGIHQ-- |
| 32_OAV91411_Puccinia_triticina | GLPVPT-SPGNAYRDRLHPSDD-GQLVH------LQRDPS---------SGLSRLRPLTIPP--R-----SAIGIMVV- |
| 32_EFP79978_Puccinia_graminis | GLPLPA-SPEDAYRDRLHPAGE-GQLVH------LQPDPS---------SSLARLCPLTIAP--R-----SAIGIMVV- |
| 32_GAT51088_Mycena_chlorophos | SLPAG------TYCDAIHDTNT-S----------PSVCTGTT-----YTVS-GGSFSATIGA--Y-----DAIALYVNN |
| 32_KNE94052_Puccinia_striiformis | GLPLPA-SPGDAYRDRLHPSGD-GQLVH------LQSDPS---------CGLTRLRPLSIPP--R-----SAIGIMVFE |
| 32_KNZ54553_Puccinia_sorghi | RLFFFWGGGGYEWFEKSG-KQR----------------L--A--------FSRGERVFVAL---N--NEDTSWEVVDVV |
| 42_AOF40721_Microbacterium_aurum | -TFTGL-PNG-TYVDAV---TG----------------D--T-----KVVSN-GTLTISLSG--Q--G---NMRAYVLN |
| 42_CAB06816_Streptomyces_lividans | -AYDGI-PNG-TYRDAV---TG----------------D--T-----RTVTD-GRLTVAAPG--K--G---NLRVYVLN |
| 42_AWA81565_Pecoramyces_ruminatium | -TFNGL-PGG-TYKEVI---TG----------------E--V-----RSIPEGGSISIPNTG--K--G---NMRIWVLD |

**Figure S1.** Sequence alignment of all 268 collected sequences representing $\alpha$-amylases from subfamilies GH13_1, GH13_5, GH13_15, GH13_24, GH13_32 and GH13_42. The alignment spans the segment of sequences from the beginning of the strand β1 (CSR-VIII) to the end of the strand β8 (CSR-VII) of the catalytic TIM-barrel (including the domain B) completed by a part succeeding the strand β8 (CSR-VII) since there are residues involved in SBSs. The CSRs are and catalytic triad are highlighted in yellow and red, respectively. The residues involved in chloride binding are signified by green; dark green is used for lysine replacing arginine in the CSR-VII. For SBS residues, blue highlighting is applied for residues conserved within a particular subfamily; outside subfamily, the corresponding residues are signified by cyan. The sequences are ordered from the top according to subfamilies starting with the subfamily GH13_1. Within each subfamily, sequences within subfamilies (except for GH13_15 and GH13_24) are grouped as bacteria followed by fungi; each group being arranged alphabetically.

**A**

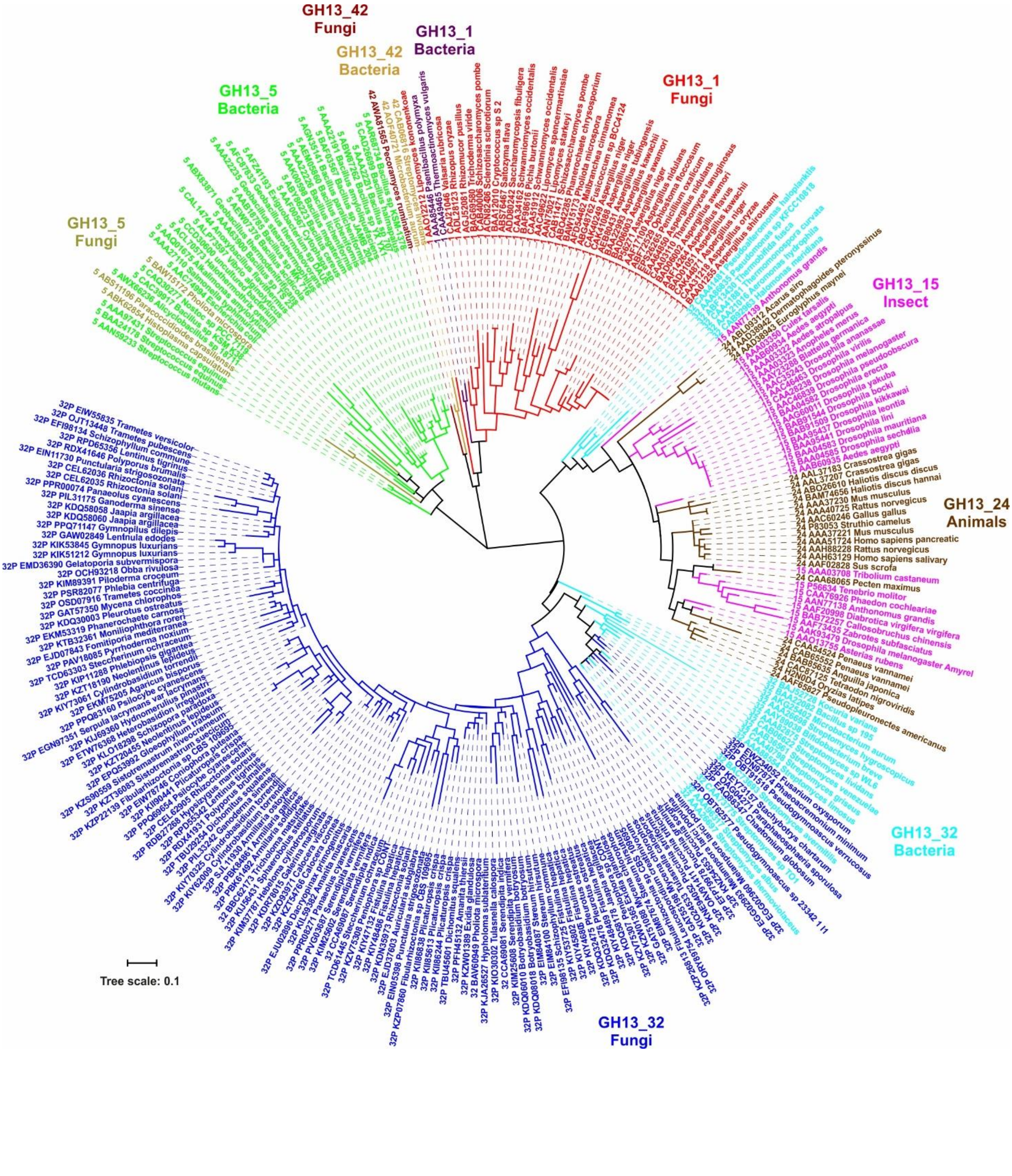

**B**

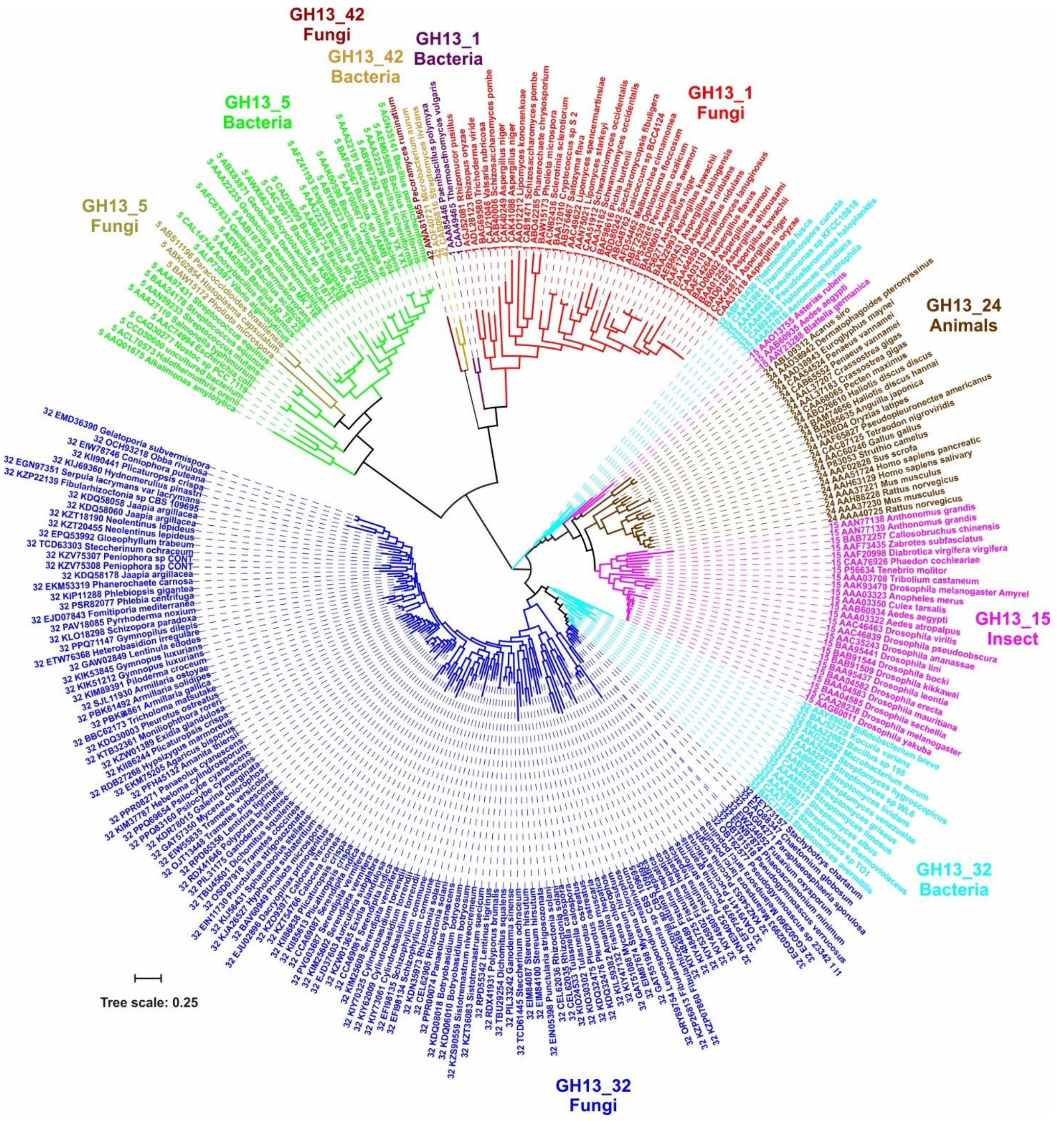


**Figure S2.** Evolutionary trees of studied $\alpha$-amylases based on the alignment of (**A**) sequences spanning the segment between the strands β1 and β8 of catalytic TIM-barrel; and (**B**) extracted eight conserved sequence regions. The same trees without all the leaves described are presented in Figure 2. Details concerning all $\alpha$-amylases compared in the trees as well as their colour codes are given in Table S1. Each protein is labelled by the GH13 subfamily number, the GenBank accession number (or rarely the UniProt accession number) and the name of the organism.

**Table S1**. List of 268 sequences from GH13 subfamilies containing fungal and chloride-dependent $\alpha$-amylases.[a]

| No. | Source | GenBank[b] | UniProt[c] | Length[d] | β1-β8[e] |
|---|---|---|---|---|---|
| | GH13_1 – *Bacteria* | | | | |
| 1 | *Paenibacillus polymyxa* | AAA85446.1 | P21543 | 1196 | 753-1072 |

| | | | | | |
|---|---|---|---|---|---|
| 2 | *Thermoactinomyces vulgaris* | CAA49465.1 | Q60051 | 482 | 44-358 |
| | GH13_1 – *Eucarya* – Fungi | | | | |
| 3 | *Aspergillus awamori* | BAD06003.1 | Q76L96 | 634 | 36-352 |
| 4 | *Aspergillus awamori* | BAD06002.1 | Q76L99 | 499 | 36-352 |
| 5 | *Aspergillus flavus* | AAF14264.1 | Q7LV45 | 499 | 36-352 |
| 6 | *Aspergillus kawachii* | BAD01051.1 | Q76CT3 | 498 | 35-351 |
| 7 | *Aspergillus kawachii* | BAA22993.1 | O13296 | 490 | 35-352 |
| 8 | *Aspergillus nidulans* | EAA64850.1 | A0A1U8QLT9 | 490 | 30-344 |
| 9 | *Aspergillus nidulans* | AAF17100.1 | Q9UV09 | 623 | 34-350 |
| 10 | *Aspergillus niger* | N.A. | P56271 | 484 | 15-331 |
| 11 | *Aspergillus niger* | CAK44871.1 | A2QL05 | 498 | 35-351 |
| 12 | *Aspergillus niger* | CAK40249.1 | A2QTS4 | 555 | 40-359 |
| 13 | *Aspergillus niger* | CAK41088.1 | A2QYT9 | 549 | 43-362 |
| 14 | *Aspergillus oryzae* | CAA31218.1 | P0C1B3 | 499 | 36-352 |
| 15 | *Aspergillus shirousami* | BAA01255.1 | P30292 | 499 | 36-352 |
| 16 | *Aspergillus tubingensis* | AEB80431.1 | G9DA07 | 640 | 36-352 |
| 17 | *Cryptococcus* sp. S-2 | BAA12010.1 | Q92394 | 631 | 35-366 |
| 18 | *Fusicoccum* sp. BCC4124 | ABG48762.1 | Q0Z8K1 | 475 | 13-329 |
| 19 | *Lipomyces kononenkoae* | AAO12212.1 | Q8J1E4 | 499 | 38-356 |
| 20 | *Lipomyces spencermartinsiae* | AAC49622.1 | Q01117 | 624 | 108-424 |
| 21 | *Lipomyces starkeyi* | AAN75021.1 | Q6YF33 | 647 | 185-501 |
| 22 | *Malbranchea cinnamomea* | AFD54462.1 | K9L8F3 | 492 | 36-350 |
| 23 | *Ophiostoma floccosum* | ABF72529.1 | Q06SN2 | 630 | 33-349 |
| 24 | *Penicillium oxalicum* | EPS26265.1 | S7Z6T2 | 626 | 32-348 |
| 25 | *Phanerochaete chrysosporium* | ABO42285.1 | A5Y589 | 530 | 34-348 |
| 26 | *Pholiota microspora* | BAW15173.1 | A0A1L7MPN9 | 535 | 34-351 |
| 27 | *Pichia burtonii* | BAF98616.1 | A9ZPM0 | 494 | 45-358 |
| 28 | *Rhizomucor pusillus* | AGJ52081.1 | M9TI89 | 471 | 48-347 |
| 29 | *Rhizopus oryzae* | ADL28123.1 | E2G4G0 | 462 | 41-340 |
| 30 | *Saccharomycopsis fibuligera* | ADD80242.1 | D4P4Y7 | 494 | 42-358 |
| 31 | *Saitozyma flava* | ABS76467.1 | A7LGW4 | 631 | 35-366 |
| 32 | *Schizosaccharomyces pombe* | CAB11471.1 | O14154 | 513 | 40-352 |
| 33 | *Schizosaccharomyces pombe* | CAB40006.1 | Q9Y7S9 | 564 | 36-356 |
| 34 | *Schwanniomyces occidentalis* | CAA34162.1 | P19269 | 512 | 51-367 |
| 35 | *Schwanniomyces occidentalis* | CAA51912.1 | Q08806 | 507 | 47-363 |
| 36 | *Sclerotinia sclerotiorum* | ACN82436.1 | C0LW29 | 499 | 36-349 |
| 37 | *Thermomyces lanuginosus* | CAA03110.1 | UPI000011D79A | 493 | 33-350 |
| 38 | *Trichoderma viride* | BAG69580.1 | B5BQC3 | 463 | 35-334 |
| 39 | *Valsaria rubricosa* | CAJ21046.1 | UPI000057623F | 493 | 35-332 |
| | GH13_5 – *Bacteria* | | | | |
| 40 | *Alicyclobacillus* sp. 18711 | AWX66236.1 | A0A3P8MUS3 | 514 | 41-399 |
| 41 | *Alkalimonas amylolytica* | AAQ01675.1 | Q6WUB6 | 587 | 50-378 |
| 42 | *Anoxybacillus flavithermus* | CAL14744.1 | UPI0000DAC7C3 | 586 | 13-369 |
| 43 | *Bacillus acidicola* | AEW07376.1 | J9PQD2 | 479 | 9-365 |
| 44 | *Bacillus amyloliquefaciens* | AAA22191.1 | P00692 | 479 | 40-396 |
| 45 | *Bacillus cereus* | ABY86223.1 | B0LVG1 | 513 | 39-395 |
| 46 | *Bacillus halmapalus* | CAD26699.1 | UPI000002EA3C | 485 | 13-370 |
| 47 | *Bacillus licheniformis* | AAA22226.1 | P06278 | 512 | 40-394 |
| 48 | *Bacillus licheniformis* | AEM05860.1 | I3P686 | 483 | 11-365 |
| 49 | *Bacillus megaterium* | AAK00598.1 | Q9AQ54 | 533 | 39-395 |
| 50 | *Bacillus paralicheniformis* | AGN35141.1 | UPI0002A8802F | 512 | 40-394 |
| 51 | *Bacillus* sp. KSM-1378 | AAR68734.1 | O82839 | 516 | 44-401 |
| 52 | *Bacillus* sp. 707 | AAA22231.1 | P19571 | 518 | 46-403 |
| 53 | *Bacillus* sp. JAMB-204 | BAF03567.1 | Q0KKZ7 | 513 | 39-395 |
| 54 | *Bacillus* sp. KSM-K38 | CAC39917.1 | Q93I48 | 501 | 32-386 |
| 55 | *Bacillus* sp. MK 716 | AAB18785.1 | P71034 | 521 | 46-402 |
| 56 | *Bacillus* sp. TS-23 | AAA63900.1 | Q59222 | 613 | 43-399 |
| 57 | *Bacillus* sp. YX-1 | ABW87262.1 | A9YDD9 | 514 | 40-396 |
| 58 | *Cytophaga* sp. | AAF00567.1 | Q9RQT8 | 519 | 45-401 |

| | | | | | |
|---|---|---|---|---|---|
| 59 | *Escherichia coli* | AAC74994.1 | P26612 | 495 | 10-369 |
| 60 | *Exiguobacterium* sp. DAU5 | AFZ41193.1 | M4I6P6 | 514 | 40-396 |
| 61 | *Geobacillus stearothermophilus* | AAA22235.2 | P06279 | 549 | 46-402 |
| 62 | *Geobacillus thermodenitrificans* | ABX83871.1 | A9UJ60 | 549 | 46-402 |
| 63 | *Geobacillus thermoleovorans* | AFC87833.1 | J9PXA2 | 549 | 46-402 |
| 64 | *Halothermothrix orenii* | ACL70573.1 | B8CZ54 | 623 | 153-508 |
| 65 | *Nostoc* sp. PCC 7119 | CAQ30277.1 | B1VK33 | 489 | 9-366 |
| 66 | *Salmonella typhimurium* | AAA27110.1 | P26613 | 494 | 10-369 |
| 67 | *Streptococcus equinus* | BAA24178.1 | O50583 | 484 | 9-367 |
| 68 | *Streptococcus equinus* | AAA97431.1 | Q53786 | 485 | 10-368 |
| 69 | *Streptococcus mutans* | AAN59233.1 | Q8DT08 | 486 | 10-368 |
| 70 | uncultured bacterium | CCD30600.1 | I7I8P3 | 486 | 10-369 |
| 71 | *Vibrio alginolyticus* | ALP73597.1 | A0A0S2UQL4 | 507 | 33-389 |
| | GH13_5 – *Eucarya* – Fungi | | | | |
| 72 | *Histoplasma capsulatum* | ABK62854.1 | A0T074 | 540 | 42-399 |
| 73 | *Paracoccidioides brasiliensis* | ABS11196.1 | A7L832 | 535 | 42-397 |
| 74 | *Pholiota microspora* | BAW15172.1 | A0A1L7MPN7 | 519 | 40-399 |
| | GH13_15 – *Eucarya* | | | | |
| 75 | *Aedes aegypti* | AAB60935.1 | P53354 | 737 | 275-594 |
| 76 | *Aedes aegypti* | AAB60934.1 | O02652 | 486 | 35-345 |
| 77 | *Aedes atropalpus* | AAA03322.1 | Q16924 | 491 | 35-345 |
| 78 | *Anopheles merus* | AAA03323.1 | Q17059 | 514 | 39-356 |
| 79 | *Anthonomus grandis* | AAN77138.1 | Q8I9K6 | 484 | 34-341 |
| 80 | *Anthonomus grandis* | AAN77139.1 | Q8I9K5 | 491 | 35-348 |
| 81 | *Asterias rubens* | AAO13755.1 | Q8IA45 | 492 | 34-345 |
| 82 | *Blattella germanica* | AAY23288.1 | Q2KJQ1 | 498 | 35-351 |
| 83 | *Callosobruchus chinensis* | BAB72257.1 | Q5KTR5 | 489 | 33-344 |
| 84 | *Culex tarsalis* | AAA03350.1 | Q23767 | 496 | 34-350 |
| 85 | *Diabrotica virgifera virgifera* | AAF20998.1 | Q9U406 | 482 | 37-343 |
| 86 | *Drosophila ananassae* | AAC35243.1 | O77407 | 494 | 35-348 |
| 87 | *Drosophila bocki* | BAB91544.1 | Q9NKZ2 | 494 | 35-348 |
| 88 | *Drosophila erecta* | BAA04582.1 | Q23932 | 494 | 35-348 |
| 89 | *Drosophila kikkawai* | BAB91509.1 | Q8MM52 | 494 | 35-348 |
| 90 | *Drosophila leontia* | BAA95437.1 | Q9NKY9 | 494 | 35-348 |
| 91 | *Drosophila lini* | BAA95441.1 | Q9N6Q7 | 494 | 35-348 |
| 92 | *Drosophila mauritiana* | BAA04583.1 | P54215 | 494 | 35-348 |
| 93 | *Drosophila melanogaster* Amyrel | AAK93479.1 | O18408 | 493 | 36-347 |
| 94 | *Drosophila melanogaster* | CAA28238.1 | P08144 | 494 | 35-348 |
| 95 | *Drosophila pseudoobscura* | AAC46839.1 | O46116 | 494 | 35-348 |
| 96 | *Drosophila sechellia* | BAA04585.1 | Q24642 | 494 | 35-348 |
| 97 | *Drosophila virilis* | AAC46463.1 | Q24737 | 494 | 35-348 |
| 98 | *Drosophila yakuba* | AAG60011.1 | P51548 | 494 | 35-348 |
| 99 | *Phaedon cochleariae* | CAA76926.1 | O97396 | 485 | 35-344 |
| 100 | *Tenebrio molitor* | N.A. | P56634 | 471 | 17-326 |
| 101 | *Tribolium castaneum* | AAA03708.1 | Q26854 | 490 | 34-345 |
| 102 | *Zabrotes subfasciatus* | AAF73435.1 | Q9N2P9 | 483 | 34-344 |
| | GH13_24 – *Eucarya* | | | | |
| 103 | *Acarus siro* | ABL09312.1 | B0KZK1 | 517 | 41-360 |
| 104 | *Anguilla japonica* | BAB85635.1 | Q8QGW2 | 512 | 32-357 |
| 105 | *Crassostrea gigas* | AAL37207.1 | Q8WSG9 | 519 | 36-356 |
| 106 | *Crassostrea gigas* | AAL37183.1 | Q8WSH2 | 520 | 36-357 |
| 107 | *Dermatophagoides pteronyssinus* | AAD38942.1 | P49274 | 496 | 18-337 |
| 108 | *Euroglyphus maynei* | AAD38943.1 | Q9Y196 | 521 | 43-362 |
| 109 | *Gallus gallus* | AAC60246.1 | Q98942 | 512 | 32-357 |
| 110 | *Haliotis discus discus* | ABO26610.1 | B6RB08 | 511 | 35-356 |
| 111 | *Haliotis discus hannai* | BAM74656.1 | L8AW48 | 511 | 35-356 |

| | | | | | |
|---|---|---|---|---|---|
| 112 | *Homo sapiens* – pancreas | AAA51724.1 | P04746 | 511 | 32-357 |
| 113 | *Homo sapiens* – saliva | AAH63129.1 | P04745 | 511 | 32-357 |
| 114 | *Mus musculus* | AAA37221.1 | P00687 | 511 | 32-357 |
| 115 | *Mus musculus* – pancreas | AAA37230.1 | P00688 | 508 | 32-354 |
| 116 | *Oryzias latipes* | N.A. | H2N0D4 | 512 | 32-357 |
| 117 | *Pecten maximus* | CAA68065.1 | P91778 | 508 | 35-354 |
| 118 | *Penaeus vannamei* | CAA54524.1 | Q26193 | 512 | 33-357 |
| 119 | *Penaeus vannamei* | CAB65552.2 | Q9U0F9 | 489 | 33-357 |
| 120 | *Pseudopleuronectes americanus* | AAF65827.1 | Q9I9H6 | 512 | 32-357 |
| 121 | *Rattus norvegicus* – pancreas | AAA40725.1 | P00689 | 508 | 32-354 |
| 122 | *Rattus norvegicus* | AAH88228.1 | Q5I0L0 | 511 | 32-357 |
| 123 | *Struthio camelus* | N.A. | P83053 | 497 | 17-342 |
| 124 | *Sus scrofa* – pancreas | AAF02828.1 | P00690 | 511 | 32-357 |
| 125 | *Tetraodon nigroviridis* | CAC87125.1 | Q8UWE5 | 513 | 32-358 |
| | GH13_32 – *Bacteria* | | | | |
| 126 | *Aeromonas hydrophila* | AAA21016.1 | P41131 | 443 | 32-328 |
| 127 | *Bacillus* sp. 195 | BAA22082.1 | O24781 | 700 | 59-357 |
| 128 | *Bifidobacterium breve* | AAY89038.1 | F9Y041 | 1708 | 58-364 |
| 129 | *Halomonas meridiana* | CAB92963.1 | Q9L4I9 | 457 | 27-326 |
| 130 | *Kocuria varians* | BAJ52728.1 | E5RKQ5 | 736 | 93-391 |
| 131 | *Microbacterium aurum* | AKG25402.1 | A0A0G2T4B5 | 1417 | 64-367 |
| 132 | *Pseudoalteromonas haloplanktis* | CAA41481.1 | P29957 | 669 | 33-329 |
| 133 | *Pseudomonas* sp. KFCC10818 | AAA86835.1 | Q52413 | 563 | 37-320 |
| 134 | *Streptomyces albus* | AAA96317.1 | Q53633 | 460 | 42-334 |
| 135 | *Streptomyces avermitilis* | BAC73693.1 | Q82AS5 | 460 | 42-334 |
| 136 | *Streptomyces griseus* | CAA40798.1 | P30270 | 566 | 41-333 |
| 137 | *Streptomyces hygroscopicus* | AAA26698.1 | P08486 | 478 | 43-329 |
| 138 | *Streptomyces limosus* | AAA88554.1 | P09794 | 566 | 41-333 |
| 139 | *Streptomyces lividans* | CAB06622.1 | P97179 | 573 | 48-340 |
| 140 | *Streptomyces* sp. TO1 | CAA73775.1 | O65947 | 453 | 37-328 |
| 141 | *Streptomyces* sp. WL6 | AAA82875.1 | Q59964 | 459 | 44-333 |
| 142 | *Streptomyces thermoviolaceus* | AAA26697.1 | P27350 | 460 | 42-334 |
| 143 | *Streptomyces venezuelae* | AAB36561.1 | P22998 | 569 | 41-333 |
| 144 | *Thermobifida fusca* | ABF13430.1 | Q1KLC8 | 605 | 46-350 |
| 145 | *Thermomonospora curvata* | CAA41881.1 | P29750 | 605 | 46-350 |
| | GH13_32 – *Eucarya* – Fungi | | | | |
| 146 | *Pholiota microspora* | BAV60949.1 | A0A1E1ERR9 | 589 | 51-347 |
| 147 | *Hypholoma sublateritium* | KJA26527.1 | A0A0D2LFX5 | 591 | 47-343 |
| 148 | *Galerina marginata* | KDR78015.1 | A0A067T4C3 | 589 | 48-344 |
| 149 | *Psilocybe cyanescens* | PPQ83160.1 | A0A409WXA0 | 586 | 47-341 |
| 150 | *Moniliophthora roreri* | KTB32361.1 | A0A0W0F7R2 | 585 | 44-340 |
| 151 | *Pleurotus ostreatus* | KDQ30003.1 | A0A067NPJ8 | 587 | 47-343 |
| 152 | *Polyporus brumalis* | RDX41646.1 | A0A371CMZ2 | 584 | 48-344 |
| 153 | *Trametes pubescens* | OJT13448.1 | A0A1M2W0S7 | 597 | 52-348 |
| 154 | *Amanita thiersii* | PFH45132.1 | A0A2A9NBQ2 | 588 | 52-349 |
| 155 | *Hebeloma cylindrosporum* | KIM37787.1 | A0A0C2Y9T3 | 580 | 44-340 |
| 156 | *Armillaria solidipes* | PBK61492.1 | A0A2H3B359 | 573 | 36-330 |
| 157 | *Heterobasidion irregulare* | ETW76368.1 | W4JS51 | 545 | 2-298 |
| 158 | *Hypsizygus marmoreus* | RDB27268.1 | A0A369JZU5 | 587 | 48-344 |
| 159 | *Gymnopilus dilepis* | PPQ71147.1 | A0A409VY19 | 654 | 108-404 |
| 160 | *Trametes coccinea* | OSD07916.1 | A0A1Y2J3F4 | 577 | 50-334 |
| 161 | *Exidia glandulosa* | KZW01389.1 | A0A165NYB6 | 595 | 53-348 |
| 162 | *Trametes versicolor* | EIW55835.1 | N.A. | 598 | 52-348 |
| 163 | *Armillaria ostoyae* | SJL11930.1 | A0A284RT72 | 591 | 36-348 |
| 164 | *Pyrrhoderma noxium* | PAV18085.1 | A0A286UF06 | 1482 | 426-722 |
| 165 | *Lentinus tigrinus* | RPD65356.1 | A0A5C2SVV8 | 593 | 51-347 |
| 166 | *Armillaria gallica* | PBK84861.1 | A0A2H3CP67 | 565 | 36-324 |
| 167 | *Schizopora paradoxa* | KLO18298.1 | A0A0H2S996 | 582 | 42-341 |
| 168 | *Panaeolus cyanescens* | PPR08271.1 | A0A409YZ37 | 590 | 43-341 |

| | | | | | |
|---|---|---|---|---|---|
| 169 | *Gymnopus luxurians* | KIK53845.1 | A0A0D0ATN4 | 598 | 54-341 |
| 170 | *Sphaerobolus stellatus* | KIJ56431.1 | N.A. | 542 | 9-307 |
| 171 | *Fomitiporia mediterranea* | EJD07843.1 | N.A. | 646 | 105-398 |
| 172 | *Gloeophyllum trabeum* | EPQ53992.1 | S7Q3A2 | 569 | 34-330 |
| 173 | *Phlebiopsis gigantea* | KIP11288.1 | A0A0C3PUH0 | 577 | 44-340 |
| 174 | *Cylindrobasidium torrendii* | KIY62009.1 | A0A0D7AVY0 | 589 | 35-333 |
| 175 | *Hydnomerulius pinastri* | KIJ69360.1 | N.A. | 568 | 35-332 |
| 176 | *Neolentinus lepideus* | KZT20455.1 | A0A165P365 | 574 | 39-335 |
| 177 | *Serpula lacrymans* | EGN97351.1 | F8Q2E0 | 585 | 46-349 |
| 178 | *Botryobasidium botryosum* | KDQ06010.1 | A0A067LUS3 | 588 | 46-342 |
| 179 | *Panaeolus cyanescens* | PPR00074.1 | A0A409YAP2 | 590 | 46-341 |
| 180 | *Phanerochaete carnosa* | EKM53319.1 | K5W345 | 577 | 43-340 |
| 181 | *Dichomitus squalens* | TBU45601.1 | A0A4Q9NZH8 | 572 | 51-347 |
| 182 | *Phlebia centrifuga* | PSR82077.1 | A0A2R6P097 | 555 | 46-312 |
| 183 | *Piloderma croceum* | KIM89391.1 | A0A0C3GFG2 | 598 | 58-359 |
| 184 | *Ganoderma sinense* | PIL31175.1 | A0A2G8SBN2 | 555 | 50-346 |
| 185 | *Cylindrobasidium torrendii* | KIY70325.1 | A0A0D7BJC3 | 579 | 36-334 |
| 186 | *Jaapia argillacea* | KDQ58060.1 | A0A067PW10 | 650 | 115-411 |
| 187 | *Rhizoctonia solani* | CEL62905.1 | A0A0B7FYW5 | 975 | 38-333 |
| 188 | *Mycena chlorophos* | GAT57350.1 | A0A146IBV9 | 748 | 143-439 |
| 189 | *Serendipita vermifera* | KIM25608.1 | A0A0C2WGV7 | 593 | 40-336 |
| 190 | *Lentinula edodes* | GAW02849.1 | A0A1Q3E749 | 669 | 60-357 |
| 191 | *Steccherinum ochraceum* | TCD63303.1 | A0A4R0R9L4 | 551 | 13-312 |
| 192 | *Fibularhizoctonia* sp. CBS 109695 | KZP22139.1 | A0A166KQ11 | 580 | 42-340 |
| 193 | *Plicaturopsis crispa* | KII90441.1 | N.A. | 584 | 45-347 |
| 194 | *Jaapia argillacea* | KDQ58058.1 | A0A067PTB3 | 573 | 22-333 |
| 195 | *Obba rivulosa* | OCH93218.1 | UPI000DC9AC67 | 571 | 41-334 |
| 196 | *Gelatoporia subvermispora* | EMD36390.1 | M2QWA3 | 569 | 40-332 |
| 197 | *Coniophora puteana* | EIW78746.1 | A0A5M3MIB8 | 569 | 32-327 |
| 198 | *Plicaturopsis crispa* | KII86244.1 | N.A. | 501 | 43-365 |
| 199 | *Agaricus bisporus* | EKM75205.1 | K5XL16 | 474 | 42-338 |
| 200 | *Sistotremastrum suecicum* | KZT36083.1 | A0A166B801 | 589 | 45-348 |
| 201 | *Sistotremastrum niveocremeum* | KZS90559.1 | A0A164RHE5 | 589 | 45-348 |
| 202 | *Schizophyllum commune* | EFI98134.1 | D8Q2K0 | 470 | 38-334 |
| 203 | *Tricholoma matsutake* | BBC62173.1 | A0A2Z5Y5L0 | 491 | 62-358 |
| 204 | *Serendipita indica* | CCA69081.1 | G4TCP2 | 570 | 24-316 |
| 205 | *Schizophyllum commune* | EFI98135.1 | D8Q2K1 | 482 | 49-345 |
| 206 | *Plicaturopsis crispa* | KII85613.1 | N.A. | 782 | 58-355 |
| 207 | *Punctularia strigosozonata* | EIN11730.1 | N.A. | 477 | 44-341 |
| 208 | *Ganoderma sinense* | PIL33242.1 | A0A2G8SHK1 | 483 | 48-342 |
| 209 | *Neolentinus lepideus* | KZT18190.1 | A0A165MD72 | 510 | 42-338 |
| 210 | *Peniophora* sp. CONT | KZV75308.1 | A0A166K504 | 485 | 57-351 |
| 211 | *Plicaturopsis crispa* | KII86838.1 | N.A. | 509 | 33-330 |
| 212 | *Botryobasidium botryosum* | KDQ08018.1 | A0A067LXA2 | 483 | 47-345 |
| 213 | *Cylindrobasidium torrendii* | KIY73061.1 | A0A0D7BTW3 | 469 | 37-333 |
| 214 | *Exidia glandulosa* | KZW01365.1 | A0A165NXQ6 | 594 | 29-327 |
| 215 | *Jaapia argillacea* | KDQ58178.1 | A0A067PTN6 | 729 | 160-457 |
| 216 | *Peniophora* sp. CONT | KZV75307.1 | A0A166K4Z3 | 474 | 45-341 |
| 217 | *Gymnopus luxurians* | KIK51212.1 | A0A0D0C0R2 | 475 | 43-340 |
| 218 | *Polyporus brumalis* | RDX41931.1 | A0A371CNS9 | 429 | 2-295 |
| 219 | *Dichomitus squalens* | TBU29254.1 | A0A4Q9MNH0 | 436 | 2-298 |
| 220 | *Fistulina hepatica* | KIY45802.1 | A0A0D7A480 | 566 | 30-329 |
| 221 | *Amanita muscaria* | KIL59382.1 | A0A0C2WDE5 | 515 | 46-344 |
| 222 | *Fistulina hepatica* | KIY53725.1 | A0A0D7APG0 | 558 | 8-315 |
| 223 | *Fistulina hepatica* | KIY47132.1 | A0A0D7A7Y2 | 549 | 10-308 |
| 224 | *Lentinus tigrinus* | RPD55342.1 | A0A5C2RUP1 | 429 | 2-295 |
| 225 | *Steccherinum ochraceum* | TCD61445.1 | A0A4R0R8J3 | 477 | 51-349 |
| 226 | *Serendipita vermifera* | KIM25602.1 | A0A0C2X8T3 | 514 | 45-341 |
| 227 | *Auricularia subglabra* | EJD37603.1 | J0WW10 | 567 | 2-304 |
| 228 | *Calocera cornea* | KZT54766.1 | A0A165EFL8 | 539 | 69-364 |
| 229 | *Fistulina hepatica* | KIY46805.1 | A0A0D7A7V0 | 479 | 40-338 |
| 230 | *Psilocybe cyanescens* | PPQ69654.1 | A0A409VTS4 | 367 | 47-343 |
| 231 | *Calocera viscosa* | KZO93971.1 | A0A167JVZ9 | 472 | 2-297 |

| | | | | | |
|---|---|---|---|---|---|
| 232 | *Fibularhizoctonia* sp. CBS 109695 | KZP07860.1 | A0A165WS59 | 590 | 45-352 |
| 233 | *Serendipita vermifera* | PVG03687.1 | N.A. | 536 | 46-362 |
| 234 | *Stereum hirsutum* | EIM87674.1 | N.A. | 600 | 41-343 |
| 235 | *Serendipita indica* | CCA69087.1 | G4TCP6 | 486 | 2-298 |
| 236 | *Rhizoctonia solani* | CEL62035.1 | A0A0B7FWG9 | 479 | 48-343 |
| 237 | *Punctularia strigosozonata* | EIN05398.1 | N.A. | 442 | 12-307 |
| 238 | *Dacryopinax primogenitus* | EJU02896.1 | M5FXX0 | 522 | 42-341 |
| 239 | *Tulasnella calospora* | KIO30302.1 | A0A0C3M9C4 | 444 | 2-298 |
| 240 | *Rhizoctonia solani* | CEL62036.1 | A0A0B7FVR4 | 477 | 48-341 |
| 241 | *Tulasnella calospora* | KIO24533.1 | A0A0C3KSS6 | 476 | 44-338 |
| 242 | *Stereum hirsutum* | EIM84100.1 | N.A. | 475 | 44-339 |
| 243 | *Rhizoctonia solani* | KDN35973.1 | A0A066V6S6 | 451 | 2-297 |
| 244 | *Fistulina hepatica* | KIY48489.1 | A0A0D7ADK8 | 447 | 16-311 |
| 245 | *Fistulina hepatica* | KIY48486.1 | A0A0D7AF22 | 459 | 27-322 |
| 246 | *Fibularhizoctonia* sp. CBS 109695 | KZP26813.1 | A0A166Q6M7 | 430 | 2-291 |
| 247 | *Pleurotus ostreatus* | KDQ32476.1 | A0A067P9A6 | 466 | 50-352 |
| 248 | *Stereum hirsutum* | EIM84087.1 | N.A. | 477 | 45-341 |
| 249 | *Pleurotus ostreatus* | KDQ32475.1 | A0A067NZR1 | 458 | 38-342 |
| 250 | *Chaetomium globosum* | EAQ88347.1 | Q2GZT0 | 458 | 40-332 |
| 251 | *Leucosporidium creatinivorum* | ORY89754.1 | A0A1Y2G021 | 594 | 45-339 |
| 252 | *Fusarium oxysporum* | EWZ34052.1 | W9JPP9 | 456 | 38-330 |
| 253 | *Pseudogymnoascus verrucosus* | OBT91518.1 | A0A1B8G6R5 | 459 | 41-333 |
| 254 | *Stachybotrys chartarum* | KEY73157.1 | A0A084B6H8 | 458 | 40-342 |
| 255 | *Pseudogymnoascus* sp. 23342-1-I1 | OBT62577.1 | A0A1B8DUF8 | 459 | 41-333 |
| 256 | *Phaeoacremonium minimum* | EON97874.1 | R8BEZ8 | 461 | 43-335 |
| 257 | *Mycena chlorophos* | GAT55198.1 | A0A146I682 | 846 | 405-708 |
| 258 | *Paraphaeosphaeria sporulosa* | OAG04271.1 | A0A177CAS9 | 458 | 40-332 |
| 259 | *Melampsora larici populina* | EGG02960.1 | F4RXF9 | 500 | 72-375 |
| 260 | *Melampsora larici populina* | EGG02993.1 | F4RXG2 | 462 | 34-337 |
| 261 | *Puccinia triticina* | OAV91411.1 | A0A180GFL8 | 516 | 77-380 |
| 262 | *Puccinia graminis* | EFP79978.1 | UPI000251D20C | 574 | 85-388 |
| 263 | *Mycena chlorophos* | GAT51088.1 | A0A146HU33 | 1085 | 44-377 |
| 264 | *Puccinia striiformis* | KNE94052.1 | A0A0L0V451 | 535 | 102-397 |
| 265 | *Puccinia sorghi* | KNZ54553.1 | A0A0L6V192 | 666 | 109-477 |
| | GH13_42 – *Bacteria* | | | | |
| 266 | *Microbacterium aurum* | AOF40721.1 | A0A1B3IKE0 | 1278 | 658-1112 |
| 267 | *Streptomyces lividans* | CAB06816.1 | P96992 | 993 | 369-827 |
| | GH13_42 – *Eucarya* – Fungi | | | | |
| 268 | *Pecoramyces ruminatium* | AWA81565.1 | A0A2S0S3D2 | 975 | 379-805 |

[a] Colour code: GH13_1: plum – bacteria, red – fungi; GH13_5: green – bacteria, olive – fungi; GH13_15: magenta – eukaryotes; GH13_24: walnut – eukaryotes; GH13_32: cyan – bacteria, blue – fungi; GH13_42: gold – *Bacteria*, ruby red – fungi. Sequences No. 146-265 of fungal α-amylases from the subfamily GH13_32 were collected based on the CAZy database and BLAST search using the sequence of *Pholiota microspora* α-amylase as a query.
[b] GenBank accession No.
[c] UniProt accession No.
[d] Length of the protein in amino acid residues.
[e] Segment of the protein from the strand β1 to the end of the strand β8 of the catalytic TIM-barrel including domain B.
